# Synthesis of BODIPY FL-tethered ridaifen-B, RID-B-BODIPY, and its localization in cancer cells

**DOI:** 10.3389/fchem.2024.1451468

**Published:** 2024-08-23

**Authors:** Takatsugu Murata, Kyoka Komukai, Yuta Semba, Eri Murata, Fumi Sato, Tomohiro Takano, Kaho Tsuchiya, Chihiro Matsuda, Anju Sakai, Amane Yoneoka, Shunsuke Takahashi, Yukitoshi Nagahara, Isamu Shiina

**Affiliations:** ^1^ Department of Applied Chemistry, Faculty of Science, Tokyo University of Science, Tokyo, Japan; ^2^ Division of Life Science and Engineering, College of Science and Engineering, Tokyo Denki University, Saitama, Japan

**Keywords:** MNBA, BODIPY FL, dehydration condensation, BODIPY, ridaifen

## Abstract

We synthesized ridaifen-B boron dipyrromethene (**RID-B-BODIPY**) using 2-methyl-6-nitro benzoic anhydride (MNBA)-mediated dehydration condensation reaction between amino alkyl-tethered RID and BODIPY FL. Comparative experiments between dicyclohexylcarbodiimide (DCC) and MNBA for their coupling reactions demonstrated that MNBA is an effective condensation reagent for amines and BODIPY FL. A cell staining study with **RID-B-BODIPY** showed intracellular localization of BODIPY FL fluorescence, attributed to the **RID-B** structure, indicating the successful development of a tool for analyzing intracellular molecular behavior efficiently.

## 1 Introduction

**FIGURE 1:**
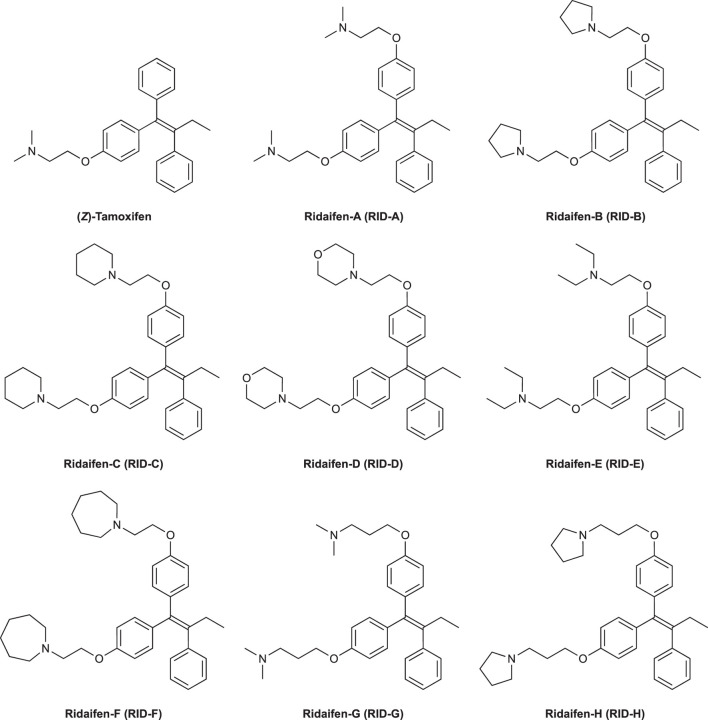
Chemical structures of (*Z*)-tamoxifen and RID-A–H.

Boron dipyrromethene (BODIPY) is widely recognized as a fluorescent core for sensing the localization of target molecules in biological spaces (Loudet and Burgess, 2007). To image the localization of a bioactive molecule, carboxyl group-possessing BODIPYs such as BODIPY FL (Barsony et al., 1995), BODIPY R6G (Metzker et al., 1996), BODIPY TMR (Meltola et al., 2004), BODIPY 581/591, BODIPY TR, BODIPY 630/650, and BODIPY 650/665 are often conjugated with the bioactive molecules. Particularly, BODIPY FL is suitable for linkage to the target bioactive molecules (Krajcovicova et al., 2018; Brandes et al., 2020).

(*Z*)-Tamoxifen^®^ (TAM), a selective estrogen receptor modulator, is used for treating estrogen-dependent breast cancer (Bedford and Richardson, 1966). Ridaifens (RIDs) share a structural similarity with TAM (Figure 1), but unlike TAM, they do not possess geometric isomers (Shiina et al., 2007). TAM often needs to be separated with the stereoisomers or to be synthesized over many steps (Tandon et al., 2020). Consequently, RIDs are more synthetically accessible from commercially available reagents than tamoxifen^®^. **RID-A** has the same side chain as TAM. **RID-B–H** have nafoxidine-type side chains, laroxifen-type side chains, morpholine-type side chains, clomiphene-type side chains, bazedoxifene-type side chains, dimethylaminopropyl-type side chains, and pyrrolidinylpropyl-type side chains, respectively. Previous studies have suggested that RIDs operate through a pharmacological mechanism distinct from that of TAM. Notably, **RID-B** exhibits superior anti-proliferative activity against cancer cells than TAM and other RIDs. Furthermore, **RID-B** is effective against estrogen receptor-negative cells, which TAM does not significantly affect (Nagahara et al., 2008). The two aminoalkoxy side chains of **RID-B** are necessary for showing high cytotoxicity (Iwasawa et al., 2019). Furthermore, it was recently reported that **RID-B** could act as a pan-filovirus inhibitor (Cooper et al., 2020). Thus, the bioactivity of RIDs has drawn the attention of biologists, and a detailed pharmacological study of **RID-B** has been pursued. Our ongoing investigations into the mechanism of **RID-B** using biological methodologies indicate its expected localization within small organelles such as mitochondria and/or lysosomes (Nagahara et al., 2013; Tsukuda et al., 2013).

**FIGURE 2:**
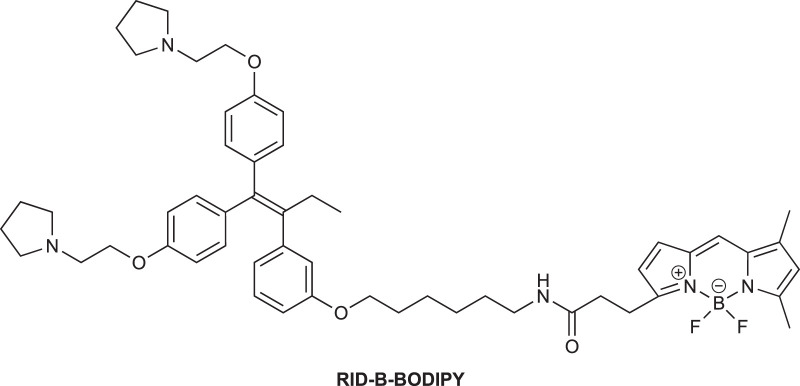
Chemical structure of RID-B-BODIPY.

To elucidate the localization of **RID-B**, we endeavored to synthesize **RID-B-BODIPY**, where fluorescent **BODIPY FL** was conjugated with **RID-B** (Figure 2). Furthermore, this study explores the MNBA-mediated dehydration condensation reaction between an amine and **BODIPY FL**, where traditionally, **BODIPY FL** has been utilized as an NHS-ester rather than a free carboxylic acid for bioconjugation.

## 2 Materials and methods

### 2.1 Chemicals and reagents

Flash column chromatography was performed on CHROMATOREX^®^ PSQ 60B (60 μm) or silica gel 60 (35–70 μm). CHROMATOREX^®^ PSQ 60B was purchased from Fuji Silysia Chemical Ltd. and used as received. Silica gel 60 was purchased from Merck KGaA and used as received. Open column chromatography was performed on Silica gel 60 (63–200 μm). Thin-layer chromatography was performed on Wakogel B5F using UV light as the visualizing agent and modified phosphomolybdic acid and heat as the developing agent. The thin layer chromatography kit was purchased from FUJIFILM Wako Pure Chemical Corp.

The human leukemia T-cell line Jurkat [Clone E6.1; obtained from the American Type Culture Collection (Manassas, VA, United States)] and the human cholangiocarcinoma cell line HuCCT1 [Cell Resource Center for Biomedical Research, Tohoku University (Sendai, Japan)] were used to investigate biological activity. Both cell lines were cultured in RPMI 1640 medium (Shimadzu Diagnostics, Tokyo, Japan) supplemented with 10% fetal bovine serum (Nichirei, Tokyo, Japan) and 75 mg/L kanamycin sulfate at 37°C in a 5% CO_2_ atmosphere.

### 2.2 Synthesis of RID-B-BODIPY

#### 2.2.1 3-(1′,1′-Bis(4″-(2‴-(pyrrolidin-1‴ ′-yl)ethoxy)phenyl)but-1′-en-2′-yl)phenol (RID-B-OH)


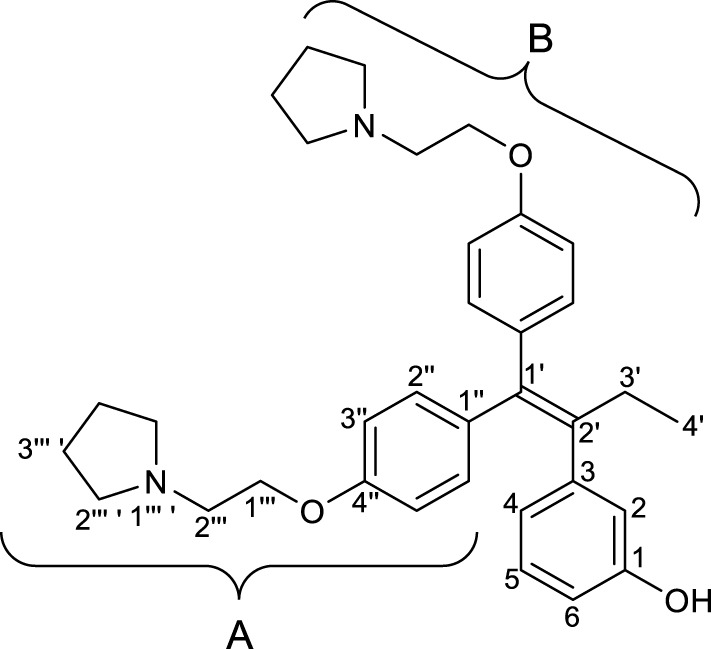


To a solution of **RID-B-OBn** (247.5 mg, 0.401 mmol) in ethyl acetate (13.4 mL), palladium 10% on carbon (M) (170.8 mg, 0.160 mmol) was added. The reaction mixture was stirred for 3 h at room temperature under an atmosphere of hydrogen (1.0 atm), and then transferred to an atmosphere of argon. After filtration of the mixture through a short pad of celite with ethyl acetate and concentration of the solvent, the residue was purified by preparative thin layer chromatography (eluant; ammoniacal chloroform/methanol = 9/1, *R*_f_: 0.50) to give **RID-B-OH** (201.5 mg, 95%) as a yellow solid.

*R*_f_: 0.50 (silica gel, ammoniacal chloroform/methanol = 9/1, UV active; stains blue with modified phosphomolybdic acid);

mp: 57.7°C;

ATR-IR *ν*_max_: 2,954, 2,785, 1,600, 1,505, 1,238, 1,031 cm^−1^;

^1^H NMR (500 MHz, CDCl_3_): *δ* 7.10 (d, *J* = 8.5 Hz, 2H, ^B^H-2″), 7.06–6.92 (m, 1H, H-5), 6.86 (d, *J* = 8.5 Hz, 2H, ^B^H-3″), 6.76 (d, *J* = 8.5 Hz, 2H, ^A^H-2″), 6.64 (d, *J* = 7.5 Hz, 1H, H-2), 6.60–6.43 (m, 4H, H-4, H-6, ^A^H-3″), 4.13 (t, *J* = 6.5 Hz, 2H, ^B^H-1‴), 3.94 (t, *J* = 6.0 Hz, 2H, ^A^H-1‴), 2.93 (t, *J* = 6.5 Hz, 2H, ^B^H-2‴), 2.82 (t, *J* = 6.0 Hz, 2H, ^A^H-2‴), 2.72–2.59 (m, 4H, ^B^H-2‴ ′), 2.64–2.55 (m, 4H, ^A^H-2‴ ′), 2.41 (q, *J* = 7.5 Hz, 2H, H-3′), 1.88–1.75 (m, 4H, ^B^H-3‴ ′), 1.83–1.72 (m, 4H, ^A^H-3‴ ′), 0.89 (t, *J* = 7.5 Hz, 3H, H-4′);

^13^C{^1^H} NMR (125 MHz, CDCl_3_): *δ* 157.5 (C-1), 156.8 (^B^C-4″), 156.7 (^A^C-4″), 144.3 (C-3), 141.1 (C-2′), 137.5 (C-1′), 136.6 (^B^C-1″), 136.0 (^A^C-1″), 131.9 (^B^C-2″), 130.7 (^A^C-2″), 129.1 (C-5), 121.2 (C-2), 117.1 (C-4), 114.1 (^B^C-3″), 113.7 (^A^C-3″), 113.4 (C-6), 66.8 (^B^C-1‴), 66.1 (^A^C-1‴), 55.2 (^B^C-2‴), 55.0 (^A^C-2‴), 54.8 (^B^C-2‴ ′), 54.6 (^A^C-2‴ ′), 29.3 (C-3′), 23.5 (^B^C-3‴ ′), 23.4 (^A^C-3‴ ′), 13.8 (C-4′);

HRMS: *m/z* [M + H]^+^ calcd for C_34_H_43_N_2_O_3_: 527.3268; found: 527.3286.

#### 2.2.2 6-((*tert*-Butoxycarbonyl)amino)hexyl methanesulfonate (Spacer)


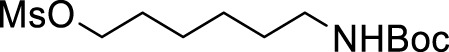


To a solution of *tert*-butyl (6-hydroxyhexyl)carbamate (200.6 mg, 0.923 mmol) in dichloromethane (9.23 mL), triethylamine (0.772 mL, 5.54 mmol) and methanesulfonyl chloride (0.214 mL, 2.77 mmol) were successively added at 0°C. After the reaction mixture was stirred for 1 h at room temperature, the reaction was quenched by a saturated sodium hydrogen carbonate. Two layers were separated, and the aqueous layer was extracted with dichloromethane. The combined organic layers were washed with brine and dried over sodium sulfate. After filtration of the mixture and concentration of the solvent, the residue was purified by silica gel column chromatography (eluant; hexane/ethyl acetate = 3/1) to give **Spacer** (261 mg, 96%) as a white solid.

*R*_f_: 0.20 (silica gel, hexane/ethyl acetate = 2/1, UV active; stains blue with modified phosphomolybdic acid);

mp: 41.0°C (lit.^7^ mp: 44°C–45°C);

ATR-IR *ν*_max_: 3,404, 2,974, 2,862, 1,696, 1,452, 1,352, 1,173 cm^−1^;

^1^H NMR (500 MHz, DMSO-*d*_6_): *δ* 6.86–6.67 (m, 1H, NH), 4.17 (t, *J* = 6.5 Hz, 2H, H-1), 3.15 (s, 3H, MeS), 2.89 (dt, *J* = 6.5, 6.5 Hz, 2H, H-6), 1.64 (tt, *J* = 7.0, 6.5 Hz, 2H, H-5), 1.37 (s, 9H, C(C*H*_*3*_)_3_), 1.38–1.21 (m, 6H, H-2, H-3, H-4);

^13^C{^1^H} NMR (125 MHz, DMSO-*d*_6_): *δ* 155.6 (C=O), 77.3 (*C*(CH_3_)_3_), 70.4 (C-1), 36.5 (MeS), 31.5 (CN), 29.3 (C-5), 28.5 (C-2), 28.3 (C(*C*H_3_)_3_), 25.7 (C-4), 24.6 (C-3);

HRMS: *m*/*z* [M + Na]^+^ calcd for C_12_H_25_NO_5_SNa: 318.1346; found: 318.1353.

#### 2.2.3 *tert*-Butyl (6-(3′-(1″,1″-bis(4‴-(2‴ ′-(pyrrolidin-1‴ ″-yl)ethoxy)phenyl)but-1″-en-2″-yl)phenoxy)hexyl)carbamate (1)


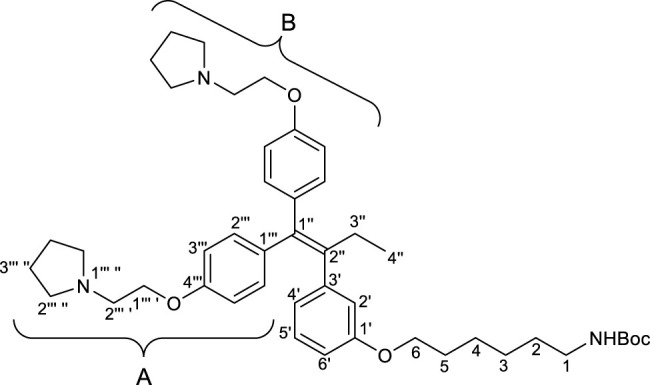


To a solution of **RID-B-OH** (246.9 mg, 0.469 mmol) in *N*,*N*-dimethylformamide (7.81 mL), 55% sodium hydride (dispersion in paraffin liquid, 40.9 mg, 0.938 mmol) and **Spacer** (275.1 mg, 0.938 mmol) were successively added at 0°C. After the reaction mixture was stirred for 2 h at room temperature. The reaction was quenched by brine. Two layers were separated, and the aqueous layer was extracted with diethyl eater. The combined organic layers were dried over sodium sulfate. After filtration of the mixture and concentration of the solvent, the residue was purified by preparative thin layer chromatography (eluant; ammoniacal chloroform/methanol = 9/1, *R*_f_: 0.95) to give crude of **1**. The crude was purified by preparative thin layer chromatography (eluant; chloroform/methanol = 9/1, *R*_f_: 0.40) to give **1** (267.8 mg, 79%) as a pale yellow oil.

*R*_f_: 0.40 (silica gel, chloroform/methanol = 9/1, UV active; stains blue with modified phosphomolybdic acid);

FT-IR (neat) *ν*_max_: 3,379, 3,039, 2,962, 2,931, 2,792, 1,075, 1,604, 1,512, 1,242, 1,173, 1,041, 833 cm^−1^;

^1^H NMR (500 MHz, CDCl_3_): *δ* 7.17–7.05 (m, 2H, ^B^H-2‴), 7.10–7.01 (m, 1H, H-5′), 6.94–6.84 (m, 2H, ^B^H-3‴), 6.81–6.72 (m, 2H, ^A^H-2‴), 6.68 (d, *J* = 7.5 Hz, 1H, H-2′), 6.68–6.57 (m, 2H, H-4′, H-6′), 6.62–6.50 (m, 2H, ^A^H-3‴), 4.69 (brs, 1H, NH), 4.12 (t, *J* = 6.5 Hz, 2H, ^B^H-1‴ ′), 3.96 (t, *J* = 6.0 Hz, 2H, ^A^H-1‴ ′), 3.77 (t, *J* = 6.5 Hz, 2H, H-6), 3.18–3.04 (m, 2H, H-1), 2.91 (t, *J* = 6.5 Hz, 2H, ^B^H-2‴ ′), 2.81 (t, *J* = 6.0 Hz, 2H, ^A^H-2‴ ′), 2.68–2.56 (brm, 4H, ^B^H-2‴ ″), 2.63–2.51 (brm, 4H, ^A^H-2‴ ″), 2.46 (q, *J* = 7.5 Hz, 2H, H-3″), 1.86–1.76 (m, 4H, ^B^H-3‴ ″), 1.81–1.72 (m, 4H, ^A^H-3‴ ″), 1.67 (tt, *J* = 7.0, 6.5 Hz, 2H, H-5), 1.59–1.23 (m, 15H, H-2, H-3, H-4, C(C*H*_*3*_)_3_), 0.93 (t, *J* = 7.5 Hz, 3H, H-4″);

^13^C{^1^H} NMR (125 MHz, CDCl_3_): *δ* 158.7 (C-1′), 157.7 (^B^C-4‴), 156.9 (^A^C-4‴), 156.2 (C=O), 144.1 (C-3′), 141.0 (C-2″), 137.9 (C-1″), 136.4 (^B^C-1‴), 136.1 (^A^C-1‴), 132.0 (^B^C-2‴), 130.7 (^A^C-2‴), 128.8 (C-5′), 122.3 (C-2′), 116.0 (C-4′), 114.2 (^B^C-3‴), 113.5 (^A^C-3‴), 112.6 (C-6′), 79.1 (*C*(CH_3_)_3_), 67.7 (C-6), 67.1 (^B^C-1‴ ′), 66.9 (^A^C-1‴ ′), 55.30 (^B^C-2‴ ′), 55.25 (^A^C-2‴ ′), 54.9 (^B^C-2‴ ″), 54.8 (^A^C-2‴ ″), 40.7 (C-1), 30.2 (C-2), 29.2 (C-5), 29.1 (C-3″), 28.6 (C(*C*H_3_)_3_), 26.7 (C-3), 25.9 (C-4), 23.63 (^B^C-3‴ ″), 23.59 (^A^C-3‴ ″), 13.8 (C-4″);

HRMS: *m/z* [M + H]^+^ calcd for C_45_H_64_N_3_O_5_: 726.4840; found: 726.4816.

#### 2.2.4 6-(3′-(1″,1″-Bis(4‴-(2‴ ′-(pyrrolidin-1‴ ″-yl)ethoxy)phenyl)but-1″-en-2″-yl)phenoxy)hexan-1-amine (2)


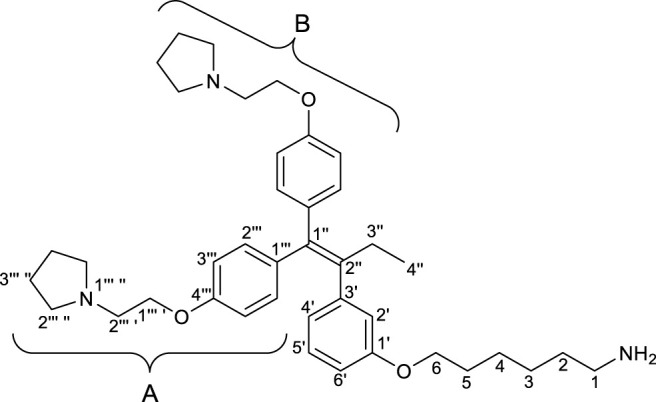


To a solution of **1** (66.7 mg, 0.0919 mmol) in dichloromethane (0.92 mL), methanesulfonic acid (59.6 μ L, 0.919 mmol) was added at 0°C. After the reaction mixture stirred for 30 min, the reaction was quenched by a saturated aqueous sodium hydrogen carbonate. Two layers were separated, and the aqueous layer was extracted with dichloromethane. The combined organic layers were dried over sodium sulfate. After filtration of the mixture and concentration of the solvent, the residue was purified by preparative thin layer chromatography (eluant; ammoniacal chloroform/methanol = 30/2, *R*_f_: 0.50) to give **2** (56.7 mg, 99%) as a pale yellow oil.

*R*_f_: 0.50 (silica gel, ammoniacal chloroform/methanol = 30/2, UV active; stains blue with modified phosphomolybdic acid);

FT-IR (neat) *ν*_max_: 3,371, 3,039, 2,931, 2,785, 1,604, 1,504, 1,242, 1,173, 1,041, 833 cm−1;

^1^H NMR (500 MHz, CDCl_3_): *δ* 7.18–7.04 (m, 2H, ^B^H-2‴), 7.10–6.99 (m, 1H, H-5′), 6.94–6.82 (m, 2H, ^B^H-3‴), 6.83–6.71 (m, 2H, ^A^H-2‴), 6.67 (d, *J* = 7.5 Hz, 1H, H-2′), 6.68–6.58 (m, 2H, H-4′, H-6′), 6.63–6.49 (m, 2H, ^A^H-3‴), 4.12 (t, *J* = 6.0 Hz, 2H, ^B^H-1‴ ′), 3.97 (t, *J* = 6.5 Hz, 2H, ^A^H-1‴ ′), 3.78 (t, *J* = 6.5 Hz, 2H, H-6), 2.91 (t, *J* = 6.0 Hz, 2H, ^B^H-2‴ ′), 2.82 (t, *J* = 6.5 Hz, 2H, ^A^H-2‴ ′), 2.80–2.65 (m, 2H, H-1), 2.74–2.48 (brm, 8H, ^B^H-2‴ ″, ^A^H-2‴ ″), 2.45 (q, *J* = 7.5 Hz, 2H, H-3″), 1.90–1.65 (brm, 8H, ^B^H-3‴ ″, ^A^H-3‴ ″), 1.68 (tt, *J* = 7.5, 6.5 Hz, 2H, H-5), 1.55–1.25 (m, 6H, H-2, H-3, H-4), 0.92 (t, *J* = 7.5 Hz, 3H, H-4″);

^13^C{^1^H} NMR (125 MHz, CDCl_3_): *δ* 158.8 (C-1′), 157.7 (^B^C-4‴), 156.9 (^A^C-4‴), 144.1 (C-3′), 141.0 (C-2″), 137.9 (C-1″), 136.4 (^B^C-1‴), 136.1 (^A^C-1‴), 131.9 (^B^C-2‴), 130.7 (^A^C-2‴), 128.8 (C-5′), 122.3 (C-2′), 115.9 (C-4′), 114.2 (^B^C-3‴), 113.5 (^A^C-3‴), 112.6 (C-6′), 67.8 (C-6), 67.1 (^B^C-1‴ ′), 66.9 (^A^C-1‴ ′), 55.3 (^B^C-2‴ ′), 55.2 (^A^C-2‴ ′), 54.9 (^B^C-2‴ ″), 54.8 (^A^C-2‴ ″), 48.0 (C-1), 29.3 (C-2), 29.2 (C-5), 29.1 (C-3″), 26.8 (C-3), 26.1 (C-4), 23.62 (^B^C-3‴ ″), 23.57 (^A^C-3‴ ″), 13.8 (C-4″);

HRMS: *m/z* [M + H]^+^ calcd for C_40_H_56_N_3_O_3_: 626.4316; found 626.4289.

#### 2.2.5 *N*-(6″-(3‴-(1‴ ′,1‴ ′-Bis(4‴ ″-(2‴ ‴-(pyrrolidin-1‴ ‴ ′-yl)ethoxy)phenyl)but-1‴ ′-en-2‴ ′-yl)phenoxy)hexyl)-3-[4′,4′-difluoro-5′,7′-dimethyl-4′-bora-3′a,4′a-diaza-*s*-indacene-3′-yl]propanamide (RID-B-BODIPY)


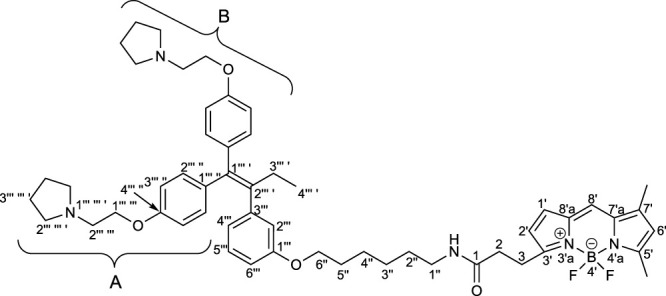


To a solution of 2-methyl-6-nitrobenzoic anhydride (MNBA) (30.0 mg, 0.0872 mmol) in dichloromethane (0.6 mL), *N*,*N*-dimethylpyridin-4-amine (DMAP) (0.9 mg, 0.0727 mmol), triethylamine (22.4 μ L, 0.160 mmol) and **BODIPY FL** (21.2 mg, 0.0727 mmol) was successively added at 0°C. After the reaction mixture was stirred for 10 min, a solution of **2** (54.6 mg, 0.0872 mmol) in dichloromethane (0.4 mL) was added. The reaction mixture was stirred for 3 h at room temperature, the reaction was quenched by a saturated aqueous sodium hydrogen carbonate. Two layers were separated, and the aqueous layer was extracted with dichloromethane. The combined organic layers were dried over sodium sulfate. After filtration of the mixture and concentration of the solvent, the residue was purified by preparative thin layer chromatography (eluant; ammoniacal chloroform/methanol = 30/1, *R*_f_: 0.95) to give **RID-B-BODIPY** (55.4 mg, 85%) as a red solid.

*R*_f_: 0.80 (silica gel, ammoniacal chloroform/methanol = 30/1, UV active; stains blue with modified phosphomolybdic acid);

mp: 51.1°C;

FT-IR (KBr) *ν*_max_: 3,417, 3,309, 3,062, 2,931, 2,870, 1,651, 1,604, 1,242, 1,134 cm−1;

^1^H NMR (500 MHz, CDCl_3_): *δ* 7.20–7.03 (m, 2H, ^B^H-2‴ ″), 7.15–6.93 (m, 2H, H-8′, H-5‴), 6.97–6.77 (m, 3H, H-1′, ^B^H-3‴ ″), 6.85–6.70 (m, 2H, ^A^H-2‴ ″), 6.69 (d, *J* = 8.0 Hz, 1H, H-2‴), 6.70–6.54 (m, 2H, H-4‴, H-6‴), 6.56–6.53 (m, 2H, ^A^H-3‴ ″), 6.29 (d, *J* = 4.0 Hz, 1H, H-2′), 6.10 (s, 1H, H-6′), 5.83 (brt, *J* = 6.0 Hz, 1H, NH), 4.14 (t, *J* = 6.0 Hz, 2H ^B^H-1‴ ‴), 3.96 (t, *J* = 6.0 Hz, 2H, ^A^H-1‴ ‴), 3.74 (t, *J* = 6.5 Hz, 2H, H-6″), 3.27 (t, *J* = 7.5 Hz, 2H, H-3), 3.19 (td, *J* = 6.5, 6.0 Hz, 2H, H-1″), 2.94 (t, *J* = 6.0 Hz, 2H, ^B^H-2‴ ‴), 2.83 (t, *J* = 6.0 Hz, 2H, ^A^H-2‴ ‴), 2.79–2.50 (m, 8H, ^B^H-2‴ ‴ ′, ^A^H-2‴ ‴ ′), 2.63 (brt, *J* = 7.5 Hz, 2H, H-2), 2.56 (s, 3H, 5′-Me), 2.45 (q, *J* = 7.5 Hz, 2H, H-3‴ ′), 2.23 (s, 3H, 7′-Me), 1.95–1.65 (brm, 8H, ^B^H-3‴ ‴ ′, ^A^H-3‴ ‴ ′), 1.61 (tt, *J* = 7.5, 6.5 Hz, 2H, H-5″), 1.55–1.20 (m, 4H, H-2″, H-4″), 1.35–1.15 (m, 2H, H-3″), 0.93 (t, *J* = 7.5 Hz, 3H, H-4‴ ′);

^13^C{^1^H} NMR (125 MHz, CDCl_3_): *δ* 171.7 (C-1), 160.3 (C-5′), 158.7 (C-1‴), 157.65 (C-3′), 157.61 (^B^C-4‴ ″), 156.8 (^A^C-4‴ ″), 144.2 (C-3‴), 144.0 (C-7′), 141.0 (C-2‴ ′), 137.9 (C-1‴ ′), 136.5 (^B^C-1‴ ″), 136.1 (^A^C-1‴ ″), 135.2 (C-7′a), 133.5 (C-8′a), 131.9 (^B^C-2‴ ″), 130.7 (^A^C-2‴ ″), 128.9 (C-5‴), 128.5 (C-1′), 124.0 (C-8′), 122.3 (C-2‴), 120.5 (C-6′), 117.8 (C-2′), 116.0 (C-4‴), 114.2 (^B^C-3‴ ″), 113.5 (^A^C-3‴ ″), 112.5 (C-6‴), 67.8 (C-6″), 66.9 (^B^C-1‴ ‴), 66.6 (^A^C-1‴ ‴), 55.2 (^B^C-2‴ ‴), 55.1 (^A^C-2‴ ‴), 54.9 (^B^C-2‴ ‴ ′), 54.8 (^A^C-2‴ ‴ ′), 39.5 (C-1″), 36.2 (C-2), 29.6 (C-2″), 29.2 (C-5″), 29.1 (C-3‴ ′), 26.7 (C-3″), 25.9 (C-4″), 25.1 (C-3), 23.62 (^B^C-3‴ ‴ ′), 23.57 (^A^C-3‴ ‴ ′), 15.1 (5′-Me), 13.8 (C-4‴ ′), 11.5 (7′-Me);

^19^F{^1^H, ^13^C} NMR (470 MHz, CDCl_3_): *δ* –145.35 (q, ^1^ *J*_19F11B_ = 32.9 Hz, 2F of ^11^BODIPY), −145.28 (sep, ^1^ *J*_19F10B_ = 11.0 Hz, 2F of ^10^BODIPY), Δ^19^F(^11^B^10^B) = 0.07 ppm;

HRMS: *m/z* [M + H]^+^ calcd for C_54_H_69_BF_2_N_5_O_4_: 900.5414; found: 900.5390.

### 2.3 Cytotoxicity of RID-B-BODIPY

For Jurkat cells, 2.0 × 10^4^ cells were seeded in 96-well plates and incubated with the drug for 24 h. For HuCCT1 cells, 4.0 × 10^3^ cells were seeded and allowed to adhere overnight before adding the test compounds, followed by 24 h incubation. Then, 1 h before the end of the drug treatment, 10 µL of 5 mg/mL 3-(4,5-dimethylthiazol-2-yl)-2,5-diphenyl tetrazolium bromide (MTT; Dojindo, Kumamoto, Japan) was added to each well. After an additional 1 h of incubation, in the case of collecting the floating Jurkat cells, the 96-well plates were centrifuged at 300×g for 5 min at room temperature. The supernatants of both the cell cultures were completely removed. MTT formazan was dissolved by adding 100 µL DMSO. The absorbance at 570 nm was measured using a microplate reader (Awareness Technology, Palm City, FL, United States), and cell viability was calculated as the percentage of the absorbance relative to the control (0 µM drug treatment).

### 2.4 Cell imaging of RID-B-BODIPY

HuCCT1 cells (1.5 × 10^5^) were seeded in non-coated glass-bottomed dishes (Matsunami Glass Inc., Osaka, Japan) and allowed to adhere overnight. Subsequently, the cells were treated with 1.0 µM RID-B-BODIPY for 1 h. Thereafter, 30 min prior to the end of the treatment, the membrane-permeable dye Hoechst 33342 (Immunochemistry Technologies, Davis, CA, United States) was added to a final concentration of 400 ng/µL to stain the nuclei. Phenol-BODIPY was used to evaluate the effect of BODIPY alone. After staining, fluorescence within the cells was observed using a confocal laser-scanning microscope FV10i (Olympus, Tokyo, Japan). The captured image data were processed with automatic background subtraction using FV10-ASW software version 04.01.01.05 (Olympus) and then merged using Adobe Photoshop CC 2024 version 25.7.0 (Adobe, San Jose, CA, United States).

### 2.5 Excitation/emission/absorbance spectrum measurements

The synthesized RID-B-BODIPY was dissolved in 100% EtOH to a concentration of 100 µM. Subsequently, 100 µL of this solution was placed into a 96-well flat-bottom microplate (Thermo Fisher Scientific, Waltham, MA, United States). A phenol-BODIPY solution prepared under the same conditions as RID-B-BODIPY was used as the standard sample for BODIPY FL fluorescence. The excitation, emission, and absorbance spectra were measured using a Varioskan LUX multimode microplate reader (Thermo Fisher Scientific). For the excitation spectrum measurement, the detection filter was set to 558 nm, and the sample was exposed to laser light ranging from 300 to 530 nm. For the emission spectrum measurement, the sample was exposed to 450 nm laser light, and the emission was detected using a 468–700 nm filter. The absorbance spectrum was measured across a range of 300–800 nm.

## 3 Results and discussion

### 3.1 Synthesis of RID-B-BODIPY

**SCHEME 1:**
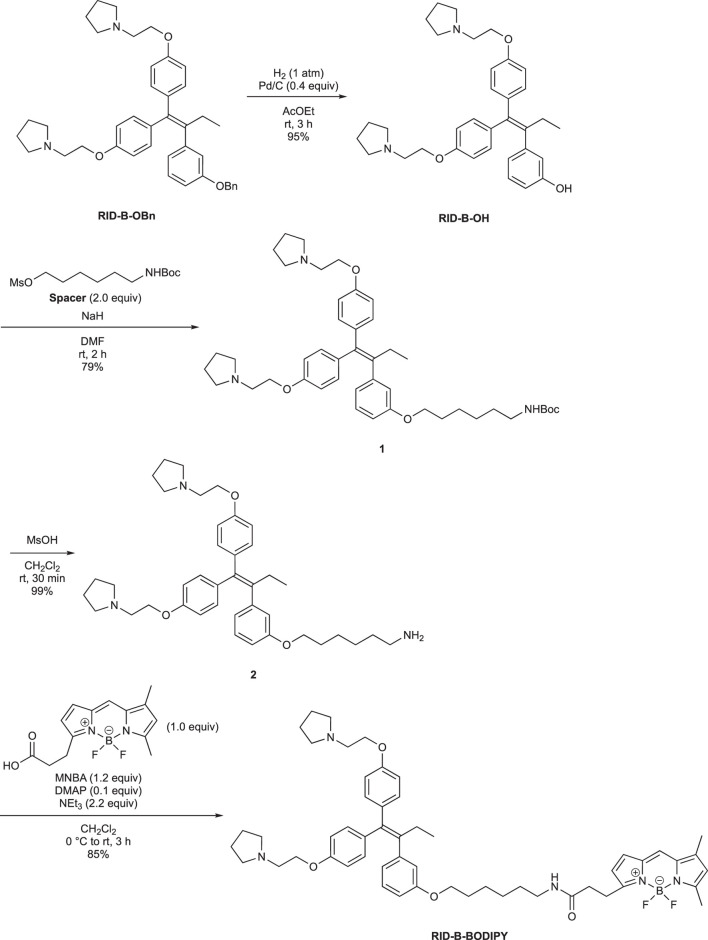
Synthesis of the amino alkyl-tethered RID.

First, we synthesized an amino alkyl chain-tethered **RID-B** (compound **2**), which was the intended coupling partner for BODIPY FL, using the previously reported **RID-B-OBn** (Tsukuda et al., 2013; Ikeda et al., 2015) (Scheme 1). The benzyl (Bn) group in **RID-B-OBn** was removed via hydrogenolysis, yielding **RID-B-OH**, with the double bond in the 1,1,2-triaryl butene skeleton remaining intact. This selective reaction was attributed to the side amino alkyl chains, which effectively poisoned the palladium catalyst, ensuring the exclusive formation of **RID-B-OH**. Subsequently, the hydroxy-free **RID-B-OH** was alkylated with protected amino alkyl mesylate (**Spacer**), which was prepared from the protected amino alcohol according to the literature (Bergbreiter et al., 2002), to produce the ether (compound **1**). The carbon length of the spacer was determined in the previous work, which is biotin-type alkyl chain-tethered RID-B, to avoid the influence on its cytotoxicity. Removal of the Boc group using an excess of mesyl acid (MsOH) provided compound **2**, ready for coupling with BODIPY FL.

Employing the synthesized coupling partners, we initiated the MNBA-mediated dehydration condensation reaction (Table 1). Although carbodiimide-type condensation reagents are typically used for such reactions with bioactive molecules (Malan et al., 2004; Reents et al., 2004), few reports exist on mixed anhydride-mediated coupling (Ling et al., 2019a; Ling et al., 2019b). Consequently, we utilized DCC and MNBA as representative carbodiimide-type and mixed anhydride-mediated coupling reagents, respectively, to compare their efficacy in coupling compound **2** with **BODIPY FL**.

**TABLE 1:** Dehydration condensation reaction between **2** and **BODIPY FL**.

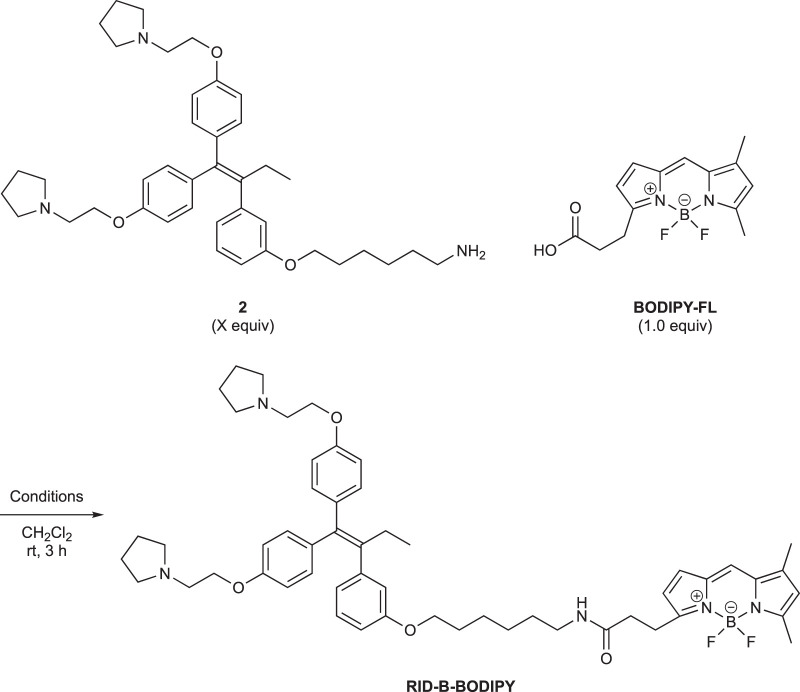			

Entry	X	Conditions	Yield of RID-B-BODIPY (%)
1	1.7	DCC (1.7 equiv), HOBt•H_2_O (1.3 equiv), and NEt_3_ (3.7 equiv)	83
2	1.2	MNBA (1.2 equiv), DMAP (0.1 equiv), and NEt_3_ (2.2 equiv)	85

The DCC-mediated reaction yielded **RID-B-BODIPY** with an 83% yield, requiring 1.7 equivalents of compound **2**. Conversely, the MNBA-mediated reaction also produced **RID-B-BODIPY**, achieving an 85% yield with fewer equivalents (1.2 equivalents) of the substrate amine **2**. Generally, when the condensation reagent could react with the substrate, high substrate-loading conditions are required, such as DCC. However, our mixed anhydride-mediating condensation reaction of BODIPY FL did not require it. These results indicated that using MNBA in the condensation reaction of carboxylic acid is effective for the coupling with not only alcohol but also amine, which is more reactive and more by-productive. Through these comparative experiments, MNBA proved to be an effective condensation reagent for linking BODIPY FL with bioactive molecules.

### 3.2 Comparison of cytotoxic activity against cancer cells between control RID-B and RID-B-BODIPY

**FIGURE 3:**
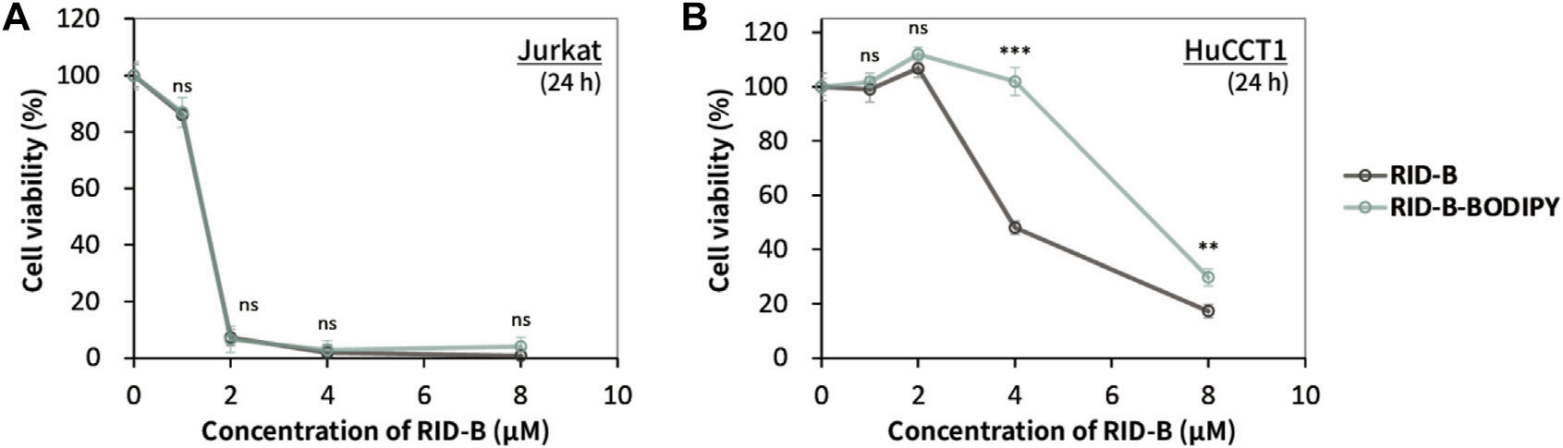
Cytotoxicity of RID-B and RID-B-BODIPY. **(A)** Jurkat or **(B)** HuCCT1 cells were treated with RID-B or RID-B-BODIPY at the indicated concentrations for 24 h.

We assessed the cytotoxicity of RID-B and RID-B-BODIPY using the MTT assay to determine the effect of BODIPY FL coupling on the anticancer activity of RID-B. Jurkat and HuCCT1 cells were treated with either RID-B or RID-B-BODIPY for 24 h. The compounds exhibited comparable cytotoxicity in Jurkat cells (Figure 3A). However, in HuCCT1 cells, RID-B-BODIPY appeared to be less damaging than RID-B, although it still significantly affected the cells at a concentration of 8 µM (Figure 3B). These findings suggest that coupling BODIPY to RID-B retains the cytotoxic efficacy of RID-B.

### 3.3 Localization of RID-B-BODIPY

**FIGURE 4:**
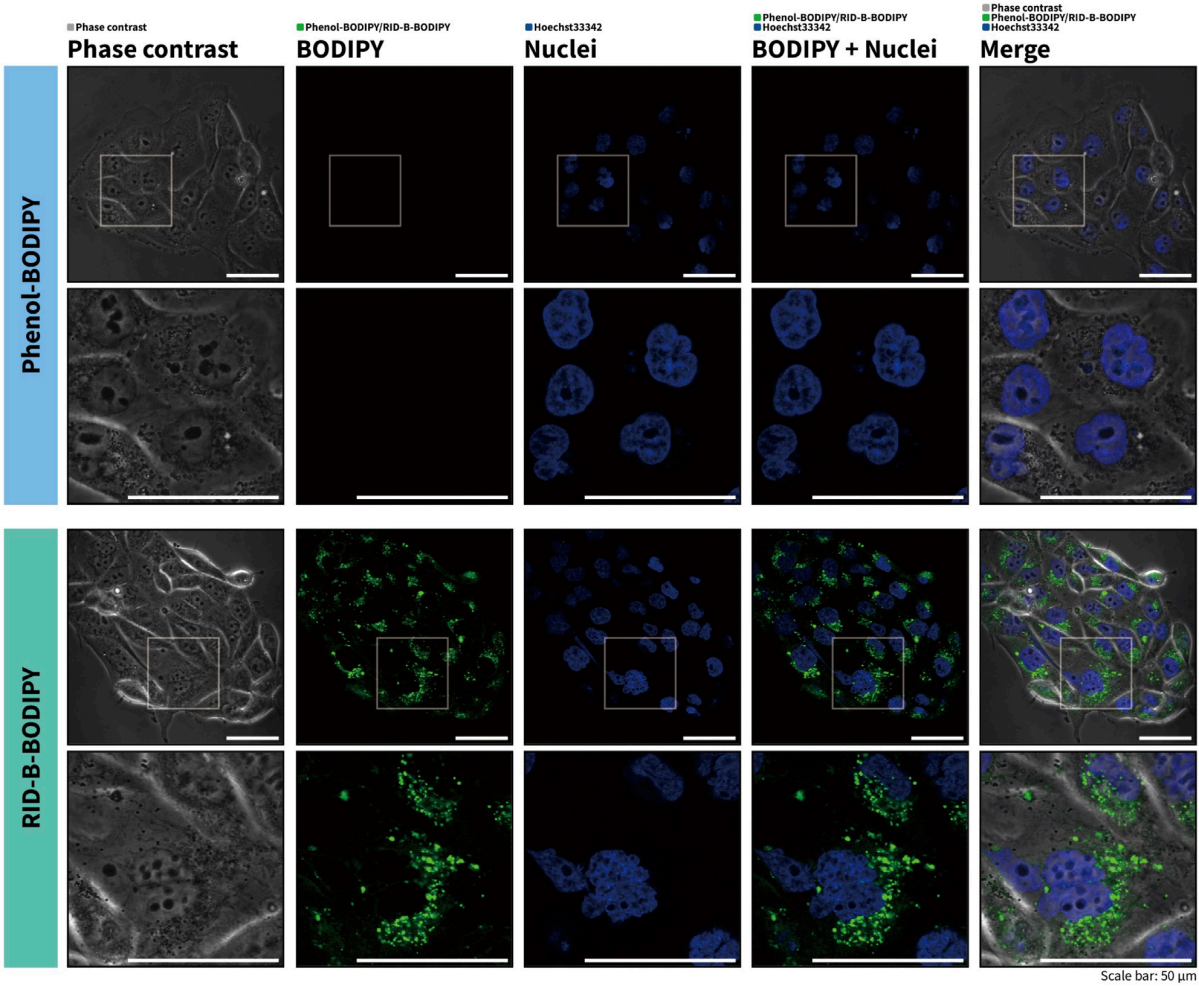
RID-B-BODIPY localizes in the cytoplasm.

To investigate the potential of RID-B-BODIPY to visualize the intracellular localization of RID-B, we treated HuCCT1 cells with RID-B-BODIPY and Hoechst 33342 and subsequently observed the fluorescence by confocal microscopy. Phenol-BODIPY served as a negative control to verify the structural specificity of RID-B localization under identical conditions. Following treatment with 1.0 µM RID-B-BODIPY, circular puncta were observed in the cytoplasm, indicating accumulation in intracellular organelles (Figure 4). Conversely, no fluorescence from BODIPY FL was detected in cells treated with phenol-BODIPY, demonstrating that the intracellular localization of RID-B-BODIPY is contingent upon the RID-B structure.

### 3.4 Photophysical property of RID-B-BODIPY

**FIGURE 5:**
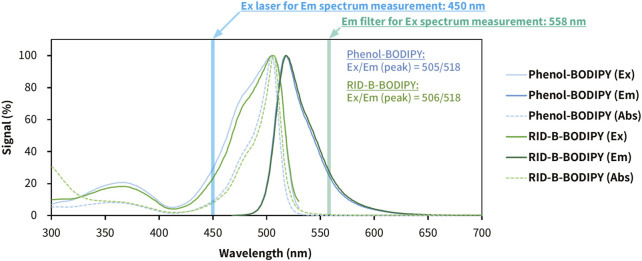
Photophysical properties of phenol-BODIPY and RID-B-BODIPY in ethanol. The excitation, emission, and absorption spectra were measured using a multimode microplate reader. Phenol-BODIPY was used as the standard sample for BODIPY FL fluorescence. Each spectral datum is presented as a relative value, with the signal at the maximum wavelength as 100%. Ex, excitation; Em, emission; Abs, absorbance.

To know the photophysical properties of RID-B-BODIPY, we measured the UV-vis fluorescence spectrum using a multimode microplate reader (Figure 5). The max emission wavelength and max fluorescent wavelength of phenol-BODIPY were 505 nm and 518 nm, respectively. On the other hand, the max emission wavelength and max fluorescent wavelength of RID-B-BODIPY were 506 nm and 518 nm, respectively. By confocal microscopy, the imaging parameter for the wavelength of absorbance of RID-B-BODIPY was 494 nm and that of emission of RID-B-BODIPY was 516 nm. The spectrum showed that these parameters of wavelength were enough for the localization study. The calculated Stokes shifts of phenol-BODIPY and RID-B-BODIPY were 497 cm^–1^ and 458 cm^–1^, respectively. Their Stokes shifts were longer than BODIPY FL in methanol (354 cm^–1^) (Umezawa et al., 2014). Nonetheless, RID-B-BODIPY was demonstrated to be useful for the localization study, given its photophysical properties.

## 4 Conclusion

We synthesized **RID-B-BODIPY** via an MNBA-mediated dehydration condensation reaction between compound **2** and BODIPY FL. Comparative experiments between DCC and MNBA indicated that MNBA is a superior condensation reagent for the coupling of amines with BODIPY FL. These results suggest that the synthesis of bioactive molecule conjugates with BODIPY FL is more efficient, not only in terms of yield but also in purification, as it does not generate urea byproducts. Application of **RID-B-BODIPY** to cancer cells revealed cytotoxicity comparable to that of **RID-B**, indicating that the BODIPY FL moiety does not detract from the inherent properties of **RID-B**. Cell staining experiments with **RID-B-BODIPY** confirmed the intracellular localization of BODIPY FL fluorescence, attributed to the RID-B structure. These findings underscore the successful development of a tool for efficient analysis of intracellular molecular behavior of **RID-B**.

## Data Availability

The original contributions presented in the study are included in the article/Supplementary Material; further inquiries can be directed to the corresponding authors.
