# Supplementary material for: Synthesis of BODIPY FL-tethered ridaifen-B, RID-B-BODIPY, and its localization in cancer cells
Source: Front Chem. 2024 Aug 23;12:1451468. doi: 10.3389/fchem.2024.1451468 (PMC11377228; doi:10.3389/fchem.2024.1451468)
Supplement: Supplementary file 1 [file DataSheet2.PDF]

Table S1. The List of the IUPAC Names of the Compounds and Their Corresponding Structures with Carbon Numbers

Compound	IUPAC	Structure
**S1**	3’-(Benzyloxy)benzaldehyde	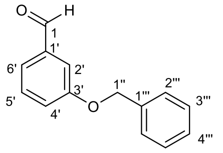
**S2**	1-(3’-(Benzyloxy)phenyl)propan-1-ol	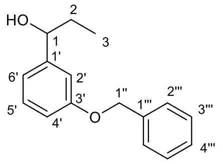
**S3**	1-(3’-(Benzyloxy)phenyl)propan-1-one	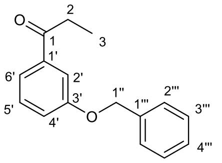
**RID-OBn**	4,4’-(2’’-(3’’’-(Benzyloxy)phenyl) but-1’’-ene-1’’,1’’-diyl)diphenol	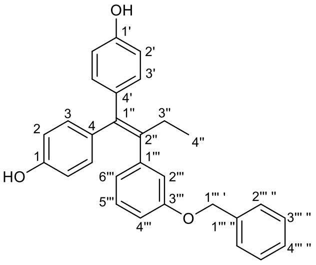
**RID-B-OBn**	1,1’-((((2’’’ ’-(3’’’ ’’-(Benzyloxy)phenyl) but-1’’’ ’-ene-1’’’ ’,1’’’ ’-diyl) bis(4’’’,1’’’- phenylene))bis(oxy)) bis(ethane-2’’,1’’-diyl))dipyrrolidine	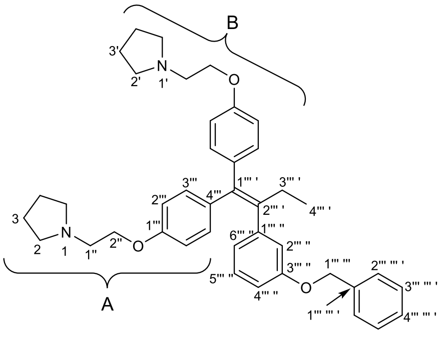
**(*E*)-S4**	Ethyl (*E*)-3-(1’*H*-pyrrol-2’-yl)prop-2-enoate	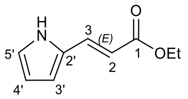
**(*Z*)-S4**	Ethyl (*Z*)-3-(1’*H*-pyrrol-2’-yl)prop-2-enoate	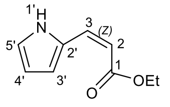
**S5**	Ethyl 3-(1’*H*-pyrrol-2’-yl)propanoate	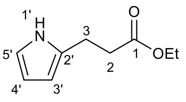
**S7**	2,2,2-Trichloroethyl 3’-(1’’*H*-pyrrol-2’’-yl)propanoate	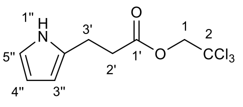

**S8**	2,2,2-Trichloroethyl 3’-(4’’,4’’-difluoro-5’’,7’’-dimethyl-4’’-bora-3’’a,4’’a-diaza-*s* -indacene-3’’-yl)propanoate	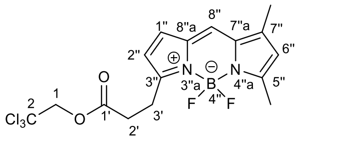
**BODIPY FL**	3-(4’,4’-Difluoro-5’,7’-dimethyl-4’-bora-3’a,4’a-diaza-*s* -indacene-3’-yl)propanoic acid	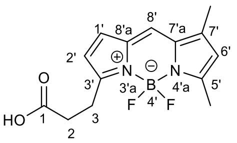
**RID-B-OH**	3-(1’,1’-Bis(4’’-(2’’’-(pyrrolidin-1’’’ ’-yl)ethoxy)phenyl) but-1’-en-2’-yl)phenol	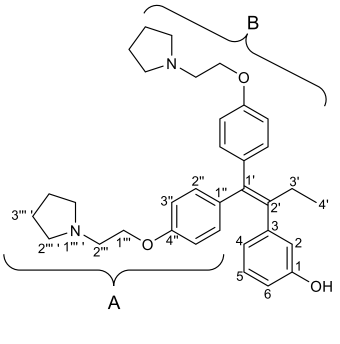
**Spacer**	6-((*tert* -Butoxycarbonyl)amino)hexyl methanesulfonate	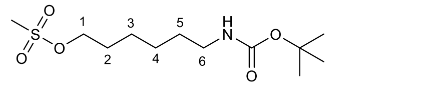
**1**	*tert* -Butyl (6-(3’-(1’’,1’’-bis(4’’’-(2’’’ ’-(pyrrolidin-1’’’ ’’-yl)<br>ethoxy)phenyl)but-1’’-en-2’’- yl)phenoxy)hexyl) carbamate	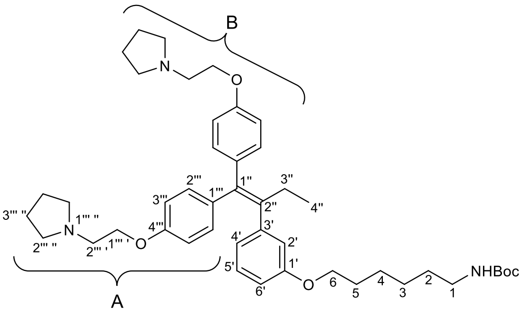
**2**	6-(3’-(1’’,1’’-Bis(4’’’-(2’’’ ’-(pyrrolidin-1’’’ ’’-yl) ethoxy)phenyl)but-1’’-en-2’’- yl)phenoxy)hexan-1-amine	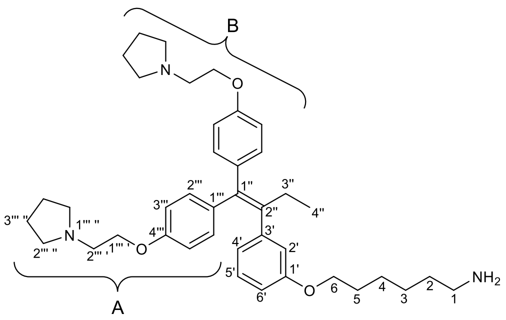
**RID-B-BODIPY**	*N* -(6’’-(3’’’-(1’’’ ’,1’’’ ’-Bis(4’’’ ’’-(2’’’ ’’’-(pyrrolidin-1’’’ ’’’ ’-yl) ethoxy)phenyl)but-1’’’ ’-en-2’’’ ’- yl)phenoxy)hexyl) -3-[4’,4’-difluoro-5’,7’-dimethyl-4’-bora-3’a,4’a-diaza -*s* -indacene-3’- yl]propanamide	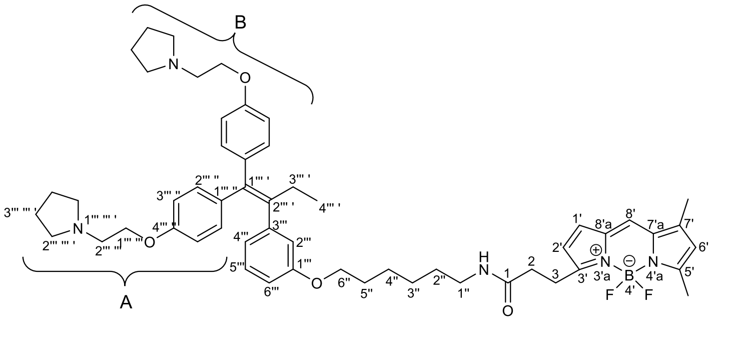
**Phenol-BODIPY**	Phenyl 3-(4’,4’-difluoro-5’,7’-dimethyl-4’-bora-3’a,4’a-diaza-*s* -indacene-3’-yl)propionate	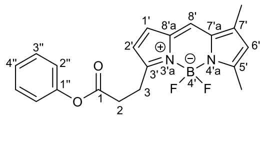
