# Supplementary material for: Synthesis of BODIPY FL-tethered ridaifen-B, RID-B-BODIPY, and its localization in cancer cells
Source: Front Chem. 2024 Aug 23;12:1451468. doi: 10.3389/fchem.2024.1451468 (PMC11377228; doi:10.3389/fchem.2024.1451468)
Supplement: Supplementary file 2 [file DataSheet3.PDF]

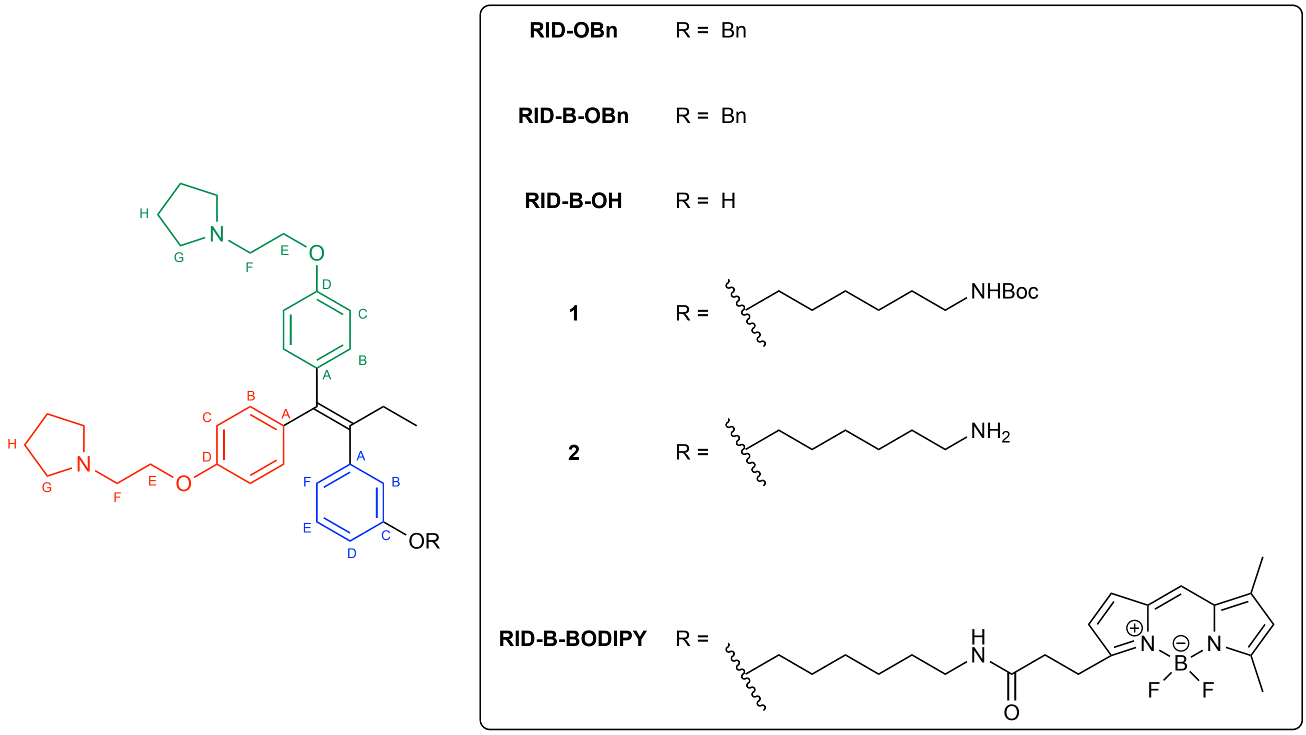

Table S3. The List of Chemical Shifts of RID Skeleton in ^{13}C NMR Spectrum of Each Compound

Numbering of Carbon	**RID-OBn**	**RID-B-OBn**[a)]	**RID-B-OH**[b)]	**1**[c)]	**2**[b)]	**RID-B-BODIPY**[c)]
A	140.9	140.9	136.6	136.4	136.4	136.5
B	132.6	131.9	131.9	132.0	131.9	131.9
C	115.7	114.2	114.1	114.2	114.2	114.2
D	157.1	157.7	156.8	157.7	157.7	157.61
E	-	67.1	66.8	67.1	67.1	66.9
F	-	54.9	55.2	55.30	55.3	55.2
G	-	55.3	54.8	54.9	54.9	54.9
H	-	23.64	23.5	23.63	23.62	23.62
A	139.6	138.1	136.0	136.1	136.1	136.1
B	131.3	130.7	130.7	130.7	130.7	130.7
C	115.1	113.5	113.7	113.5	113.5	113.5
D	156.3	157.0	156.7	156.9	156.9	156.8
E	-	66.9	66.1	66.9	66.9	66.6
F	-	54.8	55.0	55.25	55.2	55.1
G	-	55.2	54.6	54.8	54.8	54.8
H	-	23.59	23.4	23.59	23.57	23.57
A	145.2	144.3	144.3	144.1	144.1	144.2
B	117.3	122.8	121.2	122.3	122.3	122.3
C	159.5	158.6	157.5	158.7	158.8	158.7
D	113.5	112.9	113.4	112.6	112.6	112.5
E	129.6	128.9	129.1	128.8	128.8	128.9
F	123.2	116.5	117.1	116.0	115.9	116.0

a) Determined by COSY, HMQC and HMBC NMR spectra.
b) Determined by HMQC NMR spectra.
c) Determined by HMQC and HMBC NMR spectra.

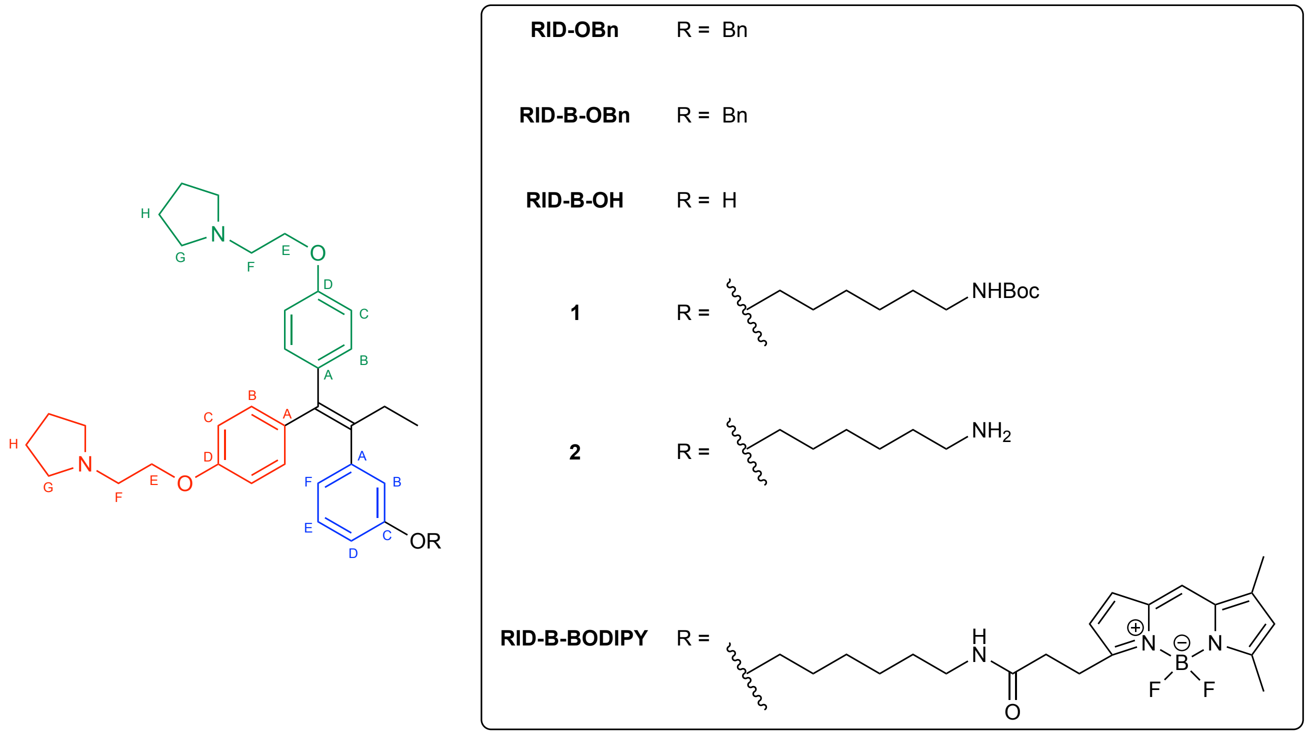

Table S2. The The List of Chemical Shifts of RID Skeleton in ^{1}H NMR Spectrum of Each Compound

Numbering of Carbon	RID-OBn	RID-B-OBn[a)]	RID-B-OH[b)]	1[c)]	2[b)]	RID-B-BODIPY[c)]
A	-	-	-	-	-	-
B	7.13–7.03	7.13	7.10	7.17–7.05	7.18–7.04	7.20–7.03
C	6.78–6.68	6.88	6.86	6.94–6.84	6.94–6.82	6.97–6.77
D	-	-	-	-	-	-
E	-	4.12	4.13	4.12	4.12	4.14
F	-	2.92	2.93	2.91	2.91	2.94
G	-	2.72–2.54	2.72–2.59	2.68–2.56	2.74–2.48	2.79–2.50
H	-	1.91–1.61	1.88–1.75	1.86–1.76	1.90–1.65	1.95–1.65
A	-	-	-	-	-	-
B	6.88–6.78	6.81–6.67	6.76	6.81–6.72	6.83–6.71	6.85–6.70
C	6.55–6.48	6.57	6.60–6.43	6.62–6.50	6.63–6.49	6.56–6.53
D	-	-	-	-	-	-
E	-	3.97	3.94	3.96	3.97	3.96
F	-	2.82	2.82	2.81	2.82	2.83
G	-	2.65–2.51	2.64–2.55	2.63–2.51	2.74–2.48	2.79–2.50
H	-	1.91–1.61	1.83–1.72	1.81–1.72	1.90–1.65	1.95–1.65
A	-	-	-	-	-	-
B	6.88–6.78	6.81–6.67	6.64	6.68	6.67	6.69
C	-	-	-	-	-	-
D	6.78–6.68	6.81–6.67	6.60–6.43	6.68–6.57	6.68–6.58	6.70–6.54
E	7.13–7.03	7.07	7.06–6.92	7.10–7.01	7.10–6.99	7.15–6.93
F	6.78–6.68	6.81–6.67	6.60–6.43	6.68–6.57	6.68–6.58	6.70–6.54

a) Determined by COSY, HMQC and HMBC NMR spectra.
b) Determined by HMQC NMR spectra.
c) Determined by HMQC and HMBC NMR spectra.
