# Supplementary material for: Synthesis of BODIPY FL-tethered ridaifen-B, RID-B-BODIPY, and its localization in cancer cells
Source: Front Chem. 2024 Aug 23;12:1451468. doi: 10.3389/fchem.2024.1451468 (PMC11377228; doi:10.3389/fchem.2024.1451468)
Supplement: Supplementary file 3 [file DataSheet1.pdf]

Supplementary Material

Synthesis of the BODIPY-Tethered Ridaifen-B, RID-B-BODIPY and Its Localization in Cancer Cells.

Takatsugu Murata[1*], Kyoka Komukai[1], Yuta Semba[2], Eri Murata[1], Fumi Sato[1], Tomohiro Takano[1], Kaho Tsuchiya[1], Chihiro Matsuda[1], Anju Sakai[1], Amane Yoneoka[1], Shunsuke Takahashi[2], Yukitoshi Nagahara[2*], Isamu Shiina[1*]

[1]Department of Applied Chemistry, Faculty of Science, Tokyo University of Science, 1-3 Kagurazaka, Shinjuku-ku, Tokyo 162-8601, Japan

[2]Division of Life Science and Engineering, College of Science and Engineering, Tokyo Denki University, Hatoyama, Hiki-gun, Saitama, Japan.

*** Correspondence:**
Takatsugu Murata
t_murata@rs.tus.ac.jp

Yukitoshi Nagahara
yuki@mail.dendai.ac.jp

Isamu Shiina
shiina@rs.kagu.tus.ac.jp

1 Supplementary Data

General Information. Melting points are recorded on a Yanaco MP-S3. ^{1}H, ^{13}C, ^{19}F and ^{11}B NMR spectra were recorded on a JEOL JNM-ECA500II and with chloroform (in chloroform-*d*), or with dimethyl sulfoxide (in dimethyl sulfoxide-d_6) with acetone (in acetone-d_6) as internal standards. ^{19}F NMR was recorded with trifluoromethylbenzene as an internal standard. ^{11}B NMR was recorded with trimethyl borate as an internal standard (18.2 ppm). Structural assignments were made with additional information from gCOSY, gHMQC and gHMBC experiments. Infrared spectra (FT-IR) were recorded on a Horiba FT-300 (FT-IR) or Jasco FT/IR-4600 (ATR-IR). Absorbance frequencies are recorded in reciprocal centimeters (cm^{-1}). High resolution mass spectra (HRMS) were obtained from a Bruker Daltonics micro TOF focus. Ionization was achieved by ESI, modes of ionization, calculated, and found mass are given.
Flash column chromatography was performed on CHROMATOREX® PSQ 60B (60 μm) or Silica gel 60 (35–70 μm). CHROMATOREX® PSQ 60B was purchased from Fuji Silysia Chemical Ltd. and used as received. Silica gel 60 was purchased from Merck KGaA and used as received. Open column chromatography was performed on Silica gel 60 (63–200 μm). Silica gel 60 was purchased from Merck KGaA and used as received.
Thin layer chromatography was performed on Wakogel B5F, using UV light as the visualizing agent and modified phosphomolybdic acid and heat as a developing agent. Thin layer chromatography was purchased from FUJIFILM Wako Pure Chemical Corp.

Reagents.

Stains

Modified phosphomolybdic acid: *ortho*-phosphoric acid (6 mL) was added to a solution of concentrated sulfuric acid (20 mL) in distilled water (400 mL) and then phosphomolybdic acid (9.68 g) was added to the mixture at room temperature. Modified phosphomolybdic acid was stored at room temperature.

ortho-Phosphoric acid was purchased from Sigma-Aldrich Co., LLC and used as received.
Concentrated sulfuric acid was purchased from Kokusan Chemical Co., Ltd. and used as received.
Distilled water was purchased from Kokusan Chemical Co., Ltd. and used as received.
Phosphomolybdic acid was purchased from FUJIFILM Wako Pure Chemical Corp. and used as received.

Solvents and drying reagents.

N,*N*-Dimethylformamide was purchased from Kanto Chemical Co., Inc. and dried over phosphorus pentoxide, then distilled from calcium hydride and dried over Molecular Sieves 4A.
Diethyl Ether, Super Dehydrated was purchased from FUJIFILM Wako Pure Chemical Corp. and used as received.
Tetrahydrofuran (Tetrahydrofuran, Super Dehydrated, Stabilizer free) was purchased from Kanto Chemical Co., Inc. and used as received in the syntheses of **S2** and **RID-OBn**.
Tetrahydrofuran was purchased from Kanto Chemical Co., Inc. and dried over Molecular Sieves 5A in the syntheses of **S6** and **BODIPY FL**.
Dimethyl sulfoxide was purchased from FUJIFILM Wako Pure Chemical Corp. and distilled from calcium hydride under negative pressure with rotary pump and dried over Molecular Sieves 4A.
Dichloromethane was purchased from Kokusan Chemical Co., Ltd. and distilled from phosphorus pentoxide, then calcium hydride and dried over Molecular Sieves 4A in the syntheses of **1** and **RID-B-BODIPY**.
Dichloromethane was purchased from Kokusan Chemical Co., Ltd. and dried over Molecular Sieves 4A for reaction in the syntheses of **S3**, **S4** and **S8**.
Dichloromethane was purchased from Shin-Etsu Chemical Co., Ltd. and used as received for purification.
Ethyl acetate (for HPLC) was purchased from Kokusan Chemical Co., Ltd. and used as received in the synthesis of **RID-B-OH**.
Ethyl acetate was purchased from Kokusan Chemical Co., Ltd. and dried over Molecular Sieves 4A in the synthesis of **S7**.
Ethyl acetate was purchased from Kokusan Chemical Co., Ltd. And used as received for purification.
Ethanol was purchased from Kokusan Chemical Co. and distilled from sodium and dried over Molecular Sieves 3A.
Methanol was purchased from FUJIFILM Wako Pure Chemical Corp. and dried over Molecular Sieves 3A for reaction solvent.
Methanol was purchased from Kanto Chemical Co., Inc. and used as received for purification.
Hexane was purchased from Kokusan Chemical Co., Ltd. and used as received for purification.
Chloroform was purchased from Shin-Etsu Chemical Co., Ltd. and used as received for purification.
Phosphorus pentoxide was purchased from Kokusan Chemical Co., Ltd. and used as received.
Molecular Sieves 3A, Molecular Sieves 4A and Molecular Sieves 5A were purchased from Kokusan Chemical Co., Ltd. and dried by heating under negative pressure with rotary pump before use.
Calcium hydride was purchased from Junsei Chemical Co., Ltd. and used as received.
Sodium, lump, in kerosene was purchased from Kanto Chemical Co., Inc. and used after cutting into small pieces.
Potassium hydroxide was purchased from Kokusan Chemical Co., Ltd. and used as received.

Internal standard.
Trifluoromethylbenzene was purchased from Tokyo Kasei Kogyo Co., Ltd., distilled and dried over Molecular Sieves 4A.
Trimethyl borate was purchased from Tokyo Kasei Kogyo Co., Ltd., distilled and dried over Molecular Sieves 4A.

Reagents for reactions.
3-Hydroxybenzaldehyde was purchased from Tokyo Kasei Kogyo Co., Ltd. and used as received.
Benzyl bromide was purchased from Tokyo Kasei Kogyo Co., Ltd used as received.
Potassium Carbonate was purchased from Kokusan Chemical Co., Ltd used as received.
Ethyl magnesium Bromide wad purchased was from FUJIFILM Wako Pure Chemical Corp used as received.
Ethyl bromide was purchased from Tokyo Kasei Kogyo Co., Ltd. and used as received.
Magnesium, turnings was purchased from NACARAI TESQUE, INC. and used as received.
Iodine was purchased from Kanto Chemical Co., Inc and used as received.
Lithium chloride was purchased from Tokyo Kasei Kogyo Co., Ltd. and used as received.
Triethylamine was purchased from Kokusan Chemical Co., Ltd. and distilled over potassium hydroxide.
Oxalyl chloride was purchased from Tokyo Kasei Kogyo Co., Ltd. and used as received.
Bis(4-hydroxyphenyl)methanone was purchased from Tokyo Kasei Kogyo Co., Ltd and used as received.
Titanium (IV) chloride was purchased from Tokyo Kasei Kogyo Co., Ltd and used as received.
Zinc powder was purchased from Kanto Chemical Co., Inc and used as received.
Sodium hydride (55% dispersion in mineral oil) was purchased from Kanto Chemical Co., Inc. and used as received.
1-(2'-Chloroethyl)pyrrolidine hydrochloride was purchased from Sigma-Aldrich Co., LLC and used as received.
Ethyl 2-(triphenyl-λ^5-phosphaneylidene)acetate was purchased from Tokyo Kasei Kogyo Co., Ltd. and used as received.
Palladium 10% on carbon (M) dry was purchased from Kawaken Fine Chemicals Co., Ltd. and used as received.
Lithium hydroxide was purchased from Merck KGaA and used as received.
2,2,2-Trichloroethanol was purchased from Sigma-Aldrich Co., LLC and used as received.
Pyridine was purchased from Kanto Chemical Co., Inc. and distilled from potassium hydroxide and dried over potassium hydroxide.
Dicyclohexylmethanediimine was purchased from Kokusan Chemical Co., Ltd and used as received.
Phosphoryl chloride was purchased from Tokyo Kasei Kogyo Co., Ltd. and used as received.
3,5-Dimethyl-1*H*-pyrrole-2-carbaldehyde was purchased from Tokyo Kasei Kogyo Co., Ltd. and used as received.
Ethoxyethane; trifluoroborane was purchased from Sigma-Aldrich Co., LLC and used as received.
N-Ethyl-*N*-(1-methylethyl)propan-2-amine was purchased from Tokyo Kasei Kogyo Co., Ltd. and used as received.
tert-Butyl (6-hydroxyhexyl)carbamate was purchased from Tokyo Kasei Kogyo Co., Ltd. and used as received.
Methanesulfonyl chloride was purchased from Tokyo Kasei Kogyo Co., Ltd and used as received.
Methanesulfonic acid was purchased from Tokyo Kasei Kogyo Co., Ltd. and used as received.
2-Methyl-6-nitrobenzoic anhydride (MNBA) was purchased from Tokyo Kasei Kogyo Co., Ltd. and used as received.
4-Dimethylaminopyridine (DMAP) was purchased from Tokyo Kasei Kogyo Co., Ltd. and used as received.
28–30% Aqueous ammonia was purchased from Kanto Chemical Co., Inc. and used as received.

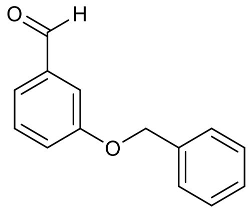

3’-(Benzyloxy)benzaldehyde (S1).

To a solution of 3-hydroxybenzaldehyde (4.88 g, 40.0 mmol) in *N*,*N*-dimethylformamide (80 mL), potassium carbonate (11.0 g, 80.0 mmol) was added. After benzyl bromide (5.70 mL, 48.0 mmol) was added to the mixture at 0 °C, the reaction mixture was stirred for 1 h at room temperature. The reaction was quenched by a saturated aqueous sodium hydrogen carbonate at 0 °C. Two layers were separated, and the aqueous layer was extracted with diethyl eater. The combined organic layers were washed with water and brine successively and dried over sodium sulfate. After filtration of the mixture and concentration of the solvent, the residue was purified by silica gel column chromatography (eluant; hexane/ethyl acetate = 9/1) to give aldehyde **S1** (7.30 g, 86%) as a white solid.

R_f: 0.60 (silica gel, hexane/ethyl acetate = 3/1, UV active; stains blue with modified phosphomolybdic acid);

mp: 55.0 °C (lit.[1] mp: 57–58 °C);

ATR-IR ν_{max}: 2805, 2722, 2324, 1693, 1591, 1440, 1254, 794, 740 cm^{-1};

^{1}H NMR (500 MHz, $CDCl_3$): δ 9.98 (s, 1H, H-1), 7.51–7.43 (m, 5H, H-2’, H-5’, H-6’, H-2’’’), 7.45–7.37 (m, 2H, H-3’’’), 7.38-7.31 (m, 1H, H-4’’’), 7.29–7.22 (m, 1H, H-4’), 5.13 (s, 2H, H-1’’);

$^{13}C\{^1H\}$ NMR (125 MHz, $CDCl_3$): δ 192.2 (C-1), 159.5 (C-3’), 138.0 (C-1’), 136.5 (C-1’’’), 130.3 (C-5’), 128.8 (C-4’’’), 128.4 (C-3’’’), 127.7 (C-2’’’), 123.8 (C-6’), 122.4 (C-4’), 113.4 (C-2’), 70.4 (C-1’’);

HRMS: *m/z* [M + H]$^+$ calcd for $C_{14}H_{13}O_2$: 213.0910; found: 213.0904.

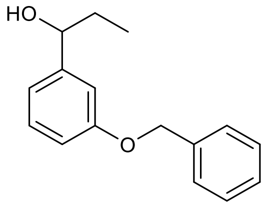

1-(3’-(Benzyloxy)phenyl)propan-1-ol (S2).

To activated magnesium (3.26 g, 134 mmol) and Iodine (1 leaf), ethyl bromide (9.11 mL, 122 mmol) in tetrahydrofuran (82 mL) was added with gently refluxing by heat of the reaction. After stirring 1 h, the reaction solution was diluted by tetrahydrofuran (40 mL). The prepared Grignard reagent was titrated according to the Knochel’s method[2)] using iodine and 0.5 M lithium chloride in tetrahydrofuran and calculated as 0.59 M.

To a solution of aldehyde **S1** (7.15 g, 33.7 mmol) in tetrahydrofuran (67 mL), 0.59 M solution of ethyl magnesium bromide in tetrahydrofuran (68.1 mL, 40.4 mmol) was added at 0 °C. The reaction mixture was stirred for 1 h at room temperature. The reaction was quenched by a saturated aqueous ammonium chloride at 0 °C. Two layers were separated, and the aqueous layer was extracted with diethyl eater. The combined organic layers were washed with water and brine successively and dried over sodium sulfate. After filtration of the mixture and concentration of the solvent, the residue was purified by silica gel column chromatography (eluant; hexane/ethyl acetate = 6/1) to give alcohol **S2** (6.76 g, 83%) as a yellow oil.

R_f: 0.30 (silica gel, hexane/ethyl acetate = 3/1, UV active; stains blue with modified phosphomolybdic acid);

FT-IR (neat) ν_{max}: 3385, 3033, 2964, 2931, 2874, 1263, 1157, 784, 740 cm^{-1};
^{1}H NMR (500 MHz, $CDCl_3$): δ 7.43 (d, J = 7.5 Hz, 2H, H-2’’’), 7.41–7.35 (m, 2H, H-3’’’), 7.35–7.29 (m, 1H, H-4’’’), 7.25 (dd, J = 7.5, 7.5 Hz, 1H, H-5’), 7.03–6.95 (m, 1H, H-2’), 6.93 (d, J = 7.5 Hz, 1H, H-4’), 6.88 (dd, J = 7.5, 2.5 Hz, 1H, H-6’), 5.07 (s, 2H, H-1’’), 4.57 (t, J = 6.5 Hz, 1H, H-1), 1.90–1.64 (m, 2H, H-2), 0.91 (t, J = 7.5 Hz, 3H, H-3);
^{13}C{^{1}H} NMR (125 MHz, $CDCl_3$): δ 159.1 (C-3’), 146.5 (C-1’), 137.1 (C-1’’’), 129.6 (C-5’), 128.7 (C-3’’’), 128.1 (C-4’’’), 127.7 (C-2’’’), 118.7 (C-6’), 113.9 (C-4’), 112.6 (C-2’), 76.0 (C-1), 70.1 (C-1’’), 32.0 (C-2), 10.2 (C-3);
HRMS: *m*/*z* [M + Na]$^+$ calcd for $C_{16}H_{18}O_2Na$: 265.1199; found: 265.1193.

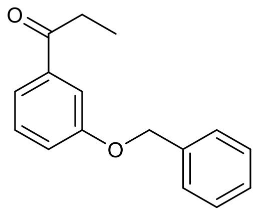

1-(3’-(Benzyloxy)phenyl)propan-1-one (S3).
To a solution of dimethyl sulfoxide (3.66 mL, 55.8 mmol) in dichloromethane (90 mL), oxalyl chloride (3.64 mL, 41.9 mmol) was added at –78 °C, and the mixture was stirred for 15 min. After a solution of alcohol **S2** (6.76 g, 27.9 mmol) in dichloromethane (50 mL) was added to the mixture, the reaction mixture was stirred for 15 min and then triethylamine (11.7 mL, 83.7 mmol) was added to the mixture. After the completion of addition, the reaction mixture was stirred for 35 min at room temperature. The reaction was quenched by water at 0 °C. Two layers were separated, and the aqueous layer was extracted with dichloromethane. The combined organic layers were washed with water and brine successively and dried over sodium sulfate. After filtration of the mixture and concentration of the solvent, the residue was purified by silica gel column chromatography (eluant; hexane/ethyl acetate = 9/1 to 6/1 to 3/1) to give ketone **S3** (6.33 g, 95%) as a pale yellow solid.
R_f: 0.60 (silica gel, hexane/ethyl acetate = 3/1, UV active; stains blue with modified phosphomolybdic acid);
mp: 31.9 °C (lit.[3] mp: 30 °C);
ATR-IR ν_{max}: 3343, 3034, 2981, 2933, 2905, 2873, 2353, 1683, 1087, 782, 741 cm^{-1};
^{1}H NMR (500 MHz, $CDCl_3$): δ 7.62–7.52 (m, 2H, H-5’, H-6’), 7.45 (d, J = 7.0 Hz, 2H, H-2’’’), 7.44–7.30 (m, 4H, H-2’, H-3’’’, H-4’’’), 7.17 (dd, J = 8.5, 2.0 Hz, 1H, H-4’), 5.11 (s, 2H, H-1’’), 2.98 (q, J = 7.5 Hz, 2H, H-2), 1.22 (t, J = 7.5 Hz, 3H, H-3);
^{13}C{^{1}H} NMR (125 MHz, $CDCl_3$): δ 200.7 (C-1), 159.1 (C-3’), 138.5 (C-1’), 136.7 (C-1’’’), 129.7 (C-5’), 128.8 (C-3’’’), 128.3 (C-4’’’), 127.7 (C-2’’’), 121.0 (C-6’), 120.1 (C-4’), 113.6 (C-2’), 70.3 (C-1’’), 32.0 (C-2), 8.4 (C-3);
HRMS: *m*/*z* [M + Na]$^+$ calcd for $C_{16}H_{16}O_2Na$: 263.1043; found: 263.1044.

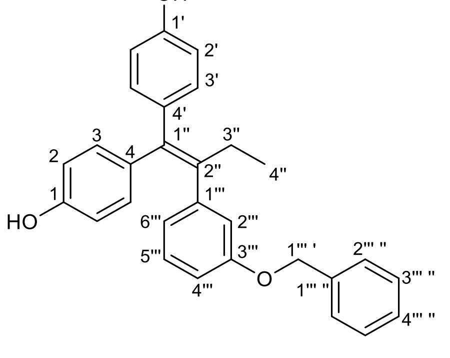

4,4’-(2’’-(3’’’-(Benzyloxy)phenyl)but-1’’-ene-1’’,1’’-diyl)diphenol (RID-OBn).

To a suspension of zinc powder (902.1 mg, 13.8 mmol) in tetrahydrofuran (9.8 mL), titanium (IV) chloride (0.68 mL, 6.20 mmol) was added dropwise at –10 °C. The reaction mixture was refluxed at 90 °C for 2 h and then a solution of bis(4-hydroxyphenyl)methanone (244.3 mg, 1.02 mmol) and **S3** (698.4 mg, 3.26 mmol) in tetrahydrofuran (10.6 mL) were added at 0 °C. After the reaction mixture was refluxed at 90 °C for 2 h in the dark, the reaction was quenched by 10% aqueous potassium carbonate at 0 °C in the light. The mixture was filtered through a short pad of celite with ethyl acetate, and the filtrate was extracted with ethyl acetate. The combined organic layers were washed with brine and dried over sodium sulfate. After filtration of the mixture and concentration of the solvent, the residue was purified by silica gel column chromatography (eluant; hexane/ethyl acetate = 2/1) to give **RID-OBn** (7.09 g, 94%) as a white solid.

R_f: 0.20 (silica gel, hexane/ethyl acetate = 2/1, UV active; stains blue with modified phosphomolybdic acid);

mp: 164.3 °C (lit.[3] mp: 153–156 °C);

ATR-IR ν_{max}: 3266, 2960, 2865, 2360, 1592, 1506, 1222 cm^{-1};

^1H NMR (500 MHz, acetone-d_6): δ 8.32 (s, 1H, 1’-OH), 8.10 (s, 1H, 1-OH), 7.45–7.34 (m, 4H, H-2’’’ ’’, H-3’’’ ’’), 7.34–7.28 (m, 1H, H-4’’’ ’’), 7.13–7.03 (m, 3H, H-3’, H-5’’’), 6.88–6.78 (m, 3H, H-3, H-2’’’), 6.78–6.68 (m, 4H, H-2’, H-4’’’, H-6’’’), 6.55–6.48 (m, 2H, H-2), 4.97 (s, 2H, H-1’’’ ’), 2.47 (q, J = 8.0 Hz, 2H, H-3’’), 0.91 (t, J = 8.0 Hz, 3H, H-4’’);

$^{13}C\{^1H\}$ NMR (125 MHz, acetone-d_6): δ 159.5 (C-3’’’), 157.1 (C-1’), 156.3 (C-1), 145.2 (C-1’’’), 140.9 (C-4’), 139.6 (C-4), 138.5 (C-1’’’ ’’), 136.0 (C-1’’), 135.8 (C-2’’), 132.6 (C-3’), 131.3 (C-3), 129.6 (C-5’’’), 129.2 (C-4’’’ ’’), 128.5 (C-3’’’ ’’), 128.4 (C-2’’’ ’’), 123.2 (C-6’’’), 117.3 (C-2’’’), 115.7 (C-2’), 115.1 (C-2), 113.5 (C-4’’’), 70.4 (C-1’’’ ’), 29.5 (C-3’’), 13.9 (C-4’’);

HRMS: m/z $[M + Na]^+$ calcd for $C_{29}H_{26}O_3Na$: 445.1774; found: 445.1781.

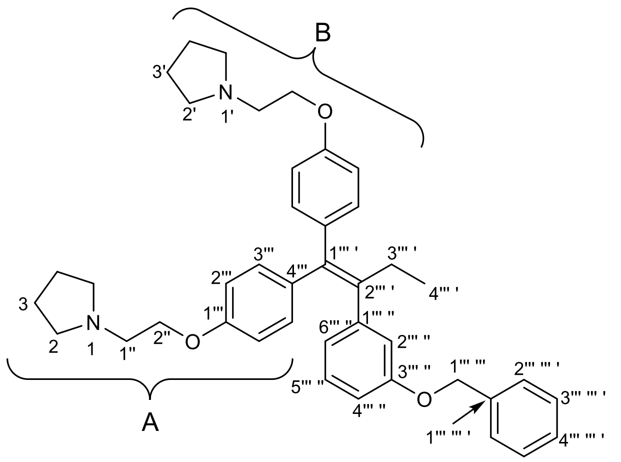

1,1’-((((2’’’ ’-(3’’’ ’’-(Benzyloxy)phenyl)but-1’’’ ’-ene-1’’’ ’,1’’’ ’-diyl)bis(4’’’,1’’’-phenylene))bis(oxy))bis(ethane-2’’,1’’-diyl))dipyrrolidine (RID-B-OBn).

To a solution of **RID-OBn** (136.4 mg, 0.323 mmol) in *N*,*N*-dimethylformamide (3.23 mL), 55% sodium hydride (dispersion in paraffin liquid, 112.7 mg, 2.58 mmol) was added. The reaction mixture was stirred for 15 min at 50 °C and then 1-(2’-chloroethyl)pyrrolidine hydrochloride (182.0 mg, 1.07 mmol) was added to the mixture at room temperature. The reaction mixture was stirred for 3 h at 50 °C. The reaction was quenched by brine at 0 °C. Two layers were separated, and the aqueous layer was extracted with diethyl eater. The combined organic layers ware dried over sodium sulfate. After filtration of the mixture and concentration of the solvent, the residue was purified by preparative thin layer chromatography (eluant; ammoniacal chloroform/methanol = 9/1, R_f: 0.95) to give crude of

RID-B-OBn. The crude was purified by preparative thin layer chromatography (eluant; chloroform/methanol = 9/1, R_f: 0.40) to give **RID-B-OBn** (61.5 mg, 93%) as a white solid.
R_f: 0.40 (silica gel, chloroform/methanol = 9/1, UV active; stains blue with modified phosphomolybdic acid);
mp: 88.7 °C;
ATR-IR ν_{max}: 2961, 1602, 1506, 1240 cm^{-1};
^{1}H NMR (500 MHz, $CDCl_3$): δ 7.43–7.23 (m, 5H, H-2’’’ ’’’ ’, H-3’’’ ’’’ ’, H-4’’’ ’’’ ’), 7.13 (d, J = 8.5 Hz, 2H, ^{B}H-3’’’), 7.07 (t, J = 7.5 Hz, 1H, H-5’’’ ’’), 6.88 (d, J = 8.5 Hz, 2H, ^{B}H-2’’’), 6.81–6.67 (m, 5H, ^{A}H-3’’’, H-2’’’ ’’, H-4 ’’’ ’’, H-6 ’’’ ’’), 6.57 (d, J = 9.0 Hz, 2H, ^{A}H-2’’’), 4.88 (s, 2H, H-1’’’ ’’’), 4.12 (t, J = 6.5 Hz, 2H, ^{B}H-2’’), 3.97 (t, J = 6.0 Hz, 2H, ^{A}H-2’’), 2.92 (t, J = 6.5 Hz, 2H, ^{B}H-1’’), 2.82 (t, J = 6.0 Hz, 2H, ^{A}H-1’’), 2.72–2.54 (m, 4H, ^{B}H-2’), 2.65–2.51 (m, 4H, ^{A}H-2), 2.45 (q, J = 7.5 Hz, 2H, H-3’’’ ’), 1.91–1.61 (m, 8H, ^{B}H-3’, ^{A}H-3), 0.92 (t, J = 7.5 Hz, 3H, H-4’’’ ’);
^{13}C{^{1}H} NMR (125 MHz, $CDCl_3$): δ 158.6 (C-3’’’ ’’), 157.7 (^{B}C-1’’’), 157.0 (^{A}C-1’’’), 144.3 (C-1’’’ ’’), 140.9 (^{B}C-4’’’), 138.1 (^{A}C-4’’’), 137.3 (C-1’’’ ’’’ ’), 136.4 (C-2’’’ ’), 136.0 (C-1’’’ ’), 131.9 (^{B}C-3’’’), 130.7 (^{A}C-3’’’), 128.9 (C-5’’’ ’’), 128.6 (C-4’’’ ’’’ ’), 128.0 (C-5’’’ ’’’ ’), 127.7 (C-6’’’ ’’’ ’), 122.8 (C-2’’’ ’’), 116.5 (C-6’’’ ’’), 114.2 (^{B}C-2’’’), 113.5 (^{A}C-2’’’), 112.9 (C-4’’’ ’’), 70.1 (C-1’’’ ’’’), 67.1 (^{B}C-2’’), 66.9 (^{A}C-2’’), 55.3 (^{B}C-2’), 55.2 (^{A}C-2), 54.9 (^{B}C-1’’), 54.8 (^{A}C-1’’), 29.1 (C-3’’’ ’), 23.64 (^{B}C-3’), 23.59 (^{A}C-3), 13.8 (C-4’’’ ’);
HRMS: m/z [M + H]$^+$ calcd for $C_{41}H_{49}N_2O_3$: 617.3738; found: 617.3762.

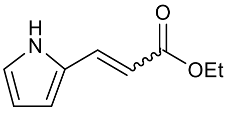

Ethyl (*E*)-3-(1’*H*-pyrrol-2’-yl)prop-2-enoate ((*E*)-S4), Ethyl (*Z*)-3-(1’*H*-pyrrol-2’-yl)prop-2-enoate ((*Z*)-S4).
To a solution of 1*H*-pyrrole-2-carbaldehyde (2.4 g, 25.3 mmol) in dichloromethane (25 mL), ethyl 2-(triphenyl-λ^5 phosphaneylidene)acetate (17.7 g, 50.7 mmol) was added at 0 °C. After the reaction mixture was stirred for 24 h at room temperature, the mixture was concentrated. Diethyl ether was added to the mixture until triphenylphosphine oxide was precipitated. After filtration of the mixture and concentration of the solvent, the residue was purified by silica gel column chromatography (eluant; hexane/ethyl acetate = 5/1 to 3/1) to give **(*E/Z*)-S4** (3.31 g, 79%, *E*/*Z* = 90/10) as a pale brown solid.
mp: 49.8 °C;
ATR-IR ν_{max}: 3245, 2985, 2328, 1675, 1622,1303, 1230, 1193 cm^{-1};
HRMS: m/z [M + Na]$^+$ calcd for $C_9H_{11}NO_2Na$: 188.0682; found: 188.0681.

Analytical sample of **(*E*)-S4** and **(*Z*)-S4** were prepared as a yellow solid and pale yellow oil respectively by purification the crude product using silica gel column chromatography (eluant; hexane/ethyl acetate = 5/1 to 3/1 to 2/1).

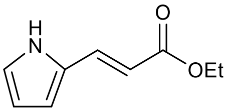

Ethyl (*E*)-3-(1’*H*-pyrrol-2’-yl)prop-2-enoate ((*E*)-S4).
R_f: 0.34 (silica gel, hexane/ethyl acetate = 4/1, UV active; stains blue with modified phosphomolybdic acid);
mp: 56.1 °C (lit.[5] mp: 58–59 °C);
ATR-IR ν_{max}: 3297, 1679, 1614, 1297, 1229, 1179 cm^{-1};

^{1}H NMR (500 MHz, $CDCl_3$): δ 8.74–8.26 (brm, 1H, H-1'), 7.54 (d, J = 16.0 Hz, 1H, H-3), 6.97–6.88 (m, 1H, H-4'), 6.60–6.52 (m, 1H, H-3'), 6.32–6.25 (m, 1H, H-5'), 5.97 (d, J = 16.0 Hz, 1H, H-2), 4.24 (q, J = 7.0 Hz, 2H, OCH_2CH$_3$), 1.32 (t, J = 7.0 Hz, 3H, OCH$_2$$CH_3$);
$^{13}C\{^{1}H\}$ NMR (125 MHz, $CDCl_3$): δ 167.9 (C-1), 134.4 (C-2'), 128.6 (C-3), 122.5 (C-4'), 114.4 (C-3'), 111.4 (C-2), 111.1 (C-5'), 60.4 (OCH$_2$CH$_3$), 14.5 (OCH$_2$$CH_3$);
HRMS: m/z [M + Na]$^+$ calcd for $C_9H_{11}NO_2Na$: 188.0682; found: 188.0683.

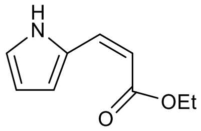

Ethyl (*Z*)-3-(1'*H*-pyrrol-2'-yl)prop-2-enoate ((*Z*)-S4).
R_f: 0.69 (silica gel, hexane/ethyl acetate = 4/1, UV active; stains blue with modified phosphomolybdic acid);
FT-IR (neat) ν_{max}: 3269, 2980, 1686, 1597, 1220, 1192 cm^{-1};
^{1}H NMR (500 MHz, $CDCl_3$): δ 12.62–11.86 (brm, 1H, H-1'), 7.08–6.92 (m, 1H, H-4'), 6.77 (d, J = 12.0 Hz, 1H, H-3), 6.60–6.42 (m, 1H, H-3'), 6.36–6.16 (m, 1H, H-5'), 5.53 (d, J = 12.0 Hz, 1H, H-2), 4.22 (q, J = 7.0 Hz, 2H, OCH_2CH$_3$), 1.32 (t, J = 7.0 Hz, 3H, OCH$_2$$CH_3$);
$^{13}C\{^{1}H\}$ NMR (125 MHz, $CDCl_3$): δ 169.3 (C-1), 134.8 (C-2'), 129.2 (C-3), 123.0 (C-4'), 118.7 (C-3'), 110.2 (C-5'), 107.8 (C-2), 60.5 (OCH$_2$CH$_3$), 14.4 (OCH$_2$$CH_3$);
HRMS: m/z [M + H]$^+$ calcd for $C_9H_{12}NO_2$: 166.0863; found: 166.0855.

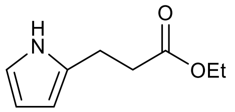

Ethyl 3-(1'*H*-pyrrol-2'-yl)propanoate (S5).
To a solution of **(*E*/*Z*)-S4** (*E*/*Z* = 90/10) (2.0 g, 12.1 mmol) in ethanol (81 mL), palladium 10% on carbon (M) (128.9 mg, 0.12 mmol) was added. The reaction mixture was stirred for 3 h at room temperature under an atmosphere of hydrogen (1.0 atm) and then transferred to an atmosphere of argon. After filtration of the mixture through a short pad of celite with ethanol and concentration of the solvent, the residue was purified by silica gel column chromatography (eluant; hexane/ethyl acetate = 4/1) to give **S5** (1.9 g, 94%) as a yellow oil.
R_f: 0.41 (silica gel, hexane/ethyl acetate = 4/1, UV active; stains blue with modified phosphomolybdic acid);
FT-IR (neat) ν_{max}: 3385, 2980, 1716, 1374, 1195 cm^{-1};
^{1}H NMR (500 MHz, $CDCl_3$): δ 8.90–8.15 (brm, 1H, H-1'), 6.73–6.61 (m, 1H, H-4'), 6.17–6.03 (m, 1H, H-5'), 5.98–5.84 (m, 1H, H-3'), 4.15 (q, J = 7.0 Hz, 2H, OCH_2CH$_3$), 2.91 (t, J = 7.0 Hz, 2H, H-3), 2.63 (t, J = 7.0 Hz, 2H, H-2), 1.26 (t, J = 7.0 Hz, 3H, OCH$_2$$CH_3$);
$^{13}C\{^{1}H\}$ NMR (125 MHz, $CDCl_3$): δ 174.3 (C-1), 131.2 (C-2'), 116.9 (C-4'), 108.1 (C-5'), 105.6 (C-3'), 60.8 (OCH$_2$CH$_3$), 34.7 (C-3), 22.7 (C-2), 14.3 (OCH$_2$$CH_3$);
HRMS: m/z [M + H]$^+$ calcd for $C_9H_{14}NO_2$: 168.1019; found: 168.1020.

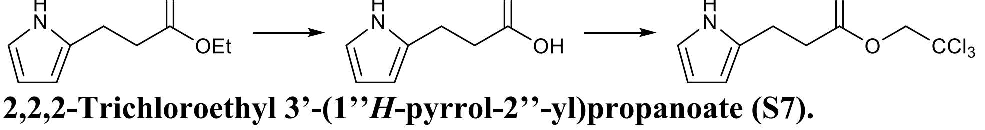

2,2,2-Trichloroethyl 3'-(1''*H*-pyrrol-2''-yl)propanoate (S7).

To a solution of **S5** (2.0 g, 12.0 mmol) in tetrahydrofuran (80 mL) and methanol (40 mL), 4 M aqueous lithium hydroxide (40 mL, 160.0 mmol) was added at 0 °C. After the reaction mixture was stirred for 1.5 h at room temperature, the reaction was quenched by 1 M aqueous hydrochloride at 0 °C. Two layers were separated, and the aqueous layer was extracted with ethyl acetate. The combined organic layers ware dried over sodium sulfate. After filtration of the mixture and concentration of the solvent, the crude 3-(1'*H*-pyrrol-2'-yl)propanoic acid **S6** was obtained, and was used in the next step without further purification.

To a solution of above crude 3-(1'*H*-pyrrol-2'-yl)propanoic acid **S6** in ethyl acetate (50 mL), pyridine (1.25 mL, 15.5 mmol) and 2,2,2,-trichloroethanol (1.37 mL, 14.4 mmol) were added successively. After *N*,*N*'-dicyclohexylmethanediimine (2.7 g, 13.16 mmol) in ethyl acetate (30 mL) was added to the mixture at 0 °C, the reaction mixture was stirred at room temperature for 8 h. The mixture was filtered with ethyl acetate and concentrated. The residue was purified by silica gel column chromatography (eluant; hexane/ethyl acetate = 10/1 to 7/1) to give **S7** (2.92 g, 90%) as a white solid.

R_f: 0.56 (silica gel, hexane/ethyl acetate = 4/1, UV active; stains blue with modified phosphomolybdic acid);

mp: 52.9 °C;

ATR-IR ν_{max}: 3402, 1733, 1166 cm^{-1};

^{1}H NMR (500 MHz, $CDCl_3$): δ 8.60–7.80 (brm, 1H, H-1''), 6.71–6.64 (m, 1H, H-4''), 6.17–6.05 (m, 1H, H-5''), 6.01–5.90 (m, 1H, H-3''), 4.76 (s, 2H, H-1), 2.99 (t, J = 7.0 Hz, 2H, H-3'), 2.81 (t, J = 7.0 Hz, 2H, H-2');

^{13}C{^{1}H} NMR (125 MHz, $CDCl_3$): δ 172.4 (C-1'), 130.3 (C-2''), 117.1 (C-4''), 108.4 (C-5''), 105.8 (C-3''), 94.9 (C-2), 74.2 (C-1), 34.4 (C-2'), 22.6 (C-3');

HRMS: m/z [M + Na]$^+$ calcd for $C_9H_{10}Cl_3NO_2Na$: 291.9669; found: 291.9672.

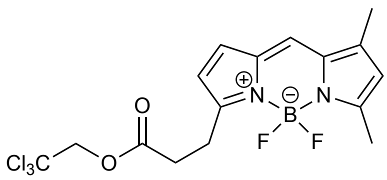

2,2,2-Trichloroethyl 3'-(4'',4''-difluoro-5'',7''-dimethyl-4''-bora-3''a,4''a-diaza-*s*-indacene-3''-yl)propanoate (S8).

To a solution of **S7** (2.83 g, 10.5 mmol) and 3,5-dimethyl-1*H*-pyrrole-2-carbaldehyde (1.55 g, 12.6 mmol) in dichloromethane (11 mL), phosphoryl chloride (1.17 mL, 12.6 mmol) in dichloromethane (10 mL) was added at 0 °C. After the reaction mixture was stirred for 3 h at room temperature, ethoxyethane; trifluoroborane (4.52 mL, 36.7 mmol) and *N*-ethyl-*N*-(1-methylethyl)propan-2-amine (8.21 mL, 47.1 mmol) were successively added at 0 °C. After the reaction mixture was stirred for 3 h at room temperature, the reaction was quenched by a saturated aqueous ammonium chloride at 0 °C. Two layers were separated, and the aqueous layer was extracted with dichloromethane. The combined organic layers ware dried over sodium sulfate. After filtration of the mixture and concentration of the solvent, the residue was purified by silica gel column chromatography (eluant; hexane/ethyl acetate = 10/1 to 7/1) to give **S8** (2.42 g, 54%) as a gray solid.

R_f: 0.34 (silica gel, hexane/ethyl acetate = 4/1, UV active; stains blue with modified phosphomolybdic acid);

mp: 86.0 °C;

FT-IR (KBr) ν_{max}: 3492, 1758, 1607, 1136, 1057 cm^{-1};

^{1}H NMR (500 MHz, $CDCl_3$): δ 7.09 (s, 1H, H-8''), 6.88 (d, J = 4.0 Hz, 1H, H-1''), 6.29 (d, J = 4.0 Hz, 1H, H-2''), 6.12 (s, 1H, H-6''), 4.77 (s, 2H, H-1), 3.35 (t, J = 7.5 Hz, 2H, H-3'), 2.94 (t, J = 7.5 Hz, 2H, H-2'), 2.57 (s, 3H, 5''-Me), 2.26 (s, 3H, 7''-Me);

$^{13}C\{^1H\}$ NMR (125 MHz, $CDCl_3$): δ 171.1 (C-1'), 161.0 (C-5''), 156.2 (C-3''), 144.3 (C-7''), 135.5 (C-7''a) , 133.4 (C-8''a), 128.0 (C-1''), 124.0 (C-8''), 120.7 (d, $^4J_{CF}$ = 2.4 Hz, C-6''), 116.7 (d, $^4J_{CF}$ = 2.4 Hz, C-2''), 95.0 (C-2), 74.2 (C-1), 33.2 (C-2'), 23.8 (C-3'), 15.1 (5''-Me), 11.4 (7''-Me);
$^{19}F\{^1H, ^{13}C\}$ NMR (470 MHz, $CDCl_3$): δ –146.31 (q, $^1J_{19F11B}$ = 32.9 Hz, 2F of ^{11}BODIPY), –146.25 (sep, $^1J_{19F10B}$ = 11.0 Hz, 2F of ^{10}BODIPY), $\Delta^{19}F(^{11}B^{10}B)$ = 0.06 ppm;
HRMS: *m*/*z* $[M + Na]^+$ calcd for $C_{16}H_{16}BCl_3F_2N_2O_2Na$: 445.0234; found: 445.0216.

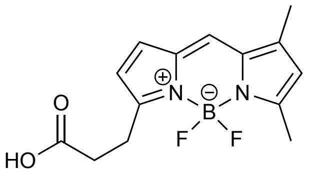

3-(4',4'-Difluoro-5',7'-dimethyl-4'-bora-3'a,4'a-diaza-*s*-indacene-3'-yl)propanoic acid (BODIPY FL).

To a solution of **S8** (2.62 g, 6.2 mmol) in tetrahydrofuran (310 mL), 0.2 M aqueous lithium hydroxide (93 mL, 18.6 mmol) was added at 0 °C. After the reaction mixture was stirred for 2 h at 0 °C, the reaction was quenched by 1 M aqueous hydrochloride at 0 °C. Two layers were separated, and the aqueous layer was extracted with ethyl acetate. The combined organic layers were dried over sodium sulfate. After filtration of the mixture and concentration of the solvent, the residue was purified by silica gel column chromatography (eluant; dichloromethane/methanol = 19/1) to give **BODIPY FL** (1.30 g, 72%) as a black solid.
R_f: 0.38 (silica gel, dichloromethane/methanol = 19/1, UV active; stains blue with modified phosphomolybdic acid);
mp: 176.5 °C (lit.[6] mp: 194–196 °C);
FT-IR (KBr) ν_{max}: 2923, 1703, 1604, 1250, 1140 cm^{-1};
^1H NMR (500 MHz, $CDCl_3$): δ 7.08 (s, 1H, H-8'), 6.88 (d, *J* = 4.0 Hz, 1H, H-1'), 6.28 (d, *J* = 4.0 Hz, 1H, H-2'), 6.11 (s, 1H, H-6'), 3.30 (t, *J* = 7.5 Hz, 2H, H-3), 2.83 (t, *J* = 7.5 Hz, 2H, H-2), 2.57 (s, 3H, 5'-Me), 2.25 (s, 3H, 7'-Me);
$^{13}C\{^1H\}$ NMR (125 MHz, $CDCl_3$): δ 177.2 (C-1), 160.9 (C-5'), 156.7 (C-3'), 144.1 (C-7'), 135.5 (C-7'a) , 133.4 (C-8'a), 128.1 (C-1'), 124.0 (C-8'), 120.7 (d, $^4J_{CF}$ = 2.4 Hz, C-6'), 116.8 (d, $^4J_{CF}$ = 3.6 Hz, C-2'), 33.1 (C-2), 23.8 (C-3), 15.1 (5'-Me), 11.5 (7'-Me);
$^{19}F\{^1H, ^{13}C\}$ NMR (470 MHz, $CDCl_3$): δ –146.35 (q, $^1J_{19F11B}$ = 32.9 Hz, 2F of ^{11}BODIPY), –146.28 (sep, $^1J_{19F10B}$ = 11.0 Hz, 2F of ^{10}BODIPY), $\Delta^{19}F(^{11}B^{10}B)$ = 0.07 ppm;
$^{11}B\{^1H, ^{13}C\}$ NMR (160 MHz, $CDCl_3$): δ 0.58 (t, $^1J_{11B19F}$ = 32.2 Hz, 1B);
HRMS: *m*/*z* $[M - H]^-$ calcd for $C_{14}H_{14}BF_2N_2O_2$: 291.1114; found: 291.1123.

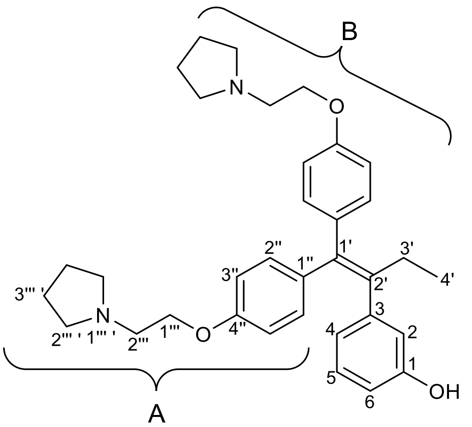

3-(1',1'-Bis(4''-(2'''-(pyrrolidin-1''' '-yl)ethoxy)phenyl)but-1'-en-2'-yl)phenol (RID-B-OH).
To a solution of **RID-B-OBn** (247.5 mg, 0.401 mmol) in ethyl acetate (13.4 mL), palladium 10% on carbon (M) (170.8 mg, 0.160 mmol) was added. The reaction mixture was stirred for 3 h at room temperature under an atmosphere of hydrogen (1.0 atm), and then transferred to an atmosphere of argon. After filtration of the mixture through a short pad of celite with ethyl acetate and concentration of the solvent, the residue was purified by preparative thin layer chromatography (eluant; ammoniacal chloroform/methanol = 9/1, R_f: 0.50) to give **RID-B-OH** (201.5 mg, 95%) as a yellow solid.
R_f: 0.50 (silica gel, ammoniacal chloroform/methanol = 9/1, UV active; stains blue with modified phosphomolybdic acid);
mp: 57.7 °C;
ATR-IR ν_{max}: 2954, 2785, 1600, 1505, 1238, 1031 cm^{-1};
^{1}H NMR (500 MHz, $CDCl_3$): δ 7.10 (d, J = 8.5 Hz, 2H, ^{B}H-2''), 7.06–6.92 (m, 1H, H-5), 6.86 (d, J = 8.5 Hz, 2H, ^{B}H-3''), 6.76 (d, J = 8.5 Hz, 2H, ^{A}H-2''), 6.64 (d, J = 7.5 Hz, 1H, H-2), 6.60–6.43 (m, 4H, H-4, H-6, ^{A}H-3''), 4.13 (t, J = 6.5 Hz, 2H, ^{B}H-1'''), 3.94 (t, J = 6.0 Hz, 2H, ^{A}H-1'''), 2.93 (t, J = 6.5 Hz, 2H, ^{B}H-2'''), 2.82 (t, J = 6.0 Hz, 2H, ^{A}H-2'''), 2.72–2.59 (m, 4H, ^{B}H-2''' '), 2.64–2.55 (m, 4H, ^{A}H-2''' '), 2.41 (q, J = 7.5 Hz, 2H, H-3'), 1.88–1.75 (m, 4H, ^{B}H-3''' '), 1.83–1.72 (m, 4H, ^{A}H-3''' '), 0.89 (t, J = 7.5 Hz, 3H, H-4');
^{13}C{^{1}H} NMR (125 MHz, $CDCl_3$): δ 157.5 (C-1), 156.8 (^{B}C-4''), 156.7 (^{A}C-4''), 144.3 (C-3), 141.1 (C-2'), 137.5 (C-1'), 136.6 (^{B}C-1''), 136.0 (^{A}C-1''), 131.9 (^{B}C-2''), 130.7 (^{A}C-2''), 129.1 (C-5), 121.2 (C-2), 117.1 (C-4), 114.1 (^{B}C-3''), 113.7 (^{A}C-3''), 113.4 (C-6), 66.8 (^{B}C-1'''), 66.1 (^{A}C-1'''), 55.2 (^{B}C-2'''), 55.0 (^{A}C-2'''), 54.8 (^{B}C-2''' '), 54.6 (^{A}C-2''' '), 29.3 (C-3'), 23.5 (^{B}C-3''' '), 23.4 (^{A}C-3''' '), 13.8 (C-4');
HRMS: m/z [M + H]$^+$ calcd for $C_{34}H_{43}N_2O_3$: 527.3268; found: 527.3286.

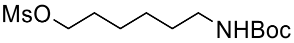

6-((*tert*-Butoxycarbonyl)amino)hexyl methanesulfonate (Spacer).
To a solution of *tert*-butyl (6-hydroxyhexyl)carbamate (200.6 mg, 0.923 mmol) in dichloromethane (9.23 mL), triethylamine (0.772 mL, 5.54 mmol) and methanesulfonyl chloride (0.214 mL, 2.77 mmol) were successively added at 0 °C. After the reaction mixture was stirred for 1 h at room temperature, the reaction was quenched by a saturated sodium hydrogen carbonate. Two layers were separated, and the aqueous layer was extracted with dichloromethane. The combined organic layers were washed with brine and dried over sodium sulfate. After filtration of the mixture and concentration of the solvent, the residue was purified by silica gel column chromatography (eluant; hexane/ethyl acetate = 3/1) to give **Spacer** (261 mg, 96%) as a white solid.
R_f: 0.20 (silica gel, hexane/ethyl acetate = 2/1, UV active; stains blue with modified phosphomolybdic acid);

mp: 41.0 °C (lit.[7] mp: 44–45 °C);
ATR-IR ν_{max}: 3404, 2974, 2862, 1696, 1452, 1352, 1173 cm^{-1};
^{1}H NMR (500 MHz, DMSO-d_6): δ 6.86–6.67 (m, 1H, NH), 4.17 (t, J = 6.5 Hz, 2H, H-1), 3.15 (s, 3H, MeS), 2.89 (dt, J = 6.5, 6.5 Hz, 2H, H-6), 1.64 (tt, J = 7.0, 6.5 Hz, 2H, H-5), 1.37 (s, 9H, C(CH_3)$_3$), 1.38–1.21 (m, 6H, H-2, H-3, H-4);
^{13}C{^{1}H} NMR (125 MHz, DMSO-d_6): δ 155.6 (C=O), 77.3 (C(CH$_3$)$_3$), 70.4 (C-1), 36.5 (MeS), 31.5 (CN), 29.3 (C-5), 28.5 (C-2), 28.3 (C(CH$_3$)$_3$), 25.7 (C-4), 24.6 (C-3);
HRMS: m/z [M + Na]$^+$ calcd for $C_{12}H_{25}NO_5SNa$: 318.1346; found: 318.1353.

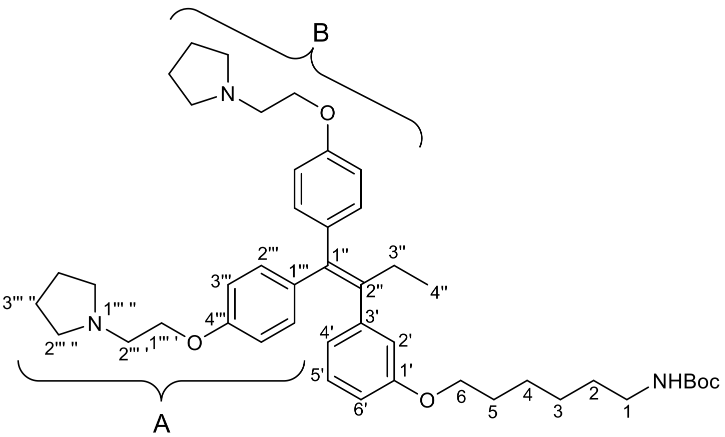

***tert*-Butyl (6-(3’-(1’’,1’’-bis(4’’’-(2’’’ ’-(pyrrolidin-1’’’ ’’-yl)ethoxy)phenyl)but-1’’-en-2’’-yl)phenoxy)hexyl)carbamate (1).**

To a solution of **RID-B-OH** (246.9 mg, 0.469 mmol) in *N*,*N*-dimethylformamide (7.81 mL), 55% sodium hydride (dispersion in paraffin liquid, 40.9 mg, 0.938 mmol) and **Spacer** (275.1 mg, 0.938 mmol) were successively added at 0 °C. After the reaction mixture was stirred for 2 h at room temperature. the reaction was quenched by brine. Two layers were separated, and the aqueous layer was extracted with diethyl eater. The combined organic layers were dried over sodium sulfate. After filtration of the mixture and concentration of the solvent, the residue was purified by preparative thin layer chromatography (eluant; ammoniacal chloroform/methanol = 9/1, R_f: 0.95) to give crude of **1**. The crude was purified by preparative thin layer chromatography (eluant; chloroform/methanol = 9/1, R_f: 0.40) to give **1** (267.8 mg, 79%) as a pale yellow oil.
R_f: 0.40 (silica gel, chloroform/methanol = 9/1, UV active; stains blue with modified phosphomolybdic acid);
FT-IR (neat) ν_{max}: 3379, 3039, 2962, 2931, 2792, 1075, 1604, 1512, 1242, 1173, 1041, 833 cm^{-1};
^{1}H NMR (500 MHz, CDCl$_3$): δ 7.17–7.05 (m, 2H, ^{B}H-2’’’), 7.10–7.01 (m, 1H, H-5’), 6.94–6.84 (m, 2H, ^{B}H-3’’’), 6.81–6.72 (m, 2H, ^{A}H-2’’’), 6.68 (d, J = 7.5 Hz, 1H, H-2’), 6.68–6.57 (m, 2H, H-4’, H-6’), 6.62–6.50 (m, 2H, ^{A}H-3’’’), 4.69 (brs, 1H, NH), 4.12 (t, J = 6.5 Hz, 2H, ^{B}H-1’’’ ’), 3.96 (t, J = 6.0 Hz, 2H, ^{A}H-1’’’ ’), 3.77 (t, J = 6.5 Hz, 2H, H-6), 3.18–3.04 (m, 2H, H-1), 2.91 (t, J = 6.5 Hz, 2H, ^{B}H-2’’’ ’), 2.81 (t, J = 6.0 Hz, 2H, ^{A}H-2’’’ ’), 2.68–2.56 (brm, 4H, ^{B}H-2’’’ ’’), 2.63–2.51 (brm, 4H, ^{A}H-2’’’ ’’), 2.46 (q, J = 7.5 Hz, 2H, H-3’’), 1.86–1.76 (m, 4H, ^{B}H-3’’’ ’’), 1.81–1.72 (m, 4H, ^{A}H-3’’’ ’’), 1.67 (tt, J = 7.0, 6.5 Hz, 2H, H-5), 1.59–1.23 (m, 15H, H-2, H-3, H-4, C(CH_3)$_3$), 0.93 (t, J = 7.5 Hz, 3H, H-4’’);
^{13}C{^{1}H} NMR (125 MHz, CDCl$_3$): δ 158.7 (C-1’), 157.7 (^{B}C-4’’’), 156.9 (^{A}C-4’’’), 156.2 (C=O), 144.1 (C-3’), 141.0 (C-2’’), 137.9 (C-1’’), 136.4 (^{B}C-1’’’), 136.1 (^{A}C-1’’’), 132.0 (^{B}C-2’’’), 130.7 (^{A}C-2’’’), 128.8 (C-5’), 122.3 (C-2’), 116.0 (C-4’), 114.2 (^{B}C-3’’’), 113.5 (^{A}C-3’’’), 112.6 (C-6’), 79.1 (C(CH$_3$)$_3$), 67.7 (C-6), 67.1 (^{B}C-1’’’ ’), 66.9 (^{A}C-1’’’ ’), 55.30 (^{B}C-2’’’ ’), 55.25 (^{A}C-2’’’ ’),

54.9 (^{B}C-2''' ''), 54.8 (^{A}C-2''' ''), 40.7 (C-1), 30.2 (C-2), 29.2 (C-5), 29.1 (C-3''), 28.6 (C(CH$_3$)$_3$), 26.7 (C-3), 25.9 (C-4), 23.63 (^{B}C-3''' ''), 23.59 (^{A}C-3''' ''), 13.8 (C-4'');
HRMS: *m/z* [M + H]$^+$ calcd for $C_{45}H_{64}N_3O_5$: 726.4840; found: 726.4816.

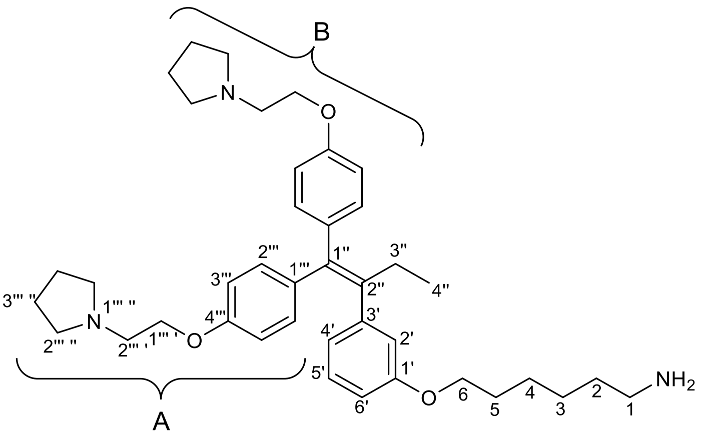

6-(3'-(1'',1''-Bis(4'''-(2''' '-(pyrrolidin-1''' ''-yl)ethoxy)phenyl)but-1''-en-2''-yl)phenoxy)hexan-1-amine (2).

To a solution of **1** (66.7 mg, 0.0919 mmol) in dichloromethane (0.92 mL), methanesulfonic acid (59.6 μL, 0.919 mmol) was added at 0 °C. After the reaction mixture stirred for 30 min, the reaction was quenched by a saturated aqueous sodium hydrogen carbonate. Two layers were separated, and the aqueous layer was extracted with dichloromethane. The combined organic layers were dried over sodium sulfate. After filtration of the mixture and concentration of the solvent, the residue was purified by preparative thin layer chromatography (eluant; ammoniacal chloroform/methanol = 30/2, R_f: 0.50) to give **2** (56.7 mg, 99%) as a pale yellow oil.

R_f: 0.50 (silica gel, ammoniacal chloroform/methanol = 30/2, UV active; stains blue with modified phosphomolybdic acid);

FT-IR (neat) ν_{max}: 3371, 3039, 2931, 2785, 1604, 1504, 1242, 1173, 1041, 833 cm^{-1};

^{1}H NMR (500 MHz, CDCl$_3$): δ 7.18–7.04 (m, 2H, ^{B}H-2'''), 7.10–6.99 (m, 1H, H-5'), 6.94–6.82 (m, 2H, ^{B}H-3'''), 6.83–6.71 (m, 2H, ^{A}H-2'''), 6.67 (d, J = 7.5 Hz, 1H, H-2'), 6.68–6.58 (m, 2H, H-4', H-6'), 6.63–6.49 (m, 2H, ^{A}H-3'''), 4.12 (t, J = 6.0 Hz, 2H, ^{B}H-1''' '), 3.97 (t, J = 6.5 Hz, 2H, ^{A}H-1''' '), 3.78 (t, J = 6.5 Hz, 2H, H-6), 2.91 (t, J = 6.0 Hz, 2H, ^{B}H-2''' '), 2.82 (t, J = 6.5 Hz, 2H, ^{A}H-2''' '), 2.80–2.65 (m, 2H, H-1), 2.74–2.48 (brm, 8H, ^{B}H-2''' '', ^{A}H-2''' ''), 2.45 (q, J = 7.5 Hz, 2H, H-3''), 1.90–1.65 (brm, 8H, ^{B}H-3''' '', ^{A}H-3''' ''), 1.68 (tt, J = 7.5, 6.5 Hz, 2H, H-5), 1.55–1.25 (m, 6H, H-2, H-3, H-4), 0.92 (t, J = 7.5 Hz, 3H, H-4'');

^{13}C{^{1}H} NMR (125 MHz, CDCl$_3$): δ 158.8 (C-1'), 157.7 (^{B}C-4'''), 156.9 (^{A}C-4'''), 144.1 (C-3'), 141.0 (C-2''), 137.9 (C-1''), 136.4 (^{B}C-1'''), 136.1 (^{A}C-1'''), 131.9 (^{B}C-2'''), 130.7 (^{A}C-2'''), 128.8 (C-5'), 122.3 (C-2'), 115.9 (C-4'), 114.2 (^{B}C-3'''), 113.5 (^{A}C-3'''), 112.6 (C-6'), 67.8 (C-6), 67.1 (^{B}C-1''' '), 66.9 (^{A}C-1''' '), 55.3 (^{B}C-2''' '), 55.2 (^{A}C-2''' '), 54.9 (^{B}C-2''' ''), 54.8 (^{A}C-2''' ''), 48.0 (C-1), 29.3 (C-2), 29.2 (C-5), 29.1 (C-3''), 26.8 (C-3), 26.1 (C-4), 23.62 (^{B}C-3''' ''), 23.57 (^{A}C-3''' ''), 13.8 (C-4'');

HRMS: *m/z* [M + H]$^+$ calcd for $C_{40}H_{56}N_3O_3$: 626.4316; found 626.4289.

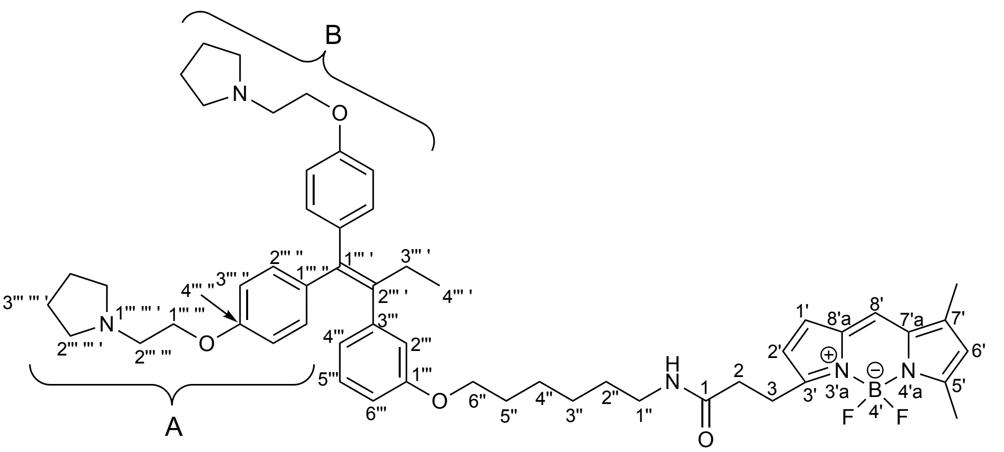

***N*-(6’’-(3’’’-(1’’’ ’,1’’’ ’-Bis(4’’’ ’’-(2’’’ ’’’-(pyrrolidin-1’’’ ’’’ ’-yl)ethoxy)phenyl)but-1’’’ ’-en-2’’’ ’-yl)phenoxy)hexyl)-3-[4’,4’-difluoro-5’,7’-dimethyl-4’-bora-3’a,4’a-diaza-*s*-indacene-3’-yl]propanamide (RID-B-BODIPY).**

To a solution of 2-methyl-6-nitrobenzoic anhydride (MNBA) (30.0 mg, 0.0872 mmol) in dichloromethane (0.6 mL), *N,N*-dimethylpyridin-4-amine (DMAP) (0.9 mg, 0.0727 mmol), triethylamine (22.4 μL, 0.160 mmol) and **BODIPY FL** (21.2 mg, 0.0727 mmol) was successively added at 0 °C. After the reaction mixture was stirred for 10 min, a solution of **2** (54.6 mg, .0.0872 mmol) in dichloromethane (0.4 mL) was added. The reaction mixture was stirred for 3 h at room temperature, the reaction was quenched by a saturated aqueous sodium hydrogen carbonate. Two layers were separated, and the aqueous layer was extracted with dichloromethane. The combined organic layers were dried over sodium sulfate. After filtration of the mixture and concentration of the solvent, the residue was purified by preparative thin layer chromatography (eluant; ammoniacal chloroform/methanol = 30/1, R_f: 0.95) to give **RID-B-BODIPY** (55.4 mg, 85%) as a red solid.

R_f: 0.80 (silica gel, ammoniacal chloroform/methanol = 30/1, UV active; stains blue with modified phosphomolybdic acid);

mp: 51.1 °C;

FT-IR (KBr) ν_{max}: 3417, 3309, 3062, 2931, 2870, 1651, 1604, 1242, 1134 cm^{-1};

^{1}H NMR (500 MHz, $CDCl_3$): δ 7.20–7.03 (m, 2H, ^{B}H-2’’’ ’’), 7.15–6.93 (m, 2H, H-8’, H-5’’’), 6.97–6.77 (m, 3H, H-1’, ^{B}H-3’’’ ’’), 6.85–6.70 (m, 2H, ^{A}H-2’’’ ’’), 6.69 (d, *J* = 8.0 Hz, 1H, H-2’’’), 6.70–6.54 (m, 2H, H-4’’’, H-6’’’), 6.56–6.53 (m, 2H, ^{A}H-3’’’ ’’), 6.29 (d, *J* = 4.0 Hz, 1H, H-2’), 6.10 (s, 1H, H-6’), 5.83 (brt, *J* = 6.0 Hz, 1H, NH), 4.14 (t, *J* = 6.0 Hz, 2H ^{B}H-1’’’ ’’’), 3.96 (t, *J* = 6.0 Hz, 2H, ^{A}H-1’’’ ’’’), 3.74 (t, *J* = 6.5 Hz, 2H, H-6’’), 3.27 (t, *J* = 7.5 Hz, 2H, H-3), 3.19 (td, *J* = 6.5, 6.0 Hz, 2H, H-1’’), 2.94 (t, *J* = 6.0 Hz, 2H, ^{B}H-2’’’ ’’’), 2.83 (t, *J* = 6.0 Hz, 2H, ^{A}H-2’’’ ’’’), 2.79–2.50 (m, 8H, ^{B}H-2’’’ ’’’ ’, ^{A}H-2’’’ ’’’ ’), 2.63 (brt, *J* = 7.5 Hz, 2H, H-2), 2.56 (s, 3H, 5’-Me), 2.45 (q, *J* = 7.5 Hz, 2H, H-3’’’ ’), 2.23 (s, 3H, 7’-Me), 1.95–1.65 (brm, 8H, ^{B}H-3’’’ ’’’ ’, ^{A}H-3’’’ ’’’ ’), 1.61 (tt, *J* = 7.5, 6.5 Hz, 2H, H-5’’), 1.55–1.20 (m, 4H, H-2’’, H-4’’), 1.35–1.15 (m, 2H, H-3’’), 0.93 (t, *J* = 7.5 Hz, 3H, H-4’’’ ’);

^{13}C{^{1}H} NMR (125 MHz, $CDCl_3$): δ 171.7 (C-1), 160.3 (C-5’), 158.7 (C-1’’’), 157.65 (C-3’), 157.61 (^{B}C-4’’’ ’’), 156.8 (^{A}C-4’’’ ’’), 144.2 (C-3’’’), 144.0 (C-7’), 141.0 (C-2’’’ ’), 137.9 (C-1’’’ ’), 136.5 (^{B}C-1’’’ ’’), 136.1 (^{A}C-1’’’ ’’), 135.2 (C-7’a), 133.5 (C-8’a), 131.9 (^{B}C-2’’’ ’’), 130.7 (^{A}C-2’’’ ’’), 128.9 (C-5’’’), 128.5 (C-1’), 124.0 (C-8’), 122.3 (C-2’’’), 120.5 (C-6’), 117.8 (C-2’), 116.0 (C-4’’’), 114.2 (^{B}C-3’’’ ’’), 113.5 (^{A}C-3’’’ ’’), 112.5 (C-6’’’), 67.8 (C-6’’), 66.9 (^{B}C-1’’’ ’’’), 66.6 (^{A}C-1’’’ ’’’), 55.2 (^{B}C-2’’’ ’’’), 55.1 (^{A}C-2’’’ ’’’), 54.9 (^{B}C-2’’’ ’’’ ’), 54.8 (^{A}C-2’’’ ’’’ ’), 39.5 (C-1’’), 36.2 (C-2), 29.6 (C-2’’), 29.2 (C-5’’), 29.1 (C-3’’’ ’), 26.7 (C-3’’), 25.9 (C-4’’), 25.1 (C-3), 23.62 (^{B}C-3’’’ ’’’ ’), 23.57 (^{A}C-3’’’ ’’’ ’), 15.1 (5’-Me), 13.8 (C-4’’’ ’), 11.5 (7’-Me);

$^{19}F\{^1H, ^{13}C\}$ NMR (470 MHz, $CDCl_3$): δ –145.35 (q, $^1J_{19F11B}$ = 32.9 Hz, 2F of ^{11}BODIPY), –145.28 (sep, $^1J_{19F10B}$ = 11.0 Hz, 2F of ^{10}BODIPY), $\Delta^{19}F(^{11}B^{10}B)$ = 0.07 ppm;
HRMS: *m/z* $[M + H]^+$ calcd for $C_{54}H_{69}BF_2N_5O_4$: 900.5414; found: 900.5390.

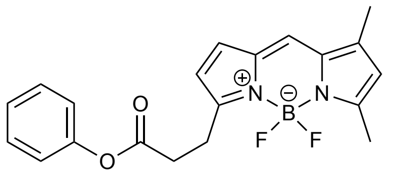

Phenyl 3-(4',4'-difluoro-5',7'-dimethyl-4'-bora-3'a,4'a-diaza-*s*-indacene-3'-yl)propionate (Phenol-BODIPY)

To a solution of 2-methyl-6-nitrobenzoic anhydride (MNBA) (56.6 mg, 0.1643 mmol), *N*,*N*-dimethylpyridin-4-amine (DMAP) (1.7 mg, 0.0137 mmol) and **BODIPY FL** (40.0 mg, 0.1369mol) in dichloromethane (2.0 mL), triethylamine (42.1 μL, 0.3012 mmol) was added at 0 °C. After the reaction mixture was stirred for 10 min, **Phenol** (12.9 mg, 0.1643 mmol) was added at 0 °C. The reaction mixture was stirred for 3 h at room temperature, the reaction was quenched by a saturated aqueous ammonium chloride. Two layers were separated, and the aqueous layer was extracted with dichloromethane. The combined organic layers were dried over sodium sulfate. After filtration of the mixture and concentration of the solvent, the residue was purified by preparative thin layer chromatography (eluant; hexane/ethyl acetate = 3/1, R_f: 0.40) to give **Phenol-BODIPY** (32.2 mg, 82%) as a red solid.

Phenol-BODIPY is solid at room temperature, but melting point was 25–30 °C to make immeasurable accurately.

R_f: 0.40 (silica gel, hexane/ethyl acetate = 3/1, UV active; stains blue with modified phosphomolybdic acid);

FT-IR (KBr) v_{max}: 2921, 1758, 1607, 1194, 1135 cm^{-1};

^{1}H NMR (500 MHz, $CDCl_3$): δ 7.43–7.31 (m, 2H, H-3''), 7.25–7.18 (m, 1H, H-4''), 7.11 (s, 1H, H-8'), 7.12–7.03 (m, 2H, H-2''), 6.91 (d, J = 4.0 Hz, 1H, H-1'), 6.35 (d, J = 4.0 Hz, 1H, H-2'), 6.13 (s, 1H, H-6'), 3.41 (t, J = 7.5 Hz, 2H, H-3), 3.03 (t, J = 7.5 Hz, 2H, H-2), 2.58 (s, 3H, 5'-Me), 2.26 (s, 3H, 7'-Me);

$^{13}C\{^1H\}$ NMR (125 MHz, $CDCl_3$): δ 171.2 (C-1), 160.8 (C-5'), 156.6 (C-3'), 150.8 (C-1''), 144.2 (C-7'), 135.5 (C-7'a), 133.5 (C-8'a), 129.5 (C-3''), 128.1 (C-1'), 125.9 (C-4''), 124.0 (C-8'), 121.7 (C-2''), 120.7 (d, $^4J_{CF}$ =3.5 Hz, C-6'), 117.0 (d, $^4J_{CF}$ = 2.4 Hz, C-2'), 33.8 (C-2), 24.0 (C-3), 15.1 (5'-Me), 11.4 (7'-Me);

$^{19}F\{^1H, ^{13}C\}$ NMR (470 MHz, $CDCl_3$): δ –146.15 (q, $^1J_{19F11B}$ = 32.9 Hz, 2F of ^{11}BODIPY), –146.08 (sep, $^1J_{19F10B}$ = 11.0 Hz, 2F of ^{10}BODIPY), $\Delta^{19}F(^{11}B^{10}B)$ = 0.07 ppm;

HRMS: *m/z* $[M + Na]^+$ calcd for $C_{20}H_{19}BF_2N_2O_2Na$: 391.1403; found: 319.1409.

References.

(1) Lin, C.-F.; Yang, J.-S.; Chang, C.-Y.; Kuo, S.-C.; Lee, M.-R.; Huang, L.-J. Synthesis and anticancer activity of benzyloxybenzaldehyde derivatives against HL-60 cells. *Bioorg. Med. Chem.* **2005**, *13*, 1537–1544.
(2) Krasovskiy, A.; Knochel, P.; Convenient Titration Method for Organometallic Zinc, Magnesium, and Lanthanide Reagents. *Synthesis* **2006**, *5*, 890–891.
(3) Erwin, B.; 1-(úÝ-hydroxyphenyl)-1-hydroxy-2-aralkylaminopropanes, **1994**, GB711905A.
(4) Tsukuda, S.; Kusayanagi, T.; Umeda, E.; Watanabe, C.; Tosaki, Y.; Kamisuki, S.; Takeuchi, T.; Takakusagi, Y.; Shiina, I.; Sugawara, F. Ridaifen B, a tamoxifen derivative, directly binds to Grb10 interacting GYF protein 2. *Bioorg. Med. Chem.* **2013**, *21*, 311–320.

(5) Marínez-Mora, E. I.; Caracas, M. A.; Escalante, C. H.; Madrigal, D. A.; Quiroz-Florentino, H.; Delgado, F.;Tamariz, J. Divergent and Selective Functionalization of 2-Formylpyrrole and its Application in the Total synthesis of the Aglycone Alkaloid Pyrrolemarumine. *J. Mex. Chem. Soc.* **2016**, *60*, 23–33.

(6) Gießler. K.; Griesser. H.; Göhringer. D.; Sabirov. T.; Richert. C. Synthesis of 3'-BODIPY-Labeled Active Esters of Nucleotides and a Chemical Primer Extension Assay on Beads. *Eur. J. Org. Chem*. **2010**, *19*, 3611–3620.

(7) Keshavarz, A.; Riahinasab, S. T.; Hirst, L. S.; Stokes, B. J. New Promesogenic Ligands for Host Medium Microencapsulation by Quantum Dots via Liquid Crystal Phase Transition Templating. *ACS Appl. Nano Mater*. **2019**, *2*, 2542−2547.

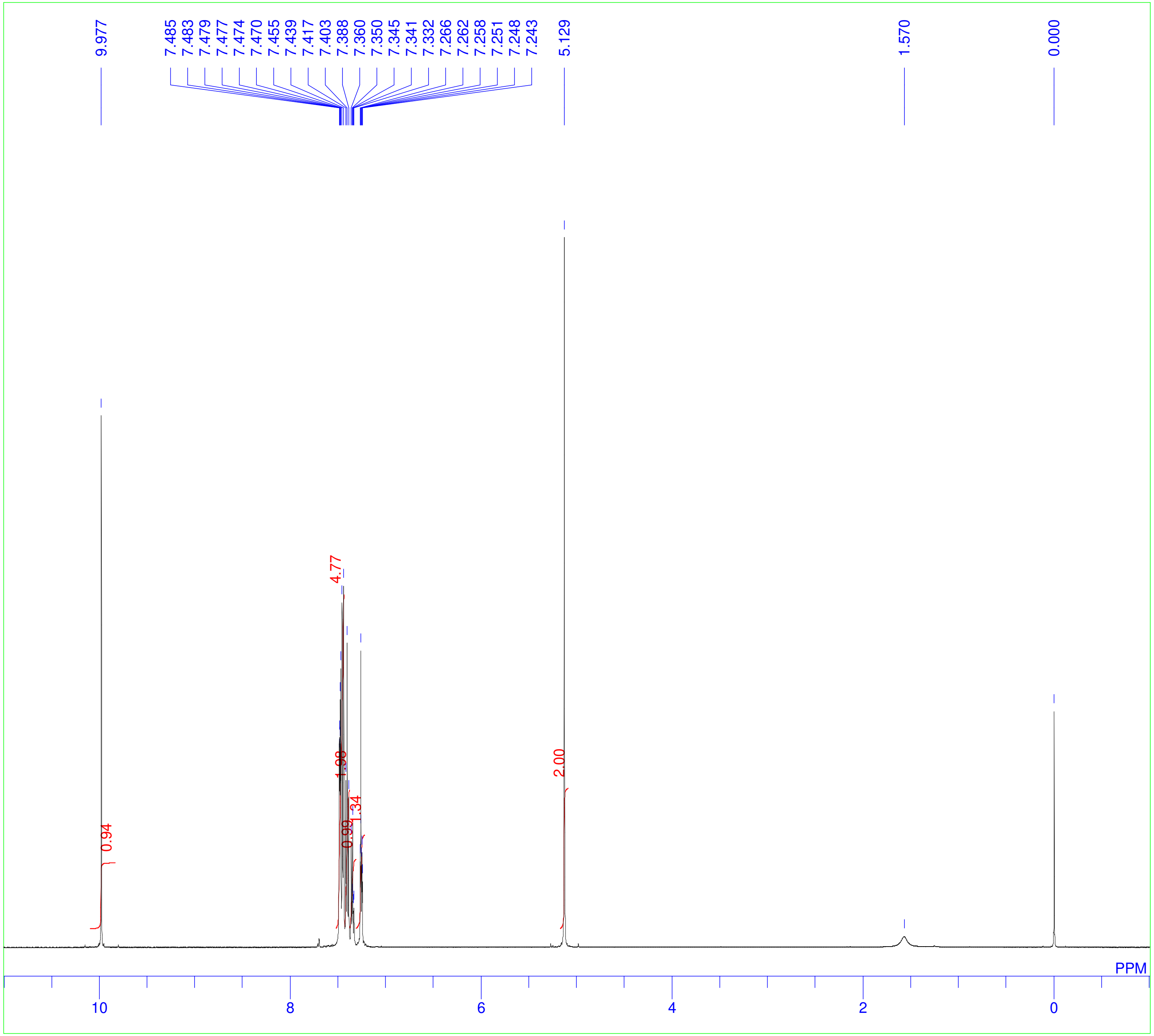

DFILE	S1_Proton.als
COMNT	
DATIM	2024-04-24 11:54:38
OBNUC	1H
EXMOD	proton.jxp
OBFRQ	500.16 MHz
OBSET	2.41 KHz
OBFIN	6.01 Hz
POINT	13107
FREQU	7507.51 Hz
SCANS	8
ACQTM	1.7459 sec
PD	5.0000 sec
PW1	3.80 usec
IRNUC	1H
CTEMP	23.6 c
SLVNT	CDCL3
EXREF	0.00 ppm
BF	0.30 Hz
RGAIN	40

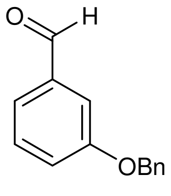

S1

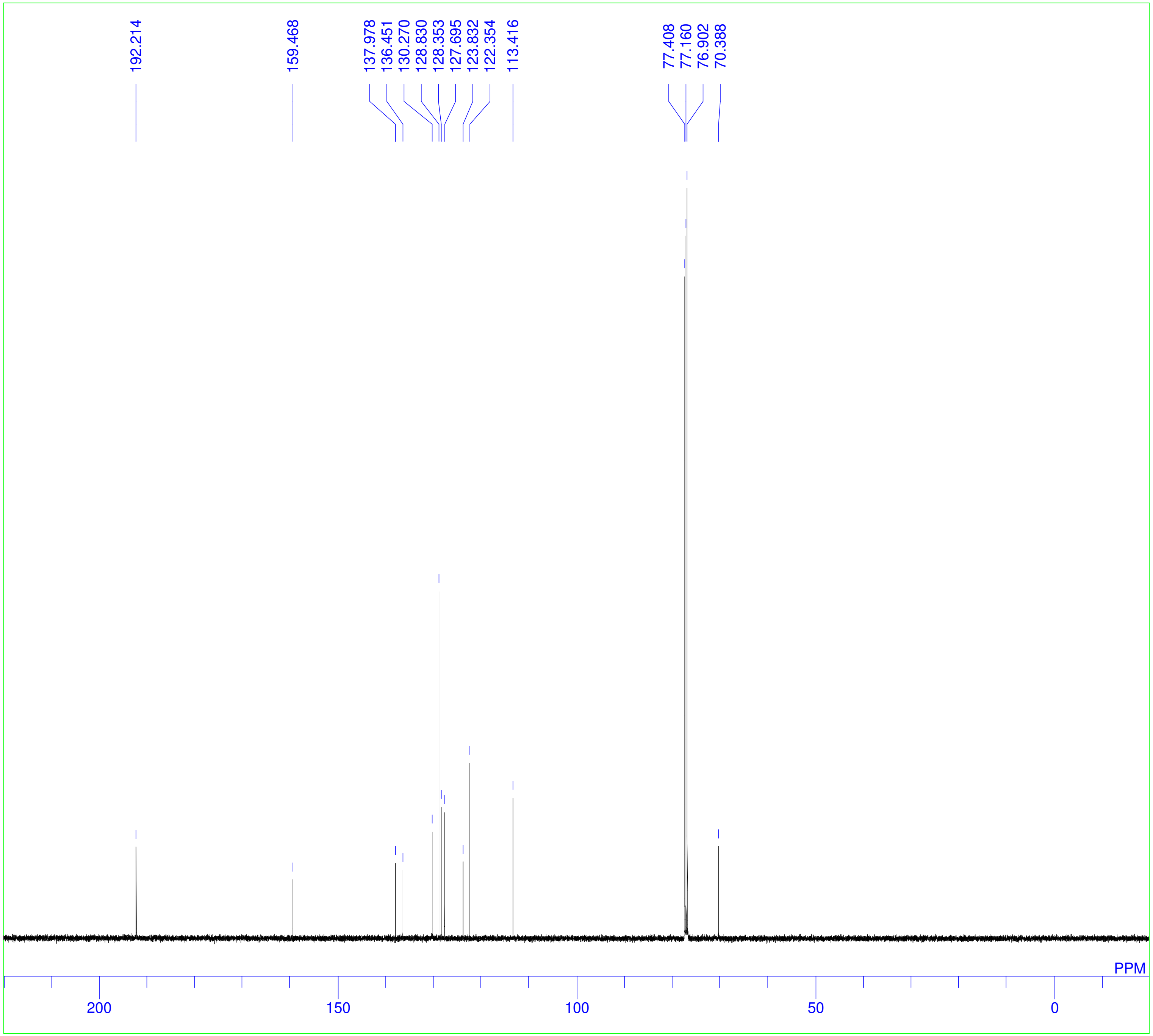

DFILE	S1_Carbon.als
COMNT	
DATIM	2024-04-24 18:26:48
OBNUC	13C
EXMOD	carbon.jxp
OBFRQ	125.77 MHz
OBSET	7.87 KHz
OBFIN	4.21 Hz
POINT	26214
FREQU	31446.54 Hz
SCANS	2048
ACQTM	0.8336 sec
PD	2.0000 sec
PW1	4.30 usec
IRNUC	1H
CTEMP	24.0 c
SLVNT	ACETN
EXREF	77.16 ppm
BF	0.30 Hz
RGAIN	30

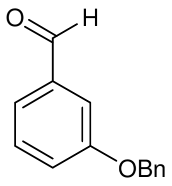

S1

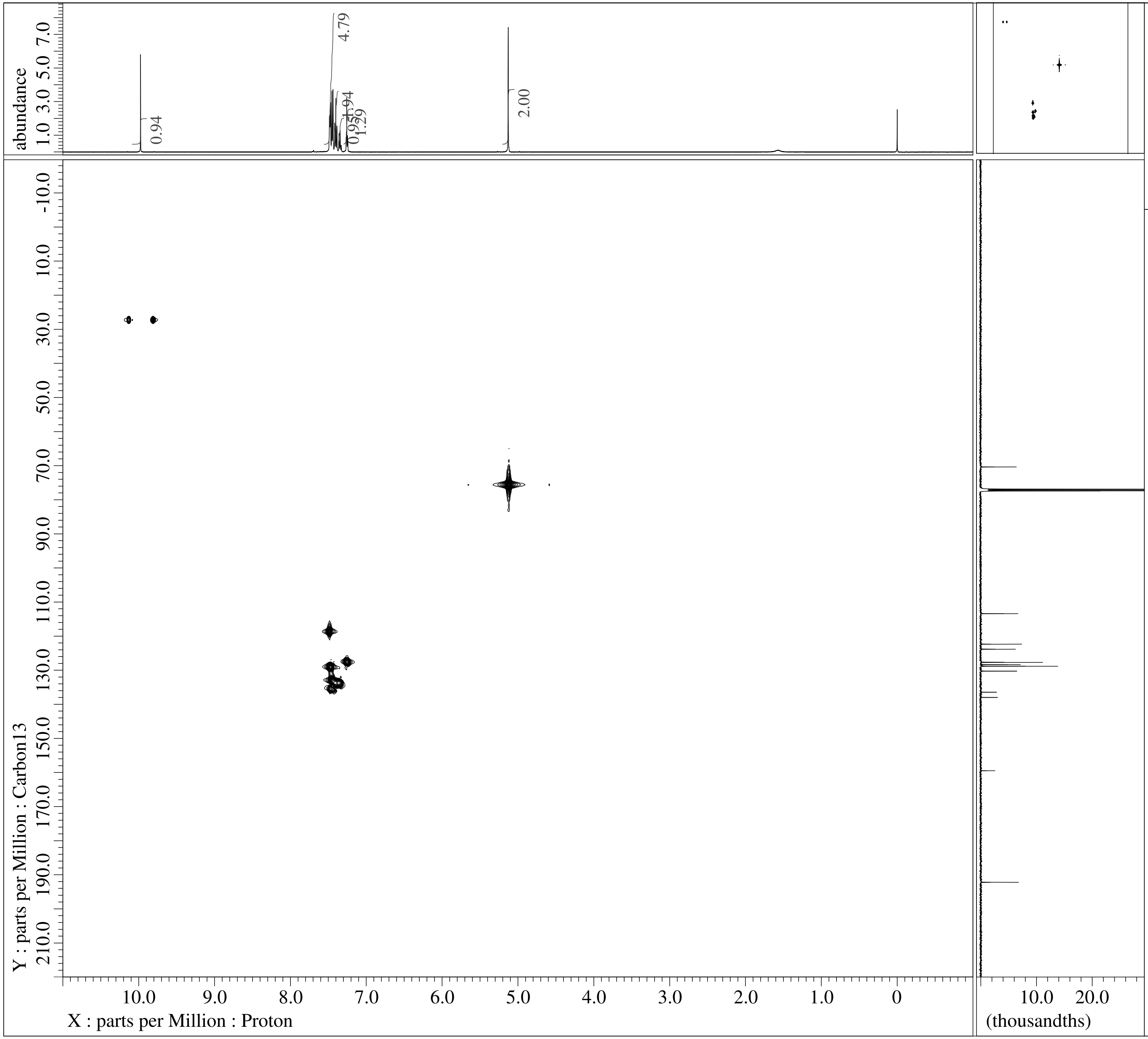


```
---- PROCESSING PARAMETERS ----
dc_balance( 0, FALSE )
sexp( 0.2[Hz], 0.0[s] )
trapezoid( 0[%], 0[%], 80[%], 100[%] )
zerofill( 1 )
fft( 1, TRUE, TRUE )
machinephase
ppm
```

```
Filename            = S1_HMQC-1-4.jdf
Author              = delta
Experiment          = hmqc.jxp
Sample_Id           = FS-RID-aldehyde
Solvent             = CHLOROFORM-D
Creation_Time       = 23-MAY-2024 17:37:55
Revision_Time       = 17-JUN-2024 17:58:01
Current_Time        = 17-JUN-2024 18:02:09

Comment             = gradient enhanced HMQC
Data_Format         = 2D REAL REAL
Dim_Size            = 819, 512
Dim_Title           = Proton Carbon13
Dim_Units           = [ppm] [ppm]
Dimensions          = X Y
Site                = JNM-ECA500II
Spectrometer        = DELTA2_NMR

Field_Strength      = 11.7473579[T] (500[MHz]
X_Acq_Duration      = 0.10911744[s]
X_Domain            = 1H
X_Freq              = 500.15991521[MHz]
X_Offset            = 5.0[ppm]
X_Points            = 1024
X_Prescans          = 4
X_Resolution        = 9.16443788[Hz]
X_Sweep             = 9.38438438[kHz]
X_Sweep_Clipped     = 7.50750751[kHz]
Y_Domain            = 13C
Y_Freq              = 125.76529768[MHz]
Y_Offset            = 85[ppm]
Y_Points            = 256
Y_Prescans          = 0
Y_Resolution        = 83.60980308[Hz]
Y_Sweep             = 21.40410959[kHz]
Tri_Domain          = Proton
Tri_Freq            = 500.15991521[MHz]
Tri_Offset          = 5.0[ppm]
Clipped             = FALSE
Scans               = 4
Total_Scans         = 1024

Relaxation_Delay    = 1.5[s]
Recvr_Gain          = 50
Temp_Get            = 24[dC]
X_Acq_Time          = 0.10911744[s]
X_Atn               = 3.2[dB]
X_Gamma             = 42576375
X_Pulse             = 7.59[us]
```

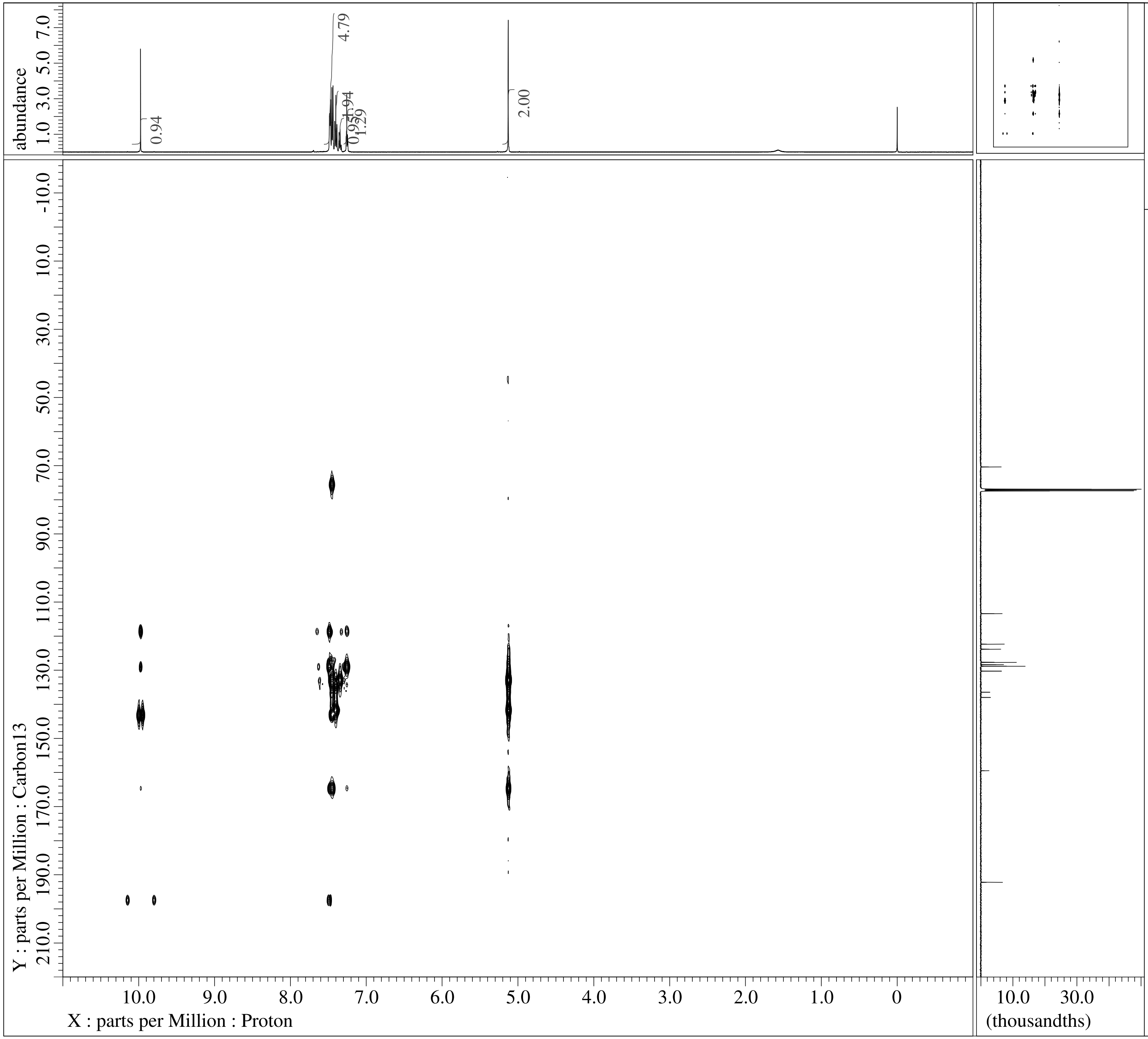


```
---- PROCESSING PARAMETERS ----
dc_balance( 0, FALSE )
sexp( 2.0[Hz], 0.0[s] )
trapezoid( 0[%], 0[%], 80[%], 100[%] )
zerofill( 1, TRUE )
fft( 1, TRUE, TRUE )
machinephase
ppm
```

```
Filename            = S1_HMBC-1-4.jdf
Author              = delta
Experiment          = hmbc.jxp
Sample_Id           = FS-RID-aldehyde
Solvent             = CHLOROFORM-D
Creation_Time       = 23-MAY-2024 18:06:15
Revision_Time       = 17-JUN-2024 18:10:29
Current_Time        = 17-JUN-2024 18:11:44

Comment             = gradient enhanced HMBC
Data_Format         = 2D REAL REAL
Dim_Size            = 1638, 512
Dim_Title           = Proton Carbon13
Dim_Units           = [ppm] [ppm]
Dimensions          = X Y
Site                = JNM-ECA500II
Spectrometer        = DELTA2_NMR

Field_Strength      = 11.7473579[T] (500[MHz]
X_Acq_Duration      = 0.21823488[s]
X_Domain            = 1H
X_Freq              = 500.15991521[MHz]
X_Offset            = 5.0[ppm]
X_Points            = 2048
X_Prescans          = 4
X_Resolution        = 4.58221894[Hz]
X_Sweep             = 9.38438438[kHz]
X_Sweep_Clipped     = 7.50750751[kHz]
Y_Domain            = 13C
Y_Freq              = 125.76529768[MHz]
Y_Offset            = 100[ppm]
Y_Points            = 256
Y_Prescans          = 0
Y_Resolution        = 122.99275819[Hz]
Y_Sweep             = 31.4861461[kHz]
Tri_Domain          = Proton
Tri_Freq            = 500.15991521[MHz]
Tri_Offset          = 5.0[ppm]
Clipped             = FALSE
Scans               = 8
Total_Scans         = 2048

Relaxation_Delay    = 1.5[s]
Recvr_Gain          = 50
Temp_Get            = 23.8[dC]
X_Acq_Time          = 0.21823488[s]
X_Atn               = 3.2[dB]
X_Gamma             = 42576375
X_Pulse             = 7.59[us]
```

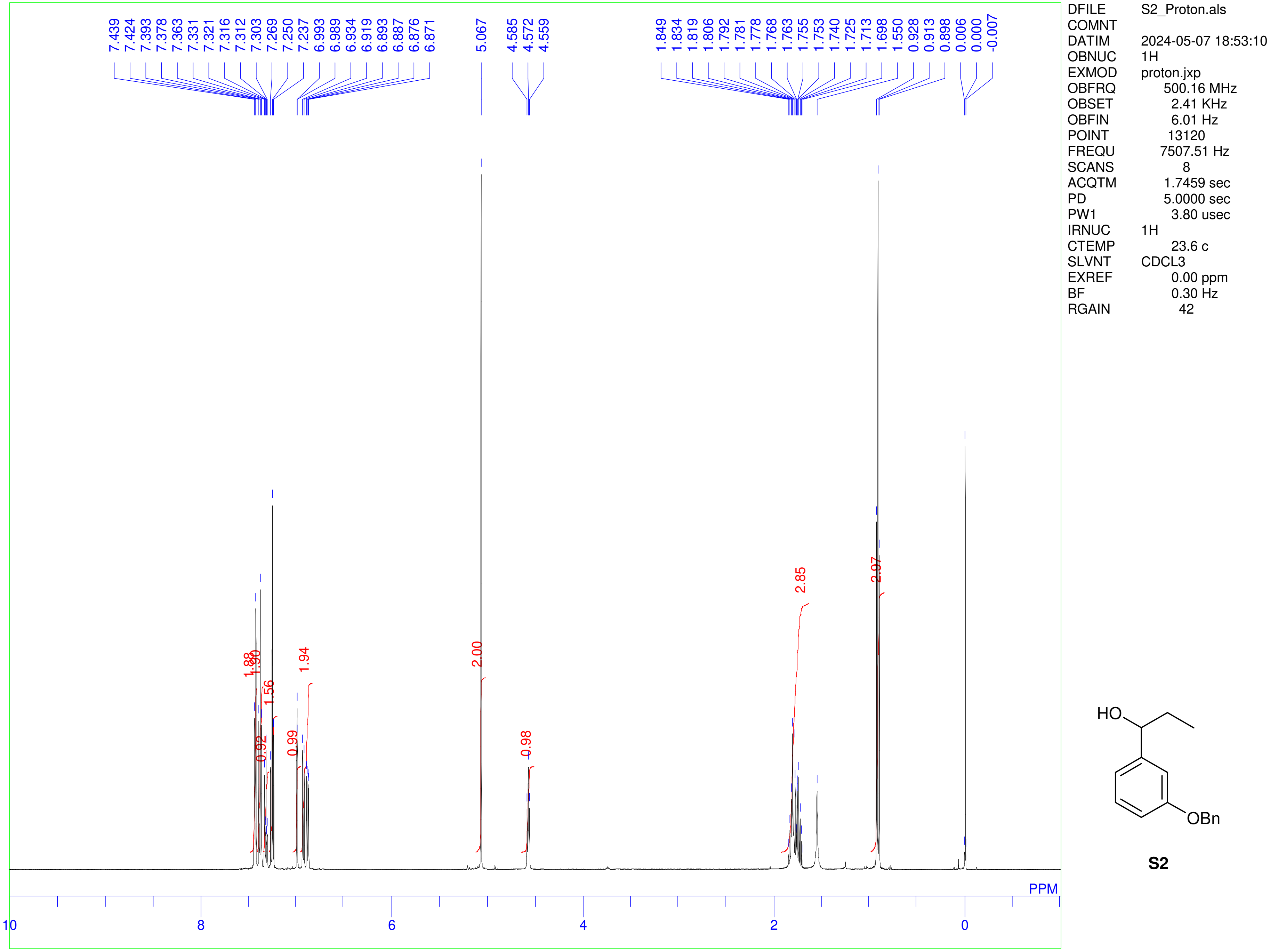
DFILE S2_Proton.als
COMNT
DATIM 2024-05-07 18:53:10
OBNUC 1H
EXMOD proton.jxp
OBFRQ 500.16 MHz
OBSET 2.41 KHz
OBFIN 6.01 Hz
POINT 13120
FREQU 7507.51 Hz
SCANS 8
ACQTM 1.7459 sec
PD 5.0000 sec
PW1 3.80 usec
IRNUC 1H
CTEMP 23.6 c
SLVNT CDCL3
EXREF 0.00 ppm
BF 0.30 Hz
RGAIN 42
PPM
HO
OBn

S2

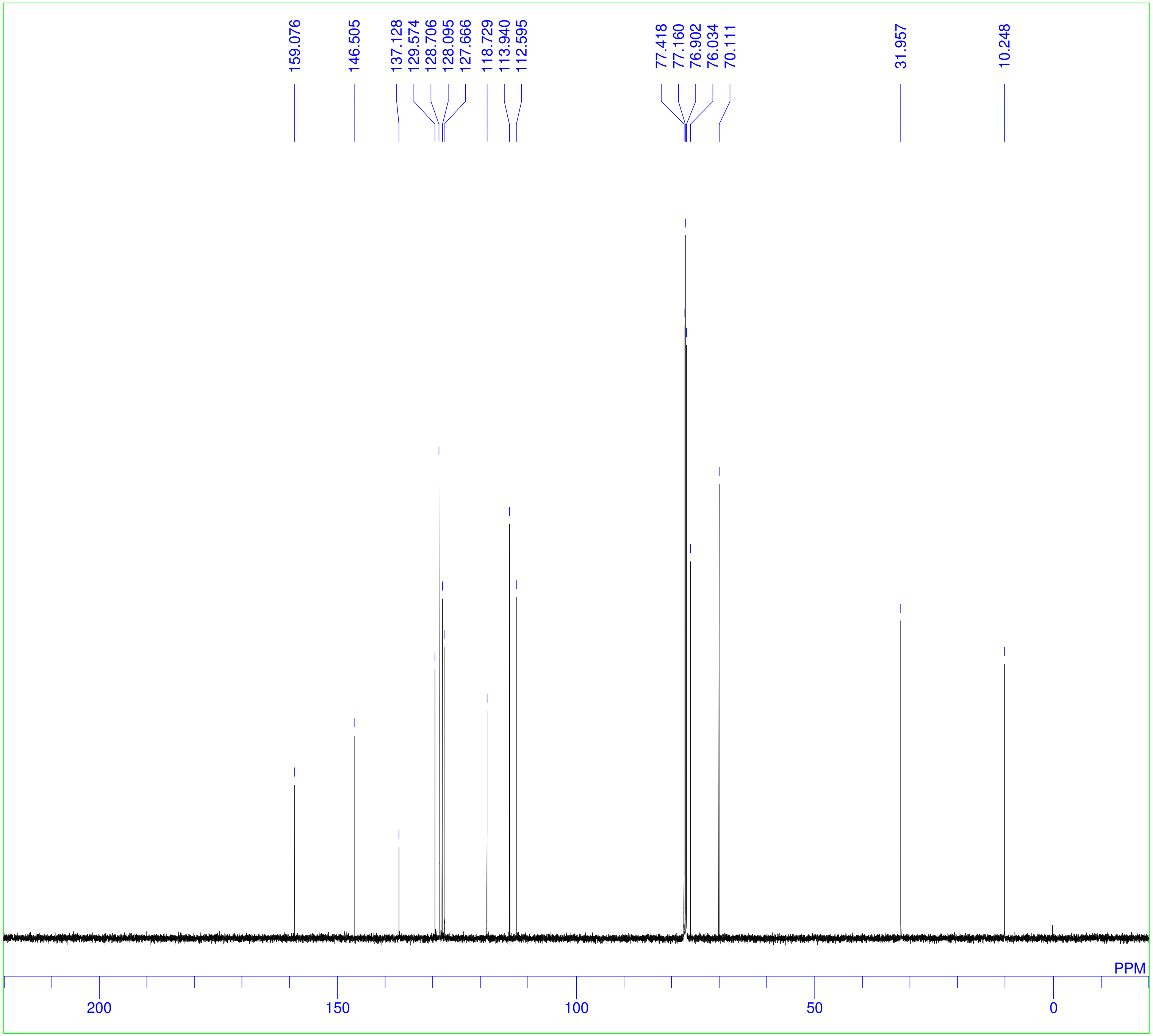
159.076
146.505
137.128
129.574
128.706
128.095
127.666
118.729
113.940
112.595
77.418
77.160
76.902
76.034
70.111
31.957
10.248
PPM
200
150
100
50
0
DFILE S2_Carbon.als
COMNT
DATIM 2024-05-07 19:25:28
OBNUC 13C
EXMOD carbon.jxp
OBFRQ 125.77 MHz
OBSET 7.87 KHz
OBFIN 4.21 Hz
POINT 26214
FREQU 31446.54 Hz
SCANS 1024
ACQTM 0.8336 sec
PD 2.0000 sec
PW1 4.30 usec
IRNUC 1H
CTEMP 23.8 c
SLVNT CDCL3
EXREF 77.16 ppm
BF 0.30 Hz
RGAIN 34

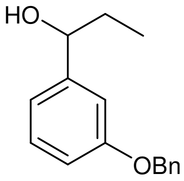
HO
OBn

S2

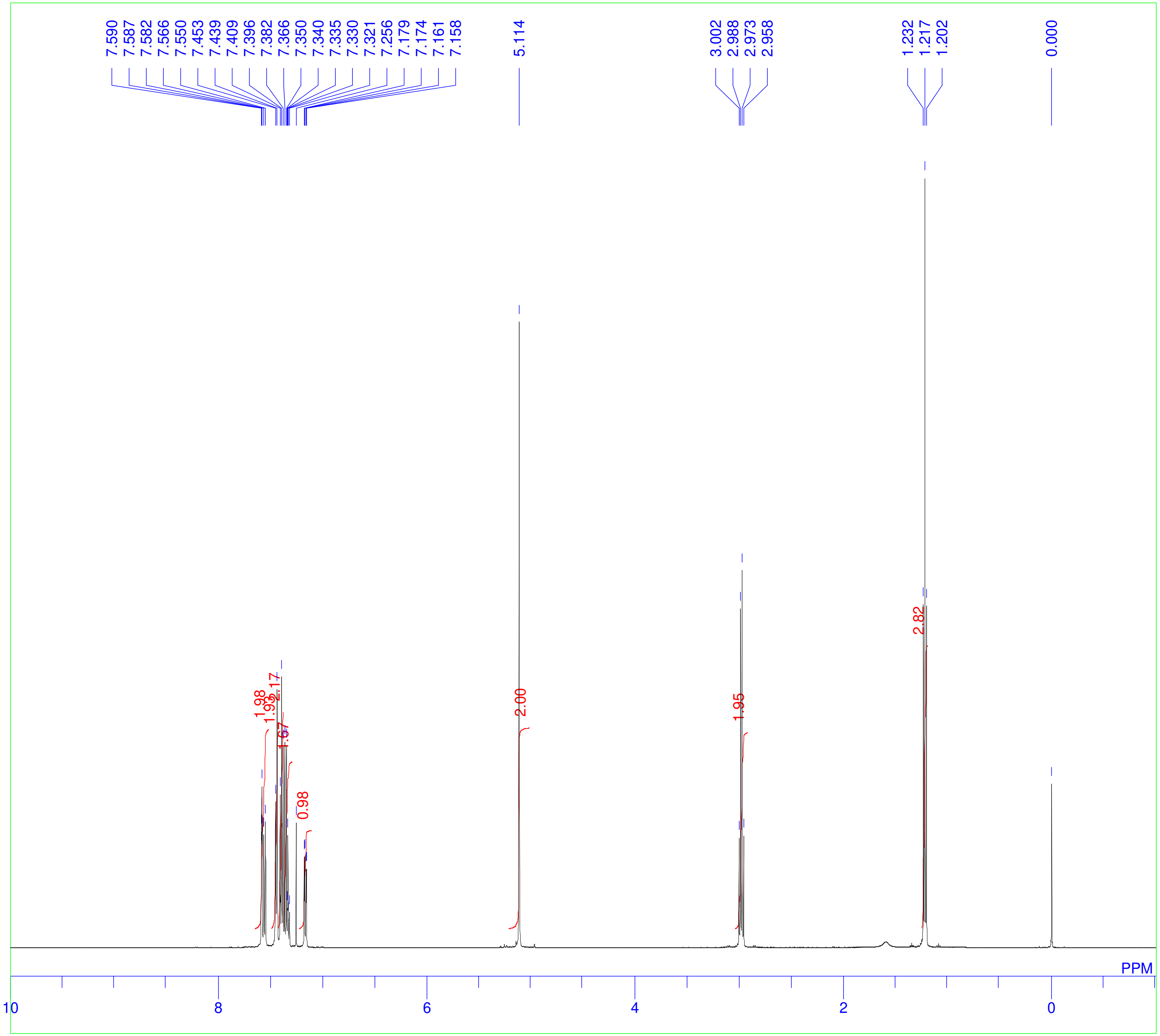

DFILE	S3_Proton.als
COMNT	
DATIM	2024-04-04 14:23:07
OBNUC	1H
EXMOD	proton.jxp
OBFRQ	500.16 MHz
OBSET	2.41 KHz
OBFIN	6.01 Hz
POINT	13107
FREQU	7507.51 Hz
SCANS	8
ACQTM	1.7459 sec
PD	5.0000 sec
PW1	3.80 usec
IRNUC	1H
CTEMP	23.5 c
SLVNT	CDCL3
EXREF	0.00 ppm
BF	0.30 Hz
RGAIN	34

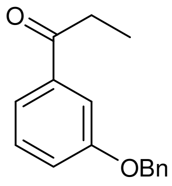

S3

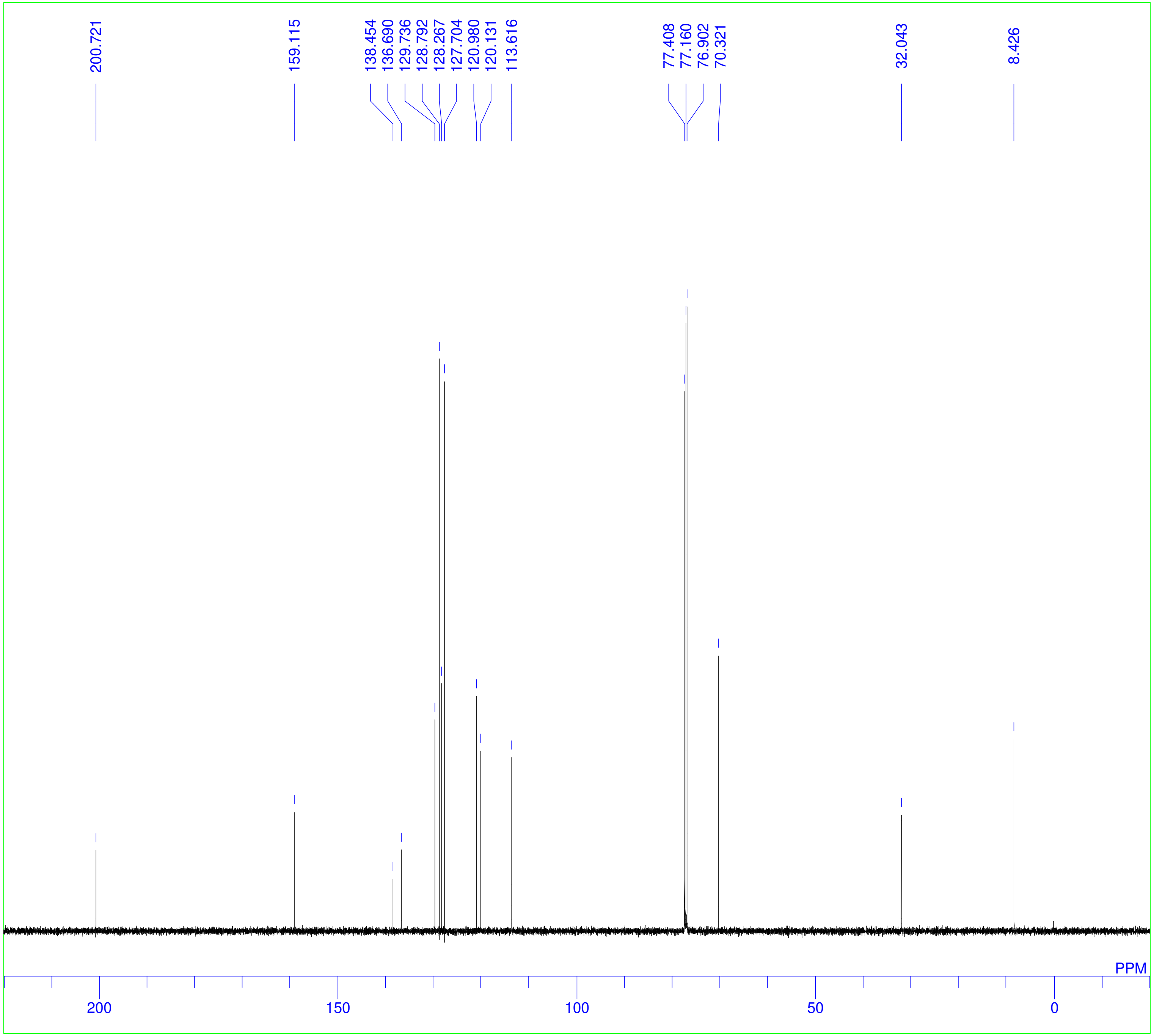

DFILE	S3_Carbon.als
COMNT	
DATIM	2024-04-04 14:24:45
OBNUC	13C
EXMOD	carbon.jxp
OBFRQ	125.77 MHz
OBSET	7.87 KHz
OBFIN	4.21 Hz
POINT	26214
FREQU	31446.54 Hz
SCANS	1024
ACQTM	0.8336 sec
PD	2.0000 sec
PW1	4.30 usec
IRNUC	1H
CTEMP	23.7 c
SLVNT	CDCL3
EXREF	77.16 ppm
BF	0.30 Hz
RGAIN	30

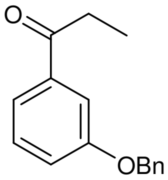

S3

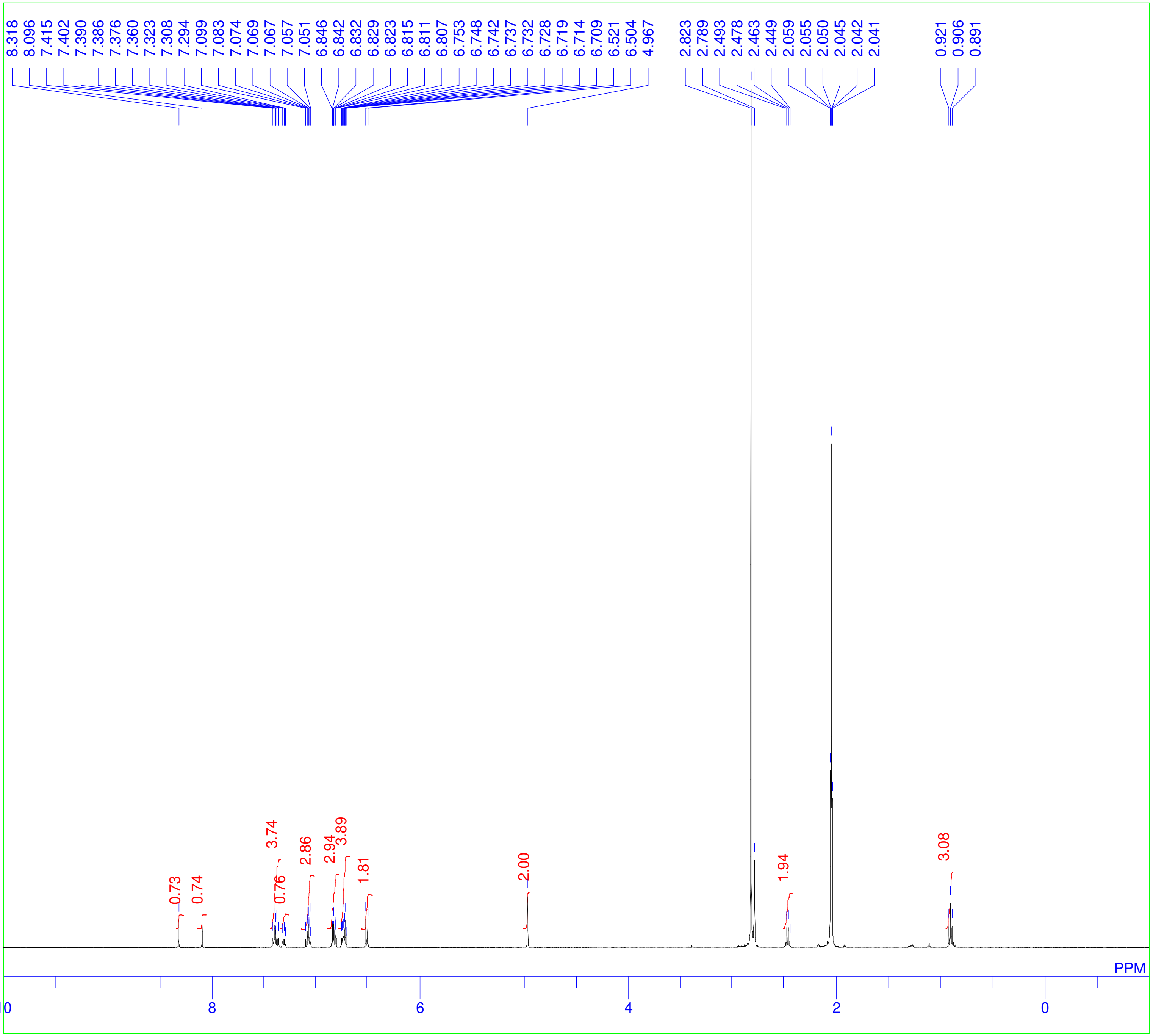

DFILE	RID-OBn_Proton.als
COMNT	
DATIM	2024-05-13 20:33:32
OBNUC	1H
EXMOD	proton.jxp
OBFRQ	500.16 MHz
OBSET	2.41 KHz
OBFIN	6.01 Hz
POINT	13107
FREQU	7507.51 Hz
SCANS	8
ACQTM	1.7459 sec
PD	5.0000 sec
PW1	3.80 usec
IRNUC	1H
CTEMP	23.5 c
SLVNT	ACETN
EXREF	2.05 ppm
BF	0.30 Hz
RGAIN	48

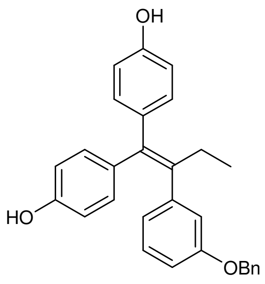

RID-OBn

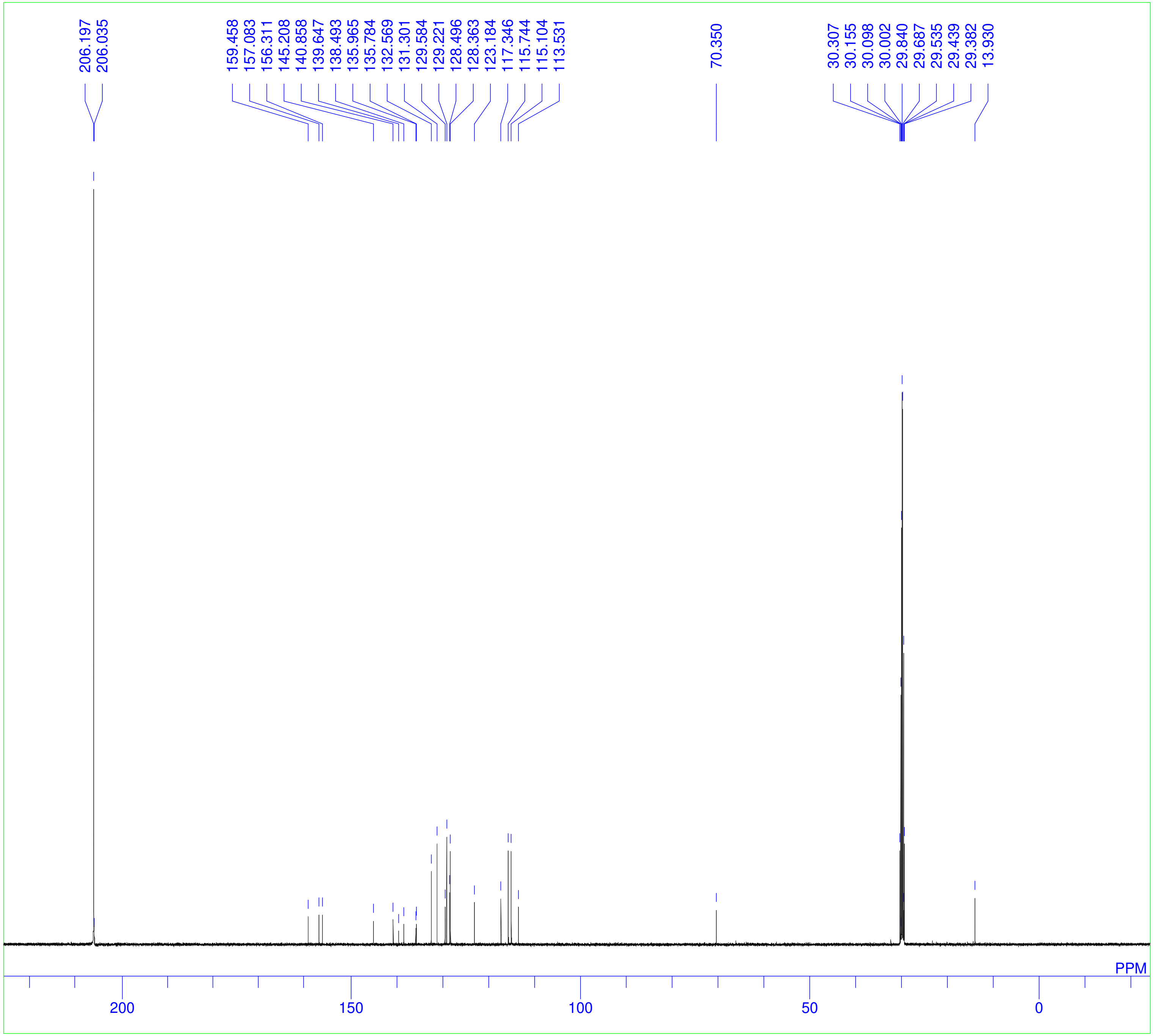

DFILE	RID-OBn_Carbon.als
COMNT	
DATIM	2024-05-09 18:04:25
OBNUC	13C
EXMOD	carbon.jxp
OBFRQ	125.77 MHz
OBSET	7.87 KHz
OBFIN	4.21 Hz
POINT	26214
FREQU	31446.54 Hz
SCANS	1024
ACQTM	0.8336 sec
PD	2.0000 sec
PW1	4.30 usec
IRNUC	1H
CTEMP	24.0 c
SLVNT	ACETN
EXREF	29.84 ppm
BF	0.30 Hz
RGAIN	30

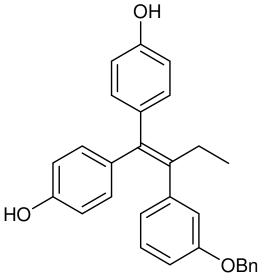

RID-OBn

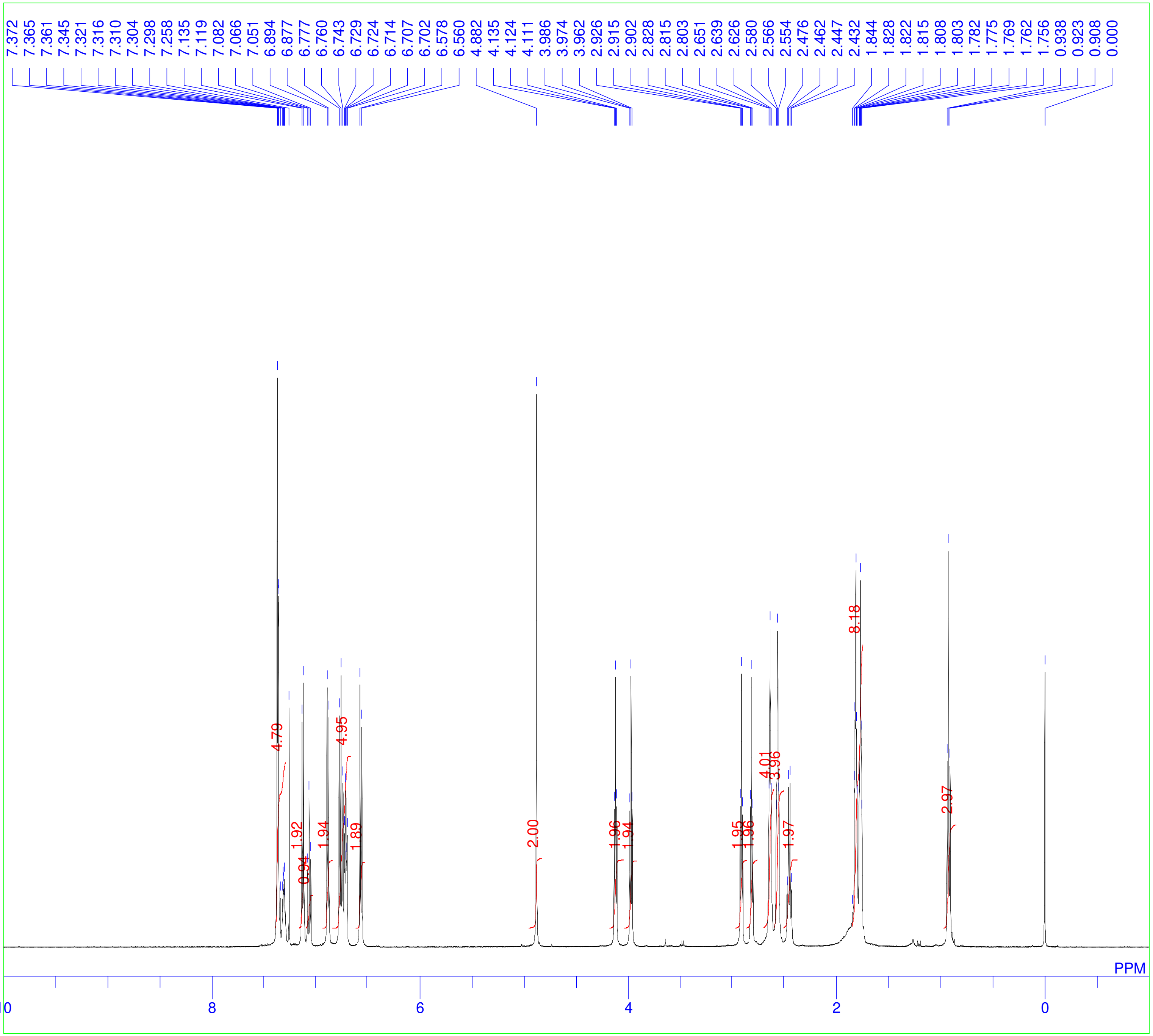

DFILE	RID-B-OBn_Proton.als
COMNT	
DATIM	2024-05-08 10:35:01
OBNUC	1H
EXMOD	proton.jxp
OBFRQ	500.16 MHz
OBSET	2.41 KHz
OBFIN	6.01 Hz
POINT	13107
FREQU	7507.51 Hz
SCANS	8
ACQTM	1.7459 sec
PD	5.0000 sec
PW1	3.80 usec
IRNUC	1H
CTEMP	23.7 c
SLVNT	CDCL3
EXREF	0.00 ppm
BF	0.30 Hz
RGAIN	36

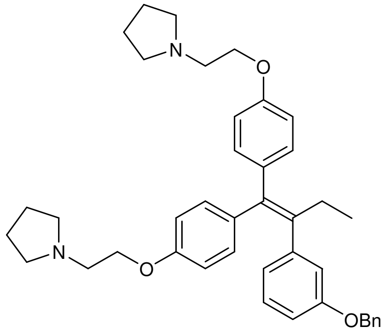

RID-B-OBn

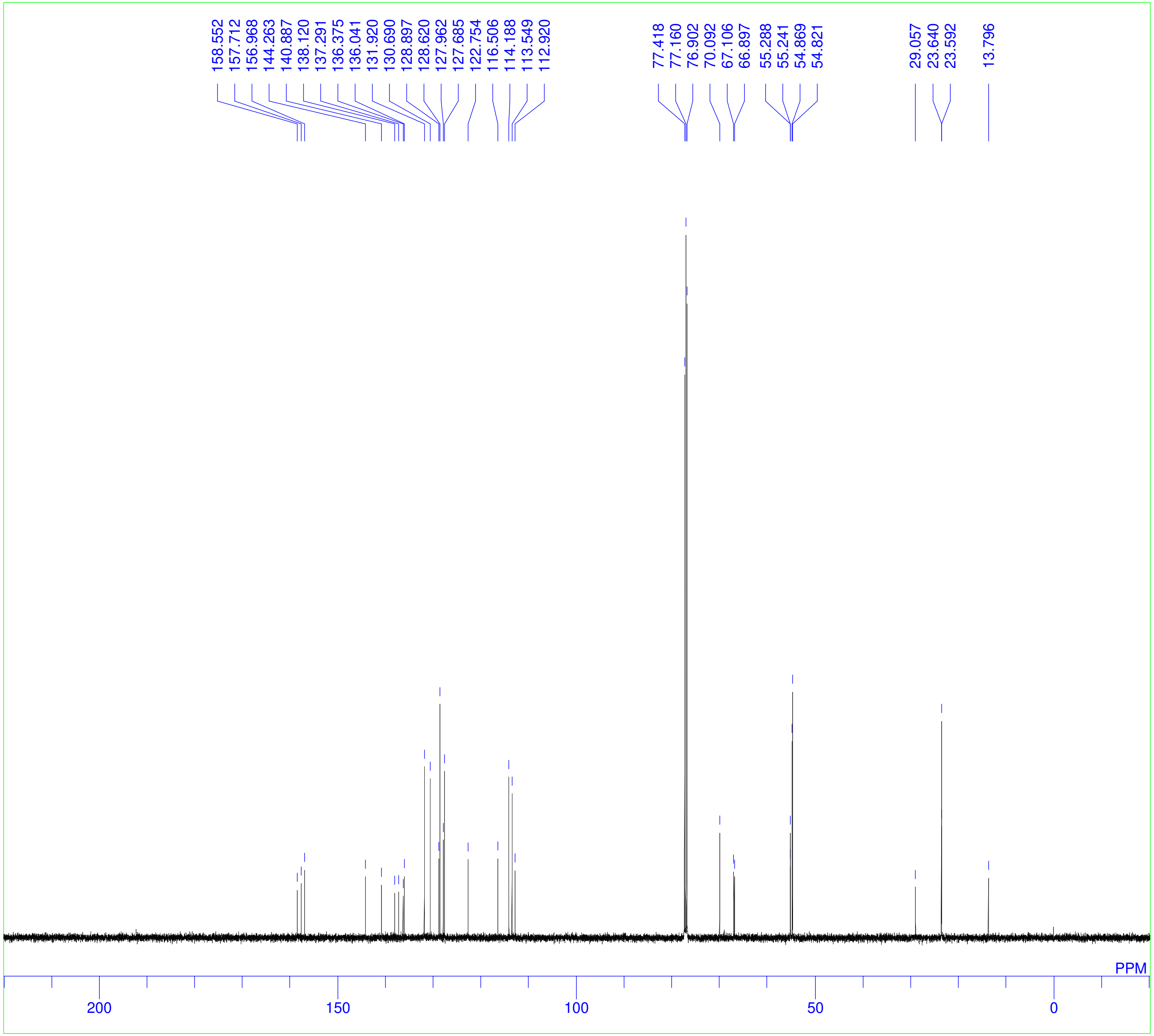

DFILE	RID-B-OBn_Carbon.als
COMNT	
DATIM	2024-05-08 13:58:14
OBNUC	13C
EXMOD	carbon.jxp
OBFRQ	125.77 MHz
OBSET	7.87 KHz
OBFIN	4.21 Hz
POINT	26214
FREQU	31446.54 Hz
SCANS	1024
ACQTM	0.8336 sec
PD	2.0000 sec
PW1	4.30 usec
IRNUC	1H
CTEMP	23.9 c
SLVNT	CDCL3
EXREF	77.16 ppm
BF	0.30 Hz
RGAIN	30

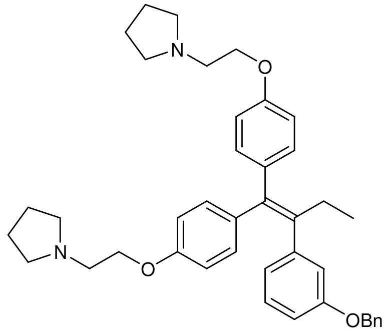

RID-B-OBn

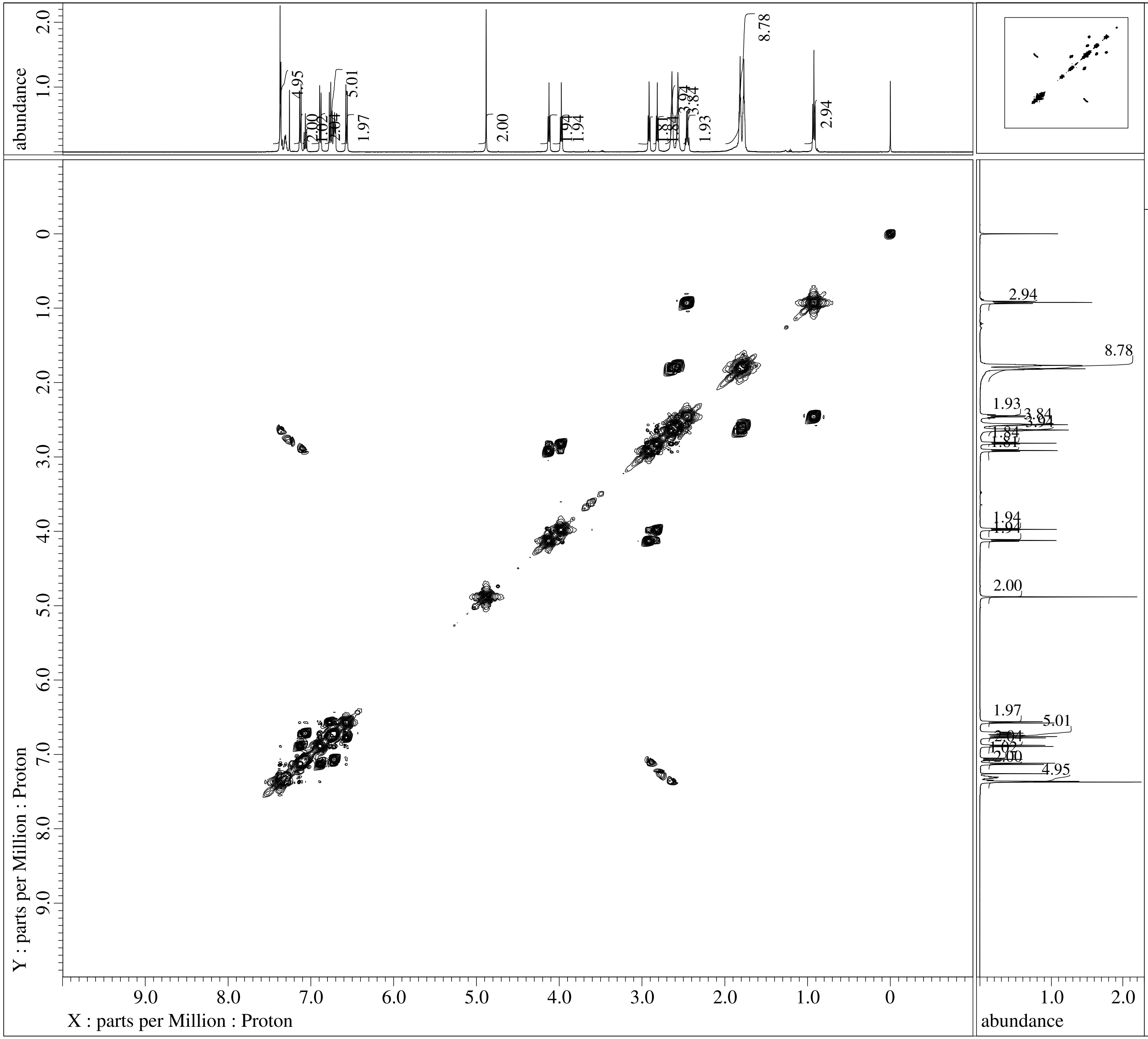


```
---- PROCESSING PARAMETERS ----
dc_balance( 0, FALSE )
sexp( 0.2[Hz], 0.0[s] )
trapezoid( 0[%], 0[%], 80[%], 100[%] )
zerofill( 1 )
fft( 1, TRUE, TRUE )
machinephase
ppm

Filename          = RID-B-OBn_COSY-1-4.jdf
Author            = delta
Experiment        = cosy.jxp
Sample_Id         = EM-RID-B-OBn
Solvent           = CHLOROFORM-D
Creation_Time     = 25-MAY-2024 12:29:44
Revision_Time     = 17-JUN-2024 19:53:08
Current_Time      = 17-JUN-2024 19:53:48

Comment           = gradient absolute value co
Data_Format       = 2D REAL REAL
Dim_Size          = 1024, 1024
Dim_Title         = Proton Proton
Dim_Units         = [ppm] [ppm]
Dimensions        = X Y
Site              = JNM-ECA500II
Spectrometer      = DELTA2_NMR

Field_Strength    = 11.7473579[T] (500[MHz])
X_Acq_Duration    = 0.1363968[s]
X_Domain          = 1H
X_Freq            = 500.15991521[MHz]
X_Offset          = 5.0[ppm]
X_Points          = 1280
X_Prescans        = 4
X_Resolution      = 7.3315503[Hz]
X_Sweep           = 9.38438438[kHz]
X_Sweep_Clipped   = 7.50750751[kHz]
Y_Domain          = 1H
Y_Freq            = 500.15991521[MHz]
Y_Offset          = 5.0[ppm]
Y_Points          = 256
Y_Prescans        = 0
Y_Resolution      = 29.31299715[Hz]
Y_Sweep           = 7.50412727[kHz]
Irr_Domain        = Proton
Irr_Freq          = 500.15991521[MHz]
Irr_Offset        = 5.0[ppm]
Tri_Domain        = Proton
Tri_Freq          = 500.15991521[MHz]
Tri_Offset        = 5.0[ppm]
Clipped           = FALSE
Scans             = 1
Total_Scans       = 256

Relaxation_Delay  = 1.5[s]
Recvr_Gain        = 38
Temp_Get          = 24.1[dC]
X_90_Width        = 7.59[us]
```

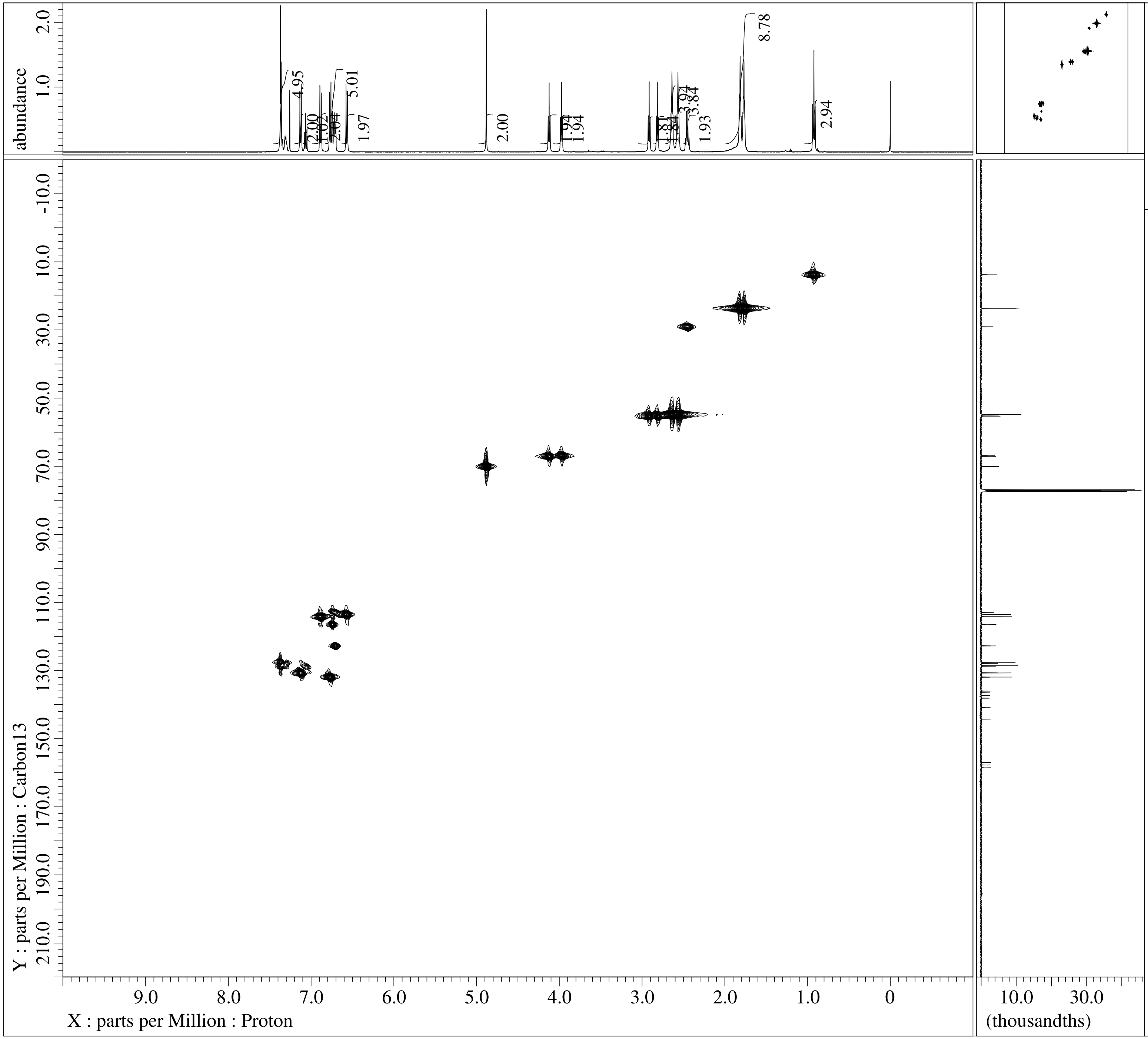

---- PROCESSING PARAMETERS ----
dc_balance(0, FALSE)
sexp(2.0[Hz], 0.0[s])
trapezoid(0[%], 0[%], 80[%], 100[%])
zerofill(1, TRUE)
fft(1, TRUE, TRUE)
machinephase
ppm

Filename	= RID-B-OBn_HMQC-1-4.jdf
Author	= delta
Experiment	= hmqc.jxp
Sample_Id	= EM-RID-B-OBn
Solvent	= CHLOROFORM-D
Creation_Time	= 24-MAY-2024 13:51:21
Revision_Time	= 17-JUN-2024 19:47:52
Current_Time	= 17-JUN-2024 19:48:46
Comment	= gradient enhanced HMQC
Data_Format	= 2D REAL REAL
Dim_Size	= 819, 512
Dim_Title	= Proton Carbon13
Dim_Units	= [ppm] [ppm]
Dimensions	= X Y
Site	= JNM-ECA500II
Spectrometer	= DELTA2_NMR
Field_Strength	= 11.7473579[T] (500[MHz]
X_Acq_Duration	= 0.10911744[s]
X_Domain	= 1H
X_Freq	= 500.15991521[MHz]
X_Offset	= 5.0[ppm]
X_Points	= 1024
X_Prescans	= 4
X_Resolution	= 9.16443788[Hz]
X_Sweep	= 9.38438438[kHz]
X_Sweep_Clipped	= 7.50750751[kHz]
Y_Domain	= 13C
Y_Freq	= 125.76529768[MHz]
Y_Offset	= 85[ppm]
Y_Points	= 256
Y_Prescans	= 0
Y_Resolution	= 83.60980308[Hz]
Y_Sweep	= 21.40410959[kHz]
Tri_Domain	= Proton
Tri_Freq	= 500.15991521[MHz]
Tri_Offset	= 5.0[ppm]
Clipped	= FALSE
Scans	= 4
Total_Scans	= 1024
Relaxation_Delay	= 1.5[s]
Recvr_Gain	= 50
Temp_Get	= 24[dC]
X_Acq_Time	= 0.10911744[s]
X_Atn	= 3.2[dB]
X_Gamma	= 42576375
X_Pulse	= 7.59[us]

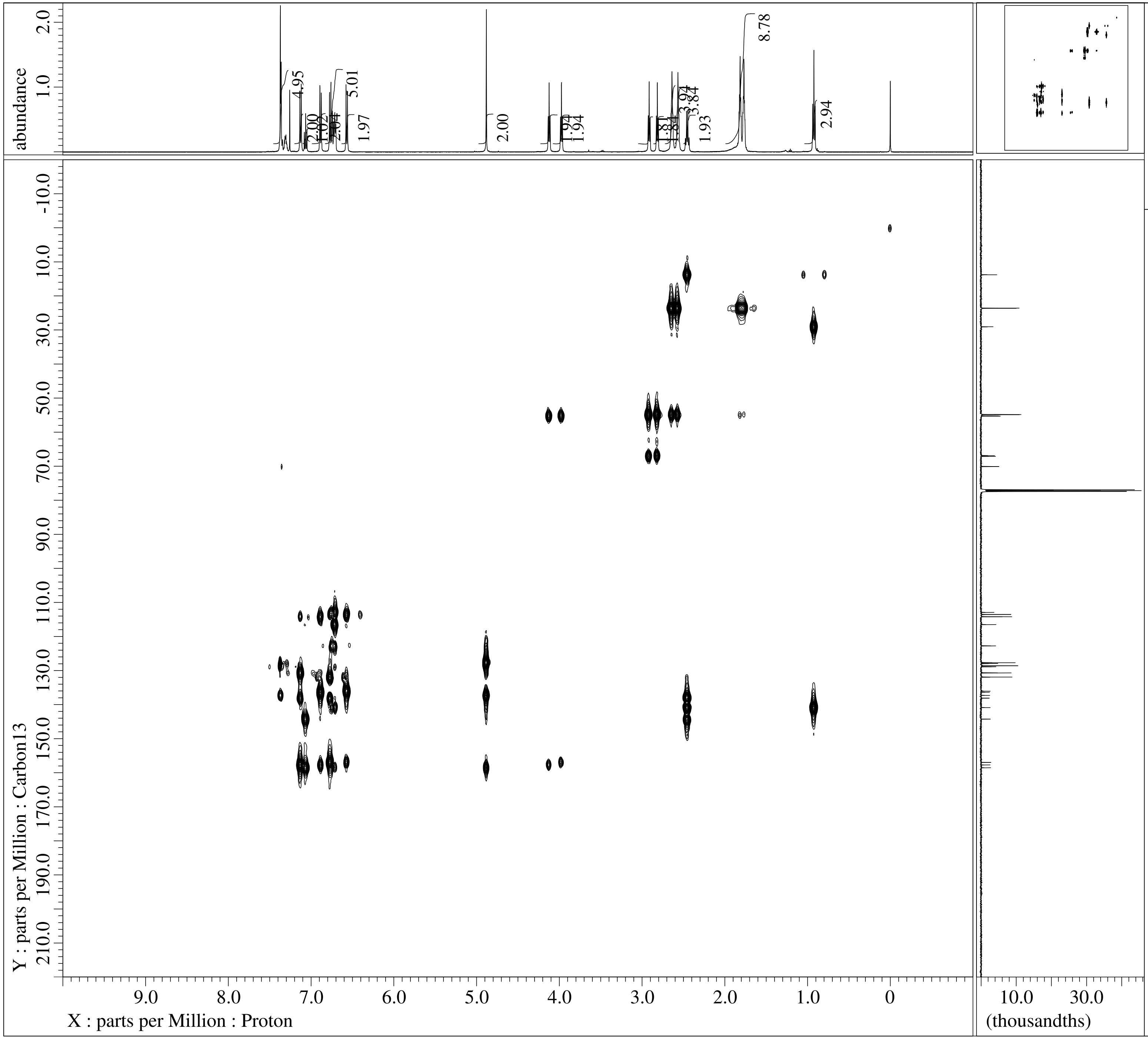


```
---- PROCESSING PARAMETERS ----
dc_balance( 0, FALSE )
sexp( 0.2[Hz], 0.0[s] )
trapezoid( 0[%], 0[%], 80[%], 100[%] )
zerofill( 1 )
fft( 1, TRUE, TRUE )
machinephase
ppm
```

```
Filename            = RID-B-OBn_HMBC-1-6.jdf
Author              = delta
Experiment          = hmbc.jxp
Sample_Id           = EM-RID-B-OBn
Solvent             = CHLOROFORM-D
Creation_Time       = 25-MAY-2024 12:37:26
Revision_Time       = 17-JUN-2024 19:51:01
Current_Time        = 17-JUN-2024 19:51:59

Comment             = gradient enhanced HMBC
Data_Format         = 2D REAL REAL
Dim_Size            = 1638, 512
Dim_Title           = Proton Carbon13
Dim_Units           = [ppm] [ppm]
Dimensions          = X Y
Site                = JNM-ECA500II
Spectrometer        = DELTA2_NMR

Field_Strength      = 11.7473579[T] (500[MHz]
X_Acq_Duration      = 0.21823488[s]
X_Domain            = 1H
X_Freq              = 500.15991521[MHz]
X_Offset            = 5.0[ppm]
X_Points            = 2048
X_Prescans          = 4
X_Resolution        = 4.58221894[Hz]
X_Sweep             = 9.38438438[kHz]
X_Sweep_Clipped     = 7.50750751[kHz]
Y_Domain            = 13C
Y_Freq              = 125.76529768[MHz]
Y_Offset            = 100[ppm]
Y_Points            = 256
Y_Prescans          = 0
Y_Resolution        = 122.99275819[Hz]
Y_Sweep             = 31.4861461[kHz]
Tri_Domain          = Proton
Tri_Freq            = 500.15991521[MHz]
Tri_Offset          = 5.0[ppm]
Clipped             = FALSE
Scans               = 8
Total_Scans         = 2048

Relaxation_Delay    = 1.5[s]
Recvr_Gain          = 50
Temp_Get            = 24.1[dC]
X_Acq_Time          = 0.21823488[s]
X_Atn               = 3.2[dB]
X_Gamma             = 42576375
X_Pulse             = 7.59[us]
```

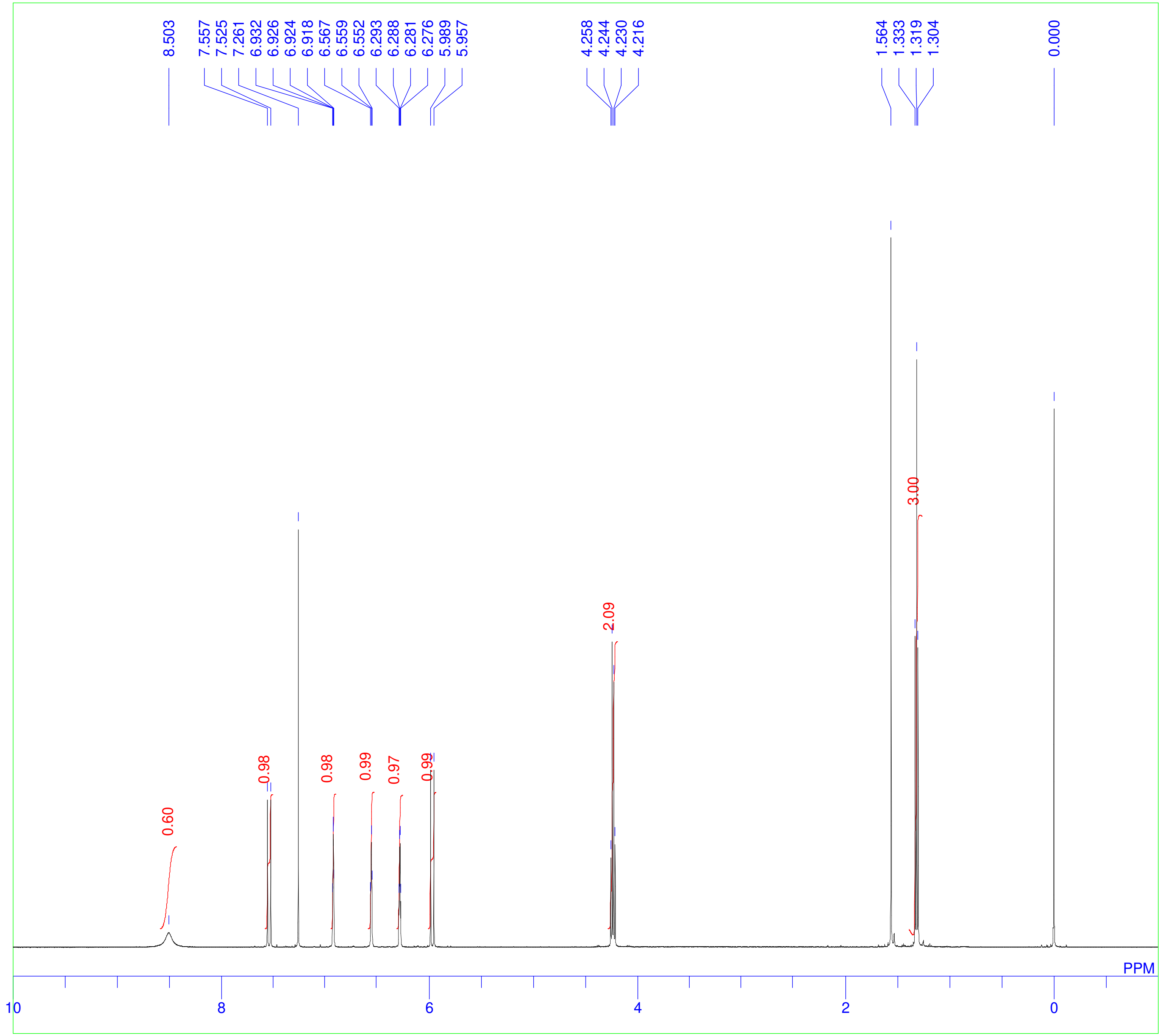

DFILE	E-S4_Proton.als
COMNT	
DATIM	2024-05-13 20:25:40
OBNUC	1H
EXMOD	proton.jxp
OBFRQ	500.16 MHz
OBSET	2.41 KHz
OBFIN	6.01 Hz
POINT	13107
FREQU	7507.51 Hz
SCANS	8
ACQTM	1.7459 sec
PD	5.0000 sec
PW1	3.80 usec
IRNUC	1H
CTEMP	23.5 c
SLVNT	CDCL3
EXREF	0.00 ppm
BF	0.30 Hz
RGAIN	46

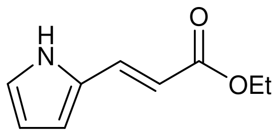

(*E*)-S4

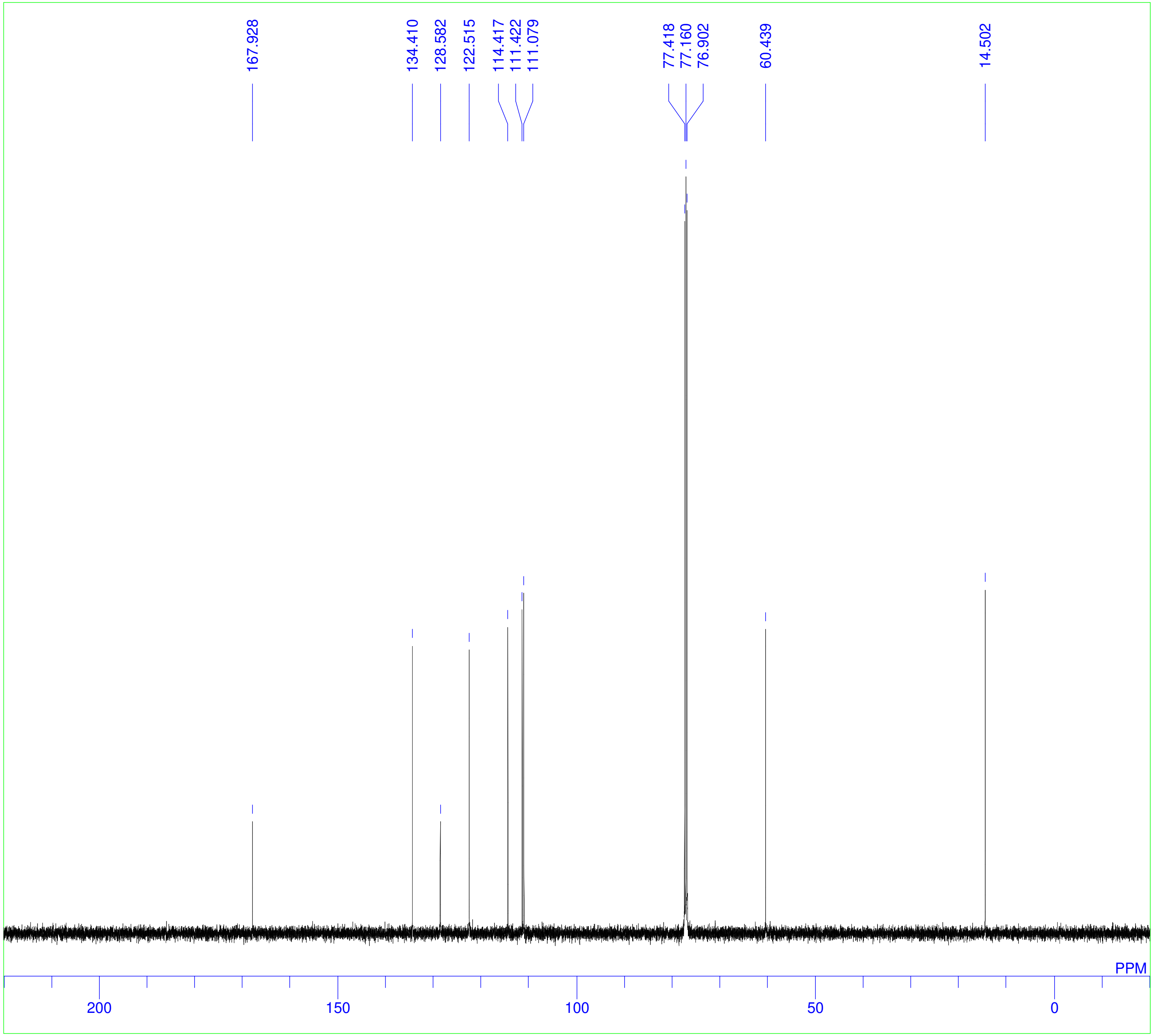

DFILE E-S4_Carbon.als
COMNT
DATIM 2024-05-08 20:12:53
OBNUC 13C
EXMOD carbon.jxp
OBFRQ 125.77 MHz
OBSET 7.87 KHz
OBFIN 4.21 Hz
POINT 26214
FREQU 31446.54 Hz
SCANS 1024
ACQTM 0.8336 sec
PD 2.0000 sec
PW1 4.30 usec
IRNUC 1H
CTEMP 24.1 c
SLVNT CDCL3
EXREF 77.16 ppm
BF 0.30 Hz
RGAIN 30

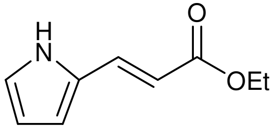

(*E*)-S4

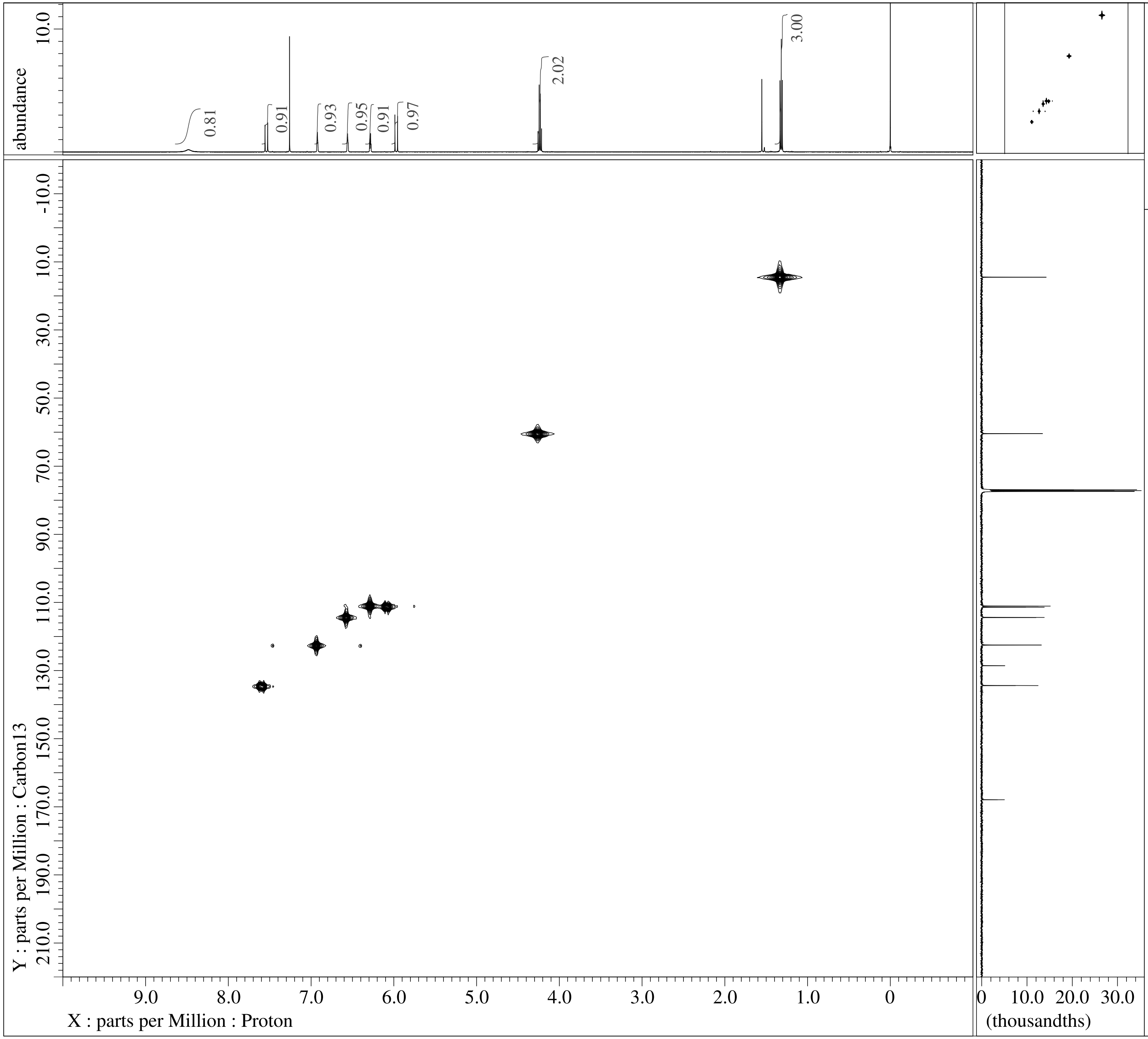


```
---- PROCESSING PARAMETERS ----
dc_balance( 0, FALSE )
sexp( 2.0[Hz], 0.0[s] )
trapezoid( 0[%], 0[%], 80[%], 100[%] )
zerofill( 1, TRUE )
fft( 1, TRUE, TRUE )
machinephase
ppm

Filename            = E-S4_HMQC-1-3.jdf
Author              = delta
Experiment          = hmqc.jxp
Sample_Id           = 1-E_BODIPY
Solvent             = CHLOROFORM-D
Creation_Time       = 19-MAY-2024 18:24:38
Revision_Time       = 17-JUN-2024 18:07:32
Current_Time        = 17-JUN-2024 18:08:30

Comment             = gradient enhanced HMQC
Data_Format         = 2D REAL REAL
Dim_Size            = 819, 512
Dim_Title           = Proton Carbon13
Dim_Units           = [ppm] [ppm]
Dimensions          = X Y
Site                = JNM-ECA500II
Spectrometer        = DELTA2_NMR

Field_Strength      = 11.7473579[T] (500[MHz]
X_Acq_Duration      = 0.10911744[s]
X_Domain            = 1H
X_Freq              = 500.15991521[MHz]
X_Offset            = 5.0[ppm]
X_Points            = 1024
X_Prescans          = 4
X_Resolution        = 9.16443788[Hz]
X_Sweep             = 9.38438438[kHz]
X_Sweep_Clipped     = 7.50750751[kHz]
Y_Domain            = 13C
Y_Freq              = 125.76529768[MHz]
Y_Offset            = 85[ppm]
Y_Points            = 256
Y_Prescans          = 0
Y_Resolution        = 83.60980308[Hz]
Y_Sweep             = 21.40410959[kHz]
Tri_Domain          = Proton
Tri_Freq            = 500.15991521[MHz]
Tri_Offset          = 5.0[ppm]
Clipped             = FALSE
Scans               = 4
Total_Scans         = 1024

Relaxation_Delay    = 1.5[s]
Recvr_Gain          = 50
Temp_Get            = 23.9[dC]
X_Acq_Time          = 0.10911744[s]
X_Atn               = 3.2[dB]
X_Gamma             = 42576375
X_Pulse             = 7.59[us]
```

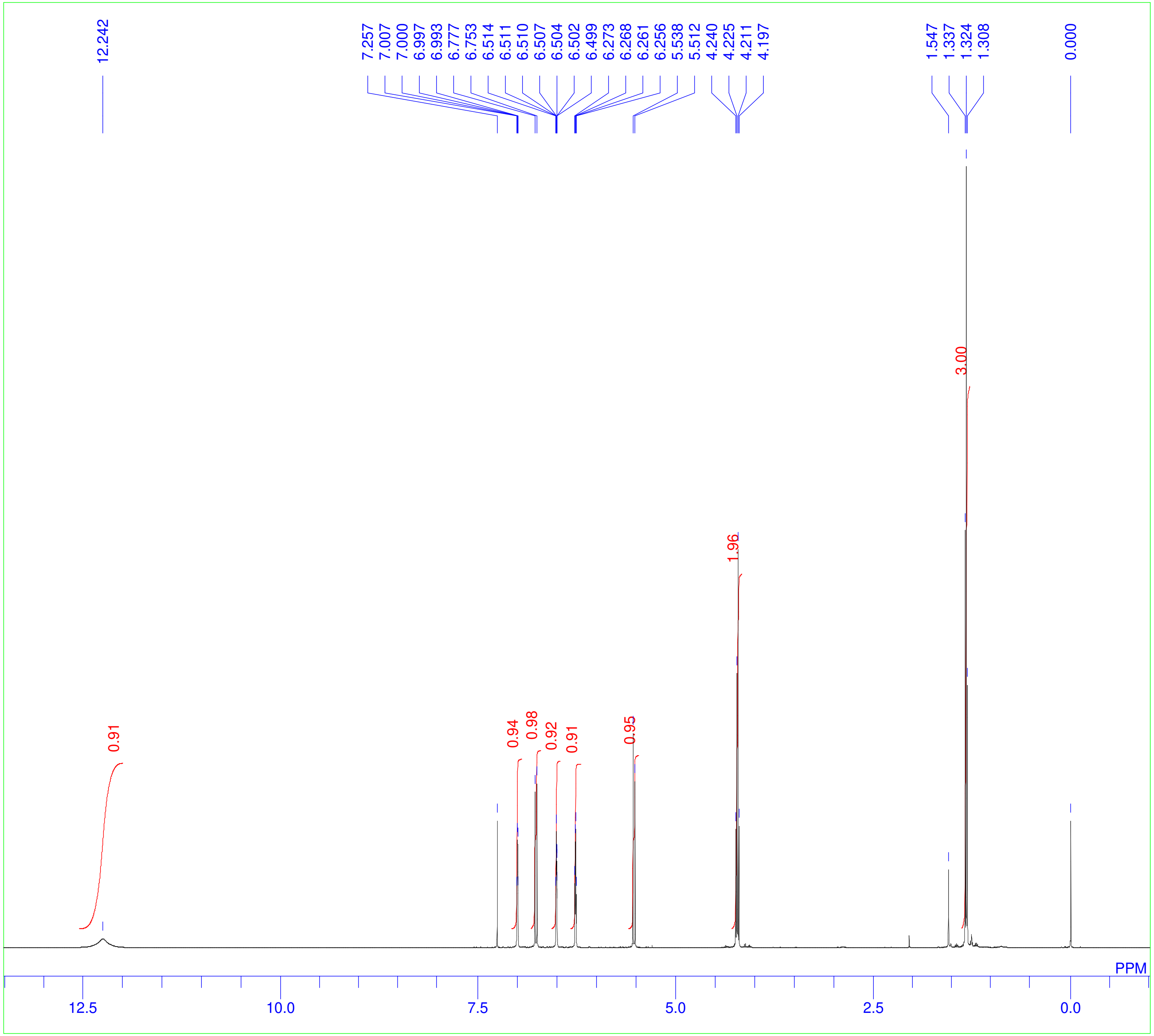

DFILE	Z-S4_Proton.als
COMNT	
DATIM	2024-05-21 13:47:47
OBNUC	1H
EXMOD	proton.jxp
OBFRQ	500.16 MHz
OBSET	2.41 KHz
OBFIN	6.01 Hz
POINT	13107
FREQU	10020.04 Hz
SCANS	8
ACQTM	1.3081 sec
PD	5.0000 sec
PW1	3.80 usec
IRNUC	1H
CTEMP	23.8 c
SLVNT	CDCL3
EXREF	0.00 ppm
BF	0.30 Hz
RGAIN	42

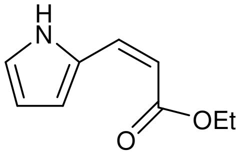

(*Z*)-S4

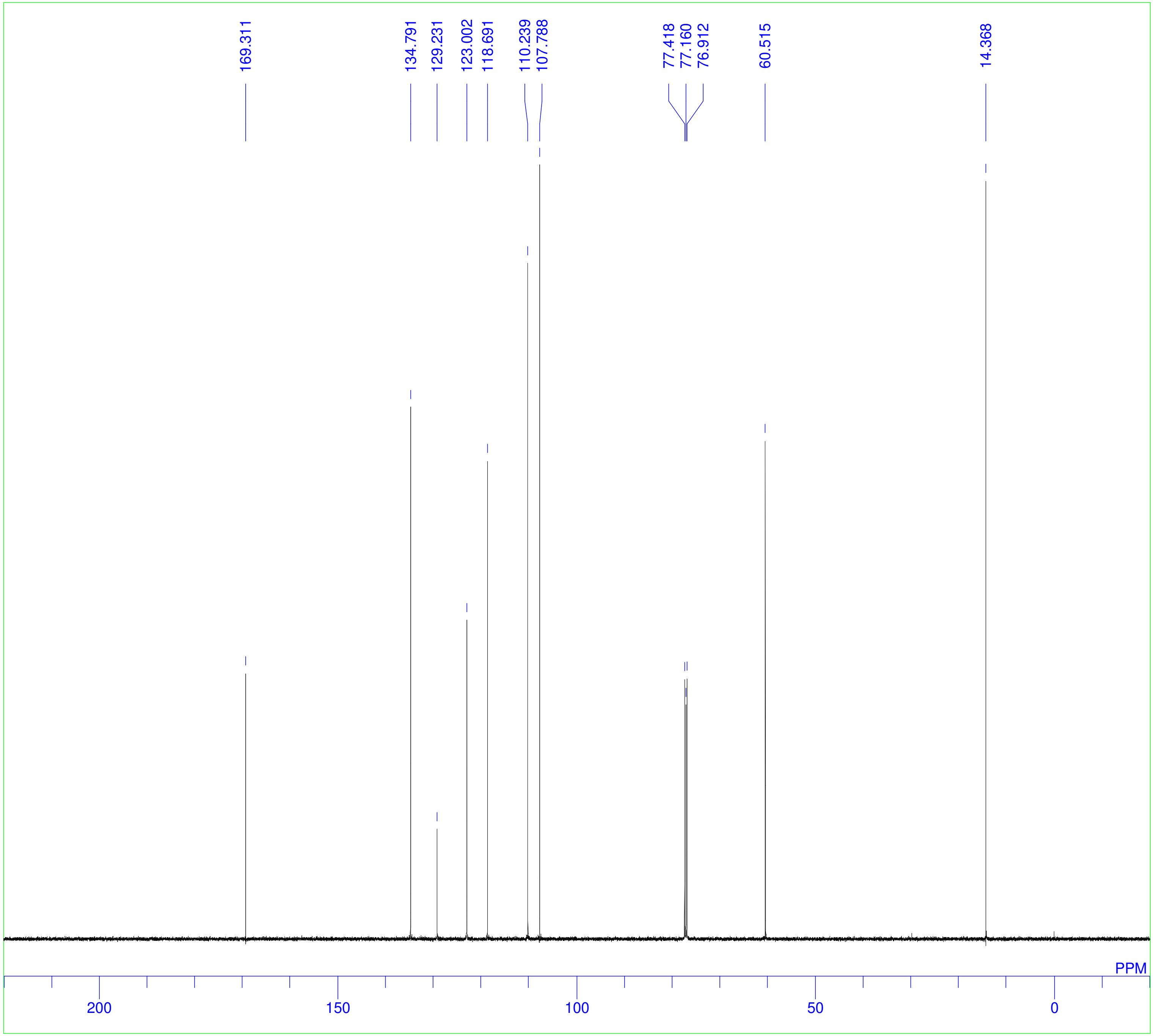

DFILE	Z-S4_Carbon.als
COMNT	
DATIM	2024-05-17 12:48:49
OBNUC	13C
EXMOD	carbon.jxp
OBFRQ	125.77 MHz
OBSET	7.87 KHz
OBFIN	4.21 Hz
POINT	26214
FREQU	31446.54 Hz
SCANS	1024
ACQTM	0.8336 sec
PD	2.0000 sec
PW1	4.30 usec
IRNUC	1H
CTEMP	24.4 c
SLVNT	CDCL3
EXREF	77.16 ppm
BF	0.30 Hz
RGAIN	36

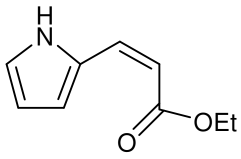

(*Z*)-S4

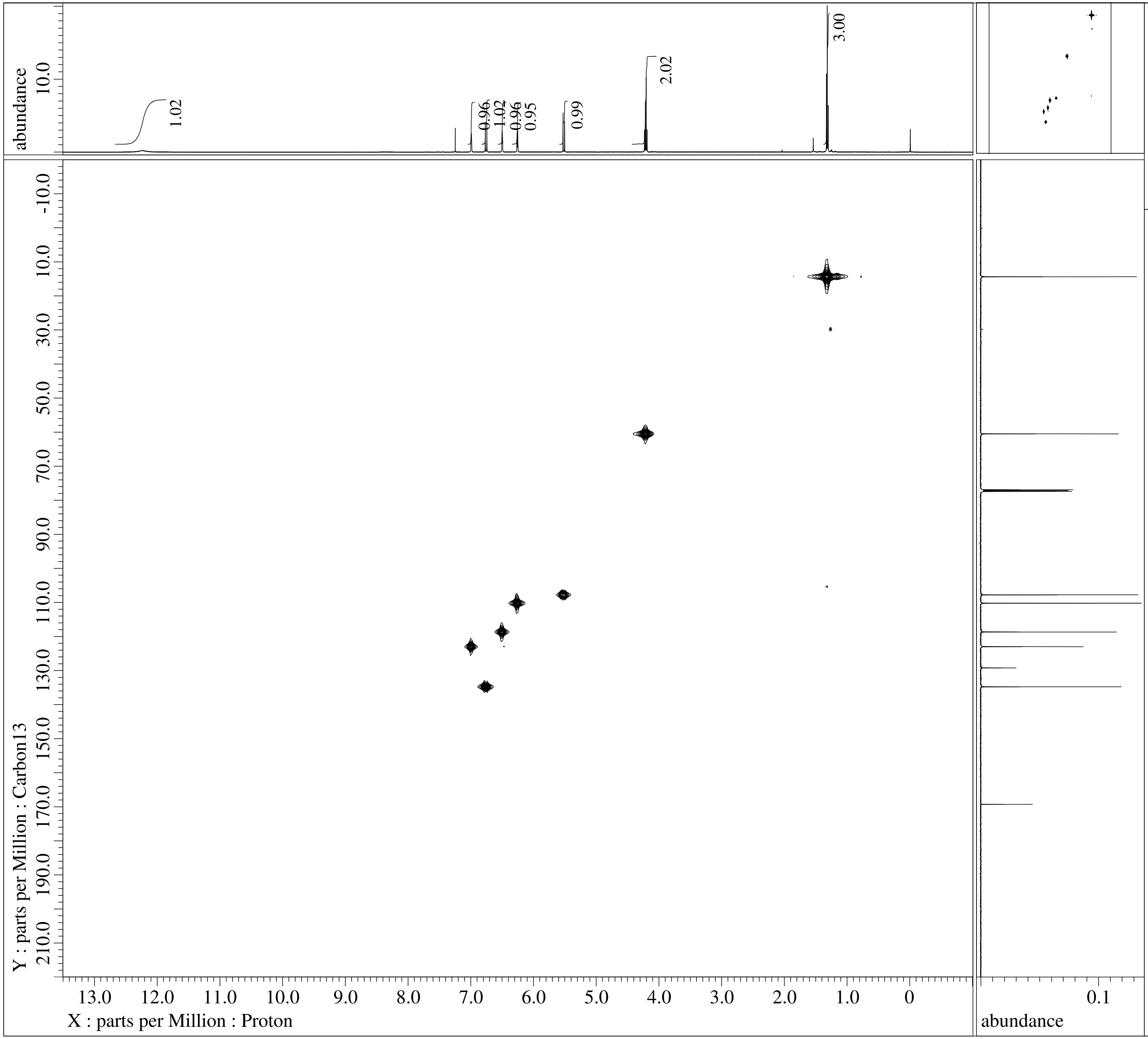


```
---- PROCESSING PARAMETERS ----
sinbell4( -60, 160 )
zerofill( 1 )
fft( 1, TRUE, TRUE )
ppm
[transpose]
sinbell4( -60, 160 )
zerofill( 2 )
fft( 1, TRUE, TRUE )
ppm
abs
```

```
Filename            = Z-S4_HMQC-1-4.jdf
Author              = delta
Experiment          = hmqc.jxp
Sample_Id           = 1-Z_BODIPY 1
Solvent             = CHLOROFORM-D
Creation_Time       = 21-MAY-2024 13:54:25
Revision_Time       = 17-JUN-2024 18:16:59
Current_Time        = 17-JUN-2024 18:17:51

Comment             = gradient enhanced HMQC
Data_Format         = 2D REAL REAL
Dim_Size            = 819, 512
Dim_Title           = Proton Carbon13
Dim_Units           = [ppm] [ppm]
Dimensions          = X Y
Site                = JNM-ECA500II
Spectrometer        = DELTA2_NMR

Field_Strength      = 11.7473579[T] (500[MHz]
X_Acq_Duration      = 81.83808[ms]
X_Domain            = 1H
X_Freq              = 500.15991521[MHz]
X_Offset            = 5.0[ppm]
X_Points            = 1024
X_Prescans          = 4
X_Resolution        = 12.2192505[Hz]
X_Sweep             = 12.51251251[kHz]
X_Sweep_Clipped     = 10.01001001[kHz]
Y_Domain            = 13C
Y_Freq              = 125.76529768[MHz]
Y_Offset            = 85[ppm]
Y_Points            = 256
Y_Prescans          = 0
Y_Resolution        = 83.60980308[Hz]
Y_Sweep             = 21.40410959[kHz]
Tri_Domain          = Proton
Tri_Freq            = 500.15991521[MHz]
Tri_Offset          = 5.0[ppm]
Clipped             = FALSE
Scans               = 4
Total_Scans         = 1024

Relaxation_Delay    = 1.5[s]
Recvr_Gain          = 50
Temp_Get            = 23.9[dC]
X_Acq_Time          = 81.83808[ms]
X_Atn               = 3.2[dB]
X_Gamma             = 42576375
X_Pulse             = 7.59[us]
```

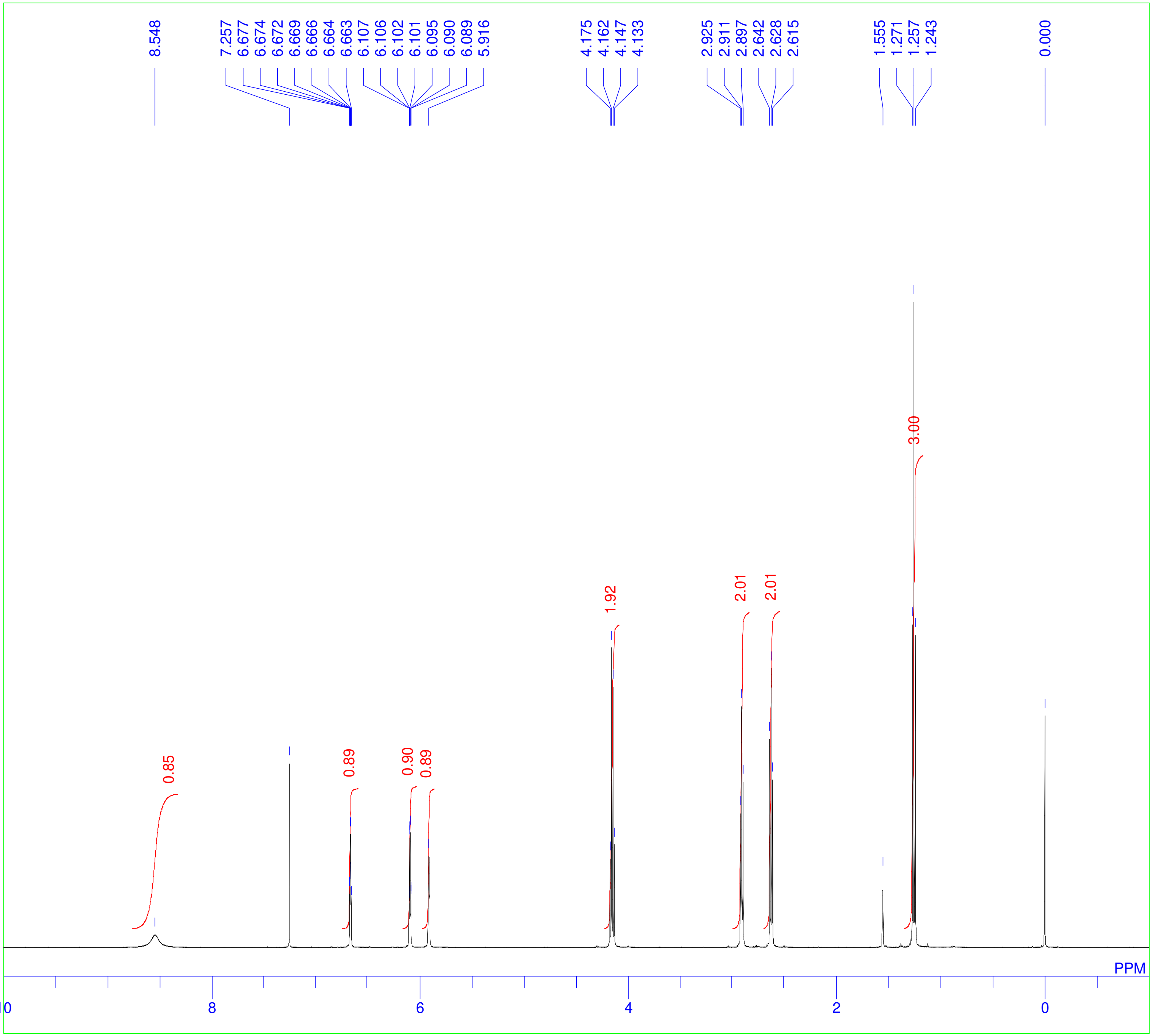

DFILE	S5_Proton.als
COMNT	
DATIM	2024-04-23 21:26:11
OBNUC	1H
EXMOD	proton.jxp
OBFRQ	500.16 MHz
OBSET	2.41 KHz
OBFIN	6.01 Hz
POINT	13107
FREQU	7507.51 Hz
SCANS	8
ACQTM	1.7459 sec
PD	5.0000 sec
PW1	3.80 usec
IRNUC	1H
CTEMP	24.0 c
SLVNT	CDCL3
EXREF	0.00 ppm
BF	0.30 Hz
RGAIN	40

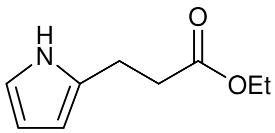

S5

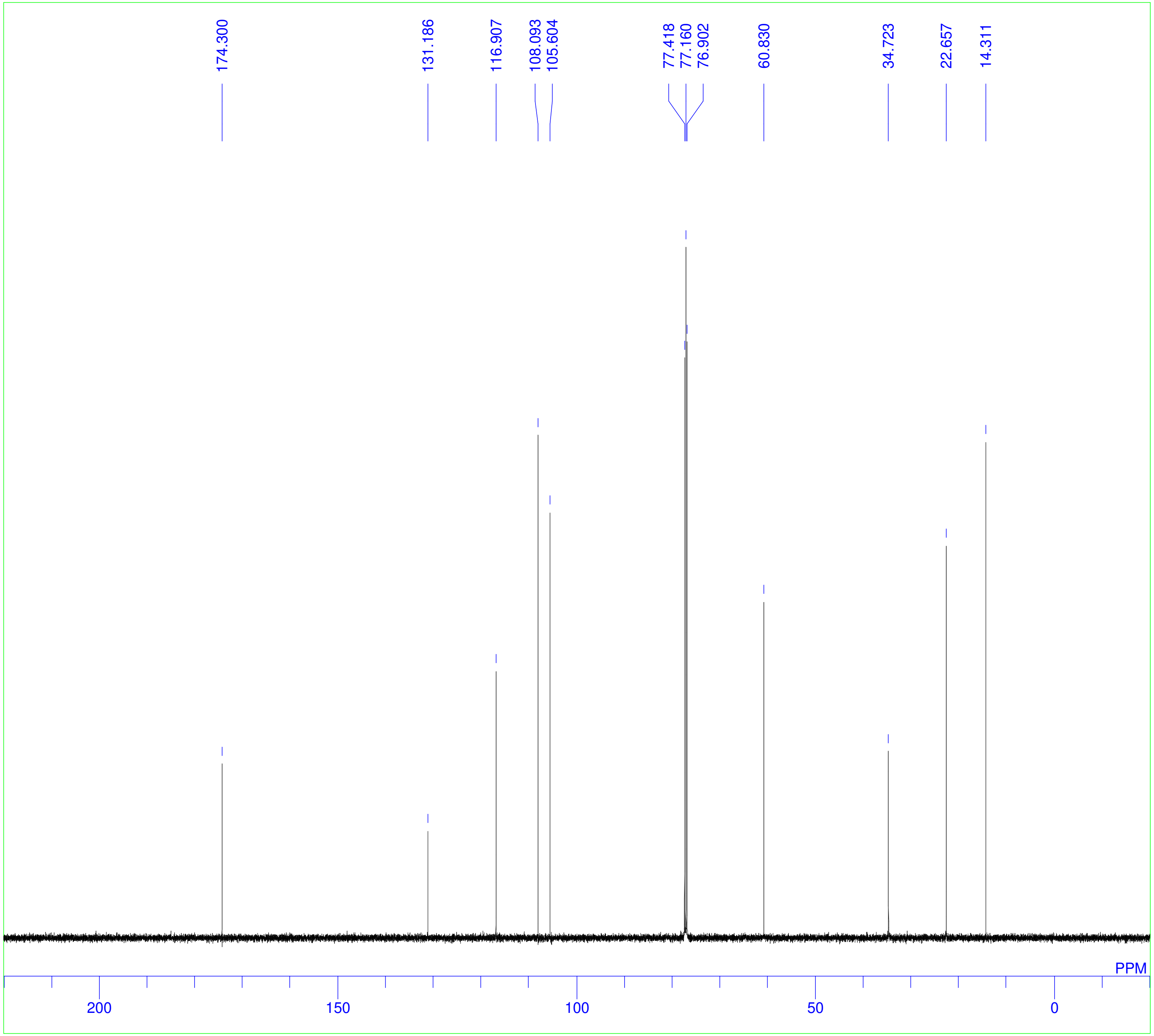

DFILE	S5_Carbon.als
COMNT	
DATIM	2024-05-07 15:38:49
OBNUC	13C
EXMOD	carbon.jxp
OBFRQ	125.77 MHz
OBSET	7.87 KHz
OBFIN	4.21 Hz
POINT	26214
FREQU	31446.54 Hz
SCANS	1024
ACQTM	0.8336 sec
PD	2.0000 sec
PW1	4.30 usec
IRNUC	1H
CTEMP	24.0 c
SLVNT	CDCL3
EXREF	77.16 ppm
BF	0.30 Hz
RGAIN	34

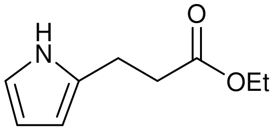

S5

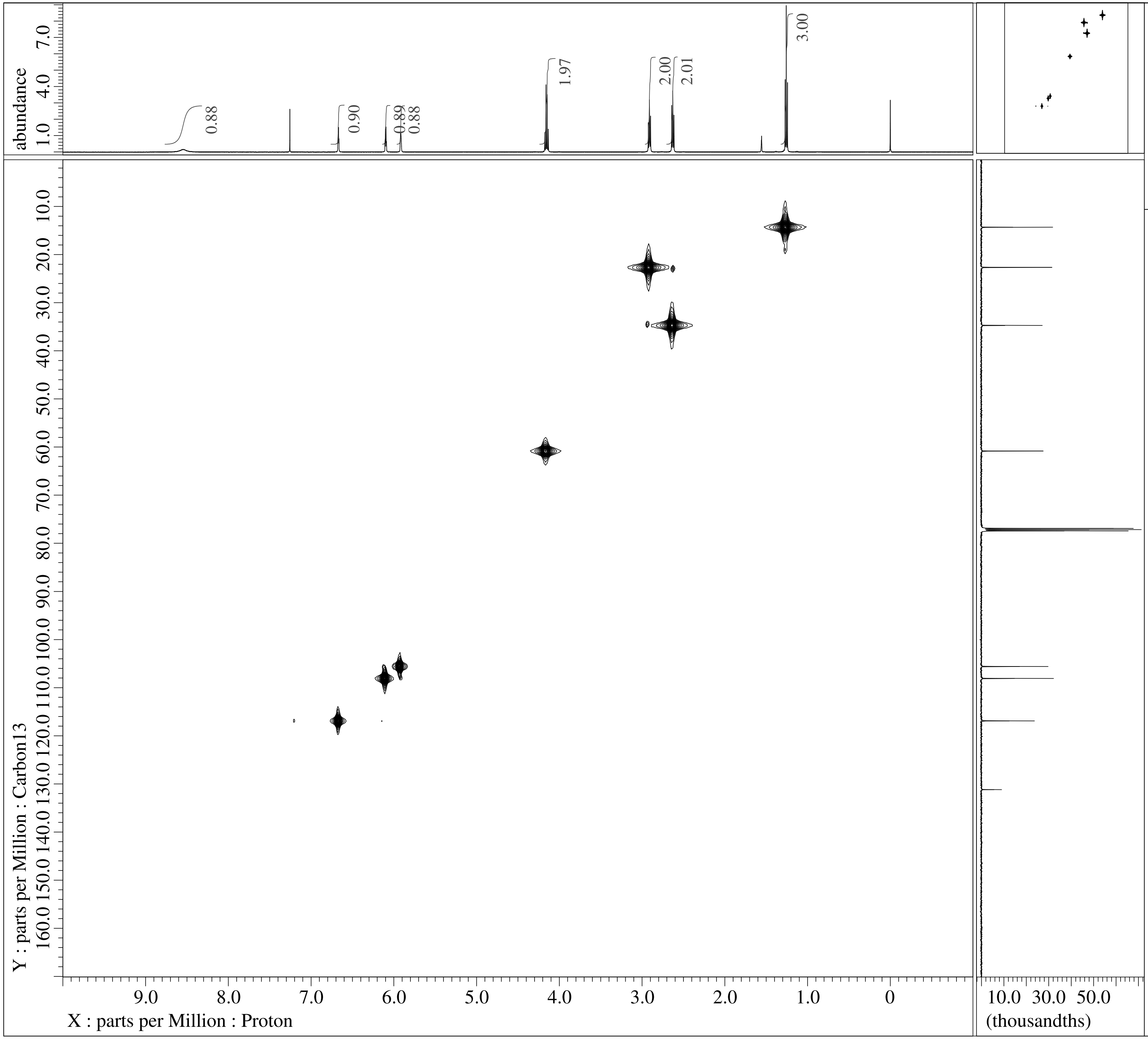


```
---- PROCESSING PARAMETERS ----
sinbell4( -60, 160 )
zerofill( 1 )
fft( 1, TRUE, TRUE )
ppm
[transpose]
sinbell4( -60, 160 )
zerofill( 2 )
fft( 1, TRUE, TRUE )
ppm
abs

Filename            = S5_HMQC-1-4.jdf
Author              = delta
Experiment          = hmqc.jxp
Sample_Id           = 2_BODIPY
Solvent             = CHLOROFORM-D
Creation_Time       = 16-MAY-2024 17:52:55
Revision_Time       = 17-JUN-2024 18:22:10
Current_Time        = 17-JUN-2024 18:23:01

Comment             = gradient enhanced HMQC
Data_Format         = 2D REAL REAL
Dim_Size            = 819, 512
Dim_Title           = Proton Carbon13
Dim_Units           = [ppm] [ppm]
Dimensions          = X Y
Site                = JNM-ECA500II
Spectrometer        = DELTA2_NMR

Field_Strength      = 11.7473579[T] (500[MHz]
X_Acq_Duration      = 0.10911744[s]
X_Domain            = 1H
X_Freq              = 500.15991521[MHz]
X_Offset            = 5.0[ppm]
X_Points            = 1024
X_Prescans          = 4
X_Resolution        = 9.16443788[Hz]
X_Sweep             = 9.38438438[kHz]
X_Sweep_Clipped     = 7.50750751[kHz]
Y_Domain            = 13C
Y_Freq              = 125.76529768[MHz]
Y_Offset            = 85[ppm]
Y_Points            = 256
Y_Prescans          = 0
Y_Resolution        = 83.60980308[Hz]
Y_Sweep             = 21.40410959[kHz]
Tri_Domain          = Proton
Tri_Freq            = 500.15991521[MHz]
Tri_Offset          = 5.0[ppm]
Clipped             = FALSE
Scans               = 4
Total_Scans         = 1024

Relaxation_Delay    = 1.5[s]
Recvr_Gain          = 50
Temp_Get            = 23.7[dC]
X_Acq_Time          = 0.10911744[s]
X_Atn               = 3.2[dB]
X_Gamma             = 42576375
X_Pulse             = 7.59[us]
```

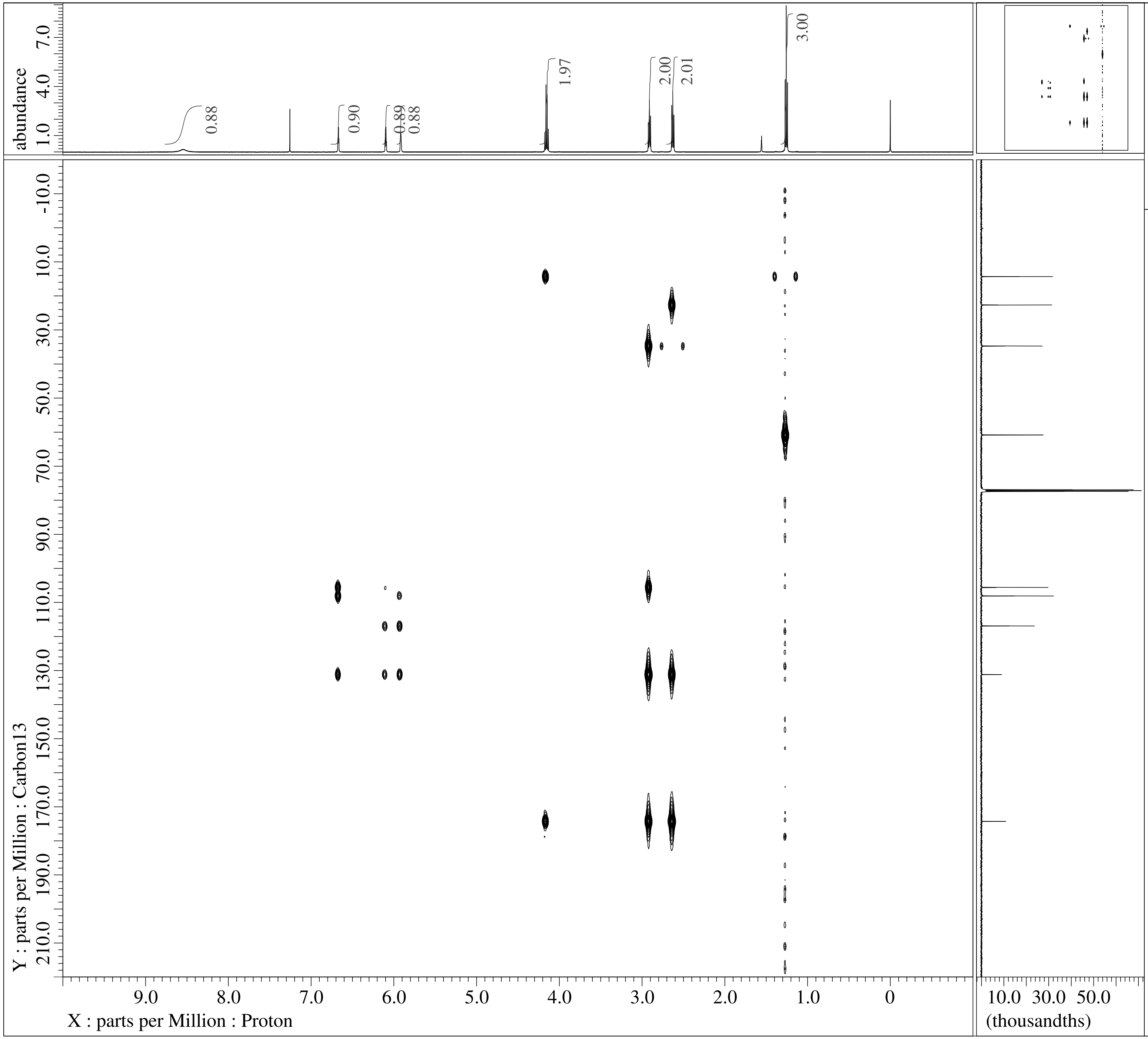

---- PROCESSING PARAMETERS ----
dc_balance(0, FALSE)
sexp(2.0[Hz], 0.0[s])
trapezoid(0[%], 0[%], 80[%], 100[%])
zerofill(1, TRUE)
fft(1, TRUE, TRUE)
machinephase
ppm

Filename	= S5_HMBC-1-4.jdf
Author	= delta
Experiment	= hmbc.jxp
Sample_Id	= 2_BODIPY
Solvent	= CHLOROFORM-D
Creation_Time	= 16-MAY-2024 18:21:15
Revision_Time	= 17-JUN-2024 18:19:50
Current_Time	= 17-JUN-2024 18:20:59
Comment	= gradient enhanced HMBC
Data_Format	= 2D REAL REAL
Dim_Size	= 1638, 512
Dim_Title	= Proton Carbon13
Dim_Units	= [ppm] [ppm]
Dimensions	= X Y
Site	= JNM-ECA500II
Spectrometer	= DELTA2_NMR
Field_Strength	= 11.7473579[T] (500[MHz]
X_Acq_Duration	= 0.21823488[s]
X_Domain	= 1H
X_Freq	= 500.15991521[MHz]
X_Offset	= 5.0[ppm]
X_Points	= 2048
X_Prescans	= 4
X_Resolution	= 4.58221894[Hz]
X_Sweep	= 9.38438438[kHz]
X_Sweep_Clipped	= 7.50750751[kHz]
Y_Domain	= 13C
Y_Freq	= 125.76529768[MHz]
Y_Offset	= 100[ppm]
Y_Points	= 256
Y_Prescans	= 0
Y_Resolution	= 122.99275819[Hz]
Y_Sweep	= 31.4861461[kHz]
Tri_Domain	= Proton
Tri_Freq	= 500.15991521[MHz]
Tri_Offset	= 5.0[ppm]
Clipped	= FALSE
Scans	= 8
Total_Scans	= 2048
Relaxation_Delay	= 1.5[s]
Recvr_Gain	= 50
Temp_Get	= 23.8[dC]
X_Acq_Time	= 0.21823488[s]
X_Atn	= 3.2[dB]
X_Gamma	= 42576375
X_Pulse	= 7.59[us]

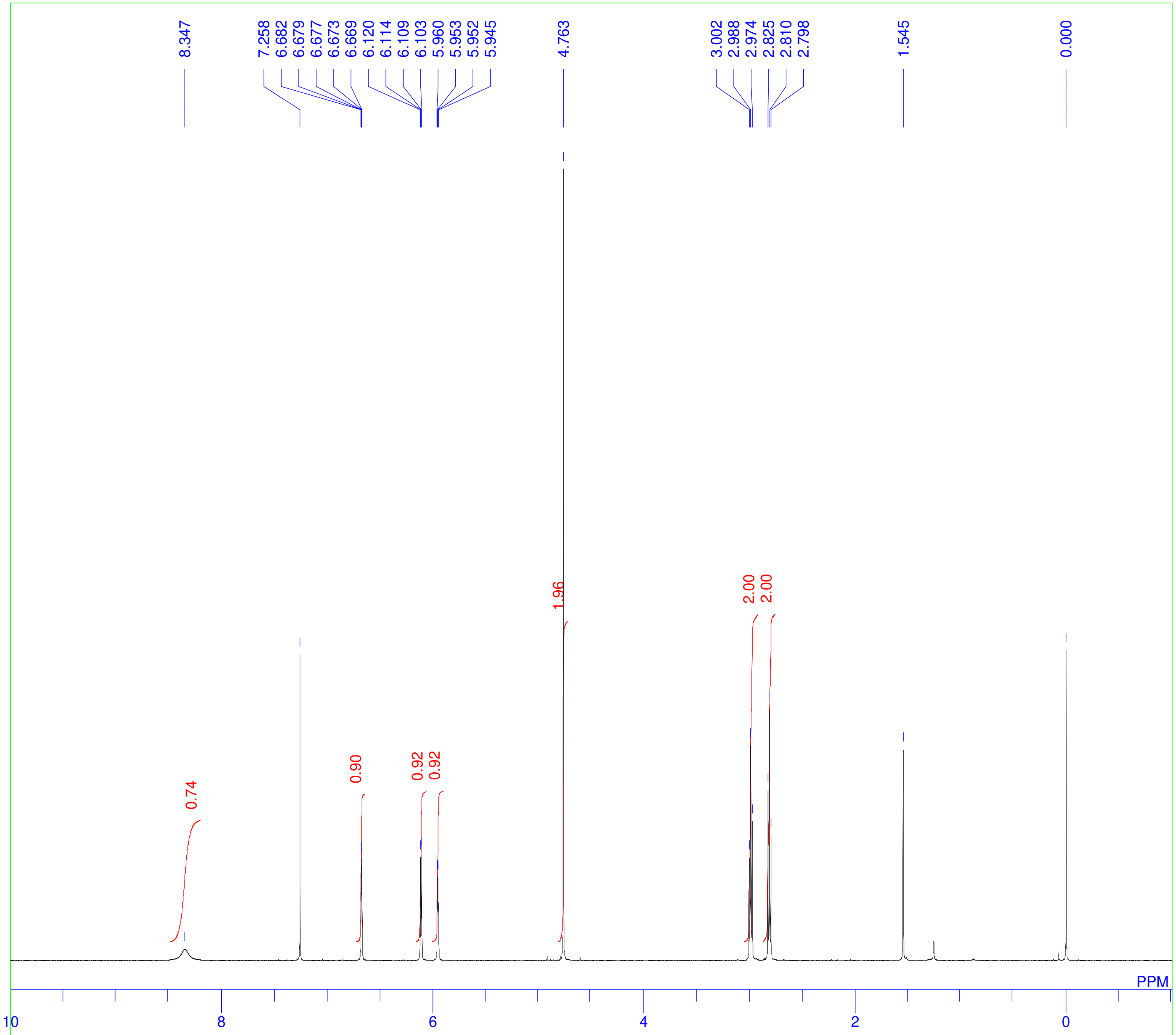

DFILE	S7_Proton.als
COMNT	
DATIM	2024-05-08 22:07:10
OBNUC	1H
EXMOD	proton.jxp
OBFRQ	500.16 MHz
OBSET	2.41 KHz
OBFIN	6.01 Hz
POINT	13107
FREQU	7507.51 Hz
SCANS	8
ACQTM	1.7459 sec
PD	5.0000 sec
PW1	3.80 usec
IRNUC	1H
CTEMP	23.8 c
SLVNT	CDCL3
EXREF	0.00 ppm
BF	0.30 Hz
RGAIN	46

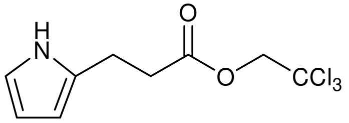

S7

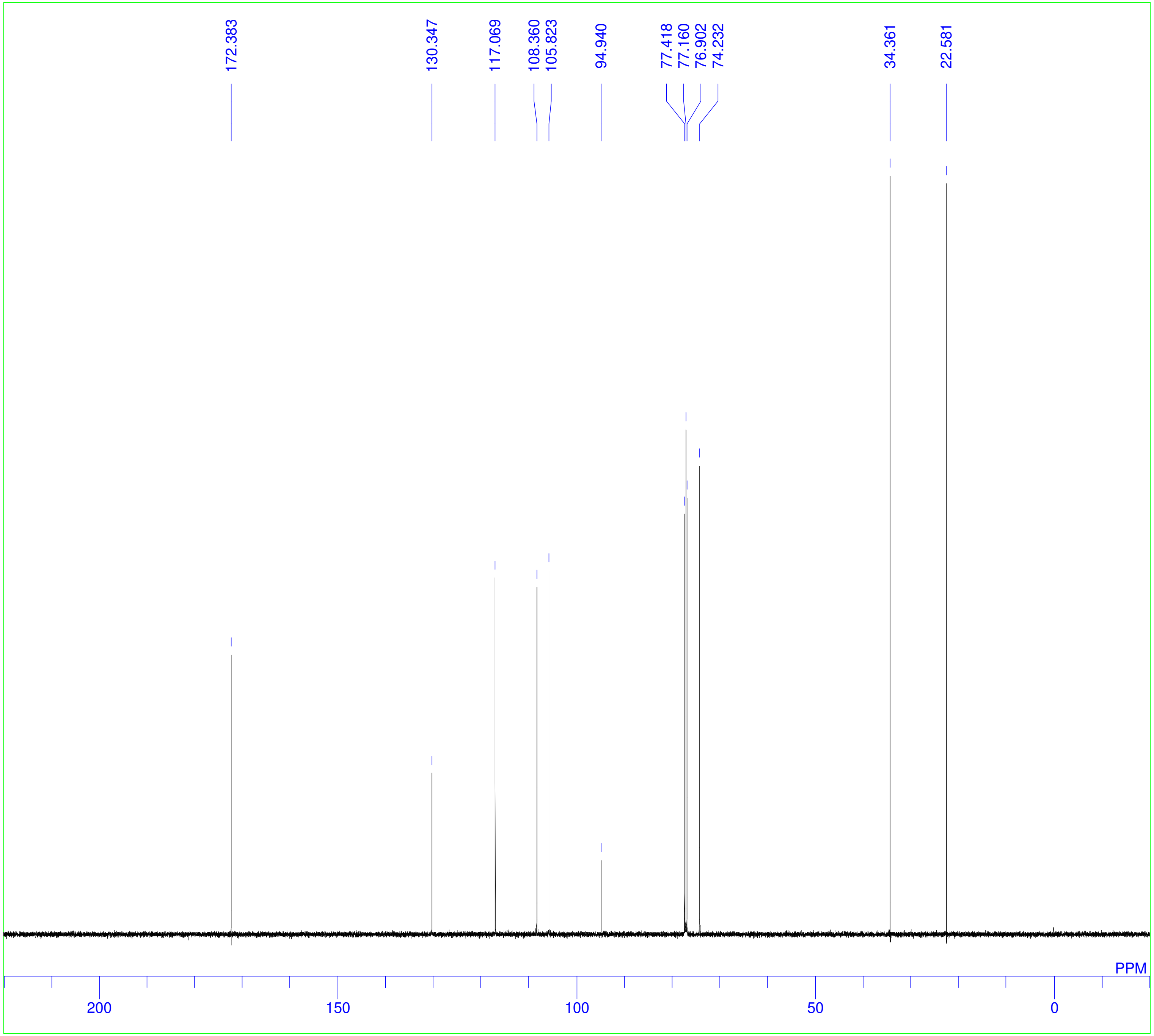

DFILE	S7_Carbon.als
COMNT	
DATIM	2024-05-09 15:06:31
OBNUC	13C
EXMOD	carbon.jxp
OBFRQ	125.77 MHz
OBSET	7.87 KHz
OBFIN	4.21 Hz
POINT	26214
FREQU	31446.54 Hz
SCANS	1024
ACQTM	0.8336 sec
PD	2.0000 sec
PW1	4.30 usec
IRNUC	1H
CTEMP	23.9 c
SLVNT	CDCL3
EXREF	77.16 ppm
BF	0.30 Hz
RGAIN	36

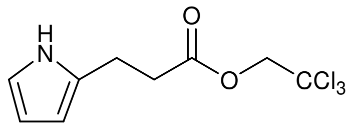

S7

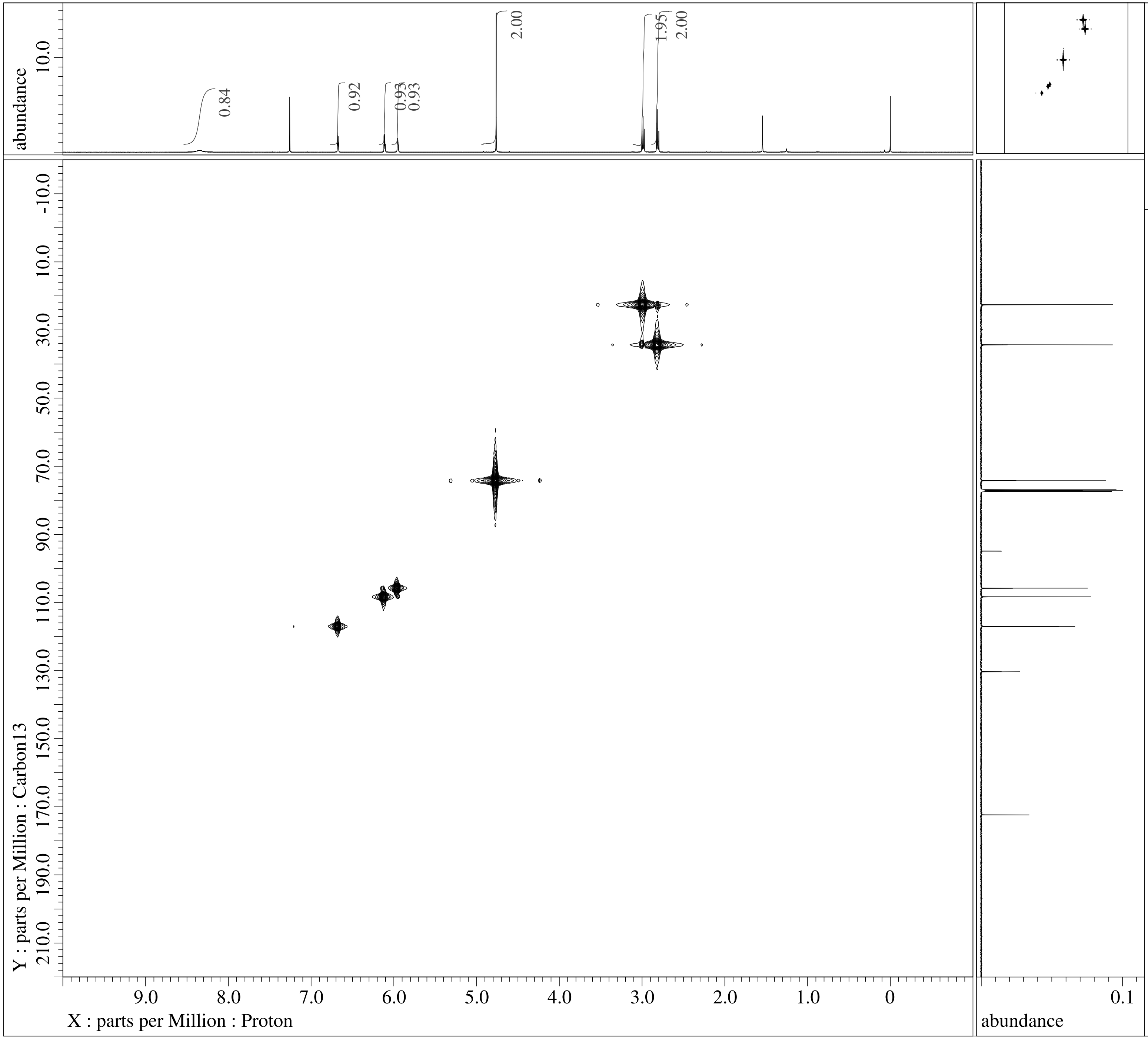


```
---- PROCESSING PARAMETERS ----
dc_balance( 0, FALSE )
sexp( 2.0[Hz], 0.0[s] )
trapezoid( 0[%], 0[%], 80[%], 100[%] )
zerofill( 1, TRUE )
fft( 1, TRUE, TRUE )
machinephase
ppm

Filename            = S7_HMQC-1-4.jdf
Author              = delta
Experiment          = hmqc.jxp
Sample_Id           = KK
Solvent             = CHLOROFORM-D
Creation_Time       =  9-MAY-2024 15:55:22
Revision_Time       = 17-JUN-2024 18:25:03
Current_Time        = 17-JUN-2024 18:26:24

Comment             = gradient enhanced HMQC
Data_Format         = 2D REAL REAL
Dim_Size            = 819, 512
Dim_Title           = Proton Carbon13
Dim_Units           = [ppm] [ppm]
Dimensions          = X Y
Site                = JNM-ECA500II
Spectrometer        = DELTA2_NMR

Field_Strength      = 11.7473579[T] (500[MHz]
X_Acq_Duration      = 0.10911744[s]
X_Domain            = 1H
X_Freq              = 500.15991521[MHz]
X_Offset            = 5.0[ppm]
X_Points            = 1024
X_Prescans          = 4
X_Resolution        = 9.16443788[Hz]
X_Sweep             = 9.38438438[kHz]
X_Sweep_Clipped     = 7.50750751[kHz]
Y_Domain            = 13C
Y_Freq              = 125.76529768[MHz]
Y_Offset            = 97.4675746[ppm]
Y_Points            = 256
Y_Prescans          = 0
Y_Resolution        = 95.74142157[Hz]
Y_Sweep             = 24.50980392[kHz]
Tri_Domain          = Proton
Tri_Freq            = 500.15991521[MHz]
Tri_Offset          = 5.0[ppm]
Clipped             = FALSE
Scans               = 4
Total_Scans         = 1024

Relaxation_Delay    = 1.5[s]
Recvr_Gain          = 50
Temp_Get            = 24.1[dC]
X_Acq_Time          = 0.10911744[s]
X_Atn               = 3.2[dB]
X_Gamma             = 42576375
X_Pulse             = 7.59[us]
```

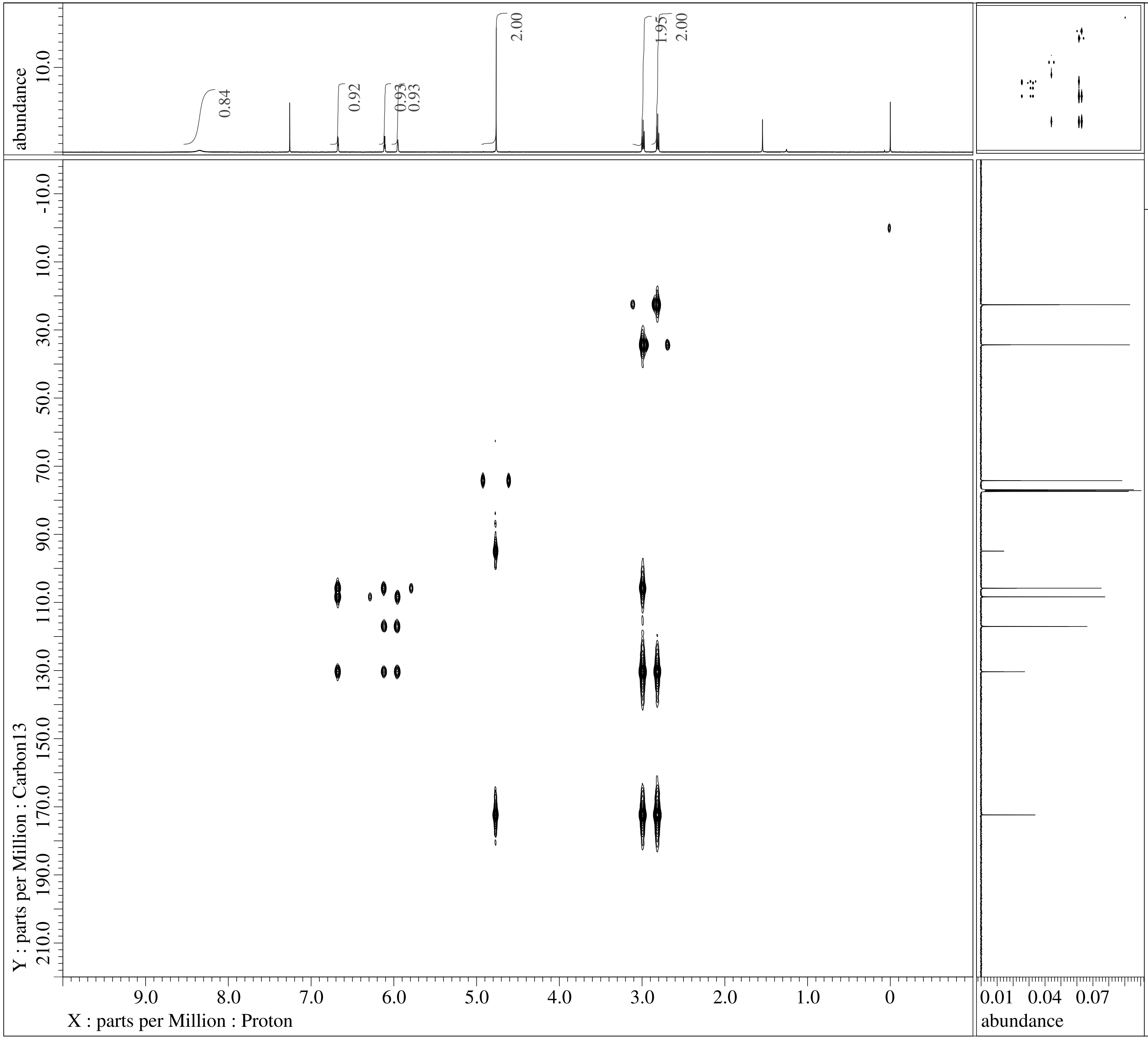


```
---- PROCESSING PARAMETERS ----
dc_balance( 0, FALSE )
sexp( 0.2[Hz], 0.0[s] )
trapezoid( 0[%], 0[%], 80[%], 100[%] )
zerofill( 1, TRUE )
fft( 1, TRUE, TRUE )
machinephase
ppm
```

```
Filename            = S7_HMBC-1-4.jdf
Author              = delta
Experiment          = hmbc.jxp
Sample_Id           = kk-237-TM-HMBC
Solvent             = CHLOROFORM-D
Creation_Time       = 17-MAY-2024 10:38:51
Revision_Time       = 17-JUN-2024 18:30:34
Current_Time        = 17-JUN-2024 18:31:38

Comment             = gradient enhanced HMBC
Data_Format         = 2D REAL REAL
Dim_Size            = 1638, 512
Dim_Title           = Proton Carbon13
Dim_Units           = [ppm] [ppm]
Dimensions          = X Y
Site                = JNM-ECA500II
Spectrometer        = DELTA2_NMR

Field_Strength      = 11.7473579[T] (500[MHz]
X_Acq_Duration      = 0.30179328[s]
X_Domain            = 1H
X_Freq              = 500.15991521[MHz]
X_Offset            = 4.16744798[ppm]
X_Points            = 2048
X_Prescans          = 4
X_Resolution        = 3.3135264[Hz]
X_Sweep             = 6.78610206[kHz]
X_Sweep_Clipped     = 5.42888165[kHz]
Y_Domain            = 13C
Y_Freq              = 125.76529768[MHz]
Y_Offset            = 100[ppm]
Y_Points            = 256
Y_Prescans          = 0
Y_Resolution        = 122.99275819[Hz]
Y_Sweep             = 31.4861461[kHz]
Tri_Domain          = Proton
Tri_Freq            = 500.15991521[MHz]
Tri_Offset          = 5.0[ppm]
Clipped             = FALSE
Scans               = 8
Total_Scans         = 2048

Relaxation_Delay    = 1.5[s]
Recvr_Gain          = 50
Temp_Get            = 24[dC]
X_Acq_Time          = 0.30179328[s]
X_Atn               = 3.2[dB]
X_Gamma             = 42576375
X_Pulse             = 7.59[us]
```

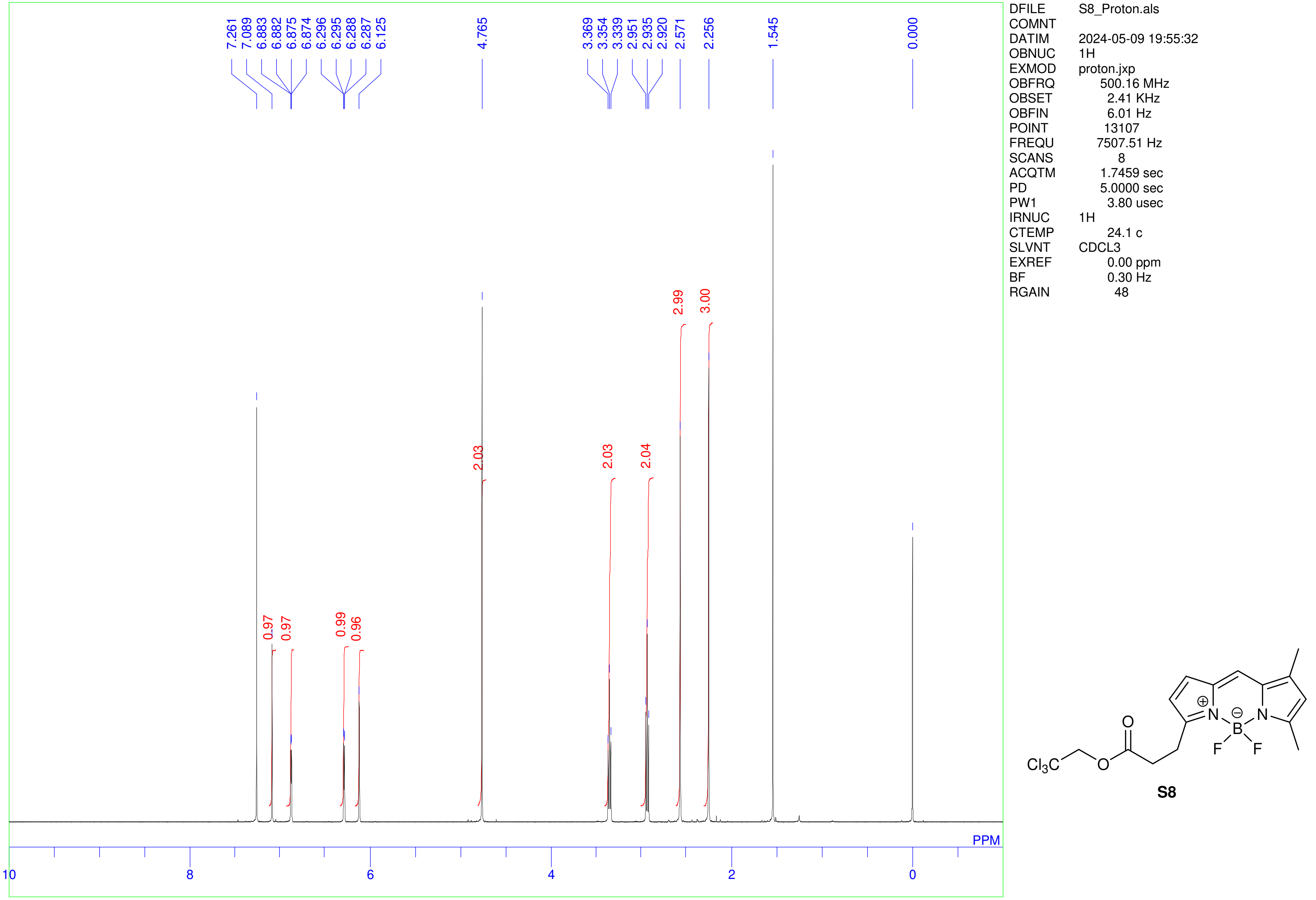
7.261 7.089 6.883 6.882 6.875 6.874 6.296 6.295 6.288 6.287 6.125
4.765
3.369 3.354 3.339 2.951 2.935 2.920 2.571
2.256
1.545
0.000
0.97 0.97 0.99 0.96 2.03 2.03 2.04 2.99 3.00
PPM
10 8 6 4 2 0
DFILE S8_Proton.als
COMNT
DATIM 2024-05-09 19:55:32
OBNUC 1H
EXMOD proton.jxp
OBFRQ 500.16 MHz
OBSET 2.41 KHz
OBFIN 6.01 Hz
POINT 13107
FREQU 7507.51 Hz
SCANS 8
ACQTM 1.7459 sec
PD 5.0000 sec
PW1 3.80 usec
IRNUC 1H
CTEMP 24.1 c
SLVNT CDCL3
EXREF 0.00 ppm
BF 0.30 Hz
RGAIN 48
Cl3C
O
O
N
B
N
F
F

S8

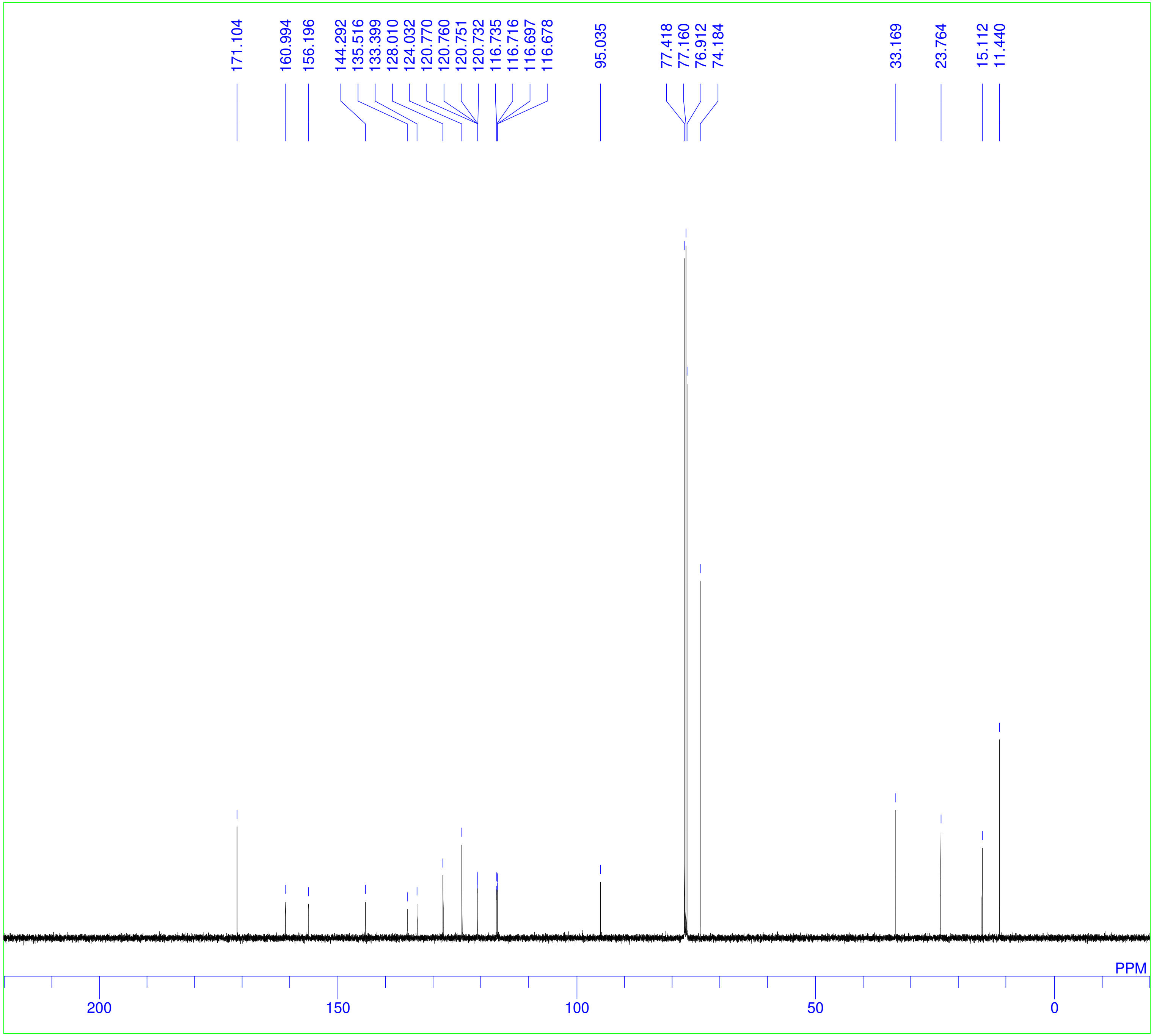

DFILE	S8_Carbon.als
COMNT	
DATIM	2024-02-14 13:32:36
OBNUC	13C
EXMOD	carbon.jxp
OBFRQ	125.77 MHz
OBSET	7.87 KHz
OBFIN	4.21 Hz
POINT	26214
FREQU	31446.54 Hz
SCANS	1024
ACQTM	0.8336 sec
PD	2.0000 sec
PW1	4.30 usec
IRNUC	1H
CTEMP	22.4 c
SLVNT	CDCL3
EXREF	77.16 ppm
BF	0.30 Hz
RGAIN	40

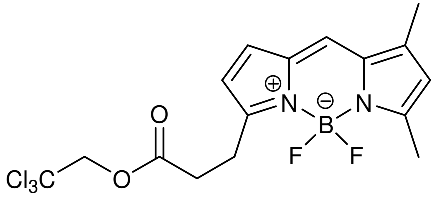

S8

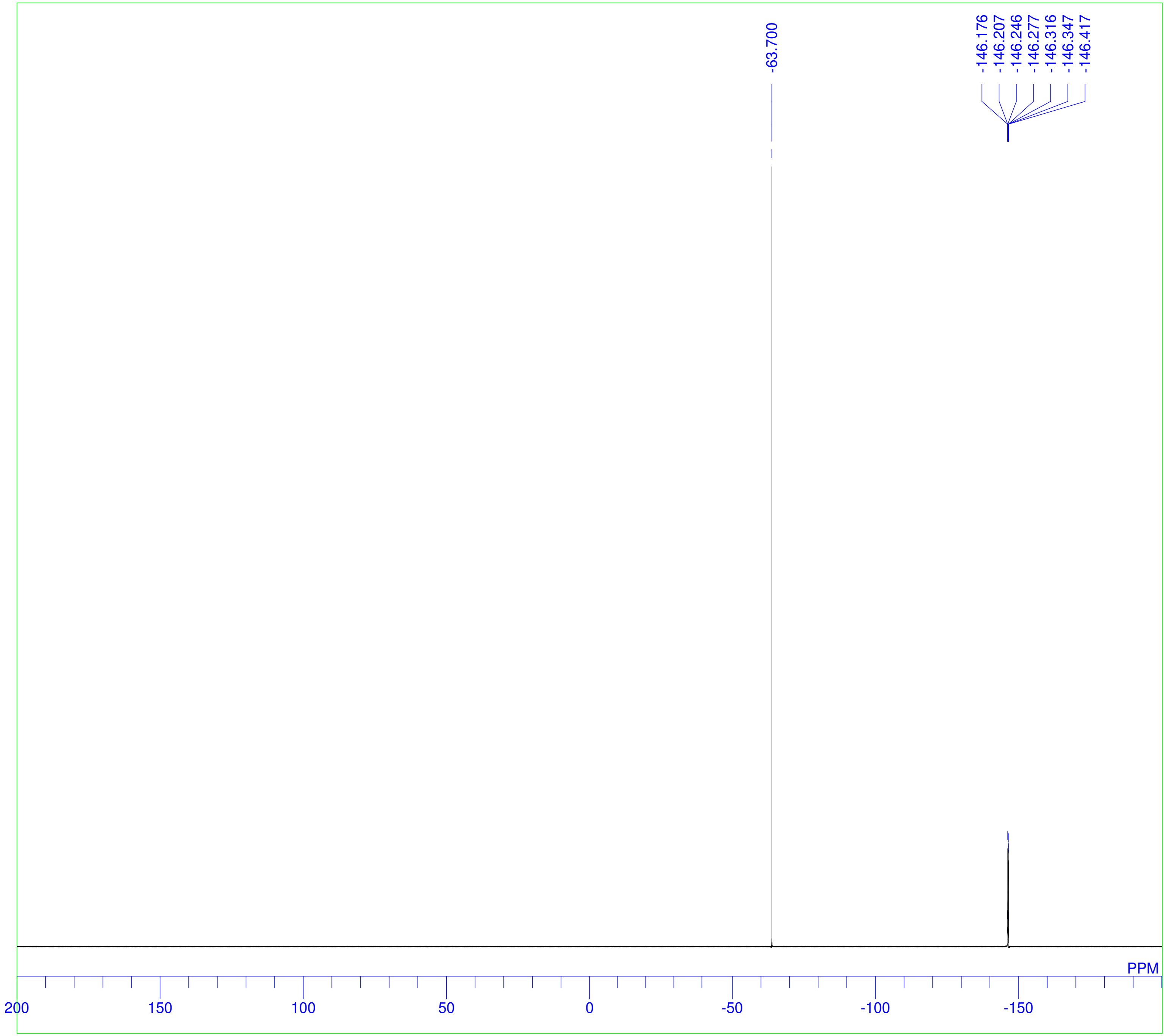

DFILE	S8_Fluorine.als
COMNT	
DATIM	2024-06-11 14:36:04
OBNUC	19F
EXMOD	proton.jxp
OBFRQ	470.62 MHz
OBSET	0.46 KHz
OBFIN	0.84 Hz
POINT	104857
FREQU	192307.69 Hz
SCANS	128
ACQTM	0.5453 sec
PD	5.0000 sec
PW1	4.25 usec
IRNUC	19F
CTEMP	24.2 c
SLVNT	CDCL3
EXREF	-63.70 ppm
BF	0.30 Hz
RGAIN	46

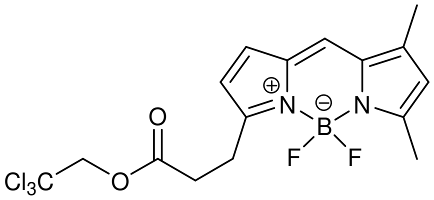

S8

Trifluoromethylbenzene as an internal standard

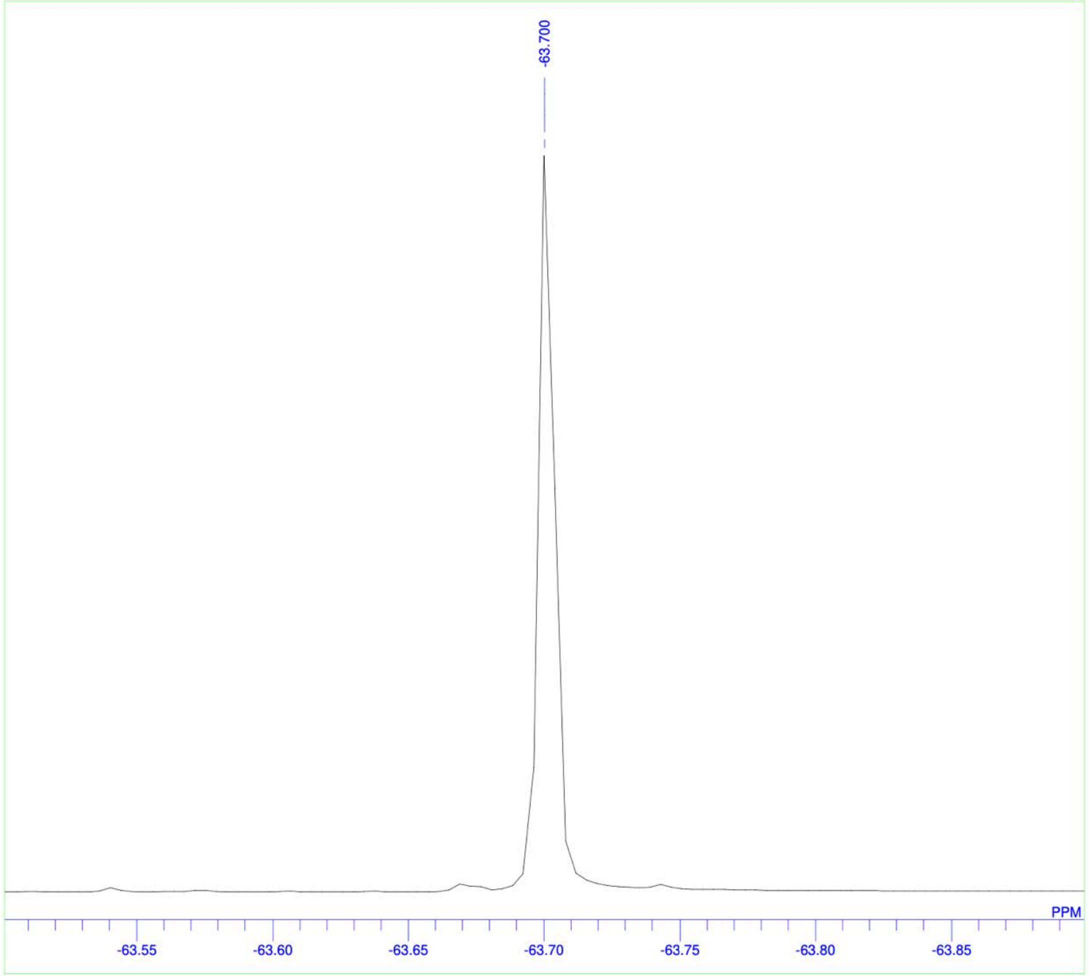

Fluorine of **S8**

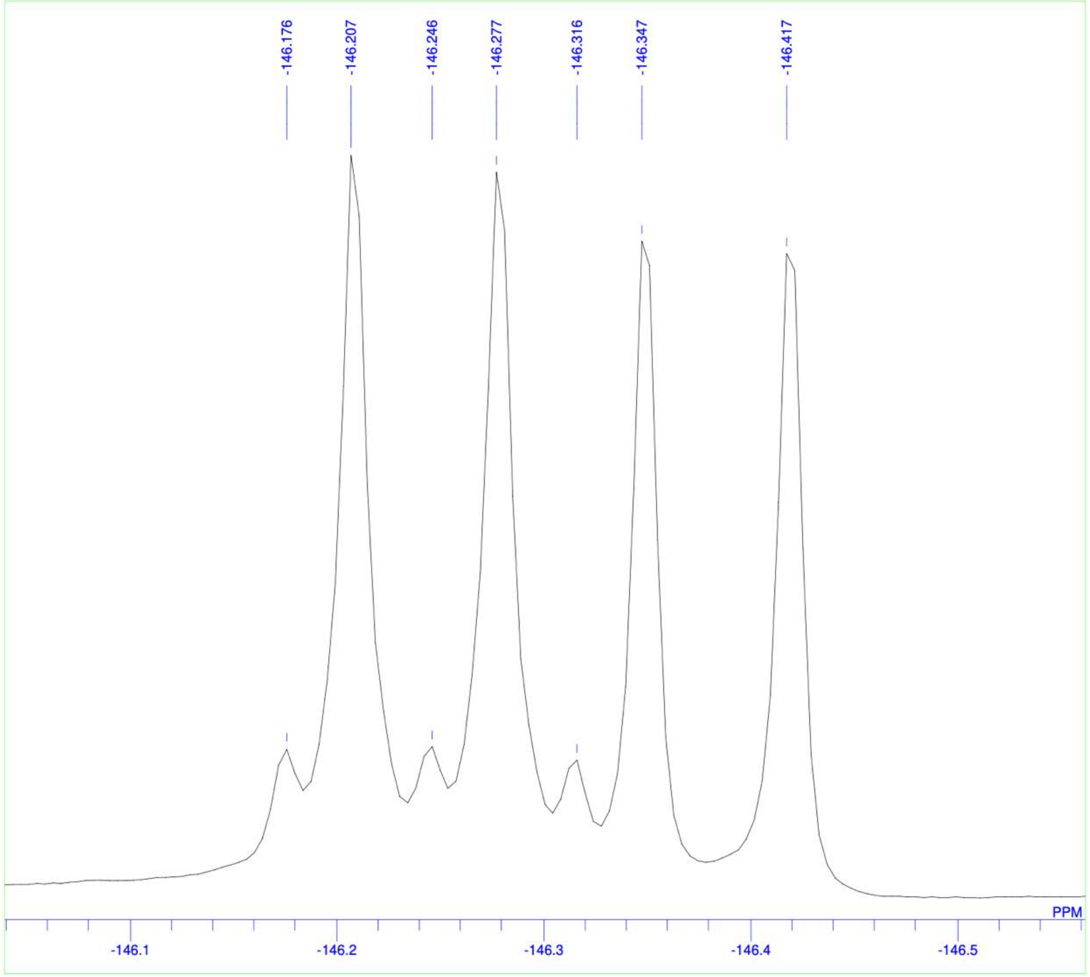

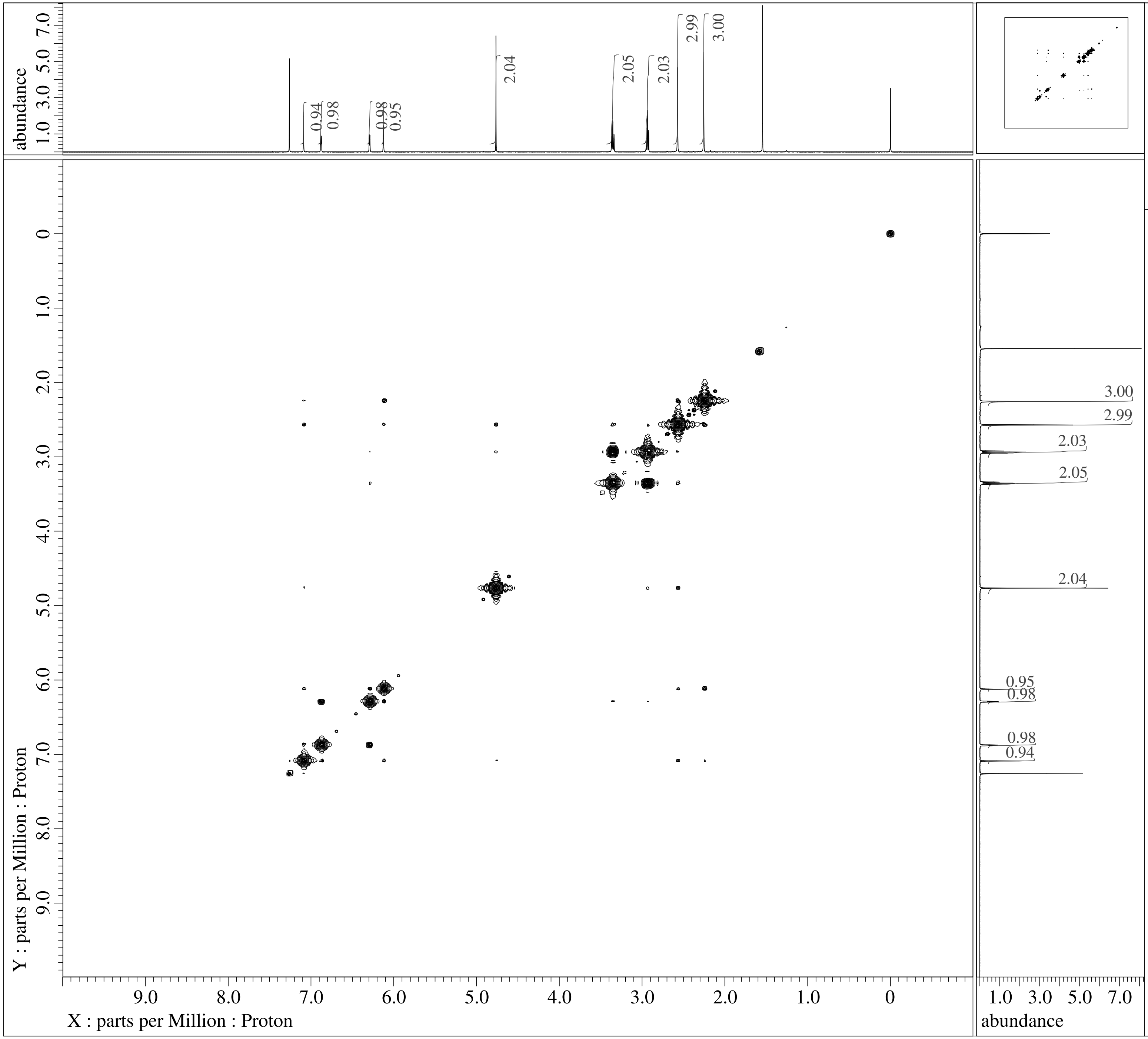


```
---- PROCESSING PARAMETERS ----
sinbell_auto
fft( 1, TRUE, TRUE )
ppm
[transpose]
sinbell_auto
zerofill( 4 )
fft( 1, TRUE, TRUE )
ppm
abs
symmetrize( Cosy, 24 )
```

```
Filename          = S8_COSY-1-4.jdf
Author            = delta
Experiment        = cosy.jxp
Sample_Id         = 4_BODIPY
Solvent           = CHLOROFORM-D
Creation_Time     = 18-MAY-2024 11:27:04
Revision_Time     = 17-JUN-2024 18:38:16
Current_Time      = 17-JUN-2024 18:39:54

Comment           = gradient absolute value co
Data_Format       = 2D REAL REAL
Dim_Size          = 1024, 1024
Dim_Title         = Proton Proton
Dim_Units         = [ppm] [ppm]
Dimensions        = X Y
Site              = JNM-ECA500II
Spectrometer      = DELTA2_NMR

Field_Strength    = 11.7473579[T] (500[MHz])
X_Acq_Duration    = 0.1363968[s]
X_Domain          = 1H
X_Freq            = 500.15991521[MHz]
X_Offset          = 5.0[ppm]
X_Points          = 1280
X_Prescans        = 4
X_Resolution      = 7.3315503[Hz]
X_Sweep           = 9.38438438[kHz]
X_Sweep_Clipped   = 7.50750751[kHz]
Y_Domain          = 1H
Y_Freq            = 500.15991521[MHz]
Y_Offset          = 5.0[ppm]
Y_Points          = 256
Y_Prescans        = 0
Y_Resolution      = 29.31299715[Hz]
Y_Sweep           = 7.50412727[kHz]
Irr_Domain        = Proton
Irr_Freq          = 500.15991521[MHz]
Irr_Offset        = 5.0[ppm]
Tri_Domain        = Proton
Tri_Freq          = 500.15991521[MHz]
Tri_Offset        = 5.0[ppm]
Clipped           = FALSE
Scans             = 2
Total_Scans       = 512

Relaxation_Delay  = 1.5[s]
Recvr_Gain        = 40
Temp_Get          = 24[dC]
X_90_Width        = 7.59[us]
```

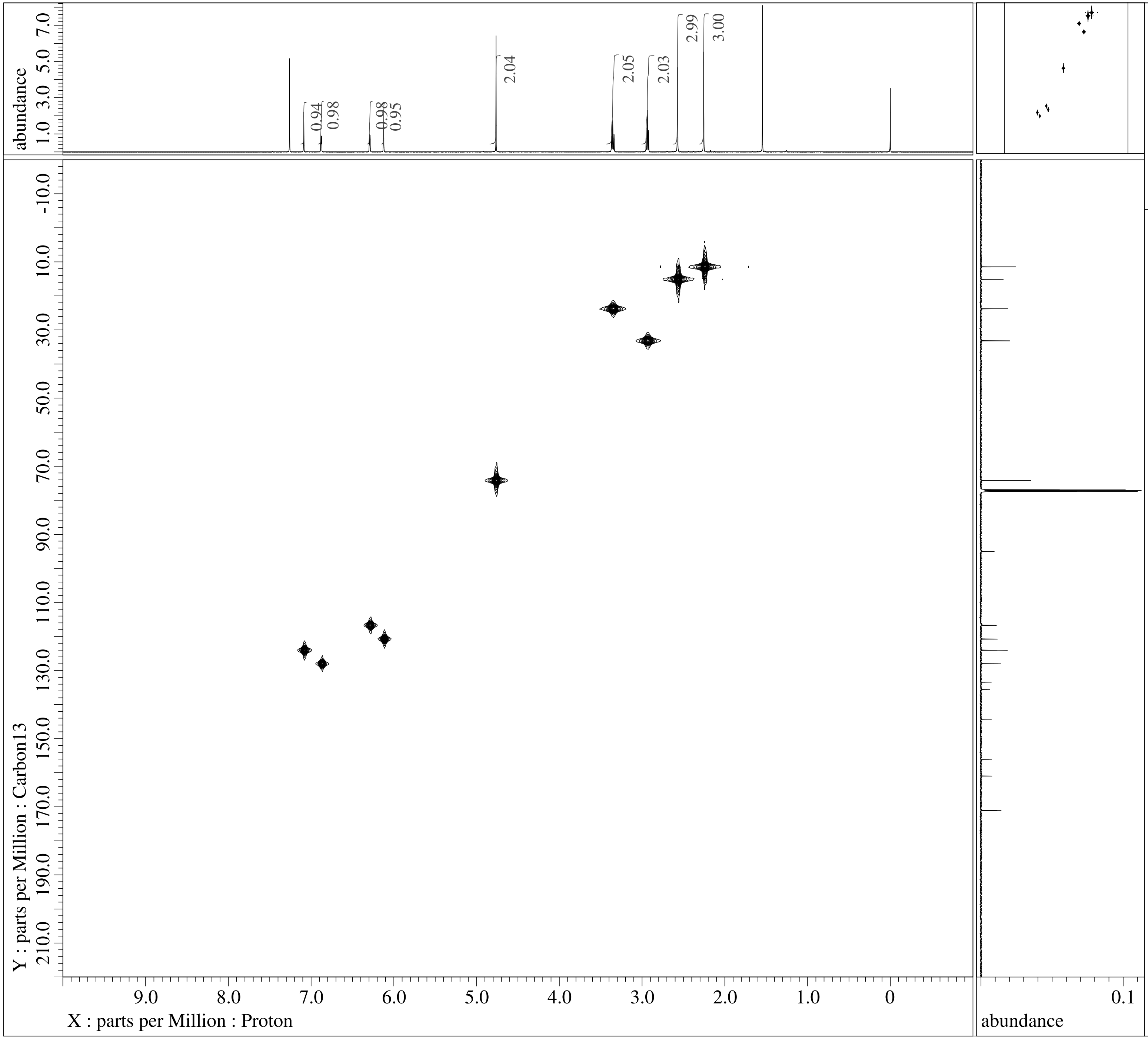


```
---- PROCESSING PARAMETERS ----
dc_balance( 0, FALSE )
sexp( 2.0[Hz], 0.0[s] )
trapezoid( 0[%], 0[%], 80[%], 100[%] )
zerofill( 1, TRUE )
fft( 1, TRUE, TRUE )
machinephase
ppm

Filename              = S8_HMQC-1-4.jdf
Author                = delta
Experiment            = hmqc.jxp
Sample_Id             = kk-CCl3 ester 1
Solvent               = CHLOROFORM-D
Creation_Time         =  9-MAY-2024 20:02:52
Revision_Time         = 17-JUN-2024 18:33:37
Current_Time          = 17-JUN-2024 18:34:40

Comment               = gradient enhanced HMQC
Data_Format           = 2D REAL REAL
Dim_Size              = 819, 512
Dim_Title             = Proton Carbon13
Dim_Units             = [ppm] [ppm]
Dimensions            = X Y
Site                  = JNM-ECA500II
Spectrometer          = DELTA2_NMR

Field_Strength        = 11.7473579[T] (500[MHz]
X_Acq_Duration        = 0.10911744[s]
X_Domain              = 1H
X_Freq                = 500.15991521[MHz]
X_Offset              = 5.0[ppm]
X_Points              = 1024
X_Prescans            = 4
X_Resolution          = 9.16443788[Hz]
X_Sweep               = 9.38438438[kHz]
X_Sweep_Clipped       = 7.50750751[kHz]
Y_Domain              = 13C
Y_Freq                = 125.76529768[MHz]
Y_Offset              = 85[ppm]
Y_Points              = 256
Y_Prescans            = 0
Y_Resolution          = 83.60980308[Hz]
Y_Sweep               = 21.40410959[kHz]
Tri_Domain            = Proton
Tri_Freq              = 500.15991521[MHz]
Tri_Offset            = 5.0[ppm]
Clipped               = FALSE
Scans                 = 4
Total_Scans           = 1024

Relaxation_Delay      = 1.5[s]
Recvr_Gain            = 50
Temp_Get              = 23.9[dC]
X_Acq_Time            = 0.10911744[s]
X_Atn                 = 3.2[dB]
X_Gamma               = 42576375
X_Pulse               = 7.59[us]
```

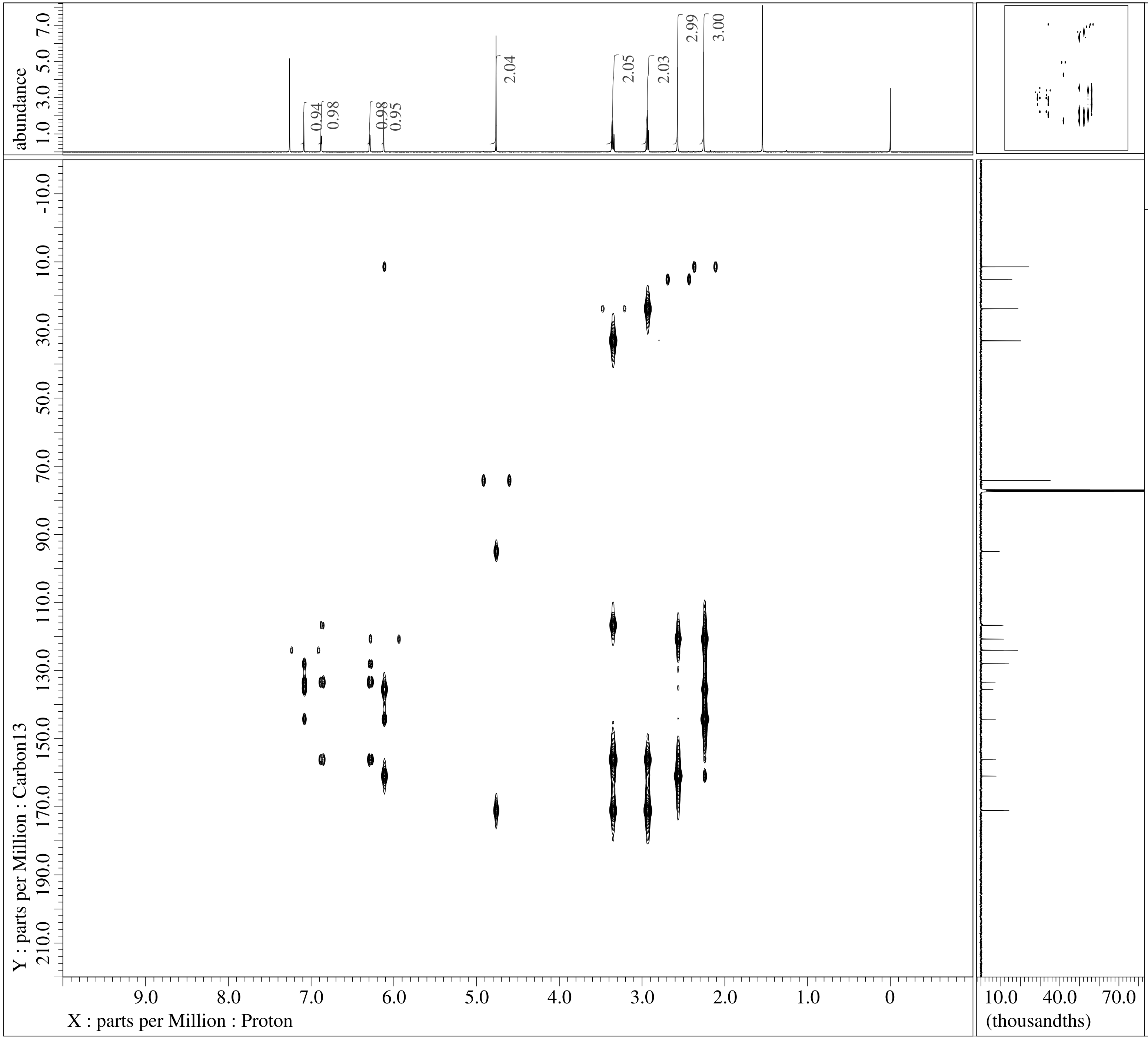


```
---- PROCESSING PARAMETERS ----
dc_balance( 0, FALSE )
sexp( 2.0[Hz], 0.0[s] )
trapezoid( 0[%], 0[%], 80[%], 100[%] )
zerofill( 1, TRUE )
fft( 1, TRUE, TRUE )
machinephase
ppm

Filename            = S8_HMBC-1-4.jdf
Author              = delta
Experiment          = hmbc.jxp
Sample_Id           = kk-CCl3 ester 1
Solvent             = CHLOROFORM-D
Creation_Time       =  9-MAY-2024 20:31:11
Revision_Time       = 17-JUN-2024 18:35:59
Current_Time        = 17-JUN-2024 18:36:57

Comment             = gradient enhanced HMBC
Data_Format         = 2D REAL REAL
Dim_Size            = 1638, 512
Dim_Title           = Proton Carbon13
Dim_Units           = [ppm] [ppm]
Dimensions          = X Y
Site                = JNM-ECA500II
Spectrometer        = DELTA2_NMR

Field_Strength      = 11.7473579[T] (500[MHz]
X_Acq_Duration      = 0.21823488[s]
X_Domain            = 1H
X_Freq              = 500.15991521[MHz]
X_Offset            = 5.0[ppm]
X_Points            = 2048
X_Prescans          = 4
X_Resolution        = 4.58221894[Hz]
X_Sweep             = 9.38438438[kHz]
X_Sweep_Clipped     = 7.50750751[kHz]
Y_Domain            = 13C
Y_Freq              = 125.76529768[MHz]
Y_Offset            = 100[ppm]
Y_Points            = 256
Y_Prescans          = 0
Y_Resolution        = 122.99275819[Hz]
Y_Sweep             = 31.4861461[kHz]
Tri_Domain          = Proton
Tri_Freq            = 500.15991521[MHz]
Tri_Offset          = 5.0[ppm]
Clipped             = FALSE
Scans               = 8
Total_Scans         = 2048

Relaxation_Delay    = 1.5[s]
Recvr_Gain          = 50
Temp_Get            = 23.8[dC]
X_Acq_Time          = 0.21823488[s]
X_Atn               = 3.2[dB]
X_Gamma             = 42576375
X_Pulse             = 7.59[us]
```

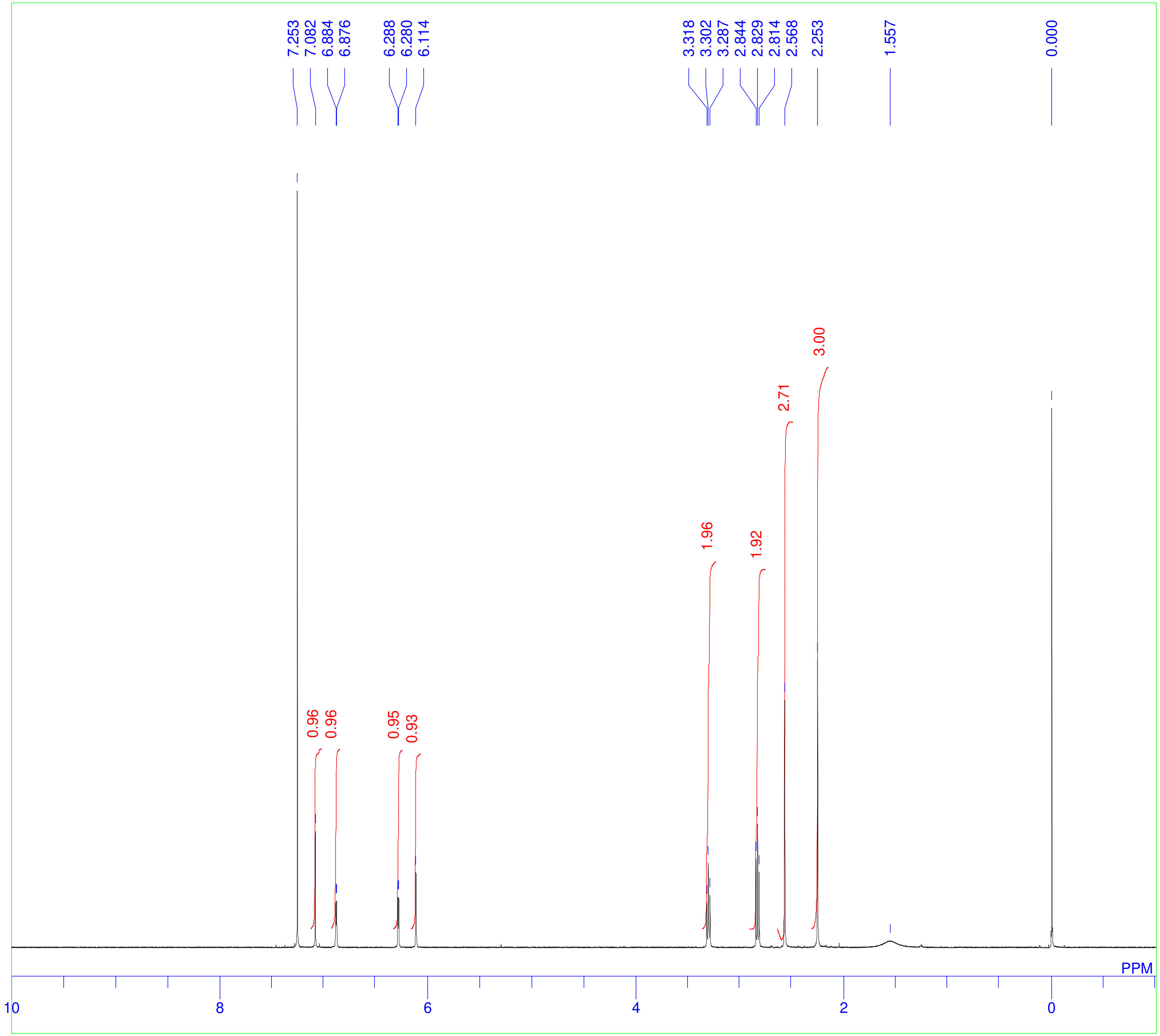

DFILE	BODIPY FL_Proton.als
COMNT	
DATIM	2024-05-26 09:53:23
OBNUC	1H
EXMOD	proton.jxp
OBFRQ	500.16 MHz
OBSET	2.41 KHz
OBFIN	6.01 Hz
POINT	13120
FREQU	7507.51 Hz
SCANS	8
ACQTM	1.7459 sec
PD	5.0000 sec
PW1	3.80 usec
IRNUC	1H
CTEMP	24.0 c
SLVNT	CDCL3
EXREF	0.00 ppm
BF	0.30 Hz
RGAIN	48

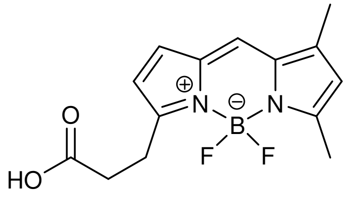

BODIPY FL

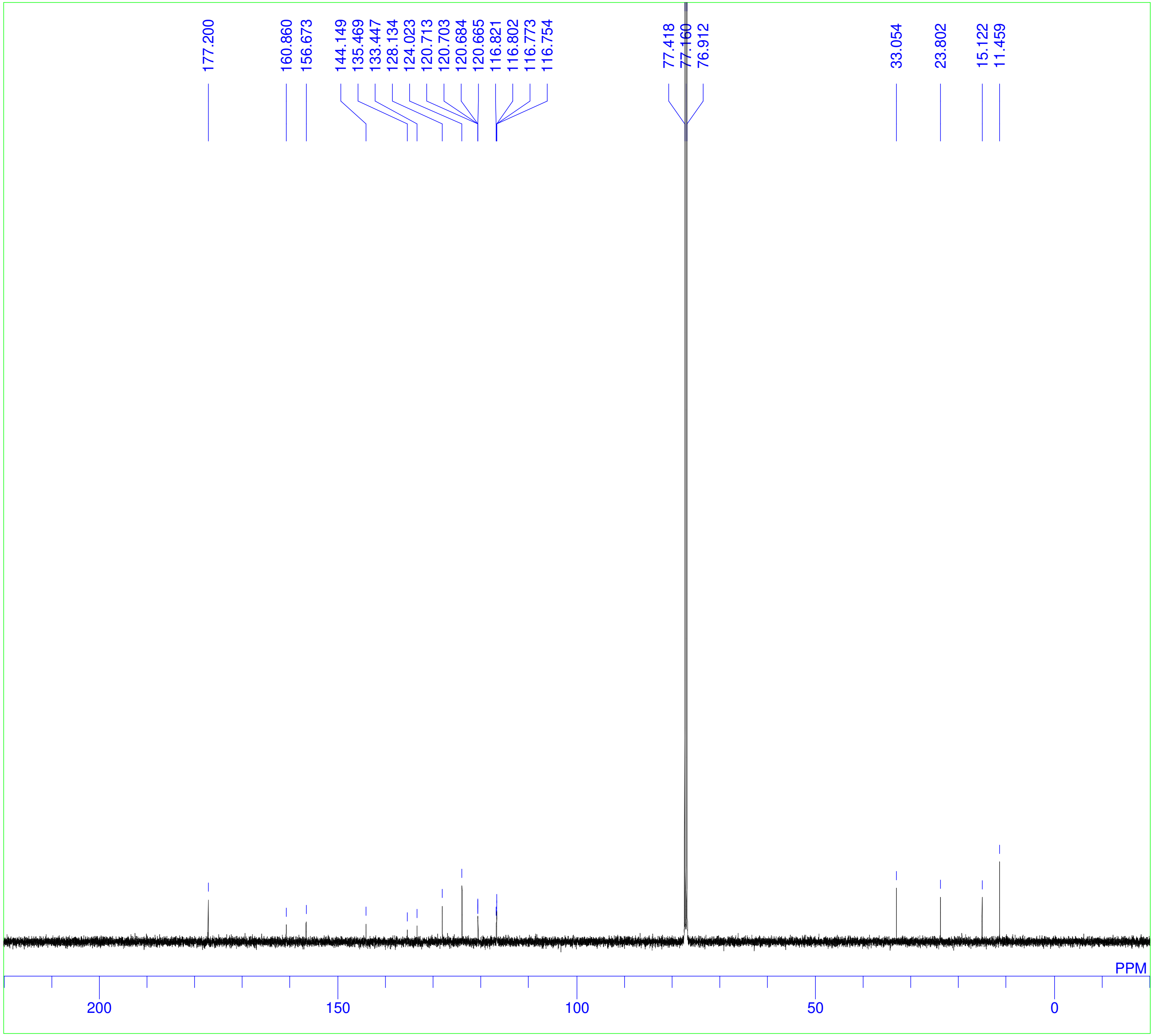

DFILE	BODIPY FL_Carbon.als
COMNT	
DATIM	2024-05-27 09:45:22
OBNUC	13C
EXMOD	carbon.jxp
OBFRQ	125.77 MHz
OBSET	7.87 KHz
OBFIN	4.21 Hz
POINT	26214
FREQU	31446.54 Hz
SCANS	4096
ACQTM	0.8336 sec
PD	2.0000 sec
PW1	4.30 usec
IRNUC	1H
CTEMP	23.8 c
SLVNT	CDCL3
EXREF	77.16 ppm
BF	0.30 Hz
RGAIN	40

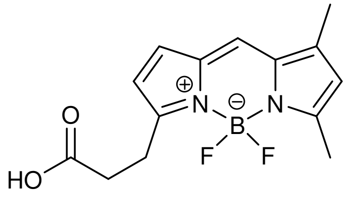

BODIPY FL

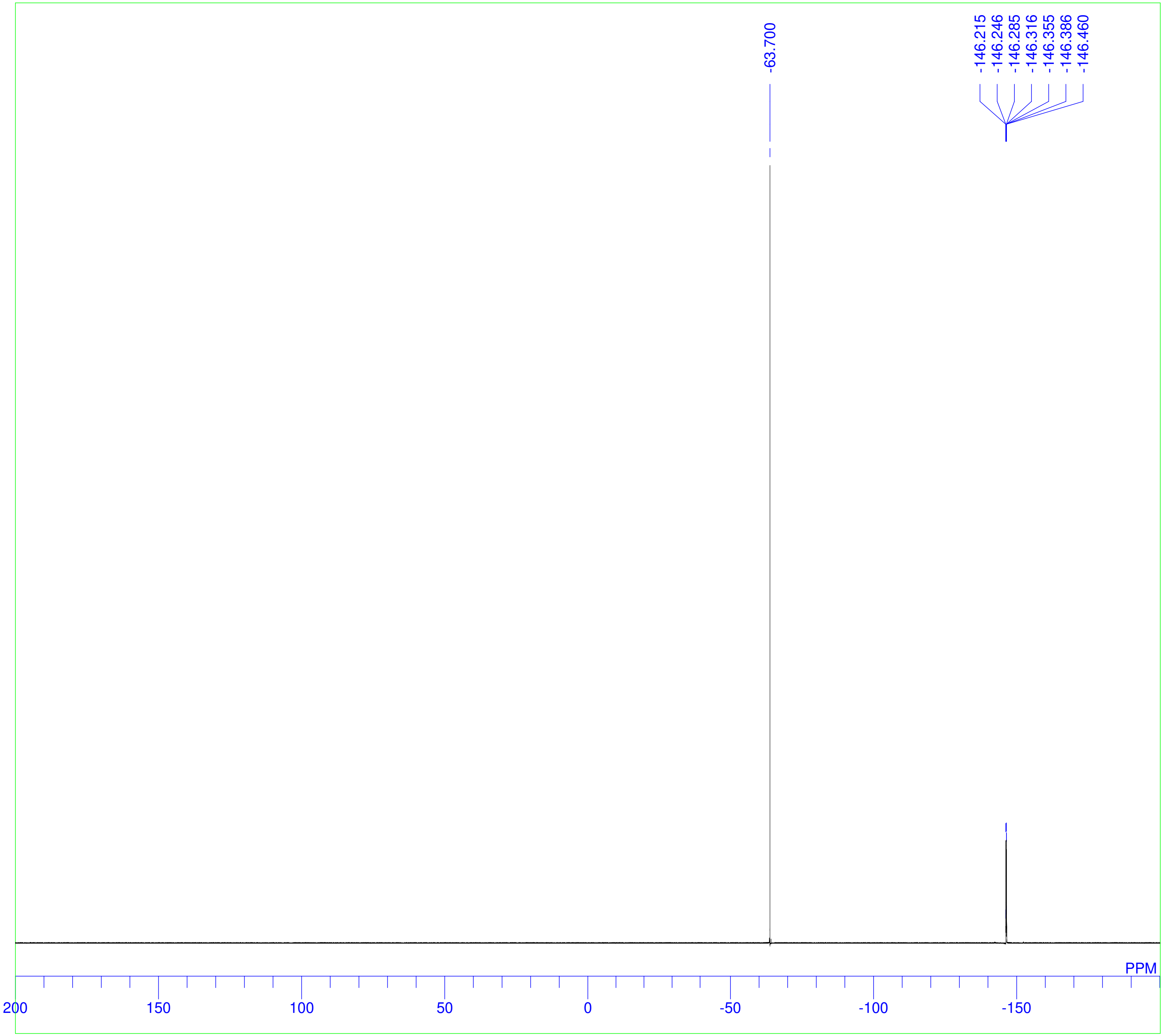

DFILE	BODIPY FL_Fluorine.als
COMNT	
DATIM	2024-06-10 16:23:23
OBNUC	19F
EXMOD	proton.jxp
OBFRQ	470.62 MHz
OBSET	0.46 KHz
OBFIN	0.84 Hz
POINT	104857
FREQU	192307.69 Hz
SCANS	128
ACQTM	0.5453 sec
PD	5.0000 sec
PW1	4.25 usec
IRNUC	19F
CTEMP	24.0 c
SLVNT	CDCL3
EXREF	-63.70 ppm
BF	0.30 Hz
RGAIN	44

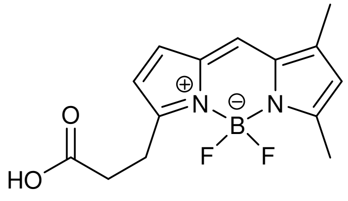

BODIPY FL

Trifluoromethylbenzene as an internal standard

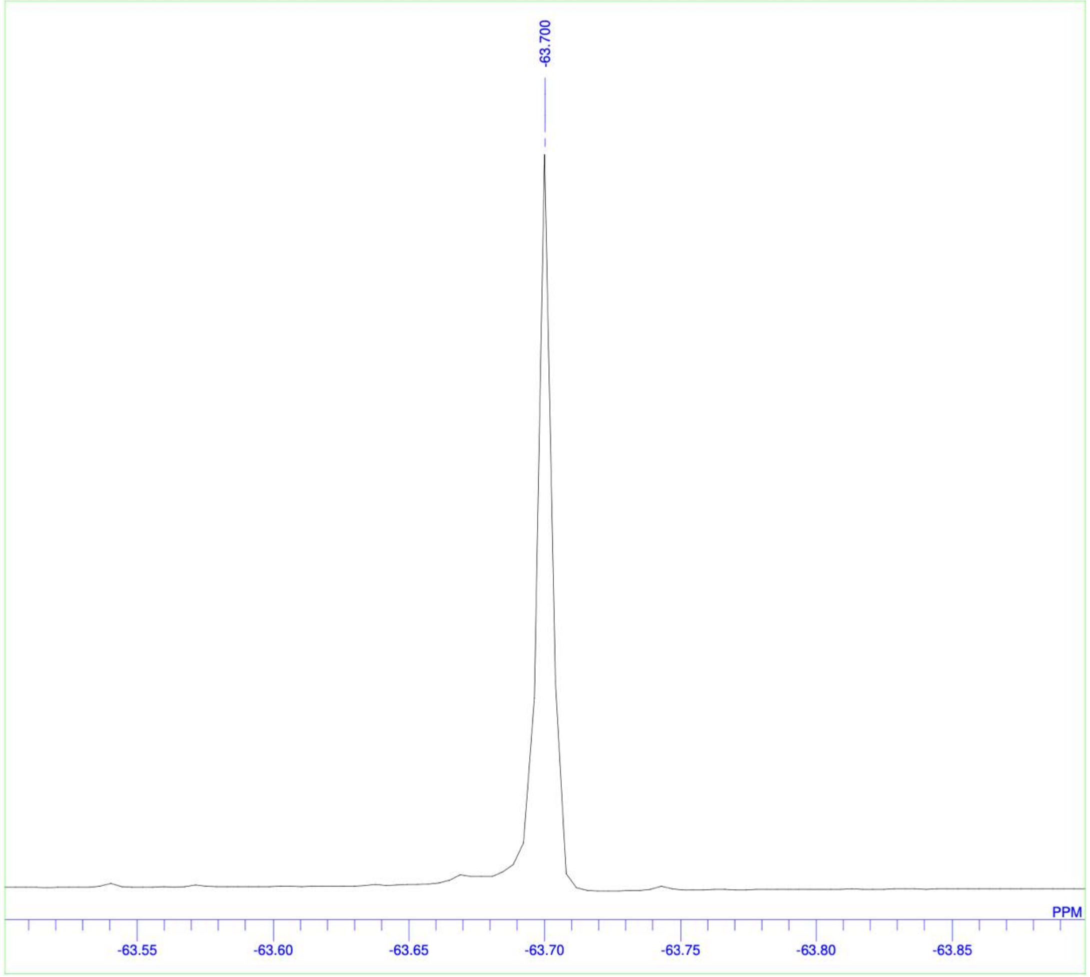

Fluorine of **BODIPY FL**

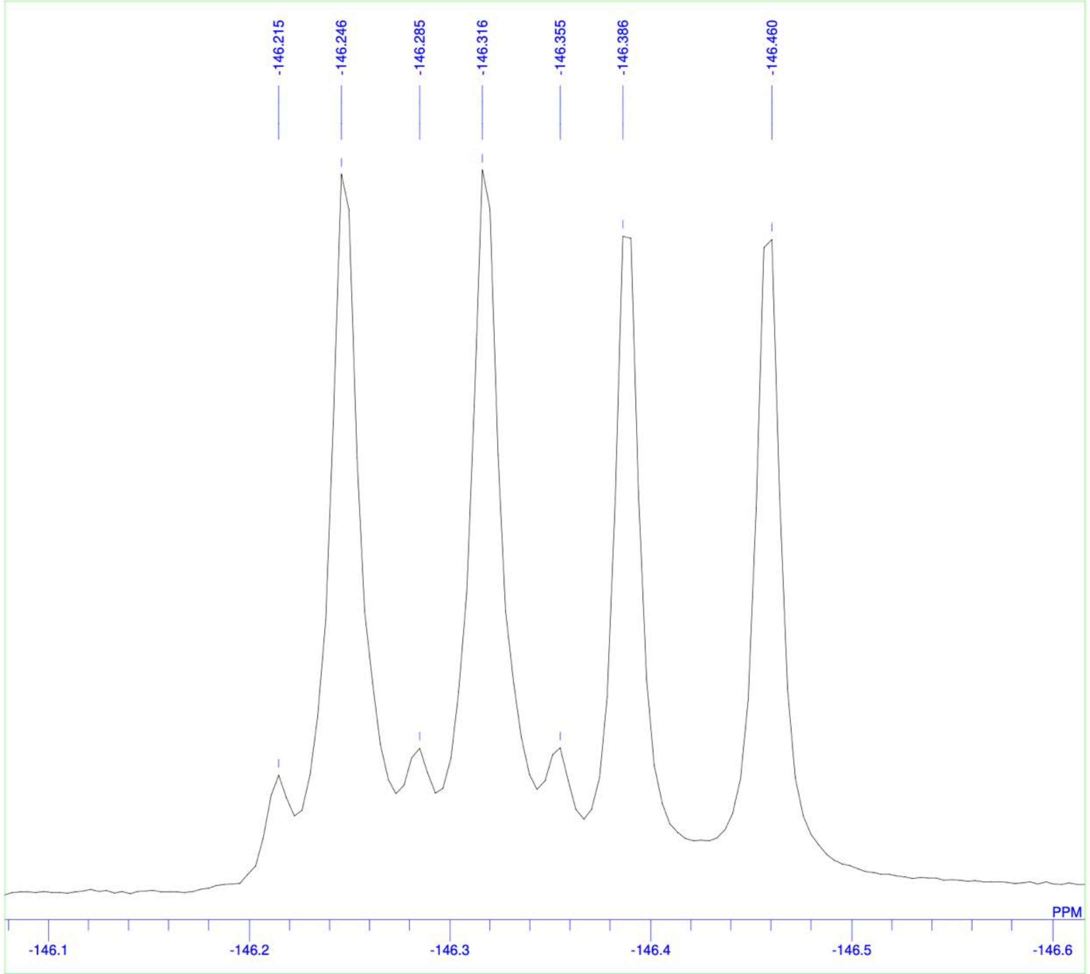

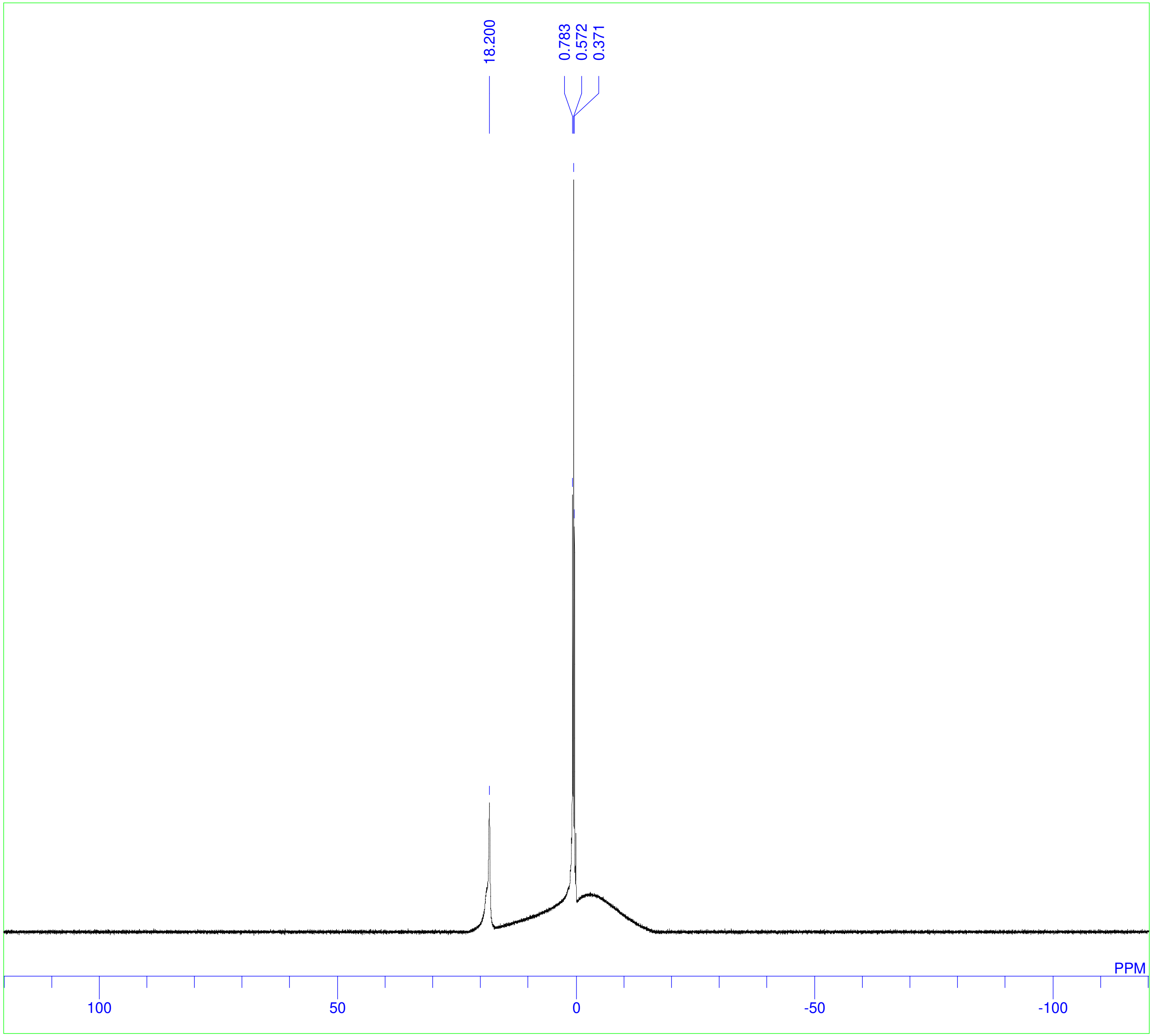

DFILE	BODIPY FL_11Boron.als
COMNT	
DATIM	2024-06-15 17:29:57
OBNUC	11B
EXMOD	proton.jxp
OBFRQ	160.47 MHz
OBSET	1.32 KHz
OBFIN	6.52 Hz
POINT	26214
FREQU	40322.58 Hz
SCANS	256
ACQTM	0.6501 sec
PD	5.0000 sec
PW1	6.55 usec
IRNUC	11B
CTEMP	24.1 c
SLVNT	CDCL3
EXREF	18.20 ppm
BF	0.30 Hz
RGAIN	40

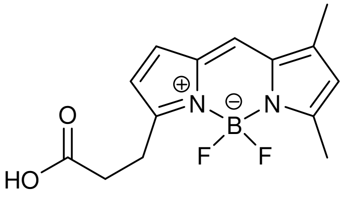

BODIPY FL

B$(OMe)_3$ as an internal standard

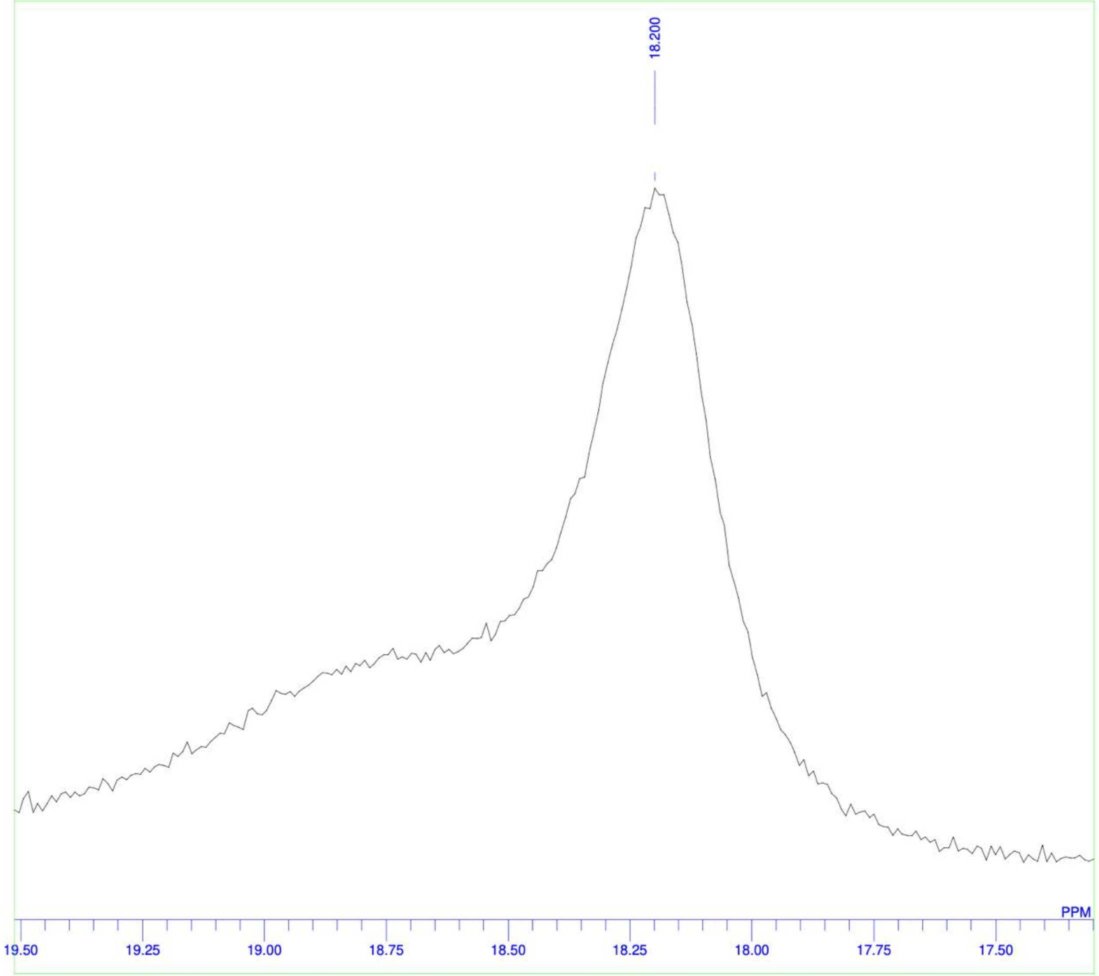

Boron of **BODIPY FL**

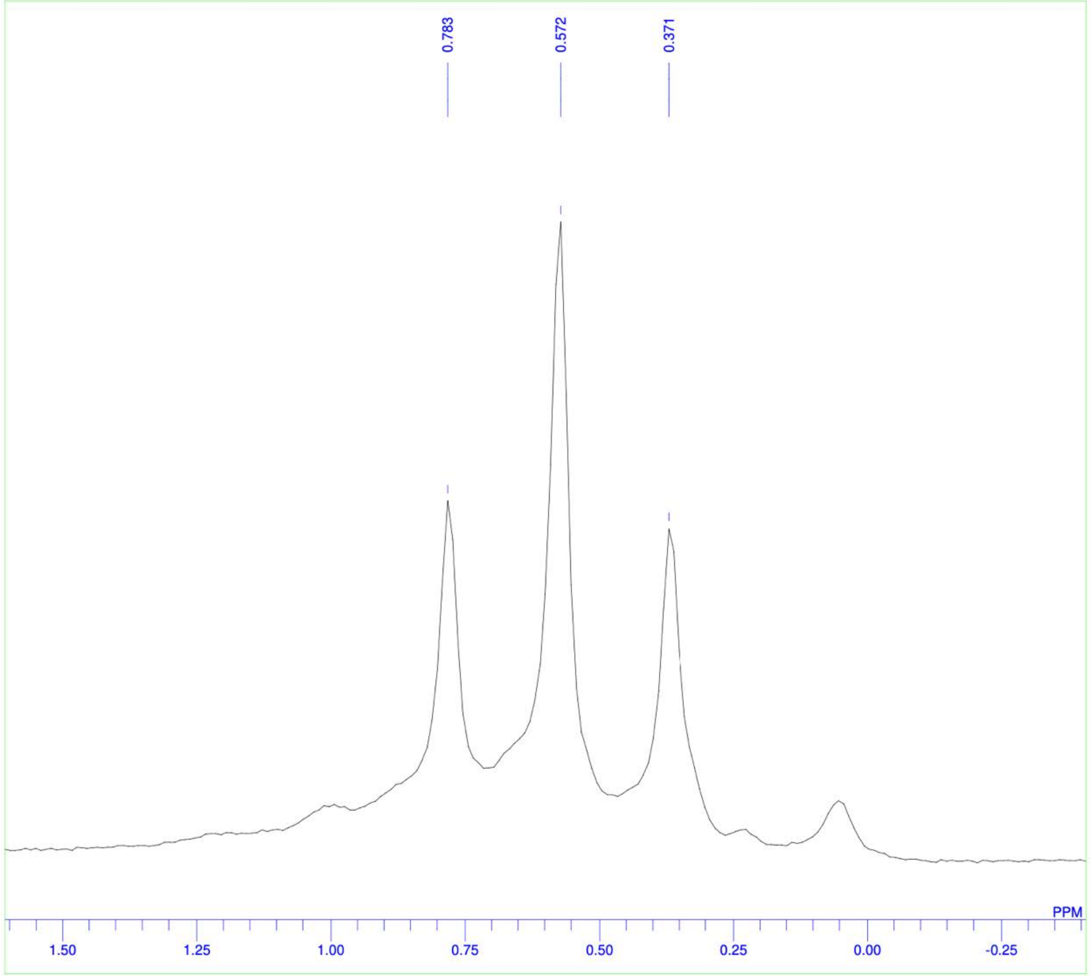

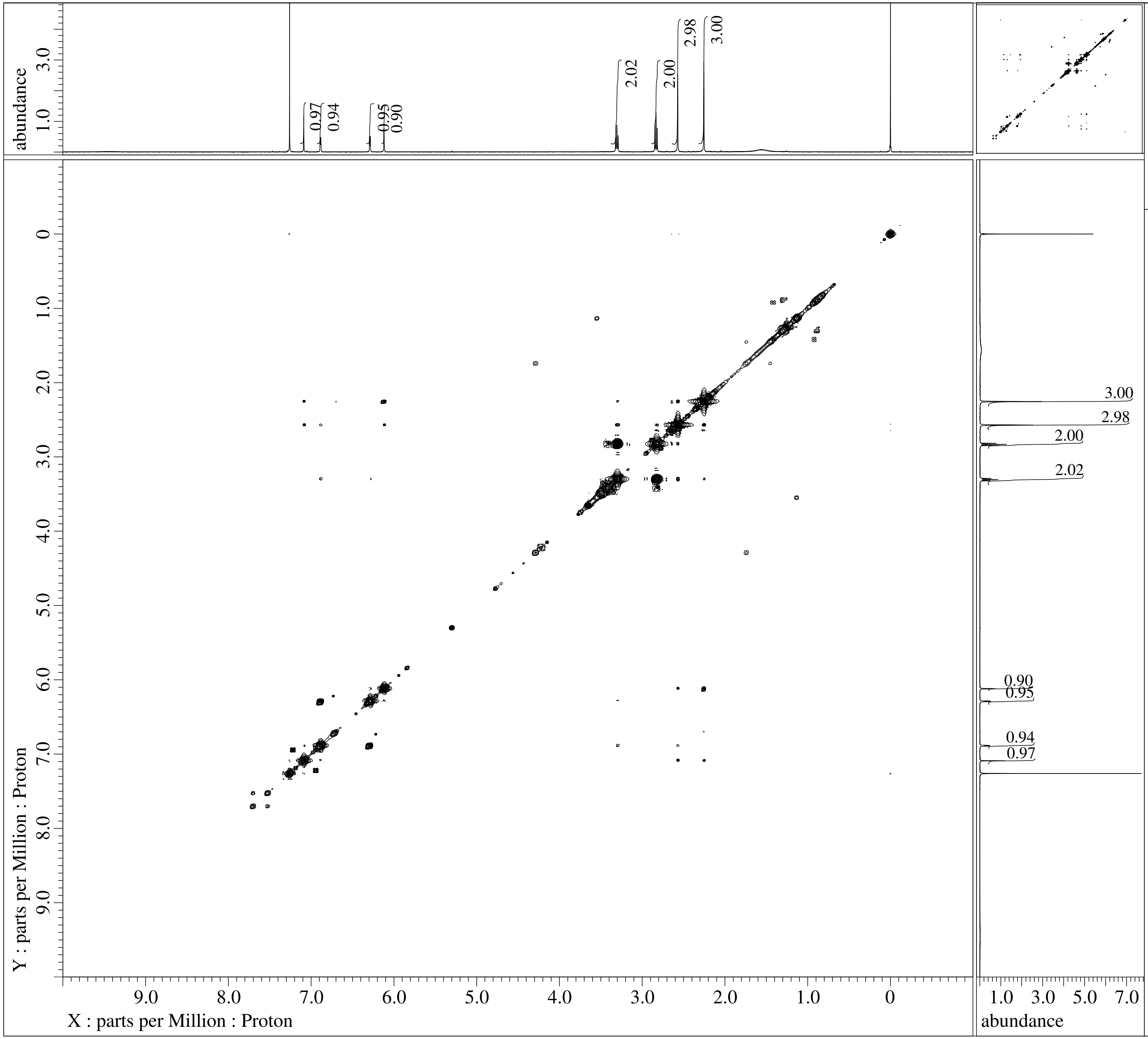

---- PROCESSING PARAMETERS ----
dc_balance(0, FALSE)
sexp(0.2[Hz], 0.0[s])
trapezoid(0[%], 0[%], 80[%], 100[%])
zerofill(1)
fft(1, TRUE, TRUE)
machinephase
ppm

Filename = BODIPY FL_COSY-1-4.jdf
Author = delta
Experiment = cosy.jxp
Sample_Id = 5-BODIPY
Solvent = CHLOROFORM-D
Creation_Time = 21-MAY-2024 22:35:00
Revision_Time = 17-JUN-2024 18:46:53
Current_Time = 17-JUN-2024 18:48:19

Comment = gradient absolute value co
Data_Format = 2D REAL REAL
Dim_Size = 1024, 1024
Dim_Title = Proton Proton
Dim_Units = [ppm] [ppm]
Dimensions = X Y
Site = JNM-ECA500II
Spectrometer = DELTA2_NMR

Field_Strength = 11.7473579[T] (500[MHz])
X_Acq_Duration = 0.2085888[s]
X_Domain = 1H
X_Freq = 500.15991521[MHz]
X_Offset = 3.75231716[ppm]
X_Points = 1280
X_Prescans = 4
X_Resolution = 4.79412126[Hz]
X_Sweep = 6.13647521[kHz]
X_Sweep_Clipped = 4.90918017[kHz]
Y_Domain = 1H
Y_Freq = 500.15991521[MHz]
Y_Offset = 3.75231716[ppm]
Y_Points = 256
Y_Prescans = 0
Y_Resolution = 19.17083824[Hz]
Y_Sweep = 4.90773459[kHz]
Irr_Domain = Proton
Irr_Freq = 500.15991521[MHz]
Irr_Offset = 5.0[ppm]
Tri_Domain = Proton
Tri_Freq = 500.15991521[MHz]
Tri_Offset = 5.0[ppm]
Clipped = FALSE
Scans = 16
Total_Scans = 4096

Relaxation_Delay = 1.5[s]
Recvr_Gain = 40
Temp_Get = 23.7[dC]
X_90_Width = 7.59[us]

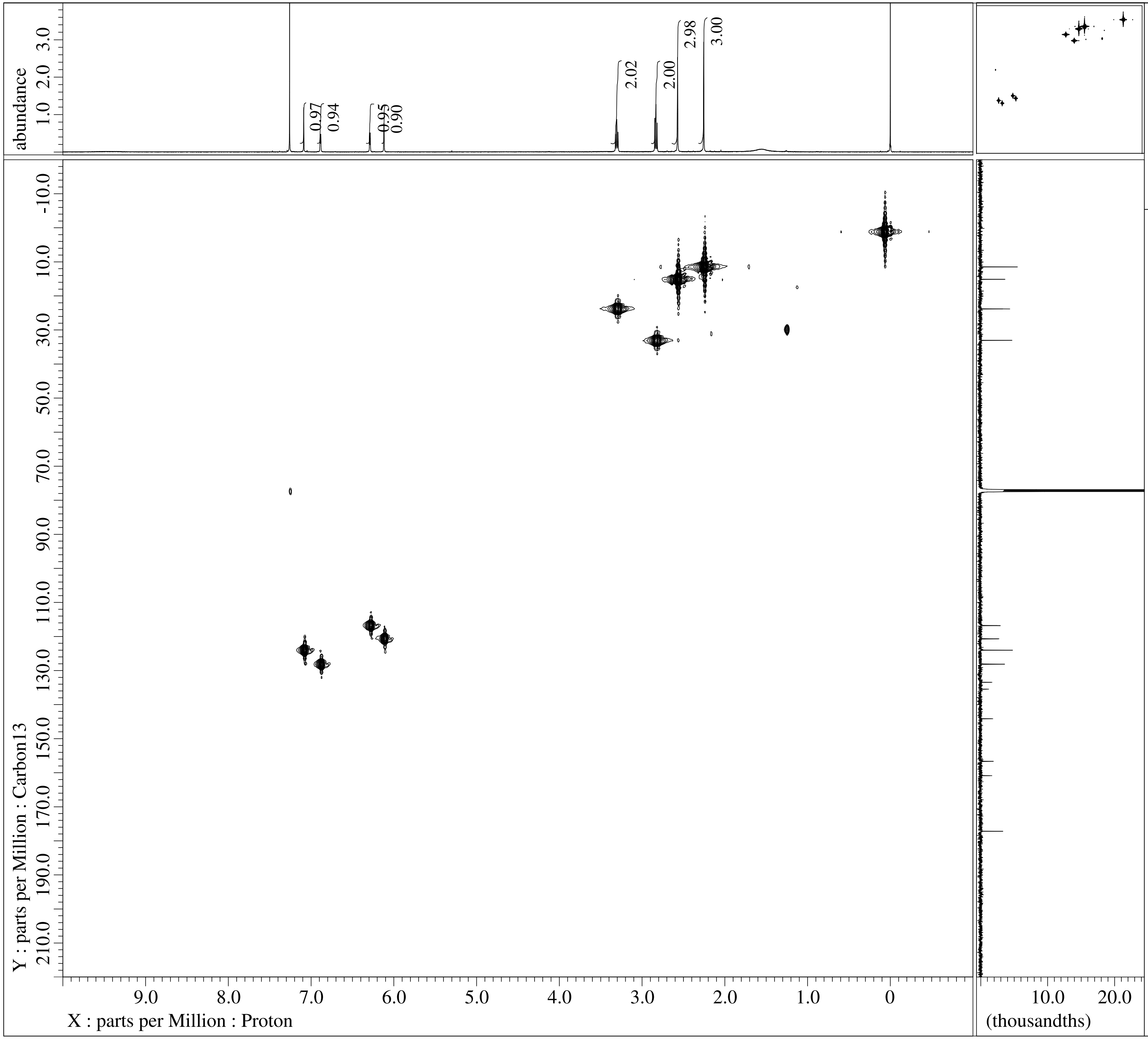

---- PROCESSING PARAMETERS ----
dc_balance(0, FALSE)
sexp(2.0[Hz], 0.0[s])
trapezoid(0[%], 0[%], 80[%], 100[%])
zerofill(1, TRUE)
fft(1, TRUE, TRUE)
machinephase
ppm

Filename = BODIPY FL_HMQC-1-4.jdf
Author = delta
Experiment = hmqc.jxp
Sample_Id = BODIPY FL 13C
Solvent = CHLOROFORM-D
Creation_Time = 15-MAY-2024 04:26:25
Revision_Time = 17-JUN-2024 18:42:20
Current_Time = 17-JUN-2024 18:43:17

Comment = gradient enhanced HMQC
Data_Format = 2D REAL REAL
Dim_Size = 819, 512
Dim_Title = Proton Carbon13
Dim_Units = [ppm] [ppm]
Dimensions = X Y
Site = JNM-ECA500II
Spectrometer = DELTA2_NMR

Field_Strength = 11.7473579[T] (500[MHz]
X_Acq_Duration = 0.17293312[s]
X_Domain = 1H
X_Freq = 500.15991521[MHz]
X_Offset = 3.57710333[ppm]
X_Points = 1024
X_Prescans = 4
X_Resolution = 5.78258231[Hz]
X_Sweep = 5.92136428[kHz]
X_Sweep_Clipped = 4.73709143[kHz]
Y_Domain = 13C
Y_Freq = 125.76529768[MHz]
Y_Offset = 89.59368697[ppm]
Y_Points = 256
Y_Prescans = 0
Y_Resolution = 112.76703233[Hz]
Y_Sweep = 28.86836028[kHz]
Tri_Domain = Proton
Tri_Freq = 500.15991521[MHz]
Tri_Offset = 5.0[ppm]
Clipped = FALSE
Scans = 32
Total_Scans = 8192

Relaxation_Delay = 1.5[s]
Recvr_Gain = 50
Temp_Get = 23.8[dC]
X_Acq_Time = 0.17293312[s]
X_Atn = 3.2[dB]
X_Gamma = 42576375
X_Pulse = 7.59[us]

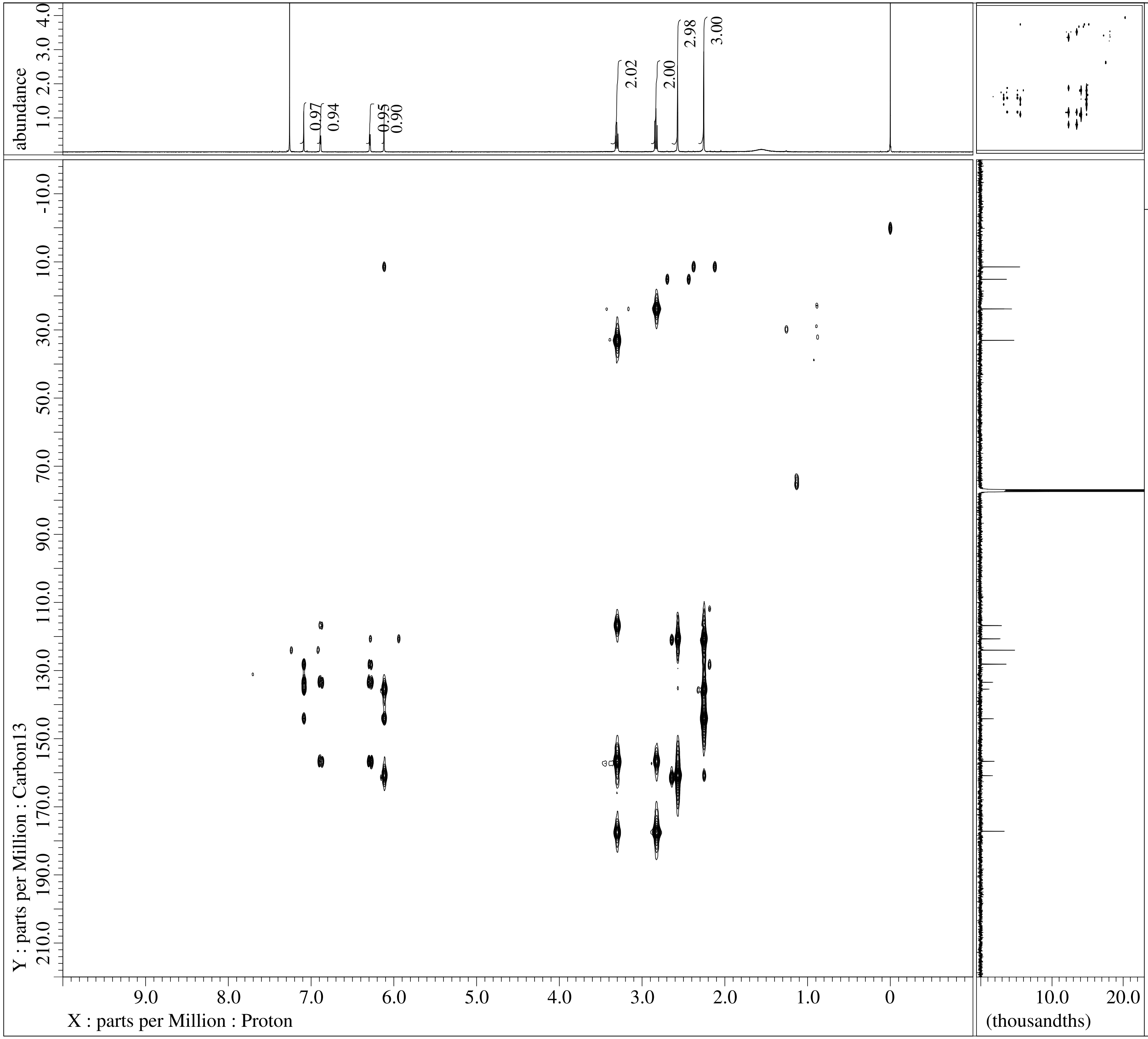


```
---- PROCESSING PARAMETERS ----
dc_balance( 0, FALSE )
sexp( 2.0[Hz], 0.0[s] )
trapezoid( 0[%], 0[%], 80[%], 100[%] )
zerofill( 1, TRUE )
fft( 1, TRUE, TRUE )
machinephase
ppm
```

```
Filename            = BODIPY FL_HMBC-1-4.jdf
Author              = delta
Experiment          = hmbc.jxp
Sample_Id           = 5-BODIPY
Solvent             = CHLOROFORM-D
Creation_Time       = 22-MAY-2024 00:34:09
Revision_Time       = 17-JUN-2024 18:44:50
Current_Time        = 17-JUN-2024 18:45:52

Comment             = gradient enhanced HMBC
Data_Format         = 2D REAL REAL
Dim_Size            = 1638, 512
Dim_Title           = Proton Carbon13
Dim_Units           = [ppm] [ppm]
Dimensions          = X Y
Site                = JNM-ECA500II
Spectrometer        = DELTA2_NMR

Field_Strength      = 11.7473579[T] (500[MHz]
X_Acq_Duration      = 0.33374208[s]
X_Domain            = 1H
X_Freq              = 500.15991521[MHz]
X_Offset            = 3.75231716[ppm]
X_Points            = 2048
X_Prescans          = 4
X_Resolution        = 2.99632579[Hz]
X_Sweep             = 6.13647521[kHz]
X_Sweep_Clipped     = 4.90918017[kHz]
Y_Domain            = 13C
Y_Freq              = 125.76529768[MHz]
Y_Offset            = 100[ppm]
Y_Points            = 256
Y_Prescans          = 0
Y_Resolution        = 122.99275819[Hz]
Y_Sweep             = 31.4861461[kHz]
Tri_Domain          = Proton
Tri_Freq            = 500.15991521[MHz]
Tri_Offset          = 5.0[ppm]
Clipped             = FALSE
Scans               = 64
Total_Scans         = 16384

Relaxation_Delay    = 1.5[s]
Recvr_Gain          = 50
Temp_Get            = 24[dC]
X_Acq_Time          = 0.33374208[s]
X_Atn               = 3.2[dB]
X_Gamma             = 42576375
X_Pulse             = 7.59[us]
```

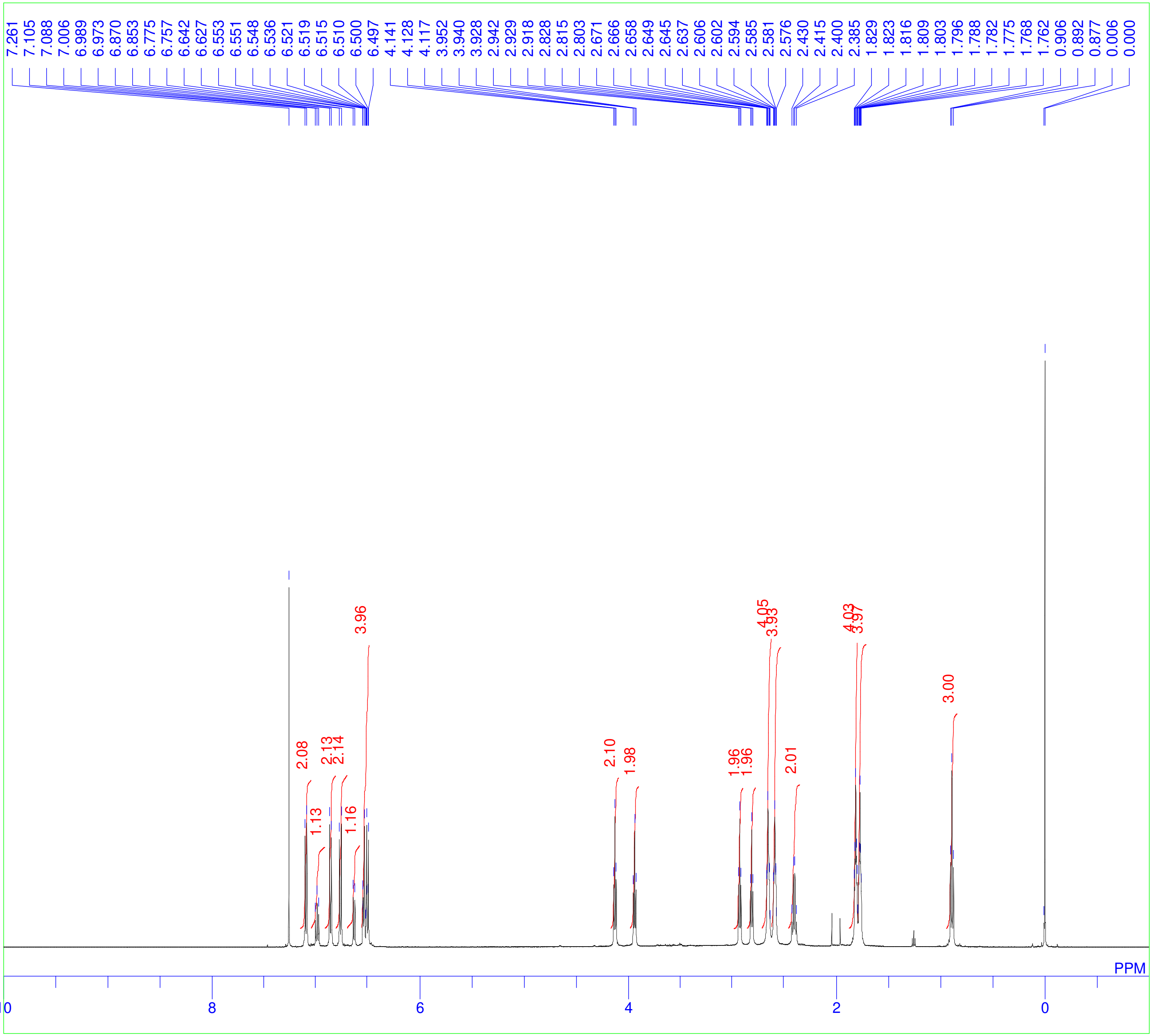

DFILE	RID-B-OH_Proton.als
COMNT	
DATIM	2024-05-10 19:10:21
OBNUC	1H
EXMOD	proton.jxp
OBFRQ	500.16 MHz
OBSET	2.41 KHz
OBFIN	6.01 Hz
POINT	13107
FREQU	7507.51 Hz
SCANS	8
ACQTM	1.7459 sec
PD	5.0000 sec
PW1	3.80 usec
IRNUC	1H
CTEMP	24.3 c
SLVNT	CDCL3
EXREF	0.00 ppm
BF	0.25 Hz
RGAIN	42

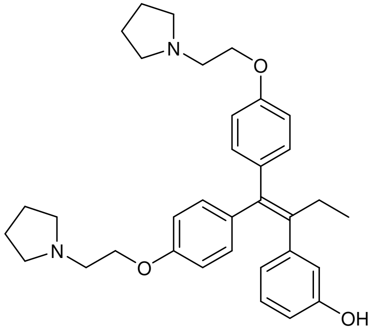

RID-B-OH

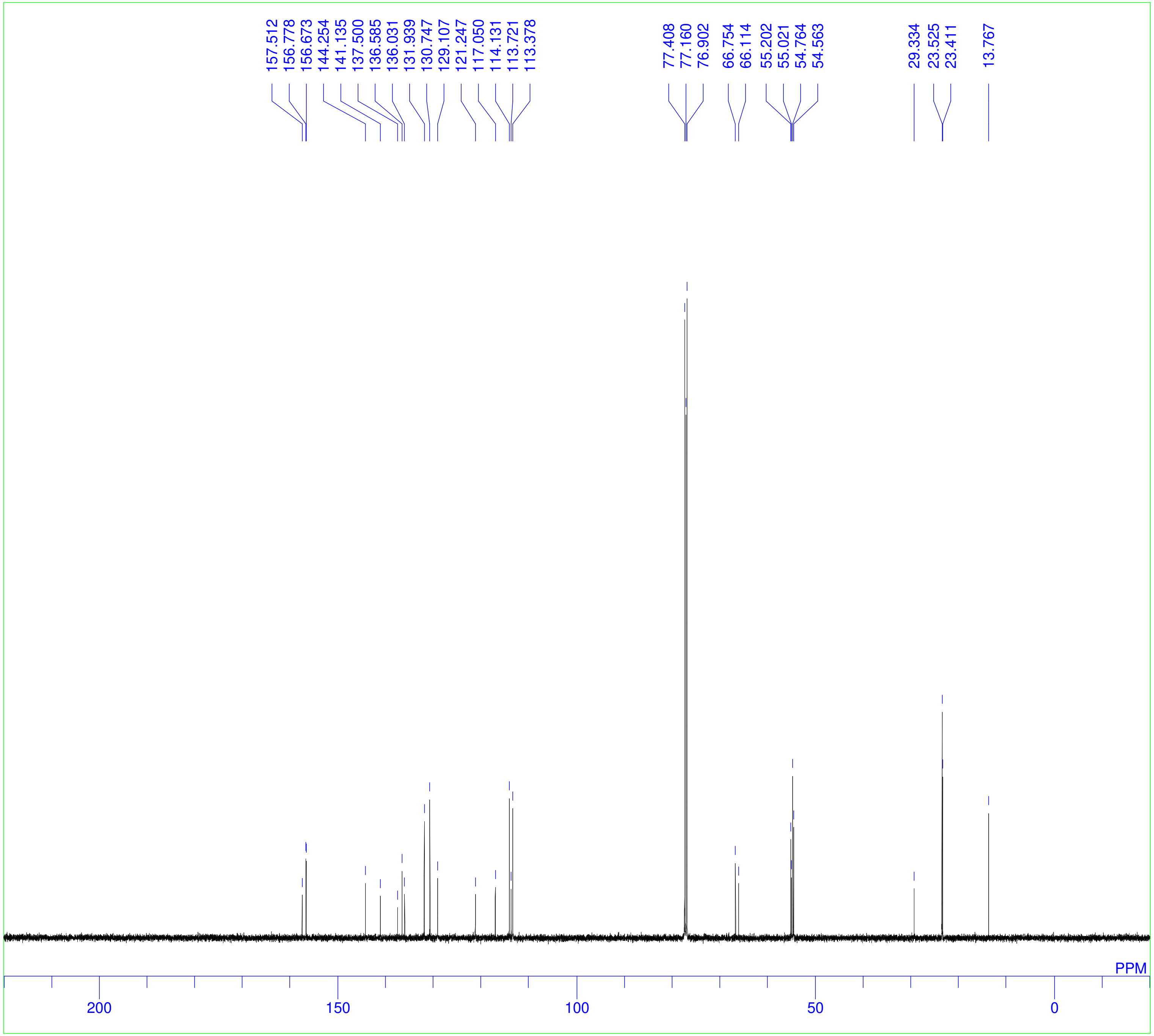

DFILE RID-B-OH_Carbon.als
COMNT
DATIM 2024-05-15 20:12:53
OBNUC 13C
EXMOD carbon.jxp
OBFRQ 125.77 MHz
OBSET 7.87 KHz
OBFIN 4.21 Hz
POINT 26214
FREQU 31446.54 Hz
SCANS 1024
ACQTM 0.8336 sec
PD 2.0000 sec
PW1 4.30 usec
IRNUC 1H
CTEMP 23.8 c
SLVNT CDCL3
EXREF 77.16 ppm
BF 0.25 Hz
RGAIN 36

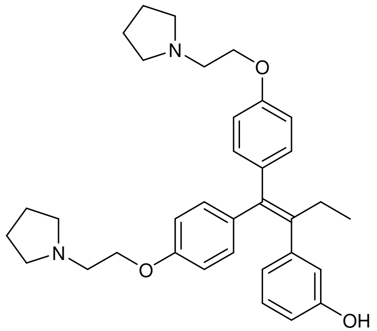

RID-B-OH

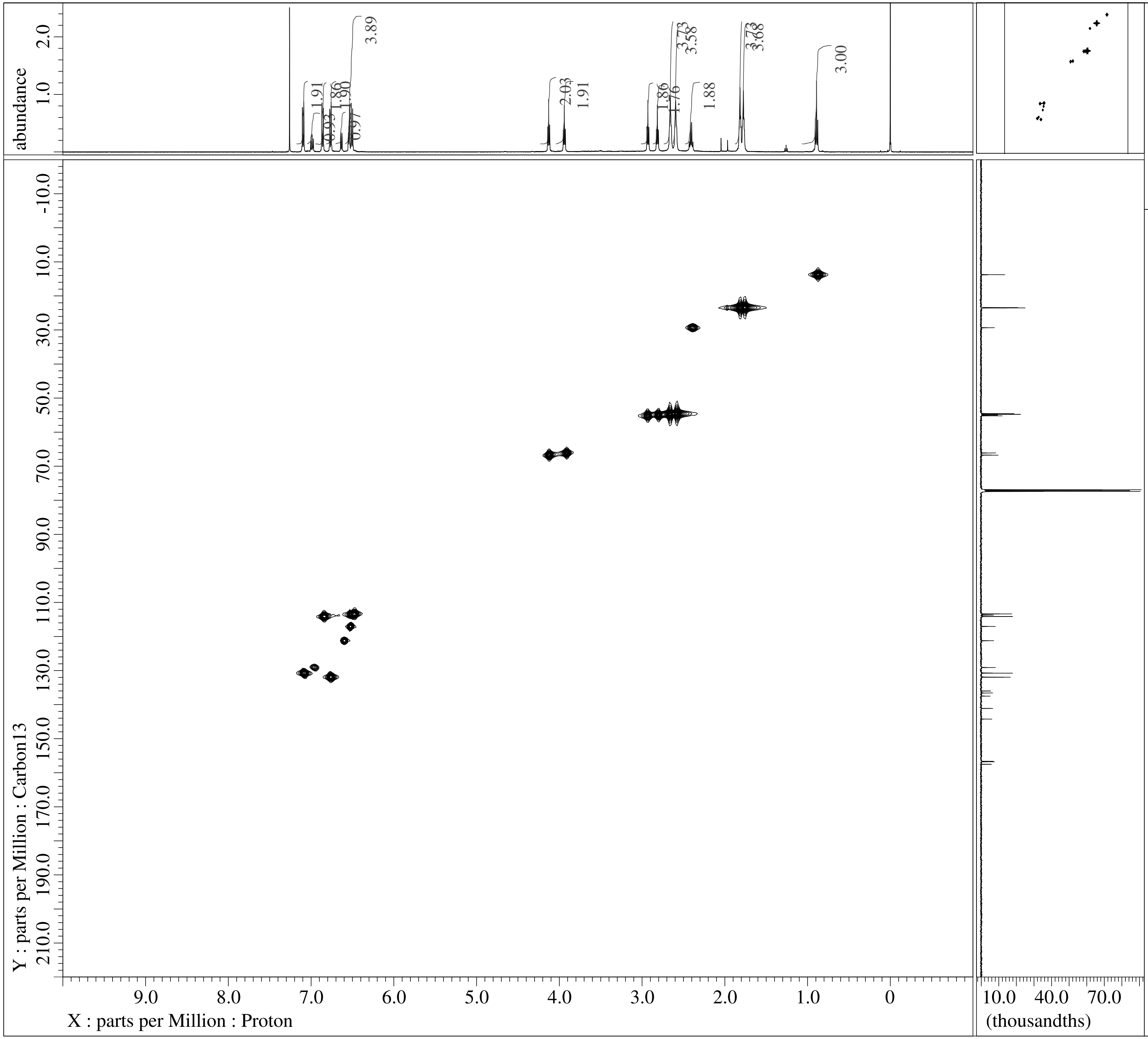

---- PROCESSING PARAMETERS ----
dc_balance(0, FALSE)
sexp(0.2[Hz], 0.0[s])
trapezoid(0[%], 0[%], 80[%], 100[%])
zerofill(1, TRUE)
fft(1, TRUE, TRUE)
machinephase
ppm

Filename	= RID-B-OH_HMQC-1-4.jdf
Author	= delta
Experiment	= hmqc.jxp
Sample_Id	= EM-RID-B-OH
Solvent	= CHLOROFORM-D
Creation_Time	= 24-MAY-2024 14:25:44
Revision_Time	= 17-JUN-2024 19:55:54
Current_Time	= 17-JUN-2024 19:56:47
Comment	= gradient enhanced HMQC
Data_Format	= 2D REAL REAL
Dim_Size	= 819, 512
Dim_Title	= Proton Carbon13
Dim_Units	= [ppm] [ppm]
Dimensions	= X Y
Site	= JNM-ECA500II
Spectrometer	= DELTA2_NMR
Field_Strength	= 11.7473579[T] (500[MHz]
X_Acq_Duration	= 0.10911744[s]
X_Domain	= 1H
X_Freq	= 500.15991521[MHz]
X_Offset	= 5.0[ppm]
X_Points	= 1024
X_Prescans	= 4
X_Resolution	= 9.16443788[Hz]
X_Sweep	= 9.38438438[kHz]
X_Sweep_Clipped	= 7.50750751[kHz]
Y_Domain	= 13C
Y_Freq	= 125.76529768[MHz]
Y_Offset	= 85[ppm]
Y_Points	= 256
Y_Prescans	= 0
Y_Resolution	= 83.60980308[Hz]
Y_Sweep	= 21.40410959[kHz]
Tri_Domain	= Proton
Tri_Freq	= 500.15991521[MHz]
Tri_Offset	= 5.0[ppm]
Clipped	= FALSE
Scans	= 4
Total_Scans	= 1024
Relaxation_Delay	= 1.5[s]
Recvr_Gain	= 50
Temp_Get	= 24.2[dC]
X_Acq_Time	= 0.10911744[s]
X_Atn	= 3.2[dB]
X_Gamma	= 42576375
X_Pulse	= 7.59[us]

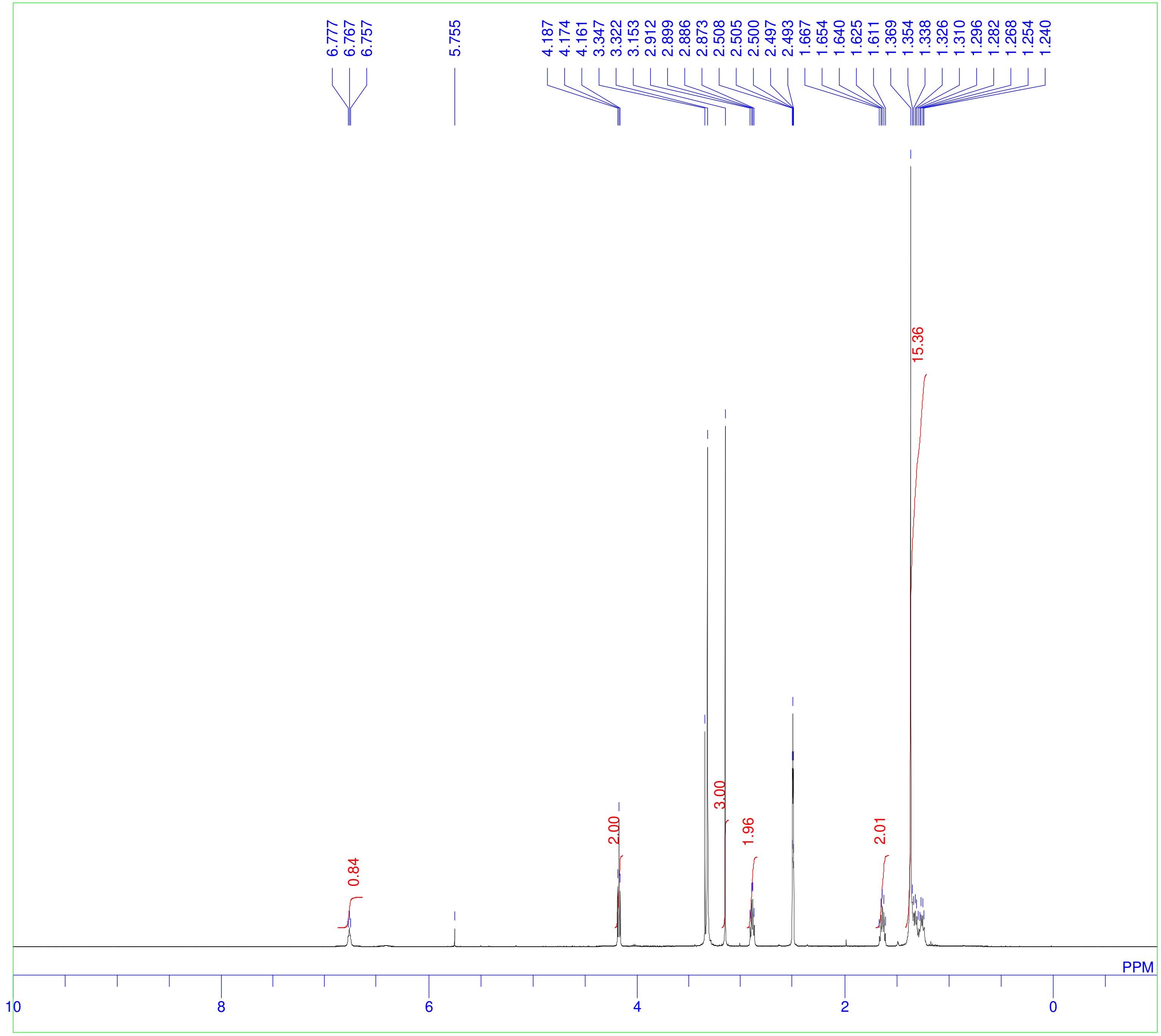

DFILE	S9_Proton.als
COMNT	
DATIM	2024-05-18 10:51:08
OBNUC	1H
EXMOD	proton.jxp
OBFRQ	500.16 MHz
OBSET	2.41 KHz
OBFIN	6.01 Hz
POINT	13107
FREQU	7507.51 Hz
SCANS	8
ACQTM	1.7459 sec
PD	5.0000 sec
PW1	3.80 usec
IRNUC	1H
CTEMP	24.0 c
SLVNT	DMSO
EXREF	2.50 ppm
BF	0.30 Hz
RGAIN	40

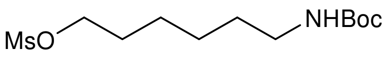

S9

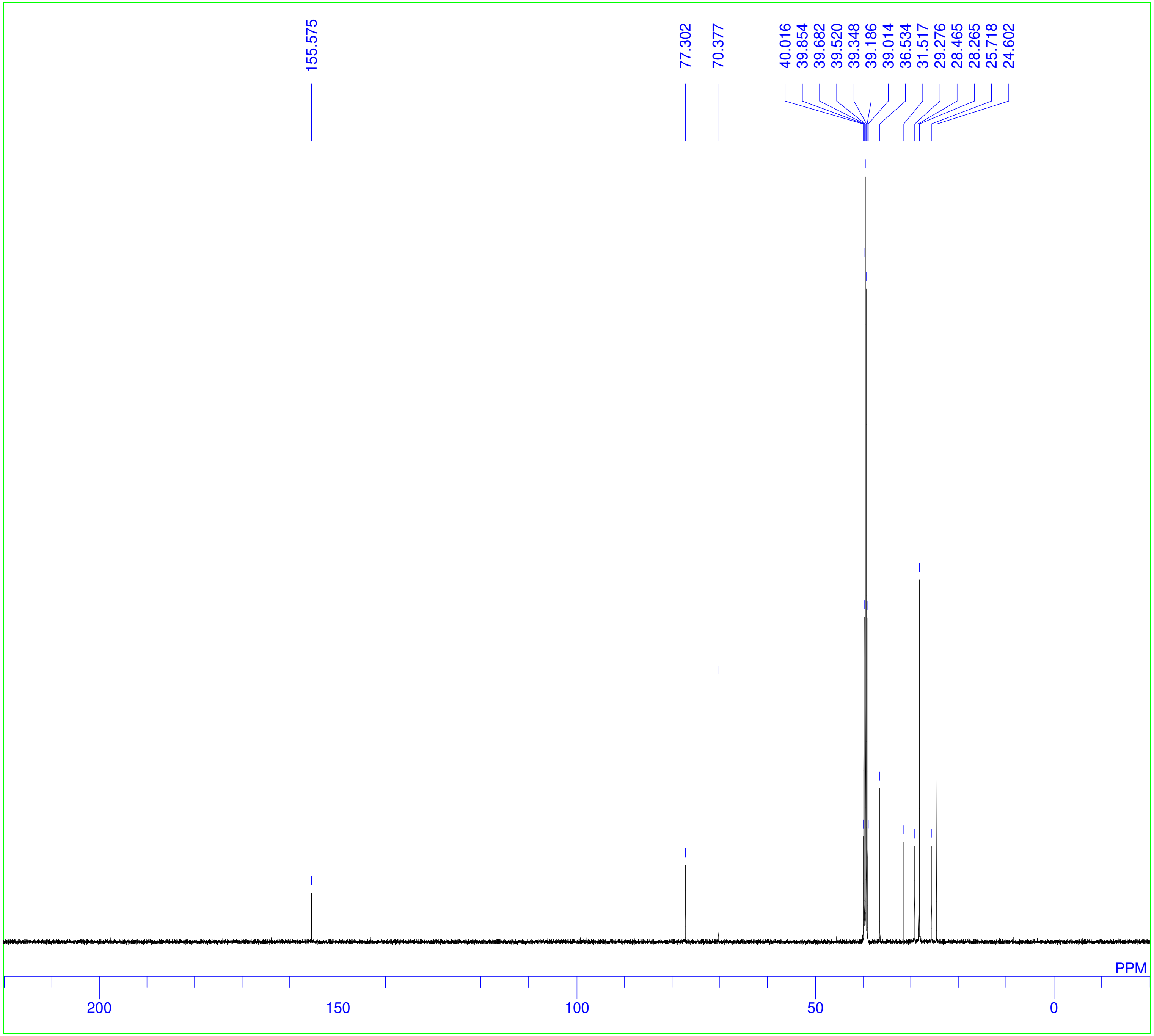

DFILE S9_Carbon.als
COMNT
DATIM 2024-05-21 09:13:50
OBNUC 13C
EXMOD carbon.jxp
OBFRQ 125.77 MHz
OBSET 7.87 KHz
OBFIN 4.21 Hz
POINT 26214
FREQU 31446.54 Hz
SCANS 1024
ACQTM 0.8336 sec
PD 2.0000 sec
PW1 4.30 usec
IRNUC 1H
CTEMP 24.0 c
SLVNT DMSO
EXREF 39.52 ppm
BF 0.30 Hz
RGAIN 30

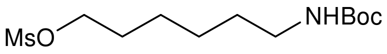

S9

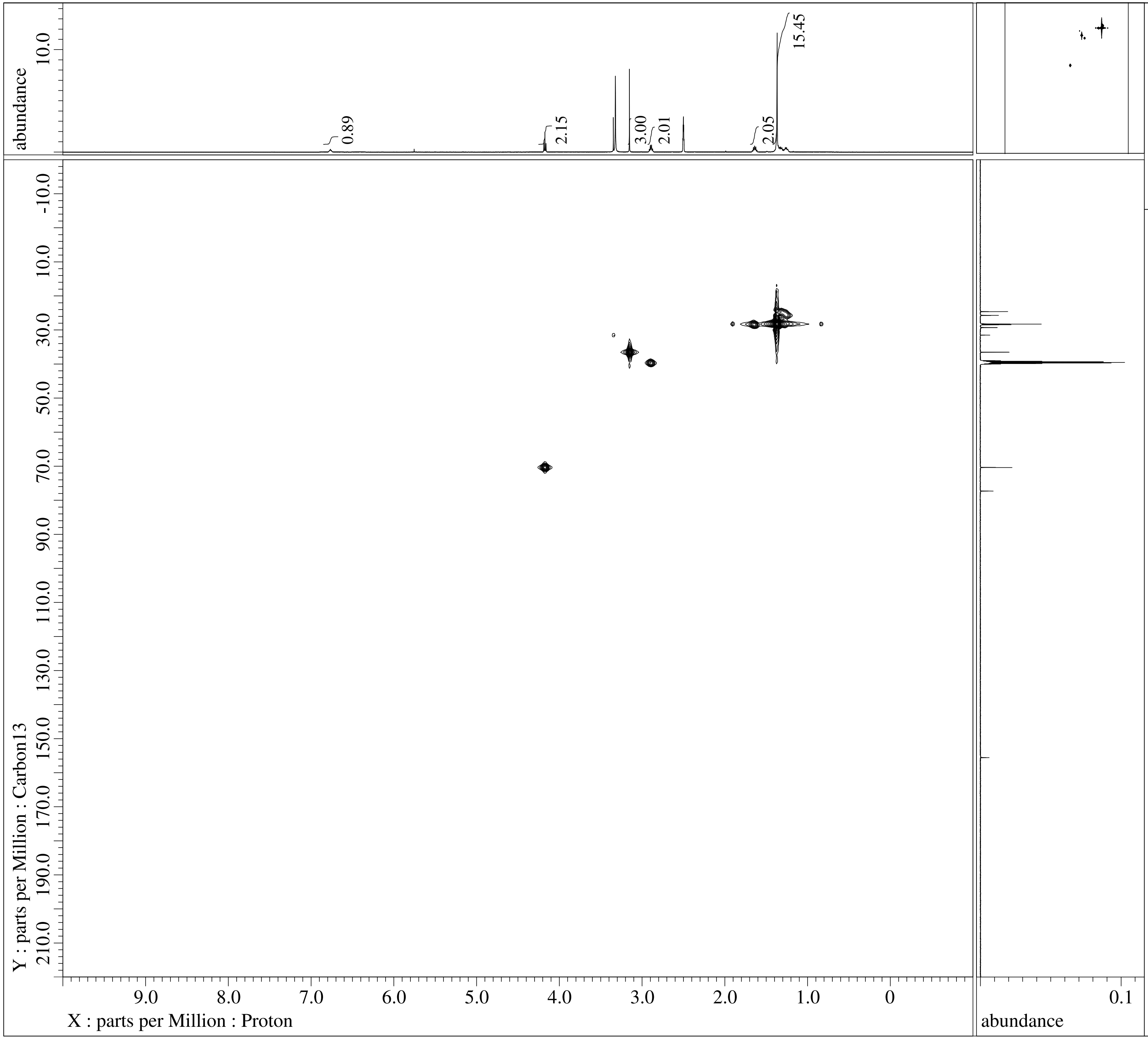


```
---- PROCESSING PARAMETERS ----
dc_balance( 0, FALSE )
sexp( 2.0[Hz], 0.0[s] )
trapezoid( 0[%], 0[%], 80[%], 100[%] )
zerofill( 1 )
fft( 1, TRUE, TRUE )
machinephase
ppm

Filename            = S9_HMQC-1-4.jdf
Author              = delta
Experiment          = hmqc.jxp
Sample_Id           = fs145-5 1
Solvent             = DMSO-D6
Creation_Time       = 21-MAY-2024 14:50:30
Revision_Time       = 17-JUN-2024 18:49:50
Current_Time        = 17-JUN-2024 18:51:18

Comment             = gradient enhanced HMQC
Data_Format         = 2D REAL REAL
Dim_Size            = 819, 512
Dim_Title           = Proton Carbon13
Dim_Units           = [ppm] [ppm]
Dimensions          = X Y
Site                = JNM-ECA500II
Spectrometer        = DELTA2_NMR

Field_Strength      = 11.7473579[T] (500[MHz]
X_Acq_Duration      = 0.10911744[s]
X_Domain            = 1H
X_Freq              = 500.15991521[MHz]
X_Offset            = 5.0[ppm]
X_Points            = 1024
X_Prescans          = 4
X_Resolution        = 9.16443788[Hz]
X_Sweep             = 9.38438438[kHz]
X_Sweep_Clipped     = 7.50750751[kHz]
Y_Domain            = 13C
Y_Freq              = 125.76529768[MHz]
Y_Offset            = 85[ppm]
Y_Points            = 256
Y_Prescans          = 0
Y_Resolution        = 83.60980308[Hz]
Y_Sweep             = 21.40410959[kHz]
Tri_Domain          = Proton
Tri_Freq            = 500.15991521[MHz]
Tri_Offset          = 5.0[ppm]
Clipped             = FALSE
Scans               = 4
Total_Scans         = 1024

Relaxation_Delay    = 1.5[s]
Recvr_Gain          = 50
Temp_Get            = 23.9[dC]
X_Acq_Time          = 0.10911744[s]
X_Atn               = 3.2[dB]
X_Gamma             = 42576375
X_Pulse             = 7.59[us]
```

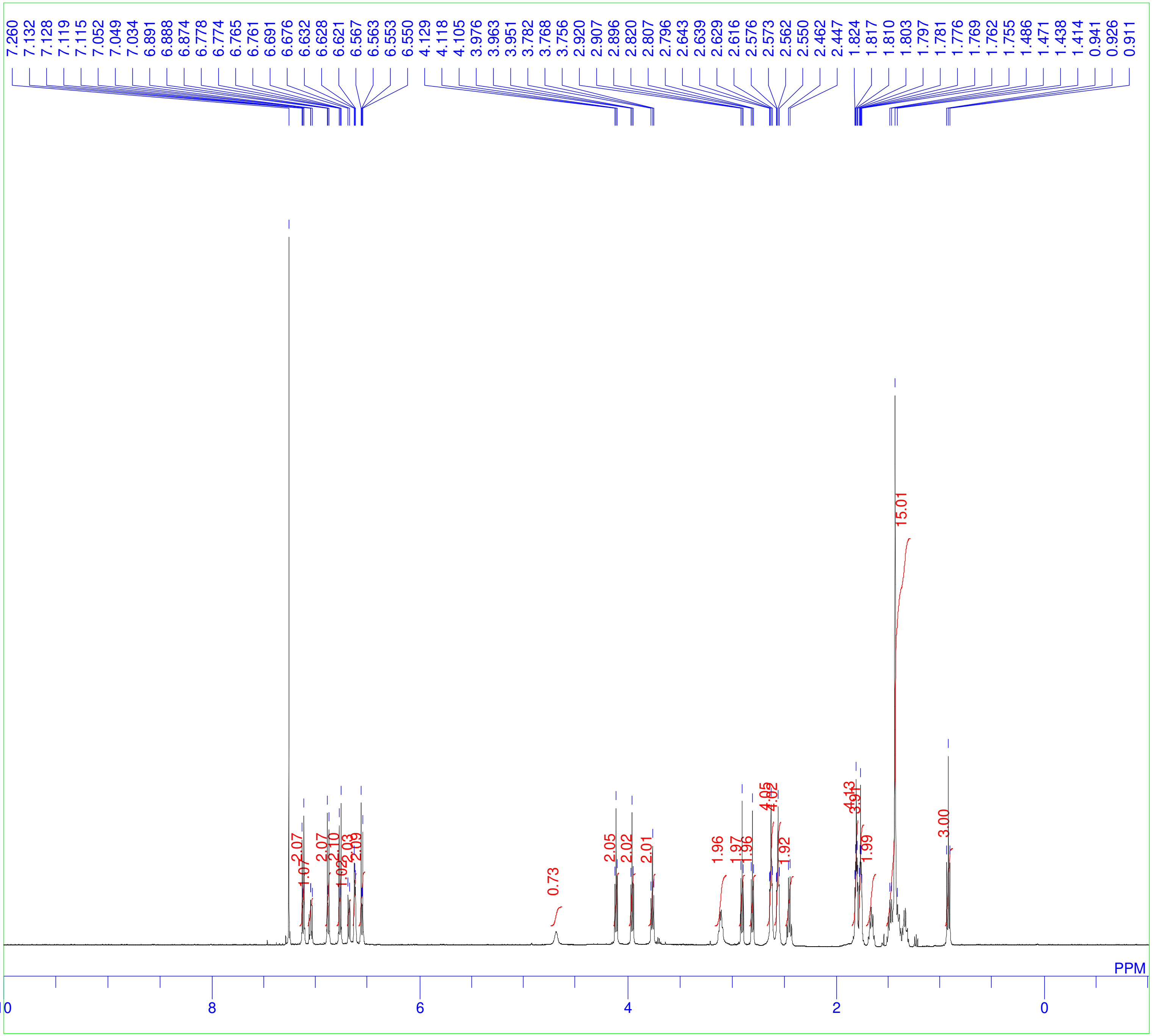

DFILE	1_proton.als
COMNT	
DATIM	2020-01-29 15:28:00
OBNUC	1H
EXMOD	proton.jxp
OBFRQ	500.16 MHz
OBSET	2.41 KHz
OBFIN	6.01 Hz
POINT	13107
FREQU	7507.51 Hz
SCANS	8
ACQTM	1.7459 sec
PD	5.0000 sec
PW1	3.84 usec
IRNUC	1H
CTEMP	21.3 c
SLVNT	CDCL3
EXREF	7.26 ppm
BF	0.12 Hz
RGAIN	34

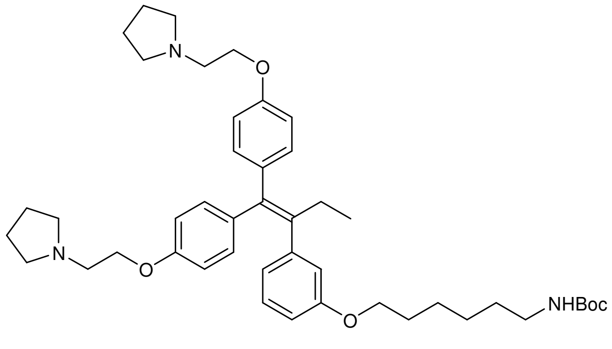

1

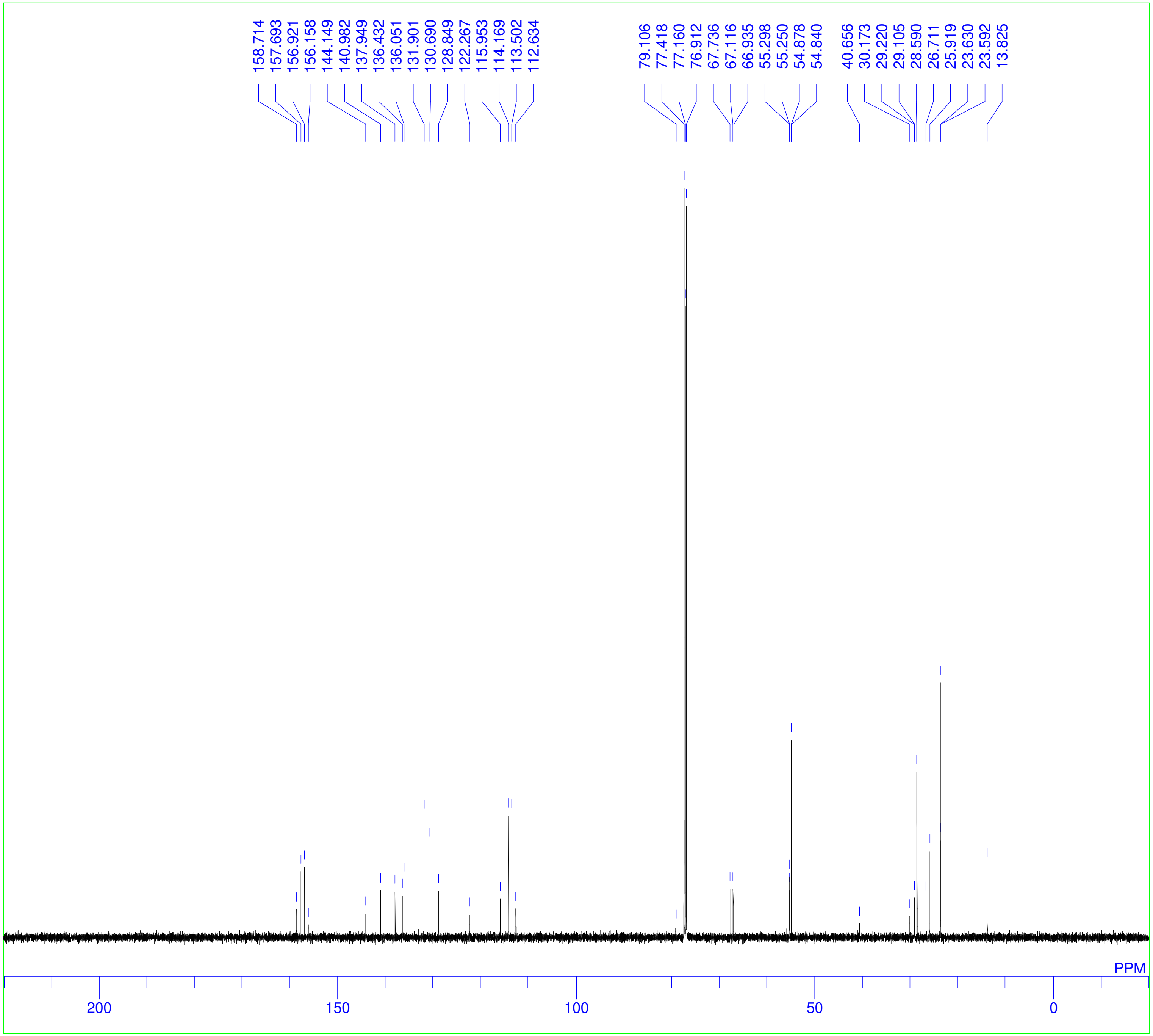

DFILE	1_carbon.als
COMNT	
DATIM	2020-01-29 15:29:50
OBNUC	13C
EXMOD	carbon.jxp
OBFRQ	125.77 MHz
OBSET	7.87 KHz
OBFIN	4.21 Hz
POINT	26214
FREQU	31446.54 Hz
SCANS	1024
ACQTM	0.8336 sec
PD	2.0000 sec
PW1	3.87 usec
IRNUC	1H
CTEMP	21.5 c
SLVNT	CDCL3
EXREF	77.16 ppm
BF	0.12 Hz
RGAIN	24

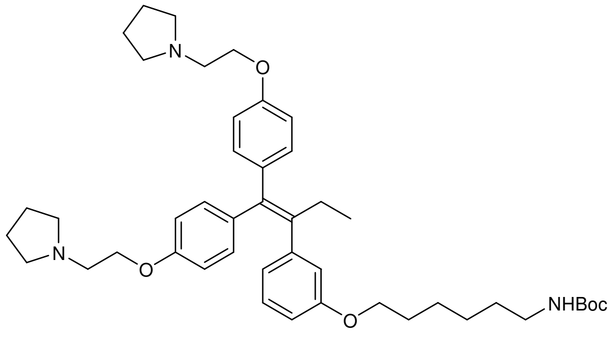

1

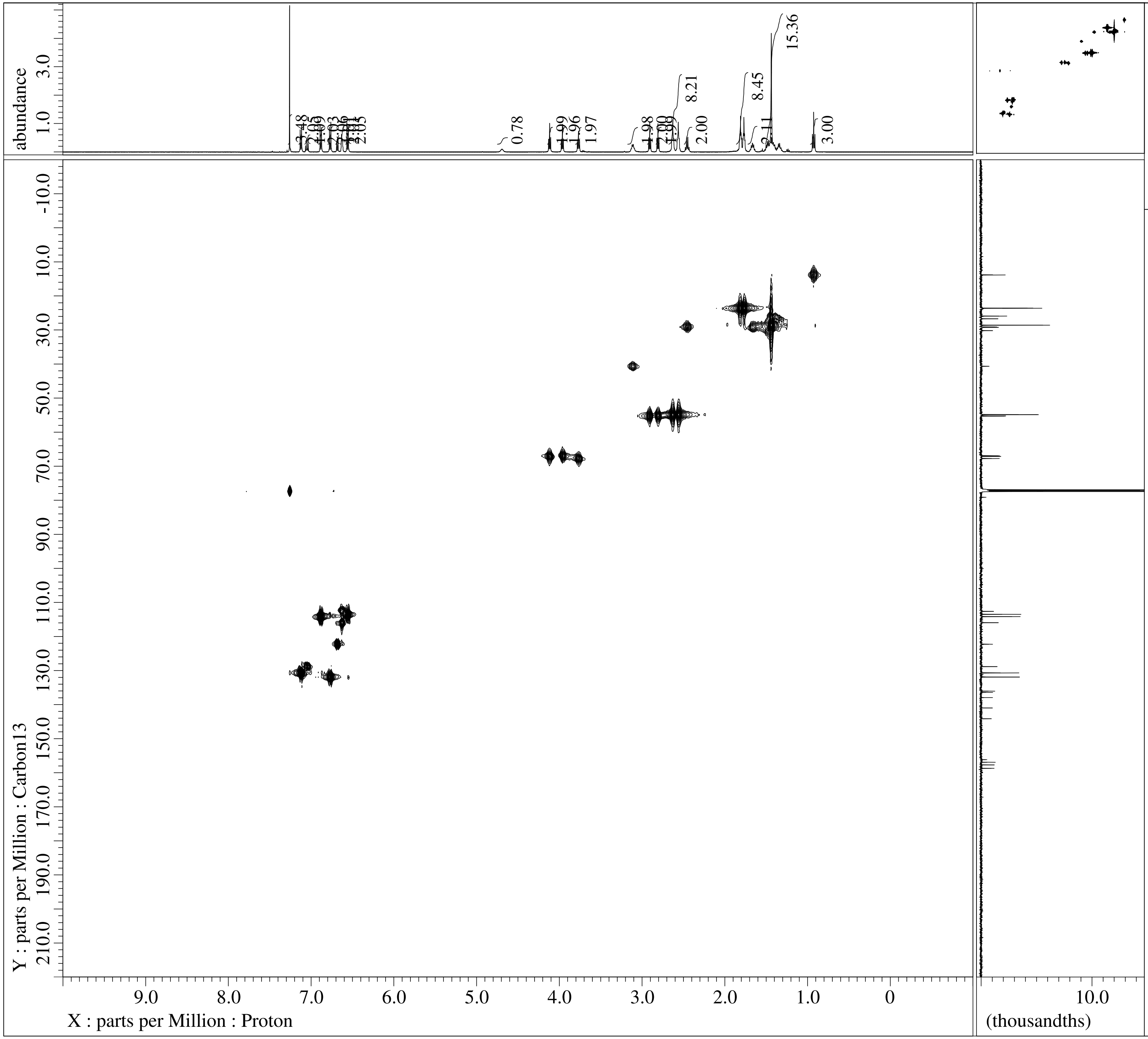


```
---- PROCESSING PARAMETERS ----
dc_balance( 0, FALSE )
sexp( 2.0[Hz], 0.0[s] )
trapezoid( 0[%], 0[%], 80[%], 100[%] )
zerofill( 1 )
fft( 1, TRUE, TRUE )
machinephase
ppm

Filename            = 1_HMQC-1-4.jdf
Author              = delta
Experiment          = hmqc.jxp
Sample_Id           = TK-2-147 data
Solvent             = CHLOROFORM-D
Creation_Time       = 29-JAN-2020 16:18:43
Revision_Time       = 17-JUN-2024 20:03:36
Current_Time        = 17-JUN-2024 20:04:41

Comment             = gradient enhanced HMQC
Data_Format         = 2D REAL REAL
Dim_Size            = 819, 512
Dim_Title           = Proton Carbon13
Dim_Units           = [ppm] [ppm]
Dimensions          = X Y
Site                = JNM-ECA500II
Spectrometer        = DELTA2_NMR

Field_Strength      = 11.7473579[T] (500[MHz]
X_Acq_Duration      = 0.19111936[s]
X_Domain            = 1H
X_Freq              = 500.15991521[MHz]
X_Offset            = 4.16000426[ppm]
X_Points            = 1024
X_Prescans          = 4
X_Resolution        = 5.2323323[Hz]
X_Sweep             = 5.35790827[kHz]
X_Sweep_Clipped     = 4.28632662[kHz]
Y_Domain            = 13C
Y_Freq              = 125.76529768[MHz]
Y_Offset            = 86.21235061[ppm]
Y_Points            = 256
Y_Prescans          = 0
Y_Resolution        = 92.65298861[Hz]
Y_Sweep             = 23.71916509[kHz]
Tri_Domain          = Proton
Tri_Freq            = 500.15991521[MHz]
Tri_Offset          = 5.0[ppm]
Clipped             = FALSE
Scans               = 4
Total_Scans         = 1024

Relaxation_Delay    = 1.5[s]
Recvr_Gain          = 50
Temp_Get            = 21.5[dC]
X_Acq_Time          = 0.19111936[s]
X_Atn               = 3.2[dB]
X_Gamma             = 42576375
X_Pulse             = 7.68[us]
```

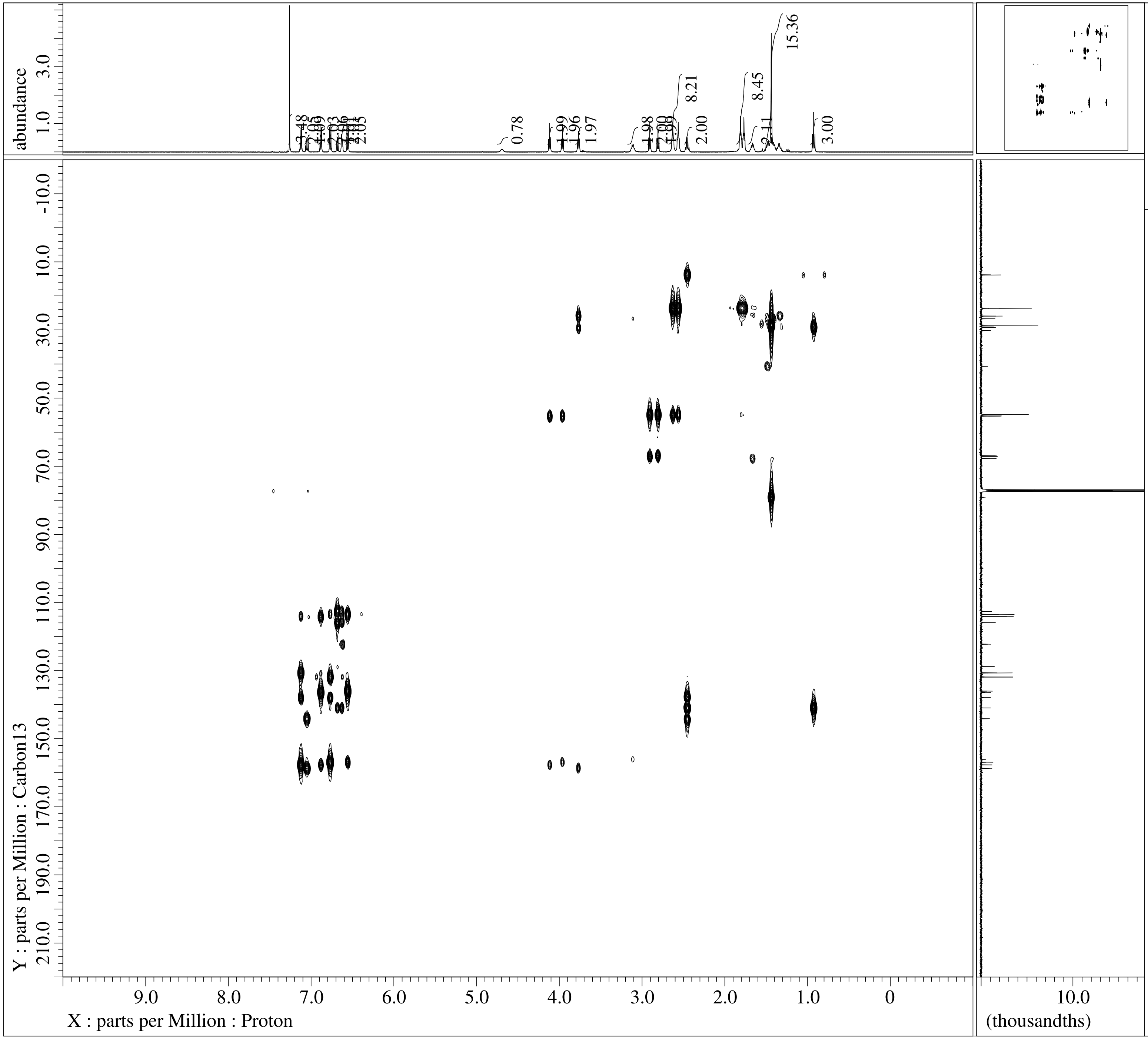


```
---- PROCESSING PARAMETERS ----
gauss( 5.0[Hz], 0.0[s] )
sinbell_auto
zerofill( 1 )
fft( 1, TRUE, TRUE )
ppm
[transpose]
sinbell4( -60, 160 )
trapezoid( 0[%], 5[%], 80[%], 100[%] )
zerofill( 2 )
fft( 1, TRUE, TRUE )
```

```
Filename            = 1_HMBC-1-4.jdf
Author              = delta
Experiment          = hmbc.jxp
Sample_Id           = TK-2-147 data
Solvent             = CHLOROFORM-D
Creation_Time       = 30-JAN-2020 12:24:12
Revision_Time       = 17-JUN-2024 19:58:32
Current_Time        = 17-JUN-2024 20:00:27

Comment             = gradient enhanced HMBC
Data_Format         = 2D REAL REAL
Dim_Size            = 1638, 512
Dim_Title           = Proton Carbon13
Dim_Units           = [ppm] [ppm]
Dimensions          = X Y
Site                = JNM-ECA500II
Spectrometer        = DELTA2_NMR

Field_Strength      = 11.7473579[T] (500[MHz]
X_Acq_Duration      = 0.21823488[s]
X_Domain            = 1H
X_Freq              = 500.15991521[MHz]
X_Offset            = 5.0[ppm]
X_Points            = 2048
X_Prescans          = 4
X_Resolution        = 4.58221894[Hz]
X_Sweep             = 9.38438438[kHz]
X_Sweep_Clipped     = 7.50750751[kHz]
Y_Domain            = 13C
Y_Freq              = 125.76529768[MHz]
Y_Offset            = 100[ppm]
Y_Points            = 256
Y_Prescans          = 0
Y_Resolution        = 122.99275819[Hz]
Y_Sweep             = 31.4861461[kHz]
Tri_Domain          = Proton
Tri_Freq            = 500.15991521[MHz]
Tri_Offset          = 5.0[ppm]
Clipped             = FALSE
Scans               = 8
Total_Scans         = 2048

Relaxation_Delay    = 1.5[s]
Recvr_Gain          = 50
Temp_Get            = 21.8[dC]
X_Acq_Time          = 0.21823488[s]
X_Atn               = 3.2[dB]
X_Gamma             = 42576375
X_Pulse             = 7.68[us]
```

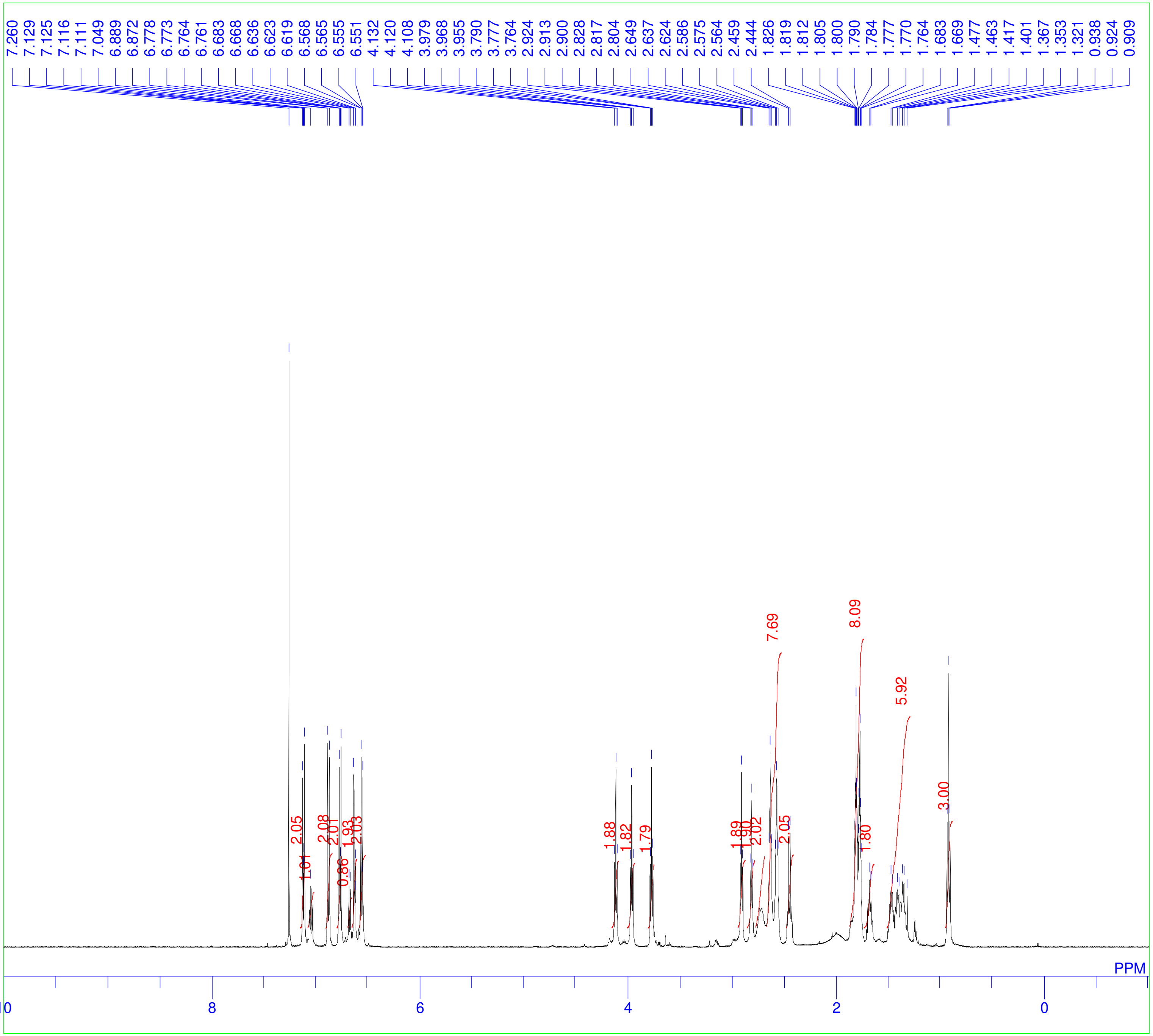

DFILE 2_proton.als
COMNT
DATIM 2020-02-08 14:49:33
OBNUC 1H
EXMOD proton.jxp
OBFRQ 500.16 MHz
OBSET 2.41 KHz
OBFIN 6.01 Hz
POINT 13107
FREQU 7507.51 Hz
SCANS 8
ACQTM 1.7459 sec
PD 5.0000 sec
PW1 3.84 usec
IRNUC 1H
CTEMP 19.9 c
SLVNT CDCL3
EXREF 7.26 ppm
BF 0.12 Hz
RGAIN 38

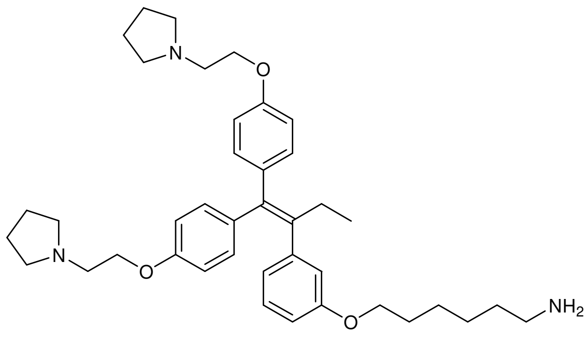

2

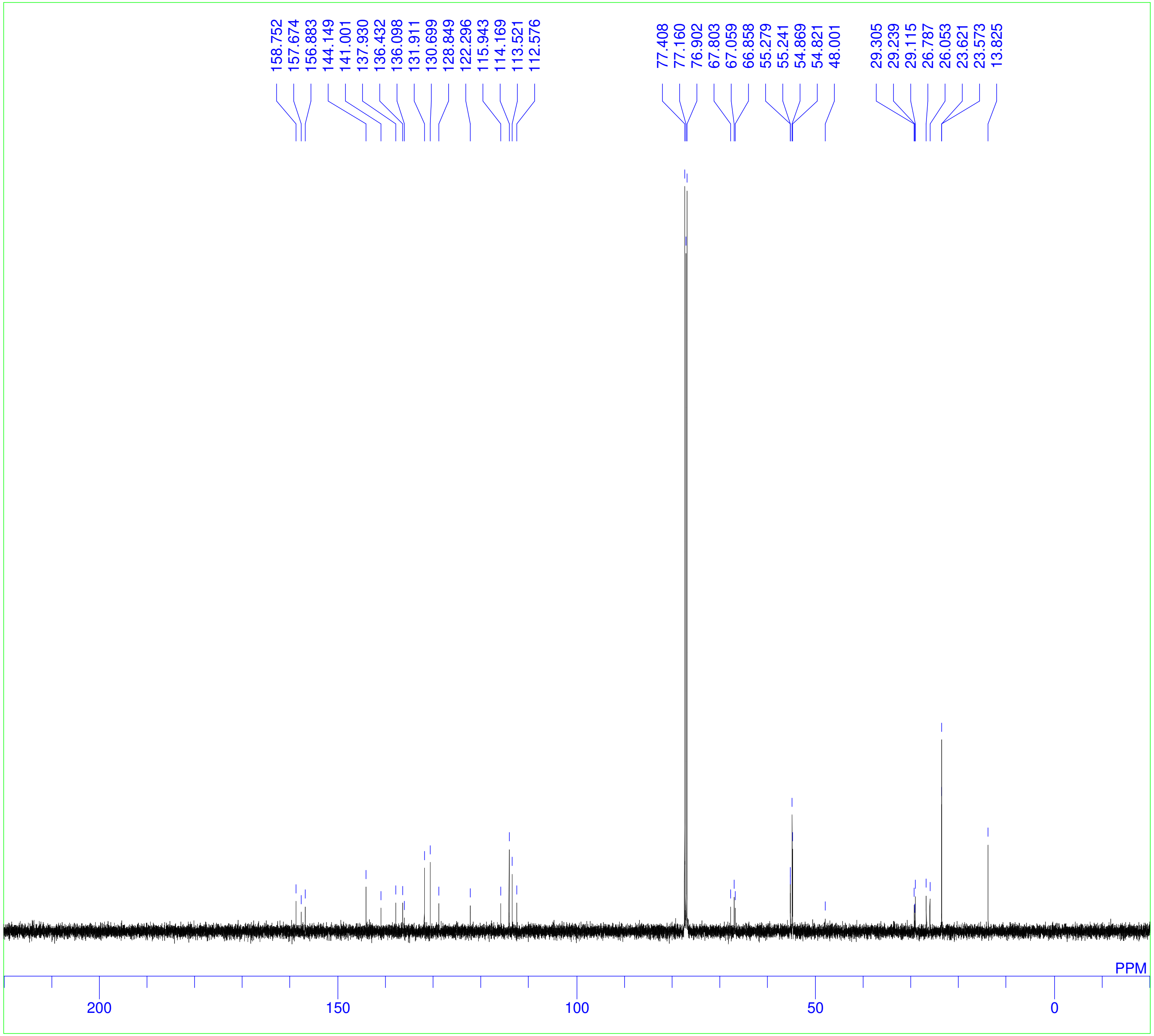

DFILE	2_carbon.als
COMNT	
DATIM	2020-02-08 14:51:23
OBNUC	13C
EXMOD	carbon.jxp
OBFRQ	125.77 MHz
OBSET	7.87 KHz
OBFIN	4.21 Hz
POINT	26214
FREQU	31446.54 Hz
SCANS	1024
ACQTM	0.8336 sec
PD	2.0000 sec
PW1	3.87 usec
IRNUC	1H
CTEMP	20.3 c
SLVNT	CDCL3
EXREF	77.16 ppm
BF	0.12 Hz
RGAIN	26

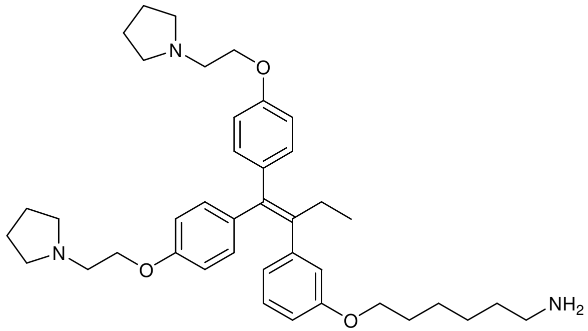

2

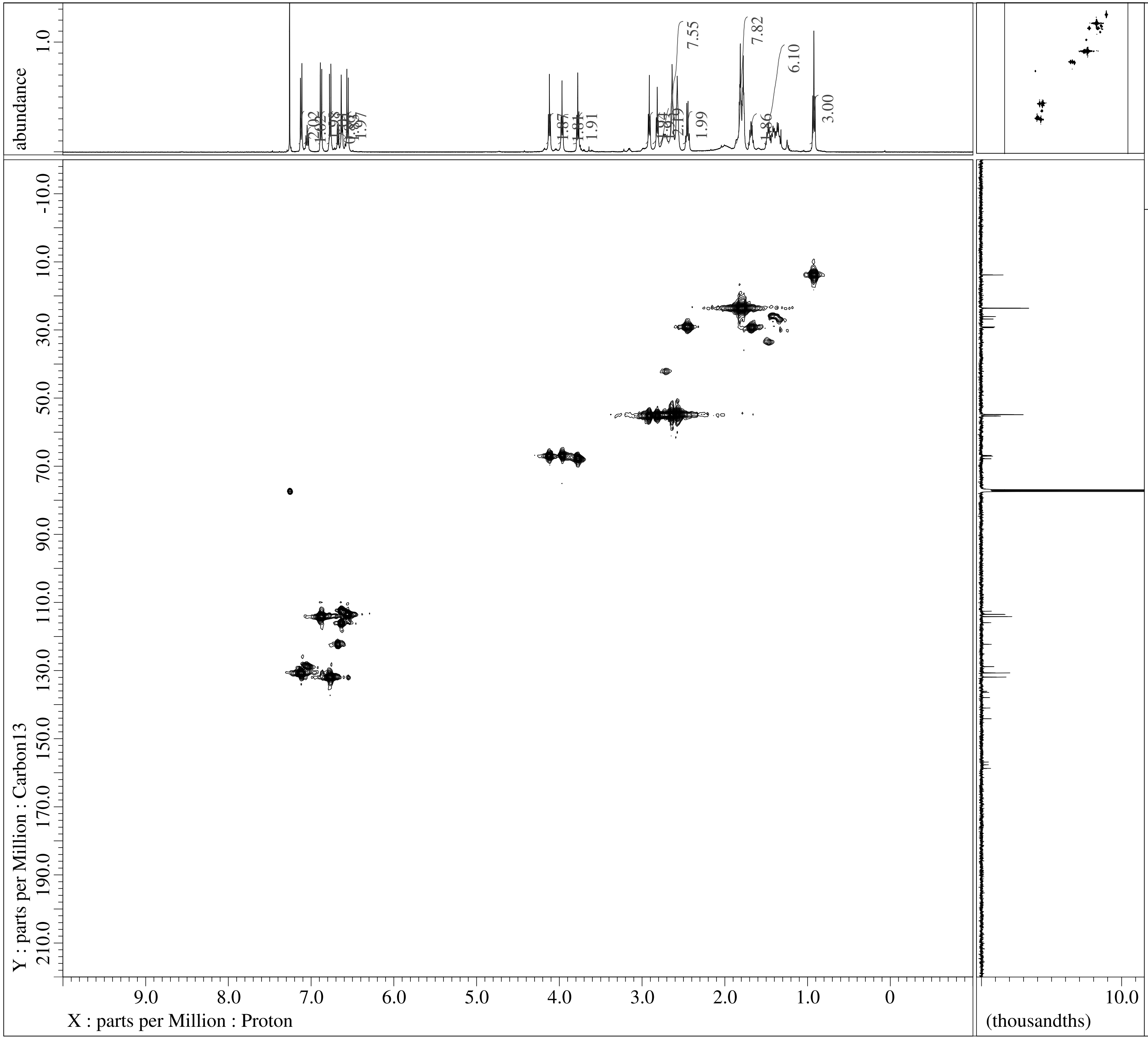


```
---- PROCESSING PARAMETERS ----
dc_balance( 0, FALSE )
sexp( 2.0[Hz], 0.0[s] )
trapezoid( 0[%], 0[%], 80[%], 100[%] )
zerofill( 1, TRUE )
fft( 1, TRUE, TRUE )
machinephase
ppm

Filename             = 2_HMQC-1-4.jdf
Author               = delta
Experiment           = hmqc.jxp
Sample_Id            = TK-2-174 data
Solvent              = CHLOROFORM-D
Creation_Time        =  8-FEB-2020 16:58:30
Revision_Time        = 17-JUN-2024 20:06:11
Current_Time         = 17-JUN-2024 20:07:45

Comment              = gradient enhanced HMQC
Data_Format          = 2D REAL REAL
Dim_Size             = 819, 512
Dim_Title            = Proton Carbon13
Dim_Units            = [ppm] [ppm]
Dimensions           = X Y
Site                 = JNM-ECA500II
Spectrometer         = DELTA2_NMR

Field_Strength       = 11.7473579[T] (500[MHz]
X_Acq_Duration       = 0.10911744[s]
X_Domain             = 1H
X_Freq               = 500.15991521[MHz]
X_Offset             = 5.0[ppm]
X_Points             = 1024
X_Prescans           = 4
X_Resolution         = 9.16443788[Hz]
X_Sweep              = 9.38438438[kHz]
X_Sweep_Clipped      = 7.50750751[kHz]
Y_Domain             = 13C
Y_Freq               = 125.76529768[MHz]
Y_Offset             = 85[ppm]
Y_Points             = 256
Y_Prescans           = 0
Y_Resolution         = 83.60980308[Hz]
Y_Sweep              = 21.40410959[kHz]
Tri_Domain           = Proton
Tri_Freq             = 500.15991521[MHz]
Tri_Offset           = 5.0[ppm]
Clipped              = FALSE
Scans                = 4
Total_Scans          = 1024

Relaxation_Delay     = 1.5[s]
Recvr_Gain           = 50
Temp_Get             = 20.5[dC]
X_Acq_Time           = 0.10911744[s]
X_Atn                = 3.2[dB]
X_Gamma              = 42576375
X_Pulse              = 7.68[us]
```

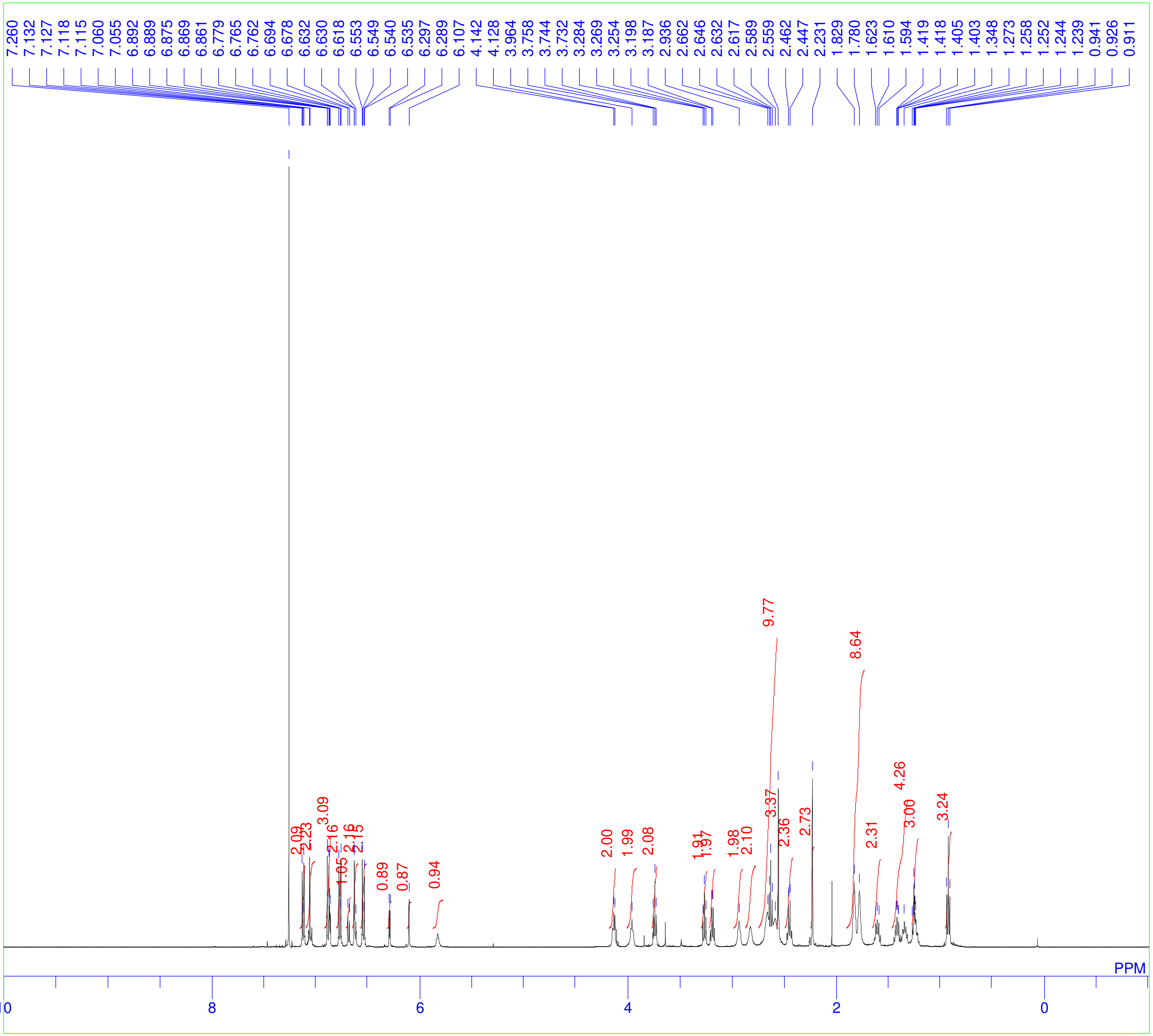

DFILE	RID-B-BODIPY_Proton.als
COMNT	
DATIM	2020-02-22 17:08:21
OBNUC	1H
EXMOD	proton.jxp
OBFRQ	500.16 MHz
OBSET	2.41 KHz
OBFIN	6.01 Hz
POINT	13107
FREQU	7507.51 Hz
SCANS	8
ACQTM	1.7459 sec
PD	5.0000 sec
PW1	3.84 usec
IRNUC	1H
CTEMP	22.6 c
SLVNT	CDCL3
EXREF	7.26 ppm
BF	0.12 Hz
RGAIN	40

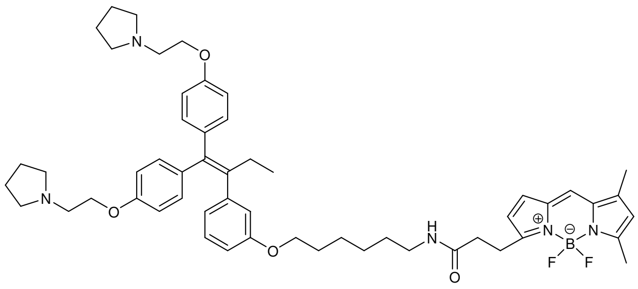

RID-B-BODIPY

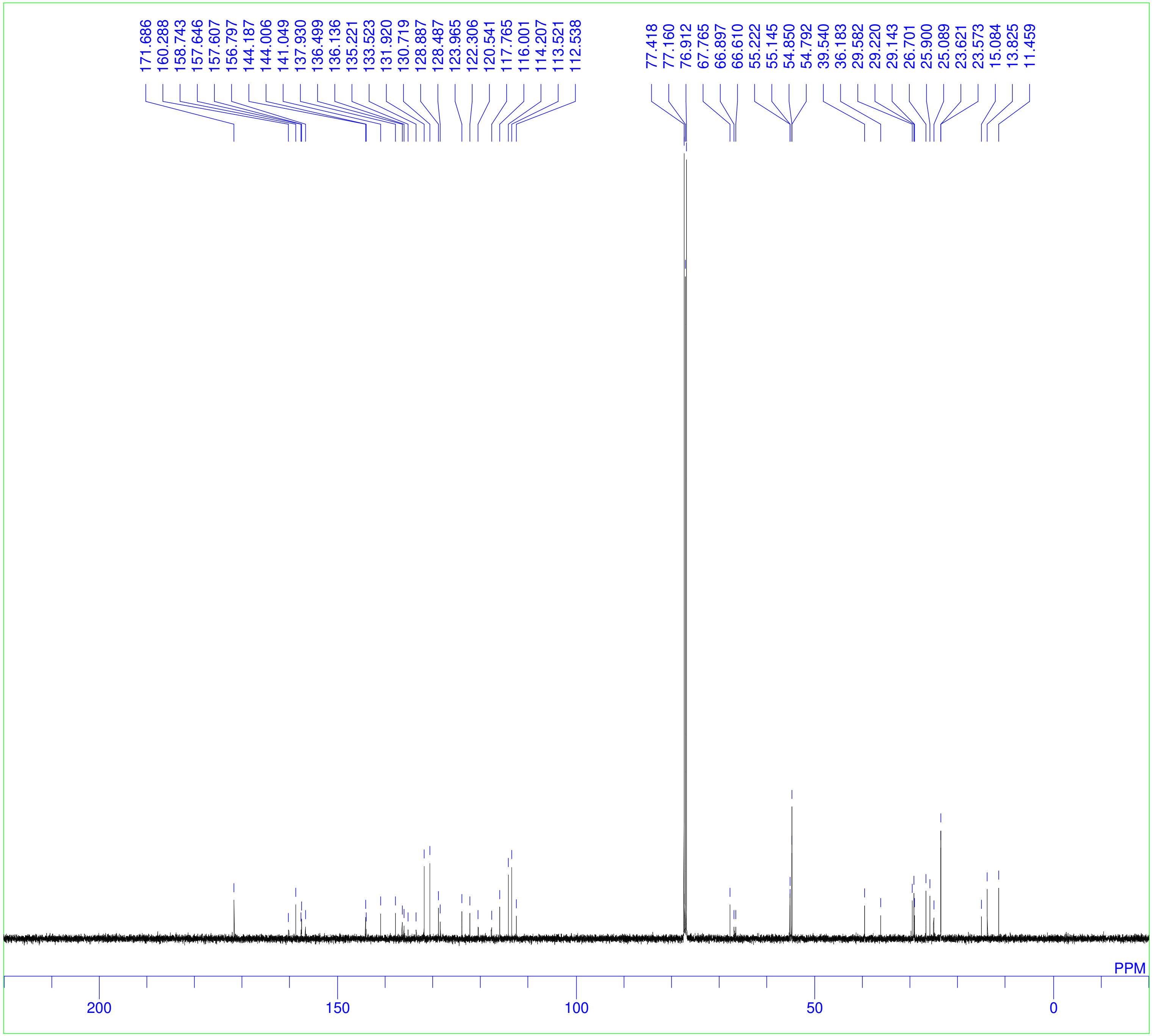

DFILE	RID-B-BODIPY_Carbon.als
COMNT	
DATIM	2020-03-20 12:02:09
OBNUC	13C
EXMOD	carbon.jxp
OBFRQ	125.77 MHz
OBSET	7.87 KHz
OBFIN	4.21 Hz
POINT	26214
FREQU	31446.54 Hz
SCANS	1800
ACQTM	0.8336 sec
PD	2.0000 sec
PW1	3.87 usec
IRNUC	1H
CTEMP	22.9 c
SLVNT	CDCL3
EXREF	77.16 ppm
BF	0.25 Hz
RGAIN	30

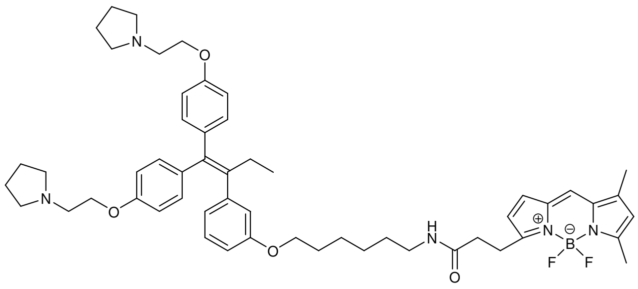

RID-B-BODIPY

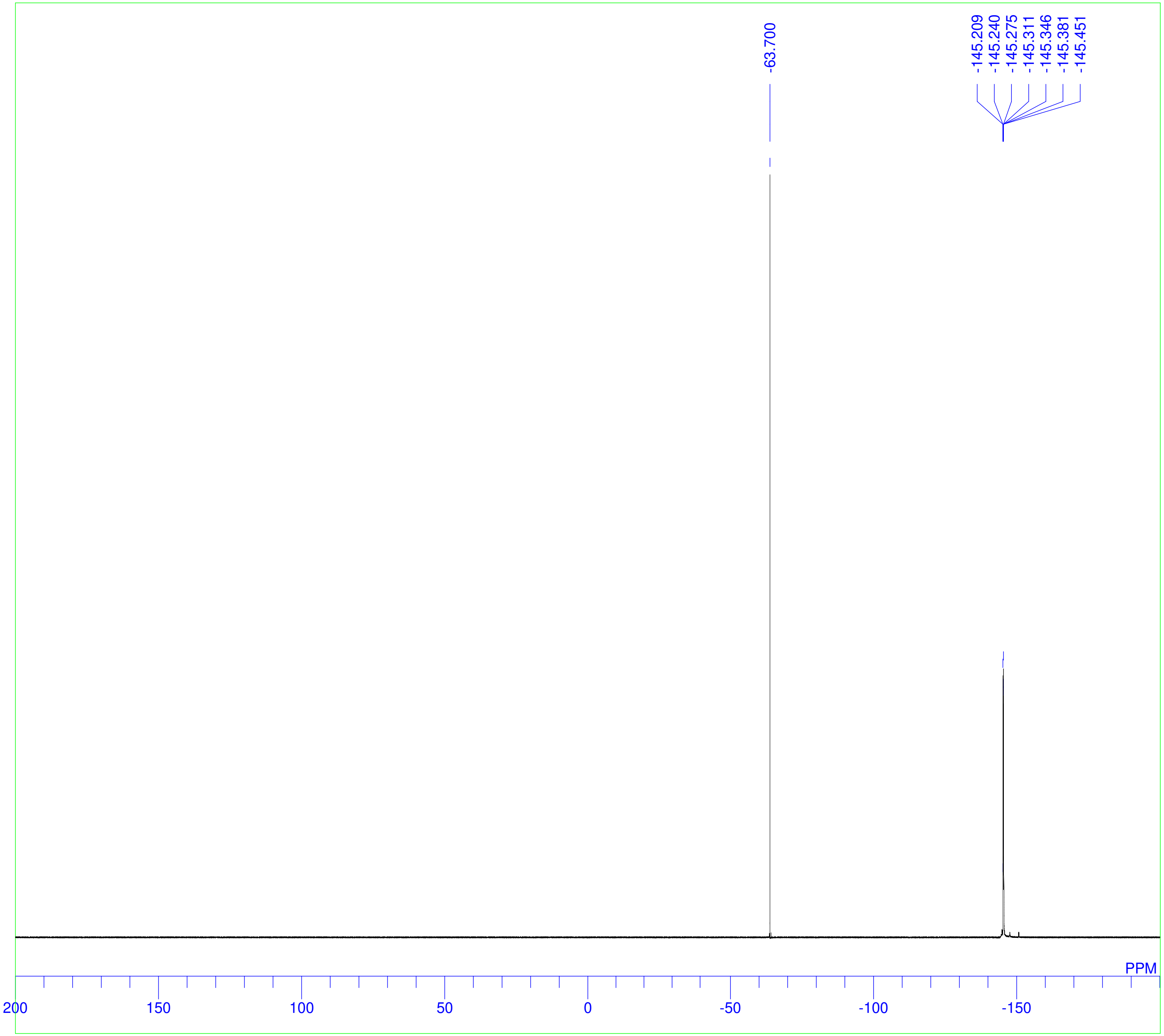

DFILE	RID-B-BODIPY_Fluorine.als
COMNT	
DATIM	2024-06-12 20:38:12
OBNUC	19F
EXMOD	proton.jxp
OBFRQ	470.62 MHz
OBSET	0.46 KHz
OBFIN	0.84 Hz
POINT	104857
FREQU	192307.69 Hz
SCANS	512
ACQTM	0.5453 sec
PD	5.0000 sec
PW1	4.25 usec
IRNUC	19F
CTEMP	24.0 c
SLVNT	CDCL3
EXREF	-63.70 ppm
BF	0.30 Hz
RGAIN	44

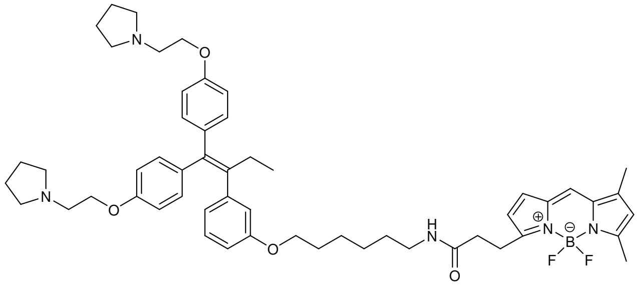

RID-B-BODIPY

Trifluoromethylbenzene as an internal standard

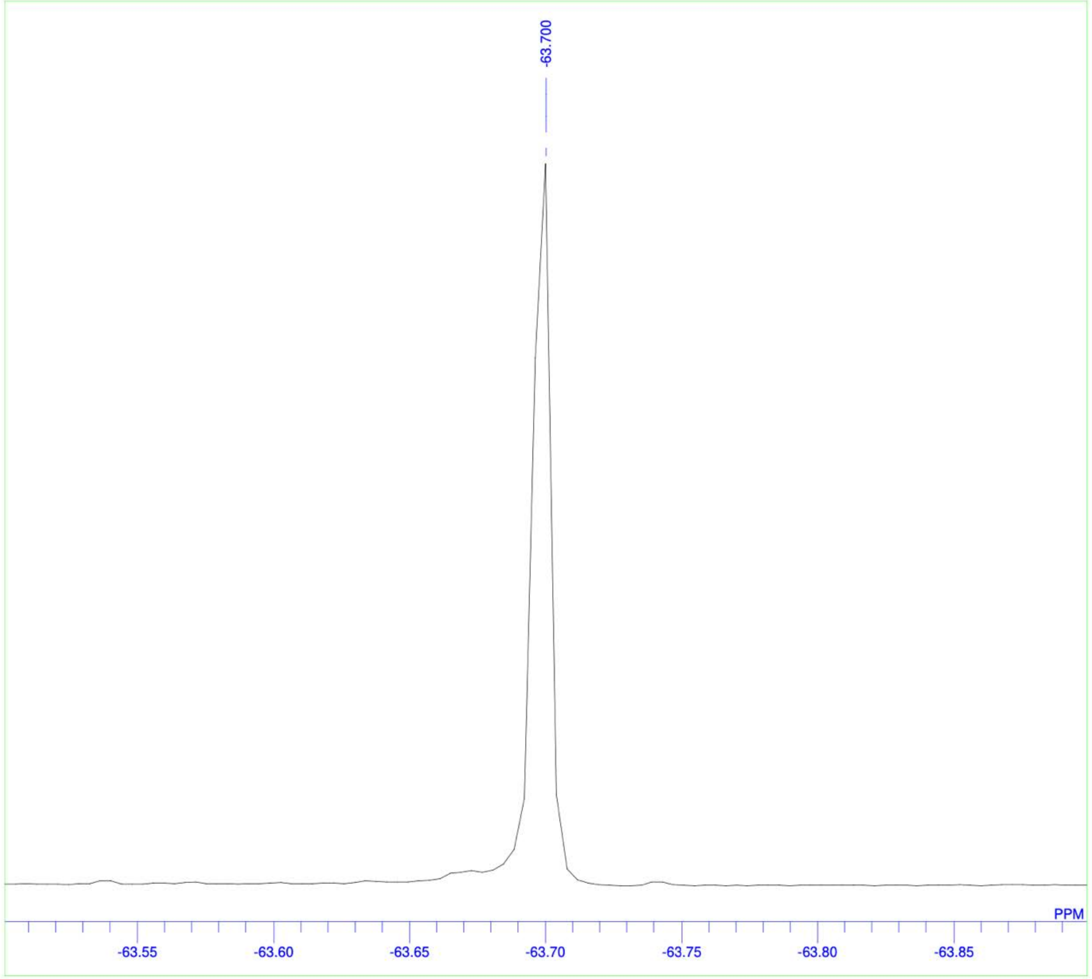

Fluorine of **RID-B-BODIPY**

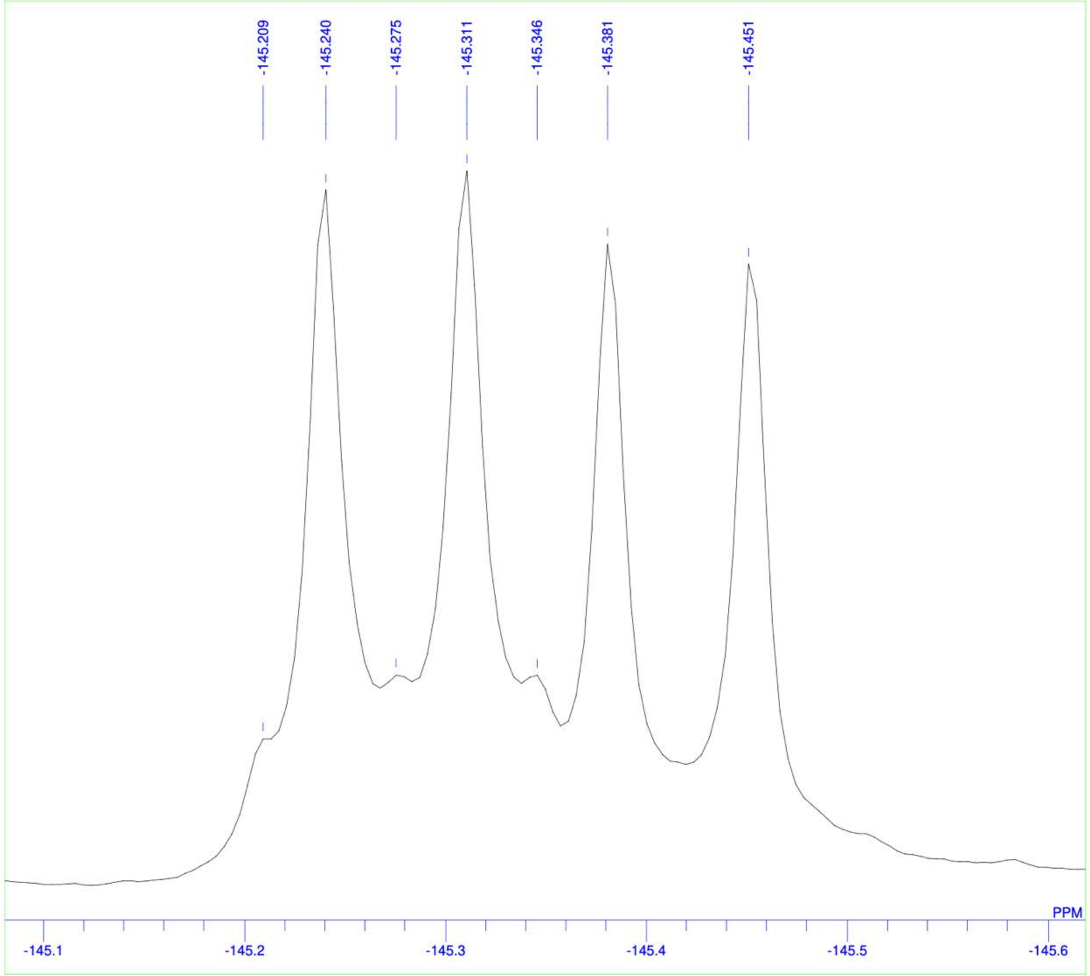

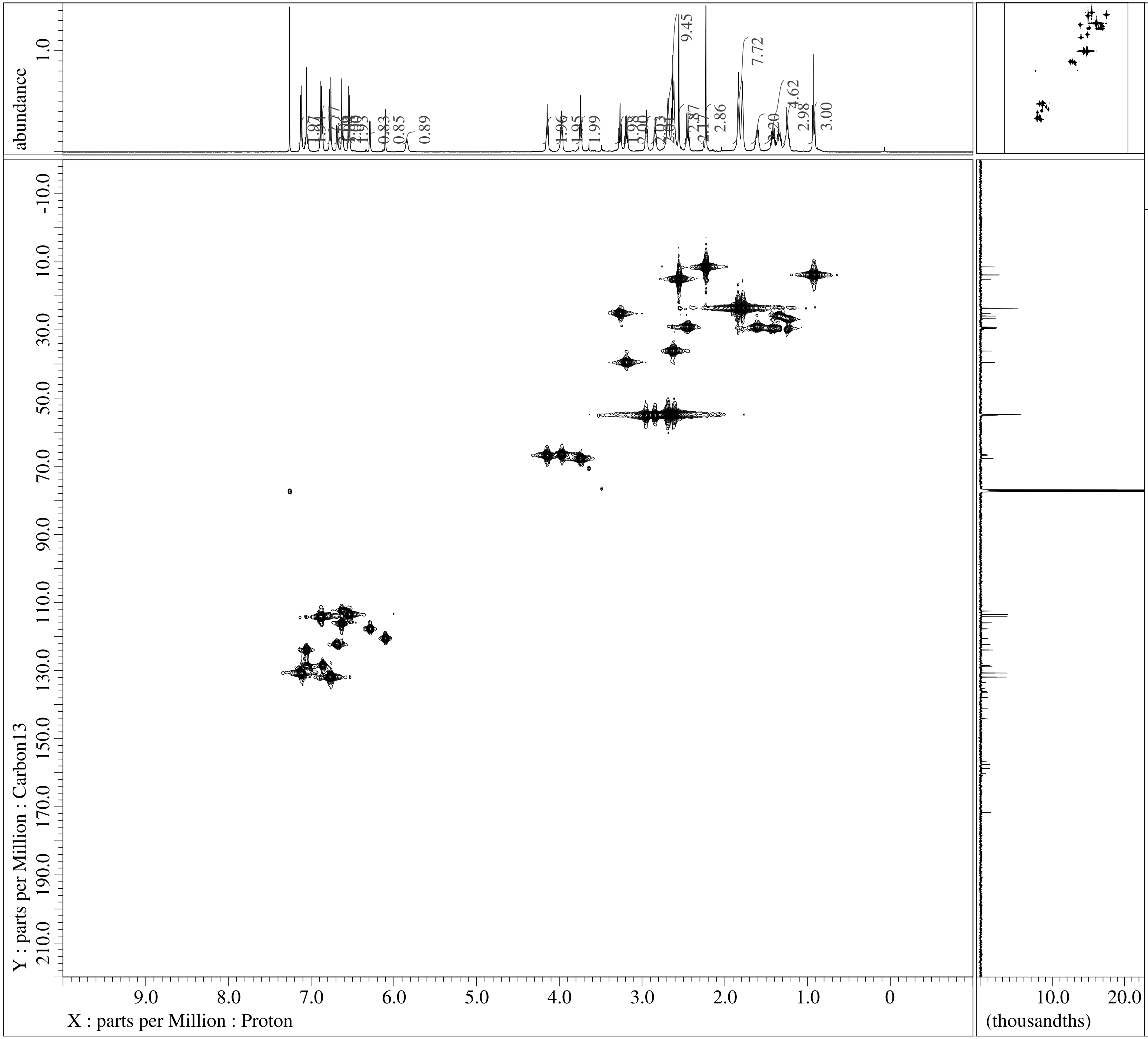


```
---- PROCESSING PARAMETERS ----
dc_balance( 0, FALSE )
sexp( 2.0[Hz], 0.0[s] )
trapezoid( 0[%], 0[%], 80[%], 100[%] )
zerofill( 1 )
fft( 1, TRUE, TRUE )
machinephase
ppm

Filename            = RID-B-BODIPY_HMQC-1-4.j
Author              = delta
Experiment          = hmqc.jxp
Sample_Id           = TK-2-96-data
Solvent             = CHLOROFORM-D
Creation_Time       = 20-MAR-2020 19:58:52
Revision_Time       = 17-JUN-2024 20:09:38
Current_Time        = 17-JUN-2024 20:11:02

Comment             = gradient enhanced HMQC
Data_Format         = 2D REAL REAL
Dim_Size            = 819, 512
Dim_Title           = Proton Carbon13
Dim_Units           = [ppm] [ppm]
Dimensions          = X Y
Site                = JNM-ECA500II
Spectrometer        = DELTA2_NMR

Field_Strength      = 11.7473579[T] (500[MHz]
X_Acq_Duration      = 0.10911744[s]
X_Domain            = 1H
X_Freq              = 500.15991521[MHz]
X_Offset            = 5.0[ppm]
X_Points            = 1024
X_Prescans          = 4
X_Resolution        = 9.16443788[Hz]
X_Sweep             = 9.38438438[kHz]
X_Sweep_Clipped     = 7.50750751[kHz]
Y_Domain            = 13C
Y_Freq              = 125.76529768[MHz]
Y_Offset            = 85[ppm]
Y_Points            = 256
Y_Prescans          = 0
Y_Resolution        = 83.60980308[Hz]
Y_Sweep             = 21.40410959[kHz]
Tri_Domain          = Proton
Tri_Freq            = 500.15991521[MHz]
Tri_Offset          = 5.0[ppm]
Clipped             = FALSE
Scans               = 4
Total_Scans         = 1024

Relaxation_Delay    = 1.5[s]
Recvr_Gain          = 50
Temp_Get            = 22.7[dC]
X_Acq_Time          = 0.10911744[s]
X_Atn               = 3.2[dB]
X_Gamma             = 42576375
X_Pulse             = 7.68[us]
```

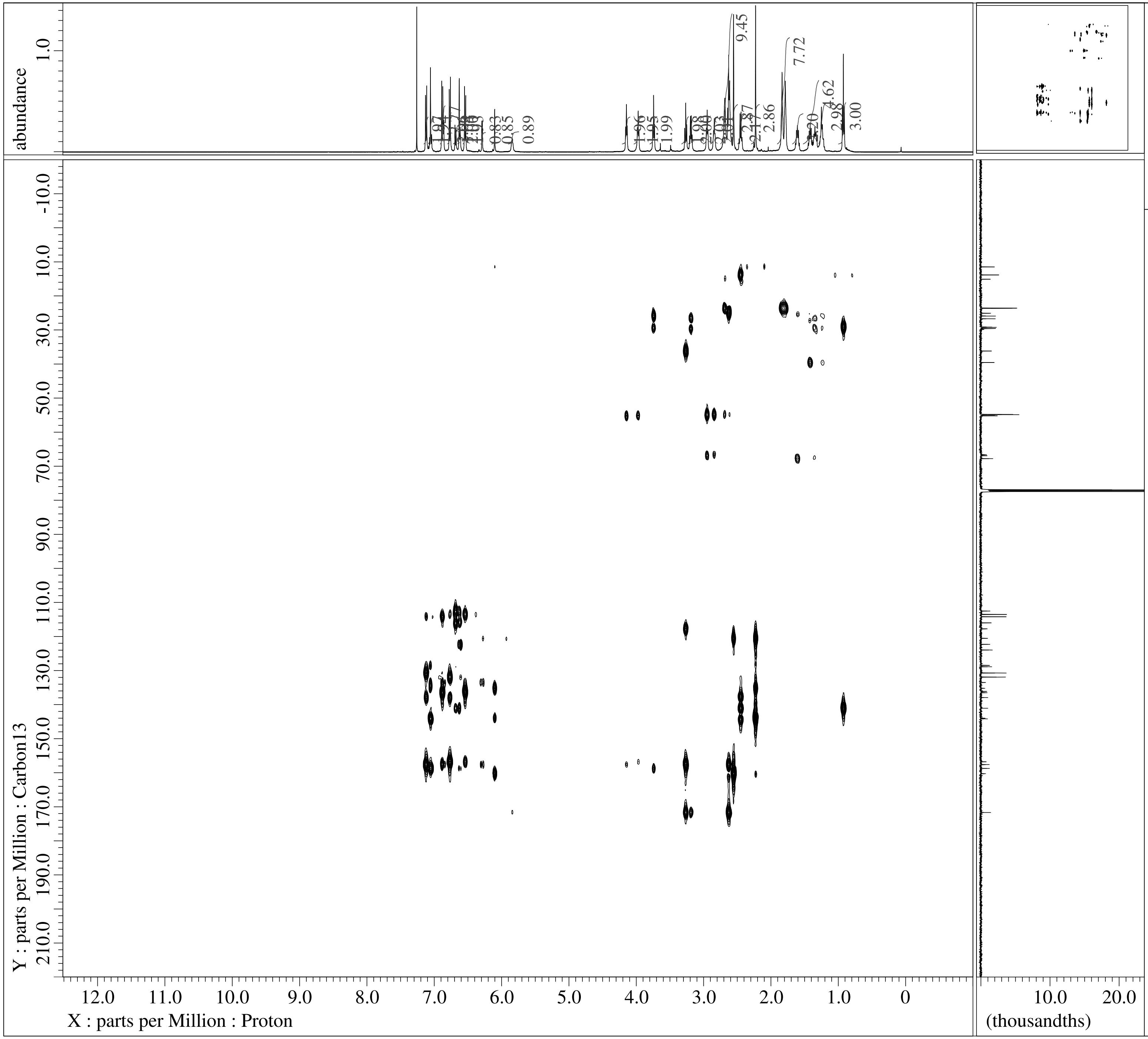


```
---- PROCESSING PARAMETERS ----
dc_balance( 0, FALSE )
sexp( 2.0[Hz], 0.0[s] )
trapezoid( 0[%], 0[%], 80[%], 100[%] )
zerofill( 1 )
fft( 1, TRUE, TRUE )
machinephase
ppm

Filename           = RID-B-BODIPY_HMBC-1-4.j
Author             = delta
Experiment         = hmbc.jxp
Sample_Id          = TK-2-96-data
Solvent            = CHLOROFORM-D
Creation_Time      = 20-MAR-2020 20:27:15
Revision_Time      = 17-JUN-2024 20:13:51
Current_Time       = 17-JUN-2024 20:14:45

Comment            = gradient enhanced HMBC
Data_Format        = 2D REAL REAL
Dim_Size           = 1638, 512
Dim_Title          = Proton Carbon13
Dim_Units          = [ppm] [ppm]
Dimensions         = X Y
Site               = JNM-ECA500II
Spectrometer       = DELTA2_NMR

Field_Strength     = 11.7473579[T] (500[MHz]
X_Acq_Duration     = 0.21823488[s]
X_Domain           = 1H
X_Freq             = 500.15991521[MHz]
X_Offset           = 5.0[ppm]
X_Points           = 2048
X_Prescans         = 4
X_Resolution       = 4.58221894[Hz]
X_Sweep            = 9.38438438[kHz]
X_Sweep_Clipped    = 7.50750751[kHz]
Y_Domain           = 13C
Y_Freq             = 125.76529768[MHz]
Y_Offset           = 100[ppm]
Y_Points           = 256
Y_Prescans         = 0
Y_Resolution       = 122.99275819[Hz]
Y_Sweep            = 31.4861461[kHz]
Tri_Domain         = Proton
Tri_Freq           = 500.15991521[MHz]
Tri_Offset         = 5.0[ppm]
Clipped            = FALSE
Scans              = 8
Total_Scans        = 2048

Relaxation_Delay   = 1.5[s]
Recvr_Gain         = 50
Temp_Get           = 22.6[dC]
X_Acq_Time         = 0.21823488[s]
X_Atn              = 3.2[dB]
X_Gamma            = 42576375
X_Pulse            = 7.68[us]
```

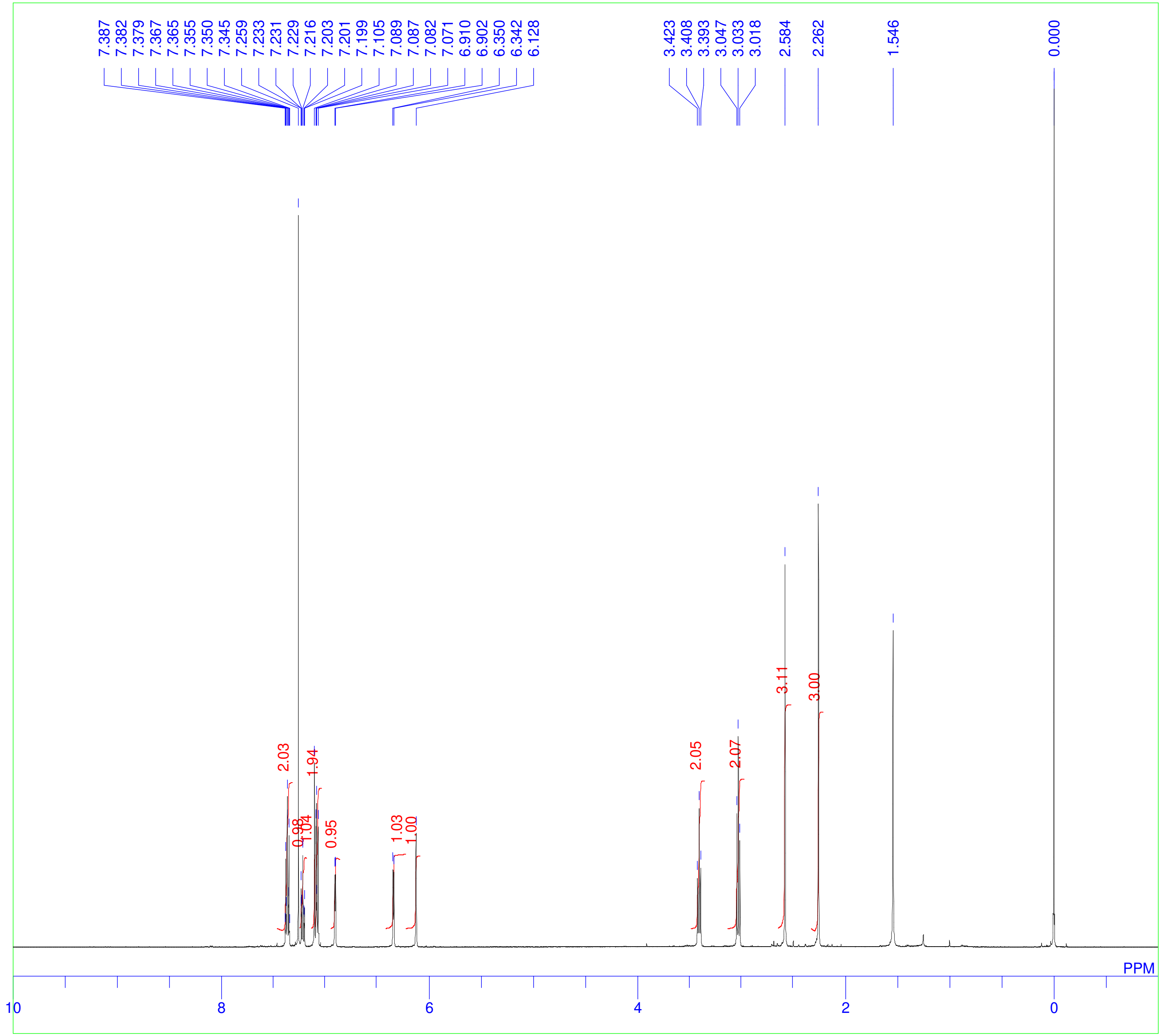

DFILE Phenol-BODIPY_Proton.als
COMNT
DATIM 2024-06-04 14:12:42
OBNUC 1H
EXMOD proton.jxp
OBFRQ 500.16 MHz
OBSET 2.41 KHz
OBFIN 6.01 Hz
POINT 13107
FREQU 7507.51 Hz
SCANS 8
ACQTM 1.7459 sec
PD 5.0000 sec
PW1 3.80 usec
IRNUC 1H
CTEMP 24.0 c
SLVNT CDCL3
EXREF 0.00 ppm
BF 0.30 Hz
RGAIN 46

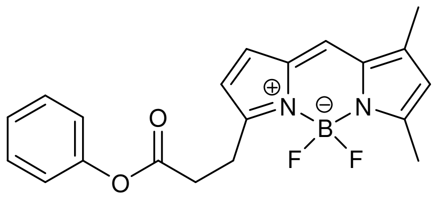

Phenol-BODIPY

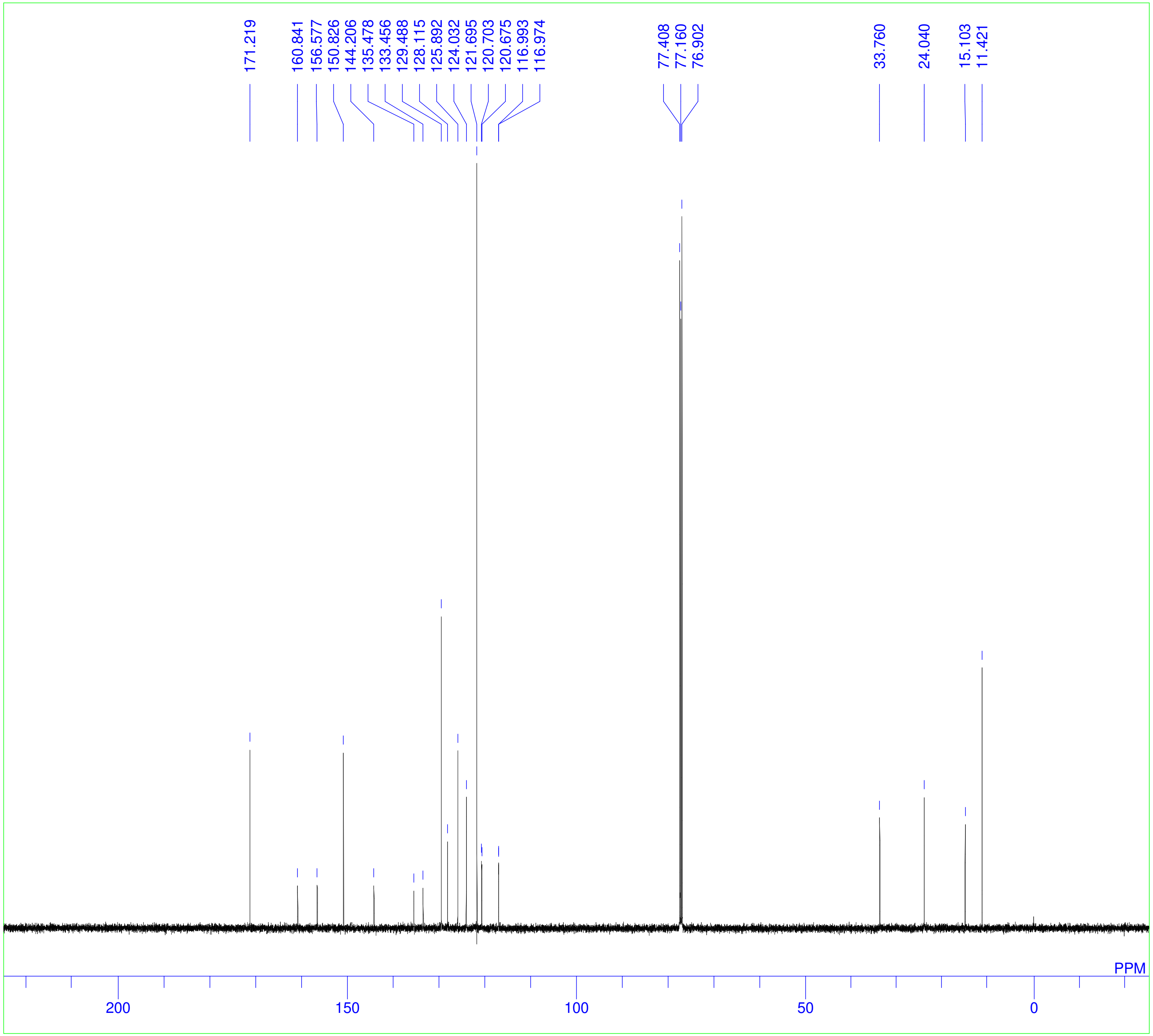

DFILE	Phenol-BODIPY_Carbon.als
COMNT	
DATIM	2024-06-04 17:33:28
OBNUC	13C
EXMOD	carbon.jxp
OBFRQ	125.77 MHz
OBSET	7.87 KHz
OBFIN	4.21 Hz
POINT	26214
FREQU	31446.54 Hz
SCANS	1024
ACQTM	0.8336 sec
PD	2.0000 sec
PW1	4.30 usec
IRNUC	1H
CTEMP	24.0 c
SLVNT	CDCL3
EXREF	77.16 ppm
BF	0.30 Hz
RGAIN	36

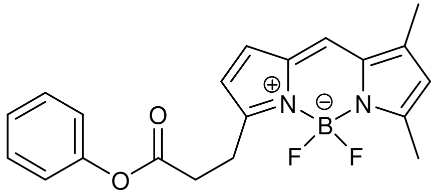

Phenol-BODIPY

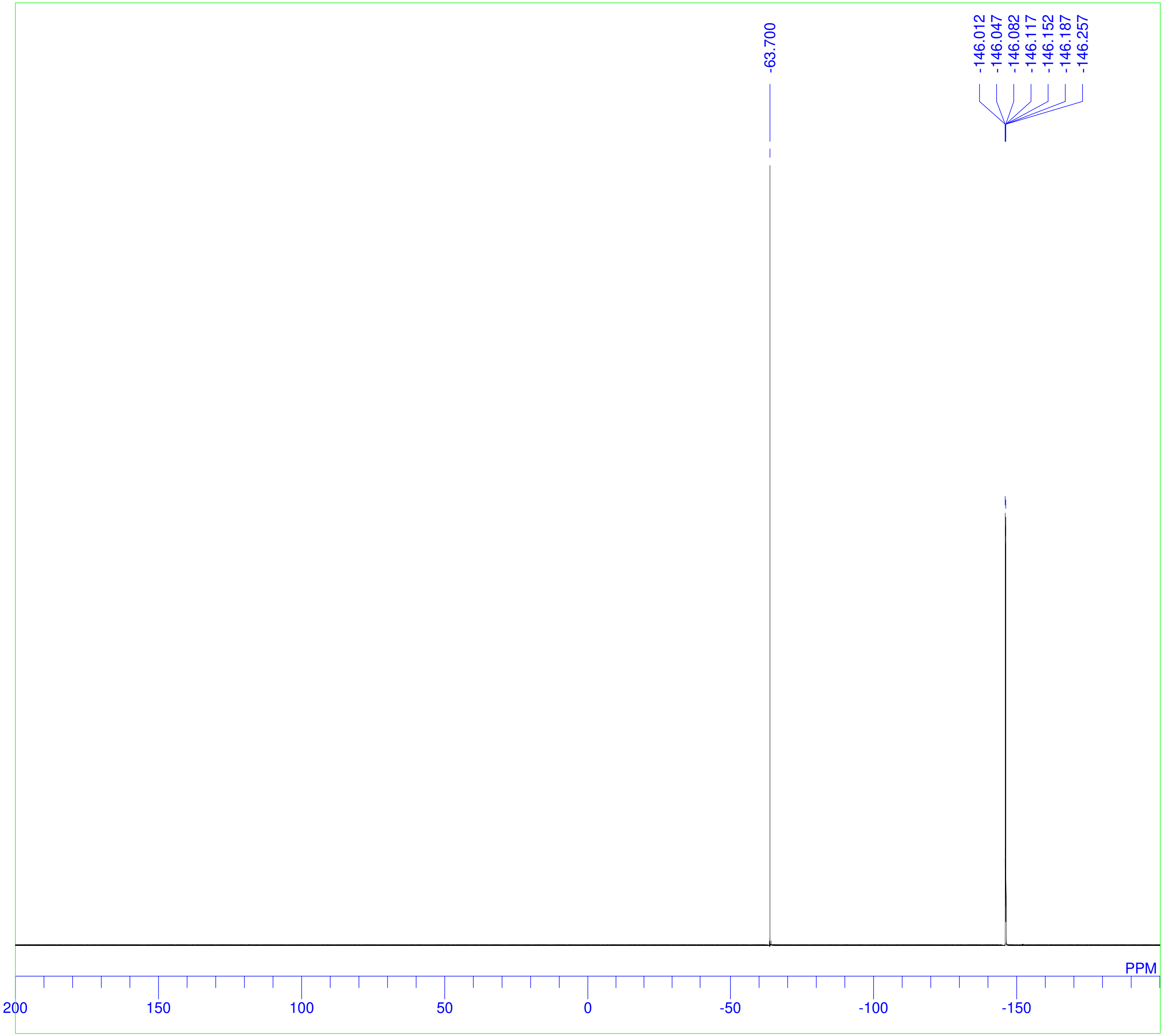

DFILE	Phenol-BODIPY_Fluorine.als
COMNT	
DATIM	2024-06-11 15:21:49
OBNUC	19F
EXMOD	proton.jxp
OBFRQ	470.62 MHz
OBSET	0.46 KHz
OBFIN	0.84 Hz
POINT	104857
FREQU	192307.69 Hz
SCANS	128
ACQTM	0.5453 sec
PD	5.0000 sec
PW1	4.25 usec
IRNUC	19F
CTEMP	23.9 c
SLVNT	CDCL3
EXREF	-63.70 ppm
BF	0.30 Hz
RGAIN	44

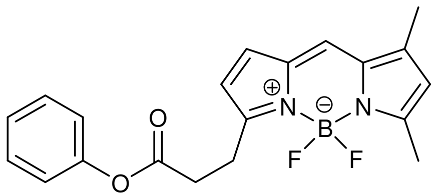

Phenol-BODIPY

Trifluoromethylbenzene as an internal standard

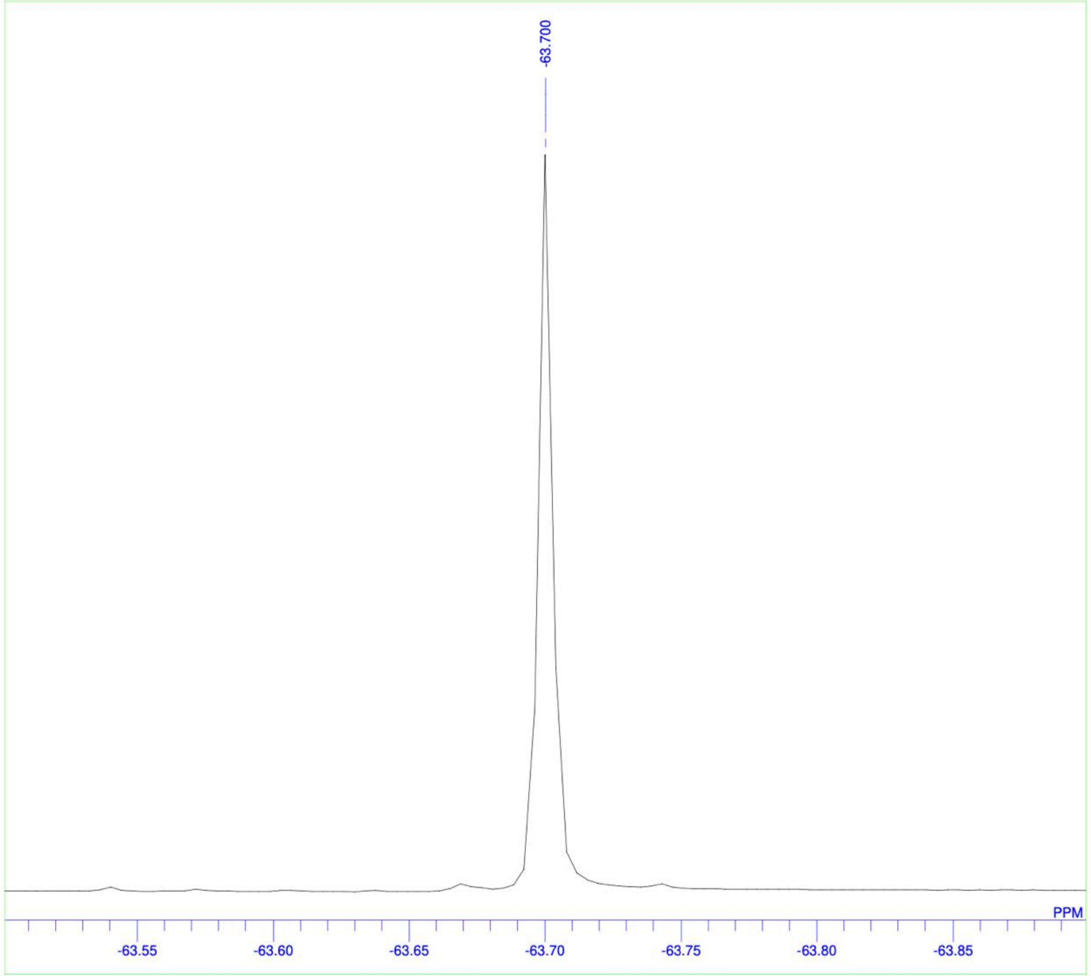

Fluorine of **Phenol-BODIPY**

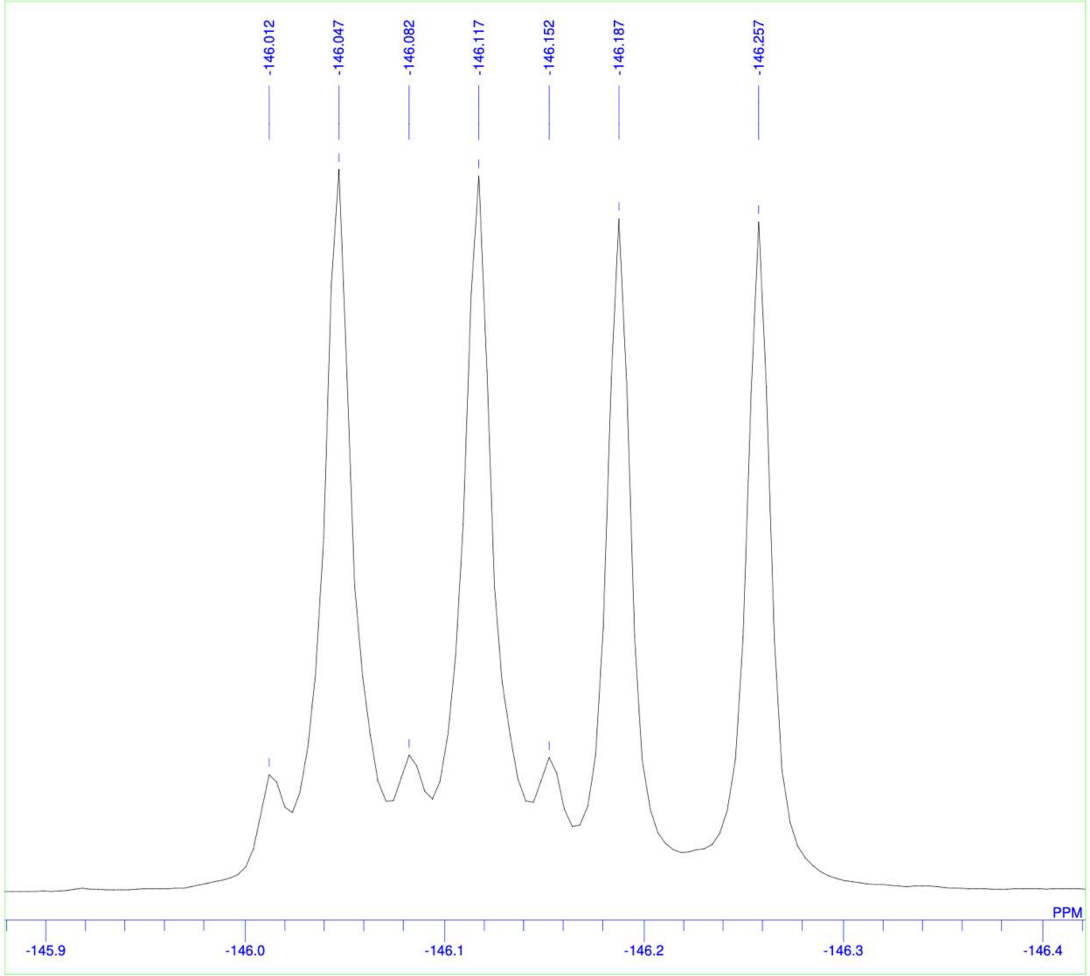

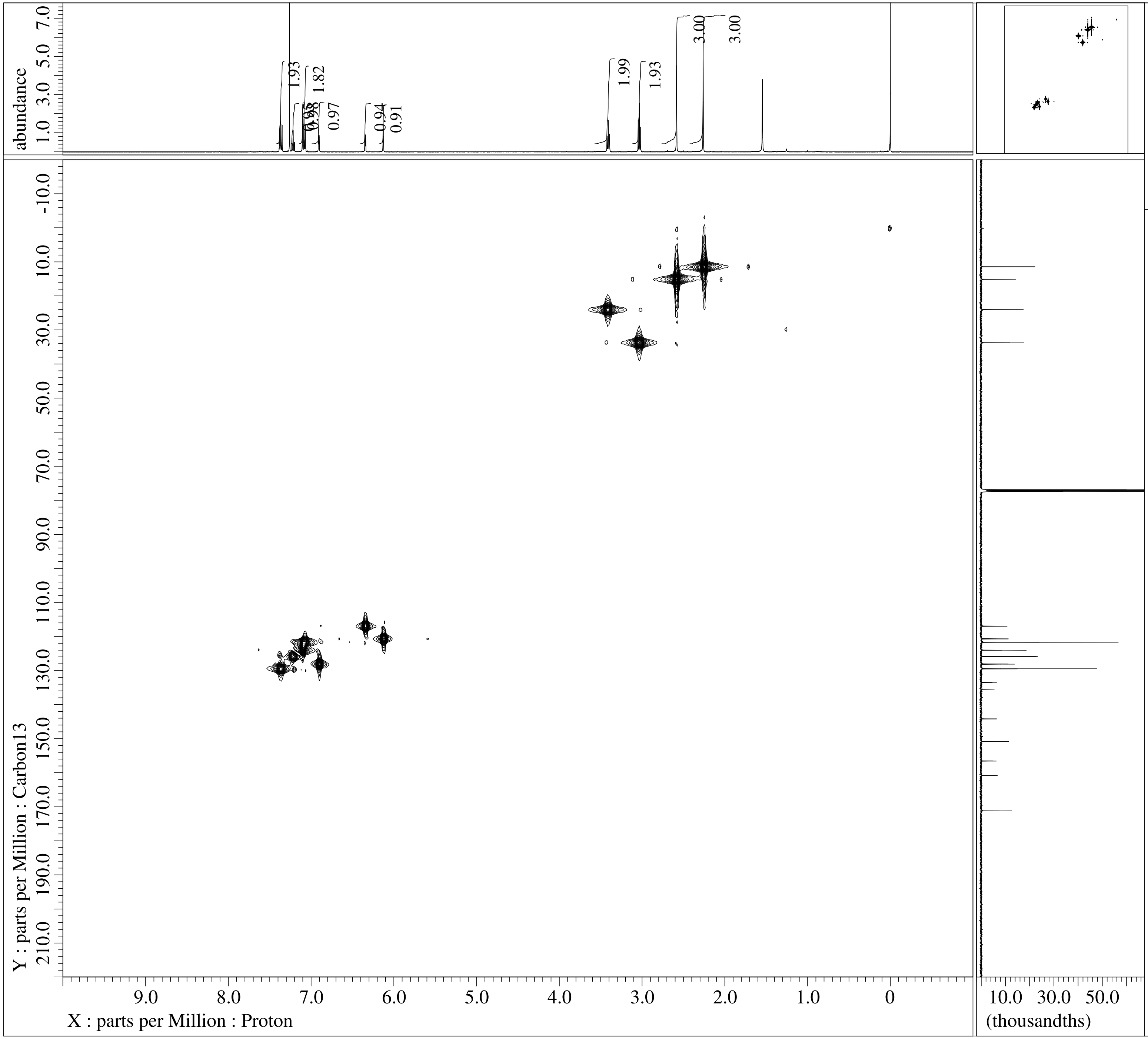


```
---- PROCESSING PARAMETERS ----
dc_balance( 0, FALSE )
sexp( 2.0[Hz], 0.0[s] )
trapezoid( 0[%], 0[%], 80[%], 100[%] )
zerofill( 1 )
fft( 1, TRUE, TRUE )
machinephase
ppm

Filename             = Phenol-BODIPY_HMQC-1-4.
Author               = delta
Experiment           = hmqc.jxp
Sample_Id            = Phenol BODIPY
Solvent              = CHLOROFORM-D
Creation_Time        =  4-JUN-2024 18:22:19
Revision_Time        = 17-JUN-2024 20:17:30
Current_Time         = 17-JUN-2024 20:18:40

Comment              = gradient enhanced HMQC
Data_Format          = 2D REAL REAL
Dim_Size             = 819, 512
Dim_Title            = Proton Carbon13
Dim_Units            = [ppm] [ppm]
Dimensions           = X Y
Site                 = JNM-ECA500II
Spectrometer         = DELTA2_NMR

Field_Strength       = 11.7473579[T] (500[MHz]
X_Acq_Duration       = 0.10911744[s]
X_Domain             = 1H
X_Freq               = 500.15991521[MHz]
X_Offset             = 5.0[ppm]
X_Points             = 1024
X_Prescans           = 4
X_Resolution         = 9.16443788[Hz]
X_Sweep              = 9.38438438[kHz]
X_Sweep_Clipped      = 7.50750751[kHz]
Y_Domain             = 13C
Y_Freq               = 125.76529768[MHz]
Y_Offset             = 85.92143169[ppm]
Y_Points             = 256
Y_Prescans           = 0
Y_Resolution         = 109.23517897[Hz]
Y_Sweep              = 27.96420582[kHz]
Tri_Domain           = Proton
Tri_Freq             = 500.15991521[MHz]
Tri_Offset           = 5.0[ppm]
Clipped              = FALSE
Scans                = 4
Total_Scans          = 1024

Relaxation_Delay     = 1.5[s]
Recvr_Gain           = 50
Temp_Get             = 23.8[dC]
X_Acq_Time           = 0.10911744[s]
X_Atn                = 3.2[dB]
X_Gamma              = 42576375
X_Pulse              = 7.59[us]
```
